# Chiral phosphoric acid catalyzed aminative dearomatization of *α*-naphthols/Michael addition sequence

**DOI:** 10.1038/s41467-019-11109-9

**Published:** 2019-07-17

**Authors:** Zi-Lei Xia, Chao Zheng, Ren-Qi Xu, Shu-Li You

**Affiliations:** 1 State Key Laboratory of Organometallic Chemistry, Center for Excellence in Molecular Synthesis, Shanghai Institute of Organic Chemistry, University of Chinese Academy of Sciences, Chinese Academy of Sciences, 345 Lingling Lu, 200032 Shanghai, China; 2 Collaborative Innovation Center of Chemical Science and Engineering, 300072 Tianjin, China

**Keywords:** Asymmetric catalysis, Synthetic chemistry methodology, Organocatalysis

## Abstract

Asymmetric dearomatization reactions have recently emerged as a powerful tool for the rapid build-up of the molecular complexity. Chiral three-dimensional polycyclic molecules bearing contiguous stereogenic centers can be synthesized from readily available planar aromatic feedstocks. Here we report that an intermolecular asymmetric dearomatization reaction of *α*-naphthols bearing a tethered nucleophile at the C4 position of the naphthol ring is achieved by a chiral phosphoric acid. The reaction proceeds via a highly chemo- and regioselective aminative dearomatization/Michael addition sequence, affording a wide array of functionalized cyclic ketones in good yields (up to 93%) with excellent enantioselectivity (up to >99% ee). The catalyst loading can be reduced to 0.1 mol%. Preliminary mechanistic investigations identify that the enantioselectivity is established in the dearomatization step, while the Michael addition is the rate-limiting step. A working model accounting for the origin of the stereochemistry is proposed based on DFT calculations.

## Abstract (web summary)

Catalytic asymmetric dearomatization (CADA) reactions readily convert common aromatic compounds to diverse 3D chiral, polycyclic molecules. Here, the authors report an intermolecular asymmetric dearomatization of nucleophile-tethered α-naphthols affording functionalized cyclic ketones with high enantioselectivity.

## Introduction

The demands for effective assembly of diverse molecular scaffolds are continuously growing along with the development of organic chemistry. Among various kinds of strategies that aim to rapidly increase molecular complexity, catalytic asymmetric dearomatization (CADA) reactions^1–12^ have recently received considerable attention of the synthetic community. They are capable of significantly enriching the chemical space by converting readily available planar aromatic compounds to diverse three-dimensional molecules bearing spiro or fused polycyclic skeletons with multiple stereogenic centers including quaternary ones.

**Fig. 1:**
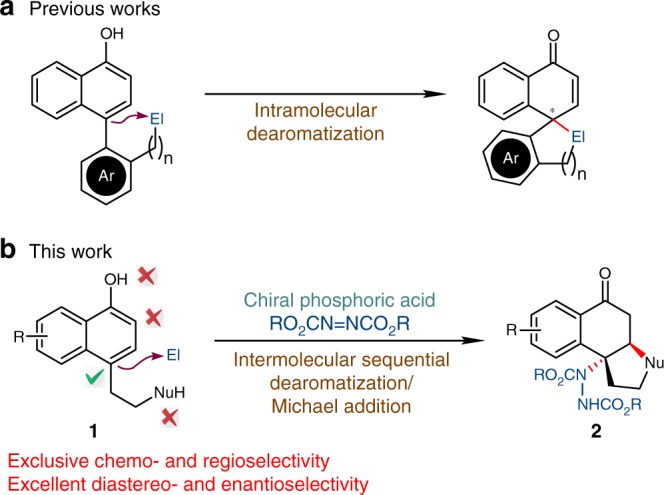
Catalytic Asymmetric Dearomatization of *α*-Naphthols at the C4 Position. **a** Intramolecular dearomatization. **b** Intermolecular sequential dearomatization/Michael addition. Ar: aromatic ring, El: electrophile, Nu: nucleophile, R: substituent

Naphthols serve as important aromatic feedstocks in organic chemistry. However, the asymmetric dearomatization reactions of naphthols are relatively less explored compared with those of other electron-rich arenes like indoles^13–18^. With the oxidation system consisting of a chiral hypervalent iodine reagent and *m*-CPBA as the terminal oxidant^19–29^, or transition-metal catalysts that are capable of promoting single electron oxidation^30,31^, *α*- or *β*-naphthols could react with intra- or intermolecular nucleophiles to yield chiral functionalized cyclic ketones. Notably, the asymmetric dearomatization reactions under non-oxidative conditions are rather limited within *β*-naphthols^32–52^, and the corresponding reactions of *α*-naphthols are relatively rare. In 2015, Luan and coworkers reported an elegant Pd-catalyzed dynamic kinetic asymmetric transformation of 4-(2-bromoaryl)-*α*-naphthols with alkynes for the synthesis of chiral spirocyclic enones^53^. Sporadic examples of non-oxidative asymmetric dearomatization of *α*-naphthols via organocatalytic chlorination^37^, annulation^54^, Ir-catalyzed allylic alkylation^38^, or Pd-catalyzed cross coupling^55–57^ were disclosed. However, the dearomatization reactions in these examples generally relied on the pre-installation of an intramolecular electrophile (Fig. 1a). Notably, during the preparation of this manuscript, Shao and coworkers reported an intermolecular asymmetric allylic dearomatization reaction of *α*-naphthols at the C2 position by a chiral phosphoric acid^58^. However, highly efficient asymmetric dearomatization reactions of *α*-naphthols with intermolecular electrophiles still remain underdeveloped.

In 2017, we reported a Pd-catalyzed arylative dearomatization/*aza*-Michael addition sequence between aryl bromide and *α*-naphthols bearing a tethered nucleophile at the C4 position of the naphthol ring^59^. The exclusive chemo-, and regioselectivity favoring the C4 position among the multiple potential nucleophilic sites encouraged us to further probe the possibility of developing a highly enantioselective dearomatization of *α*-naphthols/Michael addition sequence. However, all attempts to realize such an asymmetric reaction under Pd catalysis have been failed in our hands. In this regard, we speculated that azodicarboxylates^60–66^ might be employed as suitable electrophilic amination reagents which are compatible with the dearomatization of electron-rich arenes in the presence of a chiral phosphoric acid (CPA)^67–70^.

Here, we accomplished the efficient synthesis of a wide array of functionalized cyclic ketones bearing two consecutive stereogenic centers in excellent diastereo- and enantioselectivity (Fig. 1b). The products are readily involved in further transformations towards various enantioenriched polycyclic molecules.

## Results

### Reaction development

Our studies commenced with the attempt of the reaction of *α*-naphthol **1a** with 1.5 equivalents of diethyl azodicarboxylate (DEAD) (Table 1). In the presence of 10 mol% of chiral phosphoric acid **C1**, the target reaction proceeded smoothly in 1,2-dichloroethane (DCE) at 50 °C for 24 h, affording the dearomatized product **2a** in 85% yield with 75% ee as a single *trans* diastereoisomer (Table 1, entry 1). The structure and absolute configuration of **2a** (3*R*,4*R*) were established unambiguously by X-ray crystallographic analysis of an enantiopure sample. Interestingly, the Friedel–Crafts reaction at the C2 position was not observed. Systematic evaluation of a series of CPAs (**C2**–**C7**) was conducted (Table 1, entries 2–7). The results revealed that the catalysts have great influence on the reaction outcome. Notably, TRIP-CPA **C2** gave the optimal results (90% yield and 99% ee, Table 1, entry 2). Utilizing SiPh_3_-derived catalyst **C4** also led to comparable results with those of **C2** (90% yield and 98% ee, Table 1, entry 4). On the other hand, **2a** was obtained in the nearly racemic form (2% ee) when 4-NO_2_C_6_H_4_ derived **C6** was employed (Table 1, entry 6). Although excellent enantioselectivity has been obtained, other reaction parameters were further investigated. Typical solvents including EtOAc, THF or toluene also well facilitated the reaction (Table 1, entries 8-10). When the temperature was lowered to 40 °C, prolonged time (36 h) was necessary to provide **2a** in 88% yield (Table 1, entry 11). Similar results (85% yield and 99% ee) could be achieved in shorter time when elevating the reaction temperature to 60 °C (Table 1, entry 12). However, slightly decreased yield (77%) was observed when 1.1 equivalents of DEAD was used (Table 1, entry 13). The reactions proceeded well with reduced loading of **C2** (≤1 mol %, Table 1, entries 14-17). The remarkably high catalytic efficiency was exemplified by the fact that **2a** was afforded in 52% yield with 97% ee in the presence of 0.1 mol% of **C2** at 80 °C (Table 1, entry 17). However, only complex mixture was obtained in the absence of a CPA catalyst (Table 1, entry 18).

**Table 1:** Optimization of the reaction conditions^a^


Entry	C	Solvent	Temperature (°C)	Time (h)	Yield (%)^b^	ee (%)^c^
1	**C1**	DCE	50	24	85	75
2	**C2**	DCE	50	24	90	99
3	**C3**	DCE	50	24	87	63
4	**C4**	DCE	50	24	90	98
5	**C5**	DCE	50	24	85	57
6	**C6**	DCE	50	24	83	2
7	**C7**	DCE	50	24	83	59
8	**C2**	EtOAc	50	24	86	97
9	**C2**	THF	50	24	85	96
10	**C2**	toluene	50	24	87	98
11	**C2**	DCE	40	36	88	99
12	**C2**	DCE	60	12	85	99
13^d^	**C2**	DCE	50	36	77	99
14^e^	**C2**	DCE	50	96	81	99
15^e^	**C2**	DCE	80	30	81	99
16^f^	**C2**	DCE	80	24	69	98
17^g^	**C2**	DCE	80	24	52	97
18	**/**	DCE	50	12	complex	N.D.

*DEAD* diethyl azodicarboxylate, *Ts* tosyl, *Et* ethyl, *iPr* isopropyl, *Ph* phenyl, *DCE* 1,2-dichloroethane, *EtOAc* ethyl acetate, *THF* tetrahydrofuran, *N.D.* not determined

^a^Reaction conditions: **1a** (0.1 mmol), DEAD (0.15 mmol) and **C** (10 mol%) in solvent (2.0 mL) at specified temperature

^b^Isolated yield

^c^Determined by HPLC analysis on a chiral stationary phase

^d^DEAD (1.1 equiv) was used

^e^**C2** (1 mol%) was used

^f^**C2** (0.2 mol%) was used

^g^**C2** (0.1 mol%) was used

### Scope and limitation

**Fig. 2:**
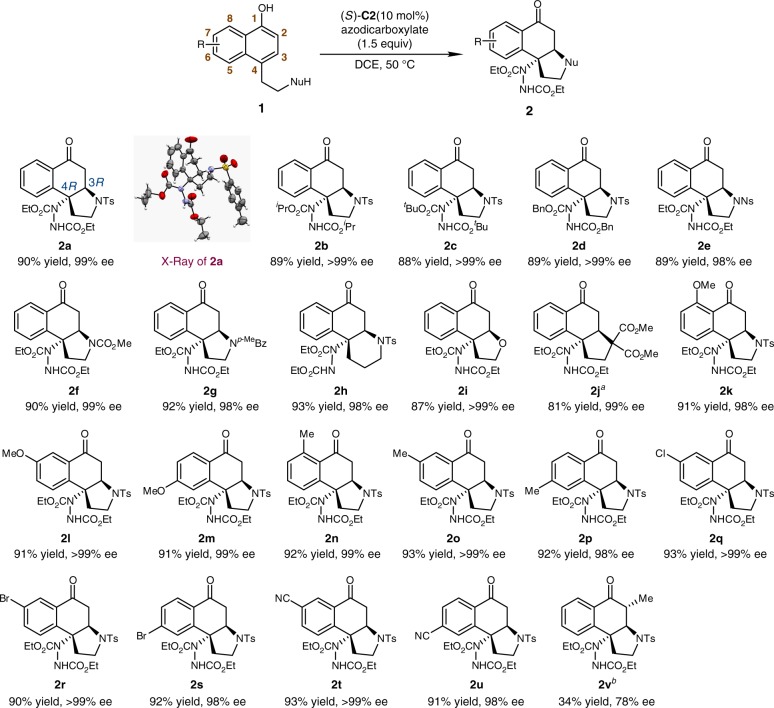
Substrate Scope of *α*-Naphthols. Reaction conditions: **1** (0.1 mmol), azodicarboxylate (0.15 mmol) and (*S*)-**C2** (10 mol%) in DCE (2 mL) at 50 °C. ^a^After work up, ^t^BuOK (2 equiv in THF) was added at rt. ^b^At 0.2 mmol scale. Bz, benzoyl

With the optimal conditions in hands (Table 1, entry 2), we next explored the substrate scope (Fig. 2). The desired reactions of **1a** with other azodicarboxylates all proceeded smoothly, leading to **2b**–**2d** in high yields (88–89%). The *N*-protecting group of *α*-naphthol substrates could be switched to Ns, CO_2_Me or *p*-MeC_6_H_4_CO_2_. The corresponding products (**2e**–**2g**) could be obtained in 89–92% yields. The prolonged tether at the C4 position of the naphthol ring was well accommodated, affording the piperidine-fused product **2h** in 93% yield. Besides nitrogen nucleophiles, the hydroxyl group could also work as the nucleophile. The tetrahydrofuran-fused product **2i** was accomplished smoothly in 87% yield. Notably, when a malonate diester nucleophile was employed, only the dearomatization process occurred under the standard conditions. The target product **2j** could be obtained (81% yield) after a subsequent Michael addition promoted by ^*t*^BuOK.

On the other hand, various substituted *α*-naphthols underwent the desired dearomatization reactions. Electron-donating groups such as MeO and Me could be tolerated at the C6 to C8 positions (**2k–2p**, 91–93% yields). The C6 and C7 positions could accommodate halogen atoms including Cl and Br (**2q–2s**, 90–93% yields). Besides, when a CN group was installed at the C6 or C7 position, the dearomatization reaction delivered the desired products in high yields (**2t**, 93% yield; **2u**, 91% yield). Gratifyingly, exceedingly high enantioselectivity was observed in all the above cases (**2a–2u**, 98 to >99% ee). However, when the substrate bearing a methyl group at the C2 position was subjected to the standard conditions, the reaction outcomes decreased apparently in terms of yield and enantioselectivity (**2v**, 34% yield and 78% ee). It should be noted that only complex mixtures were obtained when substrates bearing a substituent at the C5 position were utilized.

**Fig. 3:**
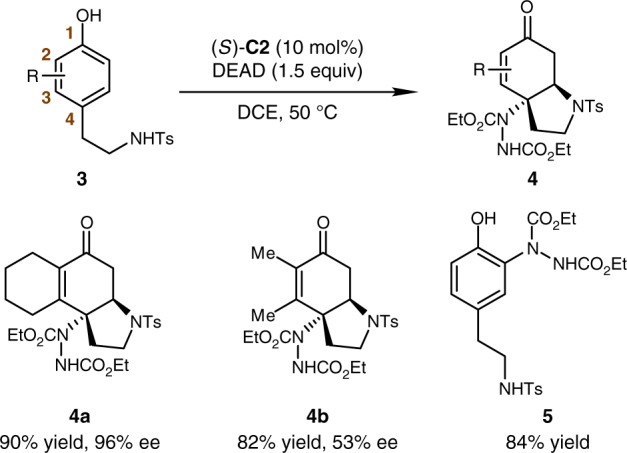
Substrate Scope of Phenols. Reaction conditions: **3** (0.1 mmol), DEAD (0.15 mmol) and (*S*)-**C2** (10 mol%) in DCE (2 mL) at 50 °C. Me, methyl

The dearomatization reaction of phenols is much more challenging compared with that of naphthols. In addition, by-products of Friedel–Crafts reactions are difficult to be avoided. To further expand the substrate scope of this aminative dearomatization/Michael addition sequence, several phenol analogs were tested (Fig. 3). Encouragingly, *α*-tetrahydronaphthol-derived substrate **3a** participated the title reaction smoothly, delivering the desired product **4a** in 90% yield with 96% ee, without the observation of Friedel–Crafts by-product. The reaction of sterically less demanding phenol substrate (2,3-Me_2_) also proceeded well. The corresponding product **4b** was obtained in good yield (82%) but with rather decreased enantioselectivity (53% ee). Notably, a mixture of Friedel–Crafts-type byproducts became dominating for the substrates bearing one substituent (Me, MeO, or Br) at the C2 or C3 position. Compound **5** was isolated in 84% yield for the phenol substrate bearing no substituent at the C2 or C3 position.

### Mechanistic studies

**Fig. 4:**
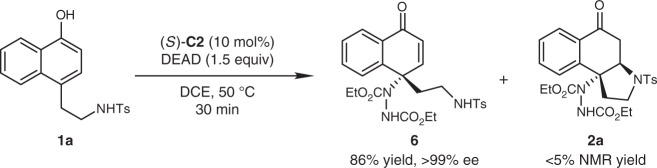
Isolation of the Intermediate. Reaction conditions: **1a** (0.1 mmol), DEAD (0.15 mmol) and (*S*)-**C2** (10 mol%) in DCE (2 mL) at 50 °C for 30 min

To shed light on the reaction mechanism, we first attempted to isolate the intermediate of this sequential reaction (Fig. 4). When the reaction of **1a** was quenched within 30 min, only trace amount of **2a** was observed (<5% NMR yield), while conjugated enone **6** was isolated in 86% yield with >99% ee. Compound **6** was found stable enough to be stored at −20 °C for weeks without apparent transformation into **2a** or decomposition. Taking into consideration that much longer time (24 h) was required for most substrates to fully transfer into the final products, and the identical enantiopurity of **6** and that of **2a** obtained under the standard conditions, we proposed that the stereochemistry of the whole reaction is established irreversibly in the aminative dearomatization step, whereas the following Michael addition is the rate-limiting step.

**Fig. 5:**
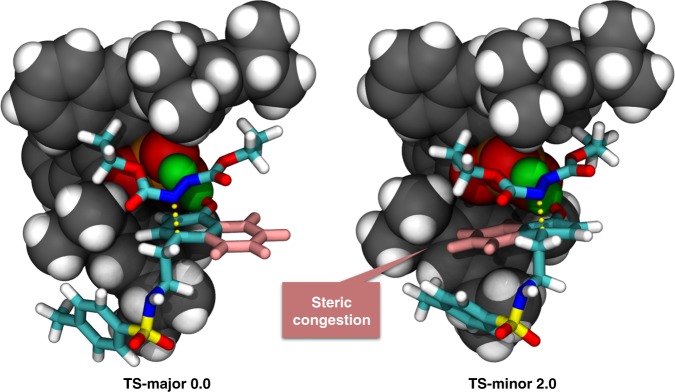
Transition states. Optimized structures and relative Gibbs free energies (in kcal/mol) of the transition states of the aminative dearomatization step (ωB97XD/def2-TZVPP//B97D/6-31G**). The catalysts are presented with the van der Waals model. The substrates are presented with the stick model. The forming C–C bonds are shown in yellow dash lines. The transferring protons are shown in green spheres. The intact benzene rings of *α*-naphthols are shown in pink

To explore the origin of the exceedingly high enantioselectivity, DFT calculations were then performed for the aminative dearomatization step (Fig. 5). The relative Gibbs free energy of **TS-major**, the transition state leading to the major product, was 2.0 kcal/mol lower than that of **TS-minor**, which was in qualitative agreement with the experimental results. The two prochiral faces at the C4 position of *α*-naphthol were well discriminated by the chiral phosphoric acid. In **TS-major**, the *Si*-face of the C4 position was attacked, which allowed the intact benzene ring of *α*-naphthol in distal to the chiral pocket. Whereas in **TS-minor**, strong steric congestion was developed due to the *Re*-face attack and the concomitant approximation of the intact benzene ring to one TRIP group of **C2**. These computational results corresponded well with the fact that substituents were tolerated at the C6 to C8 positions of *α*-naphthol (**2k–2u**), but not at the C2 position (**2v**). In addition, a fused saturated skeleton that is sterically roughly comparable with a benzene ring should be beneficial for the high enantioselectivity (**4a**), but sterically less demanding 2,3-Me_2_ groups were not that effective (**4b**).

### Product transformations

**Fig. 6:**
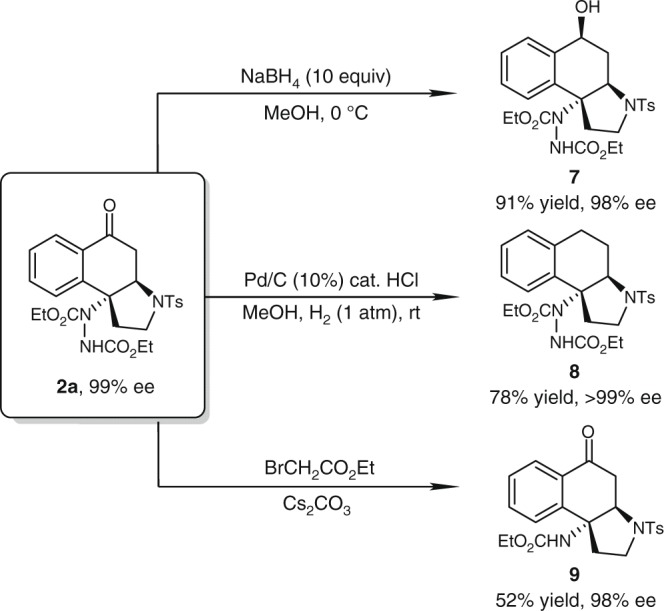
Transformations of **2a**. Reaction conditions for synthesis of **7**: **2a** (0.2 mmol), NaBH_4_ (2.0 mmol) in MeOH (4 mL) at 0 °C. Reaction conditions for synthesis of **8**: **2a** (0.2 mmol), Pd/C (10%, 50 mg), HCl (conc. 20 μL) in MeOH (4 mL) under H_2_ (1 atm) at room temperature. Reaction conditions for synthesis of **9**: **2a** (0.2 mmol), Cs_2_CO_3_ (0.26 mmol), BrCH_2_CO_2_Et (0.22 mmol) in CH_3_CN (4 mL) at 50 °C

To highlight the practicality and synthetic utility of this method, a gram-scale reaction of **1a** with DEAD was performed under the standard reaction conditions. **2a** was obtained in 87% yield (1.35 g) with 99% ee. In addition, several functional group transformations of **2a** were demonstrated (Fig. 6). Tertiary alcohol **7** was delivered in 91% yield when treated with NaBH_4_. The ketone group could also be hydrogenated to methylene group under the catalysis of palladium on charcoal, leading to **8** in 78% yield. In addition, the N–N bond could be cleaved by treating **2a** with ethyl bromoacetate and cesium carbonate. The corresponding product **9** was afforded in 52% yield. Notably, no erosion of enantiomeric purity was observed in all these transformations.

## Discussion

In conclusion, we have achieved a chiral phosphoric acid-catalyzed dearomatization reaction of *α*-naphthols bearing a tethered nucleophile at the C4 position of the naphthol ring. Preliminary mechanistic investigations confirmed that the reaction proceeded via two steps, a stereochemistry-determining aminative dearomatization followed by a rate-limiting Michael addition. The reaction occurred under mild conditions, affording a wide array of polycyclic ketones in good yields with excellent enantioselectivity. Besides, the reaction features high catalytic efficiency and diverse transformations of the products. A working model accounting for the origin of the stereochemical induction was proposed based on DFT calculations.

## Methods

### Representative procedure

To a flask containing a mixture of **1a** (0.1 mmol) and (*S*)-**C2** (7.5 mg, 0.01 mmol) under argon was added a solution of the corresponding azodicarboxylate (0.15 mmol) in anhydrous 1,2-dichloroethane (2 mL). The reaction was stirred at 50 °C until TLC showed complete consumption of the starting material. The reaction mixture was cooled to room temperature, quenched with NaHCO_3_ (aq., 10 mL) and extracted with CH_2_Cl_2_ (3 × 15 mL). The combined organic layer was washed with brine, separated, dried over Na_2_SO_4_ and filtrated. After the solvent was removed under reduced pressure, the residue was purified by silica gel column chromatography (ethyl acetate/petroleum ether = 1/6 to 1/2) to afford **2a**.

## Supplementary information

Supplementary Information

Description of Additional Supplementary Files

Supplementary Data 1

## Data Availability

The X-ray crystallographic coordinates for product **2a** have been deposited at the Cambridge Crystallographic Data Centre (CCDC) with the accession code 1888563. These data can be obtained free of charge from The Cambridge Crystallographic Data Centre via www.ccdc.cam.ac.uk/data_request/cif. The authors declare that all other data supporting the findings of this work, including experimental procedures, compound characterization data, and computational details, are available within the article and its Supplementary Information files.

## References

[1] Pape AR, Kaliappan KP, Kündig EP (2000). Transition-metal-mediated dearomatization reactions. Chem. Rev..

[2] Roche SP, Porco JA (2011). Dearomatization strategies in the synthesis of complex natural products. Angew. Chem. Int. Ed..

[3] Zhuo C-X, Zhang W, You S-L (2012). Catalytic asymmetric dearomatization reactions. Angew. Chem. Int. Ed..

[4] Zhuo C-X, Zheng C, You S-L (2014). Transition-metal-catalyzed asymmetric allylic dearomatization reactions. Acc. Chem. Res..

[5] Ding Q, Zhou X, Fan R (2014). Recent advances in dearomatization of heteroaromatic compounds. Org. Biomol. Chem..

[6] Zheng C, You S-L (2016). Catalytic asymmetric dearomatization by transition-metal catalysis: a method for transformations of aromatic compounds. Chem.

[7] Manoni E, De Nisi A, Bandini M (2016). New opportunities in the stereoselective dearomatization of indoles. Pure Appl. Chem..

[8] Chen J-B, Jia Y-X (2017). Recent progress in transition-metal-catalyzed enantioselective indole functionalizations. Org. Biomol. Chem..

[9] Wu W-T, Zhang L, You S-L (2017). Recent progress on gold-catalyzed dearomatization reactions. Acta Chim. Sin..

[10] Wertjes WC, Southgate EH, Sarlah D (2018). Recent advances in chemical dearomatization of nonactivated arenes. Chem. Soc. Rev..

[11] An J, Bandini M (2018). Gold-catalyzed dearomatization reactions. Chimia.

[12] You, S.-L. Ed. Asymmetric Dearomatization Reactions (Wiley-VCH, Weinheim, Germany, 2016).

[13] Quideau, S., Pouységu, L. & Deffieux, D. Oxidative dearomatization of phenols: why, how and what for? *Synlett* 467-495 (2008).

[14] Pouységu L, Deffieux D, Quideau S (2010). Hypervalent iodine-mediated phenol dearomatization in natural product synthesis. Tetrahedron.

[15] Pouységu L (2010). Hypervalent iodine-mediated oxygenative phenol dearomatization reactions. Tetrahedron.

[16] Wu W-T, Zhang L, You S-L (2016). Catalytic asymmetric dearomatization (CADA) reactions of phenol and aniline derivatives. Chem. Soc. Rev..

[17] Sun W, Li G, Hong L, Wang R (2016). Asymmetric dearomatization of phenols. Org. Biomol. Chem..

[18] Flores A, Cots E, Bergès J, Muñiz K (2019). Enantioselective iodine(I/III) catalysis in organic synthesis. Adv. Synth. Catal..

[19] Dohi T (2008). A chiral hypervalent iodine(III) reagent for enantioselective dearomatization of phenols. Angew. Chem. Int. Ed..

[20] Quideau S (2009). Asymmetric hydroxylative phenol dearomatization through in situ generation of iodanes from chiral iodoarenes and *m*-CPBA. Angew. Chem. Int. Ed..

[21] Uyanik M, Yasui T, Ishihara K (2010). Enantioselective Kita oxidative spirolactonization catalyzed by in situ generated chiral hypervalent iodine(III) species. Angew. Chem. Int. Ed..

[22] Dohi T (2013). Asymmetric dearomatizing spirolactonization of naphthols catalyzed by spirobiindane-based chiral hypervalent iodine species. J. Am. Chem. Soc..

[23] Uyanik M, Yasui T, Ishihara K (2013). Hydrogen bonding and alcohol effects in asymmetric hypervalent iodine catalysis: enantioselective oxidative dearomatization of phenols. Angew. Chem. Int. Ed..

[24] Volp KA, Harned AM (2013). Chiral aryl iodide catalysts for the enantioselective synthesis of para-quinols. Chem. Commun..

[25] Bosset C (2014). Asymmetric hydroxylative phenol dearomatization promoted by chiral binaphthylic and biphenylic iodanes. Angew. Chem. Int. Ed..

[26] Murray SJ, Ibrahim H (2015). Asymmetric Kita spirolactonisation catalysed by anti-dimethanoanthracene-based iodoarenes. Chem. Commun..

[27] Zhang D-Y, Xu L, Wu H, Gong L-Z (2015). Chiral iodine-catalyzed dearomatizative spirocyclization for the enantioselective construction of an all-carbon stereogenic center. Chem. Eur. J..

[28] Uyanik M, Yasui T, Ishihara K (2017). Chiral hypervalent organoiodine-catalyzed enantioselective oxidative spirolactonization of naphthol derivatives. J. Org. Chem..

[29] Hempel C, Maichle-Mössmer C, Pericàs MA, Nachtsheim BJ (2018). Modular synthesis of triazole-based chiral iodoarenes for enantioselective spirocyclizations. Adv. Synth. Catal..

[30] Oguma T, Katsuki T (2012). Iron-catalyzed dioxygen-driven C–C bond formation: oxidative dearomatization of 2-naphthols with construction of a chiral quaternary stereocenter. J. Am. Chem. Soc..

[31] Oguma T, Katsuki T (2014). Iron-catalysed asymmetric tandem spiro-cyclization using dioxygen in air as the hydrogen acceptor. Chem. Commun..

[32] Zhuo C-X, You S-L (2013). Palladium-catalyzed intermolecular asymmetric allylic dearomatization reaction of naphthol derivatives. Angew. Chem. Int. Ed..

[33] Zheng J, Wang S-B, Zheng C, You S-L (2015). Asymmetric dearomatization of naphthols via a Rh-catalyzed C(sp^2^)–H functionalization/annulation reaction. J. Am. Chem. Soc..

[34] Yang D (2015). Intermolecular enantioselective dearomatization reaction of *β*-naphthol using *meso*-aziridine: a bifunctional in situ generated magnesium catalyst. Angew. Chem. Int. Ed..

[35] Yang D (2015). Application of a C–C bond-forming conjugate addition reaction in asymmetric dearomatization of *β*-naphthols. Angew. Chem. Int. Ed..

[36] Wang S-G (2015). Asymmetric dearomatization of *β*-naphthols through a bifunctional-thiourea-catalyzed Michael reaction. Angew. Chem. Int. Ed..

[37] Yin Q (2015). Organocatalytic asymmetric chlorinative dearomatization of naphthols. Chem. Sci..

[38] Cheng Q, Wang Y, You S-L (2016). Chemo-, diastereo-, and enantioselective iridium-catalyzed allylic intramolecular dearomatization reaction of naphthol derivatives. Angew. Chem. Int. Ed..

[39] Wang L (2016). Mg^II^-mediated catalytic asymmetric dearomatization (CADA) reaction of *β*-naphthols with dialkyl acetylenedicarboxylates. Chem. Eur. J..

[40] Zhu G (2016). Chiral phosphoric acid catalyzed asymmetric oxidative dearomatization of naphthols with quinones. Org. Lett..

[41] Tu H-F, Zheng C, Xu R-Q, Liu X-J, You S-L (2017). Iridium-catalyzed intermolecular asymmetric dearomatization of *β*-naphthols with allyl alcohols or allyl ethers. Angew. Chem. Int. Ed..

[42] Shen D, Chen Q, Yan P, Zeng X, Zhong G (2017). Enantioselective dearomatization of naphthol derivatives with allylic alcohols by cooperative iridium and Brønsted acid catalysis. Angew. Chem. Int. Ed..

[43] Zhang Y (2017). Catalytic asymmetric hydroxylative dearomatization of 2-naphthols: synthesis of lacinilene derivatives. Chem. Sci..

[44] Ge S (2017). Chiral N,N’-dioxide/Sc(OTf)_3_ complex-catalyzed asymmetric dearomatization of *β*-naphthols. Chem. Commun..

[45] Li X-Q (2017). Asymmetric arylative dearomatization of *β*-naphthols catalyzed by a chiral phosphoric acid. Chem. Eur. J..

[46] An J (2017). Gold-catalyzed dearomatization of 2-naphthols with alkynes. Chem. Eur. J..

[47] Liu X (2018). Construction of vicinal all-carbon quaternary stereocenters enabled by a catalytic asymmetric dearomatization reaction of *β*-naphthols with 3-bromooxindoles. ACS Catal..

[48] Wang P (2018). Asymmetric dearomative halogenation of *β*-naphthols: the axial chirality transfer reaction. Adv. Synth. Catal..

[49] Wang Y-F (2018). Asymmetric brominative dearomatization of naphthols catalyzed by chiral copper complexes. Adv. Synth. Catal..

[50] Wang L (2018). Diversiform reactivity of naphthols in asymmetric dearomatization or O-alkylation reactions with aziridines. Adv. Synth. Catal..

[51] Han L, Wang H, Luan X (2018). Pd(II)-Catalyzed [3+2] spiroannulation of *α*-aryl-*β*-naphthols with alkynes via a C–H activation/dearomatization approach. Org. Chem. Front.

[52] Wang J-J, Yang H, Gou B-B, Zhou L, Chen J (2018). Enantioselective organocatalytic sulfenylation of *β*-naphthols. J. Org. Chem..

[53] Yang L (2015). Palladium-catalyzed dynamic kinetic asymmetric transformation of racemic biaryls: axial-to-central chirality transfer. J. Am. Chem. Soc..

[54] Zhu G (2018). Catalytic kinetic resolution of spiro-epoxyoxindoles with 1-naphthols: switchable asymmetric tandem dearomatization/oxa-Michael reaction and Friedel–Crafts alkylation of 1-naphthols at the C4 position. ACS Catal..

[55] Rousseaux S, García-Fortanet J, Del Aguila Sanchez MA, Buchwald SL (2011). Palladium(0)-catalyzed arylative dearomatization of phenols. J. Am. Chem. Soc..

[56] Bai L (2016). Palladium(0)-catalyzed intermolecular carbocyclization of (1,n)-diynes and bromophenols: an efficient route to tricyclic scaffolds. Angew. Chem. Int. Ed..

[57] Zhao G, Xu G, Qian C, Tang W (2017). Efficient enantioselective syntheses of (+)-dalesconol A and B. J. Am. Chem. Soc..

[58] Yang B (2019). Organocatalyzed intermolecular asymmetric allylic dearomatization of both *α*- and *β*-naphthols. Org. Lett..

[59] Xu R-Q, Gu Q, You S-L (2017). Construction of the benzomesembrine skeleton: palladium(0)-catalyzed intermolecular arylative dearomatization of *α*-naphthols and subsequent aza-Michael reaction. Angew. Chem. Int. Ed..

[60] List B (2002). Direct catalytic asymmetric *α*-amination of aldehydes. J. Am. Chem. Soc..

[61] Brandes S, Bella M, Kjærsgaard A, Jørgensen KA (2006). Chirally aminated 2-naphthols–organocatalytic synthesis of non-biaryl atropisomers by asymmetric Friedel–Crafts amination. Angew. Chem. Int. Ed..

[62] Matsubara R, Kobayashi S (2006). Catalytic asymmetric amination of enecarbamates. Angew. Chem. Int. Ed..

[63] Zhang Z, Antilla JC (2012). Enantioselective construction of pyrroloindolines catalyzed by chiral phosphoric acids: total synthesis of (−)-debromoflustramine B. Angew. Chem. Int. Ed..

[64] Wang S-G, Yin Q, Zhuo C-X, You S-L (2015). Asymmetric dearomatization of *β*-naphthols through an amination reaction catalyzed by a chiral phosphoric acid. Angew. Chem. Int. Ed..

[65] Nan J (2015). Direct asymmetric dearomatization of 2-naphthols by scandium-catalyzed electrophilic amination. Angew. Chem. Int. Ed..

[66] Lian X, Lin L, Wang G, Liu X, Feng X (2015). Chiral N,N’-dioxide-scandium(III)-catalyzed asymmetric dearomatization of 2-naphthols through an amination reaction. Chem. Eur. J..

[67] Akiyama T (2007). Stronger Brønsted Acids. Chem. Rev..

[68] Terada, M. Binaphthol-derived phosphoric acid as a versatile catalyst for enantioselective carbon–carbon bond forming reactions. *Chem. Commun*. 4097-4112 (2008). 10.1039/b807577h 18802501

[69] Yu J, Shi F, Gong L-Z (2011). Brønsted-acid-catalyzed asymmetric multicomponent reactions for the facile synthesis of highly enantioenriched structurally diverse nitrogenous heterocycles. Acc. Chem. Res..

[70] Parmar D, Sugiono E, Raja S, Rueping M (2014). Complete field guide to asymmetric BINOL-phosphate derived Brønsted acid and metal catalysis: history and classification by mode of activation; Brønsted acidity, hydrogen bonding, ion pairing, and metal phosphates. Chem. Rev..

