# Supplementary material for: Chiral phosphoric acid catalyzed aminative dearomatization of α-naphthols/Michael addition sequence
Source: Nat Commun. 2019 Jul 17;10:3150. doi: 10.1038/s41467-019-11109-9 (PMC6637135; doi:10.1038/s41467-019-11109-9)
Supplement: Supplementary file 1 — Supplementary Information [file 41467_2019_11109_MOESM1_ESM.pdf]

Supplementary Information

Chiral Phosphoric Acid Catalyzed Aminative Dearomatization of α-Naphthols/Michael Addition Sequence

Zi-Lei Xia, *et al.*

General methods

Unless otherwise stated, all reactions were carried out in flame-dried glassware under a dry argon atmosphere. All solvents were purified and dried according to standard methods prior to use. ^{1}H and ^{13}C NMR spectra were recorded on a Varian instrument (300 MHz and 75 MHz, 400 MHz and 100 MHz respectively) and internally referenced to tetramethylsilane signal or residual protio solvent signals. Data for ^{1}H NMR are recorded as follows: chemical shift (δ, ppm), multiplicity (s = singlet, d = doublet, t = triplet, m = multiplet or unresolved, br = broad singlet, coupling constant(s) in Hz, integration). Data for ^{13}C NMR are reported in terms of chemical shift (δ, ppm).

Experimental section

General procedure for the preparation of substrates **1** and **3**

The substrates **1a**-**1j**, **1v** and substrate of **5** are known compounds. The synthesis of **1k**-**1u** and **3a**-**3b** was accomplished following the reported procedures.[1-11]

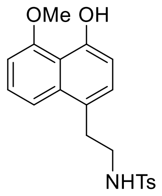

1k, gray solid, m.p. = 43-45 °C. Analytical data: ^{1}H NMR (400 MHz, $CDCl_3$) δ 9.39 (s, 1H), 7.62 (d, *J* = 7.2 Hz, 2H), 7.38 (d, *J* = 8.0 Hz, 1H), 7.32-7.22 (m, 1H), 7.17 (d, *J* = 7.2 Hz, 2H), 7.06 (d, *J* = 7.2 Hz, 1H), 6.78-6.65 (m, 2H), 4.83 (s, 1H), 4.01 (s, 3H), 3.26-3.17 (m, 2H), 3.10-3.01 (m, 2H), 2.35 (s, 3H). ^{13}C NMR (100 MHz, $CDCl_3$) δ 156.7, 153.9, 143.2, 136.8, 134.5, 129.6, 129.0, 127.0, 126.1, 123.9, 117.3, 115.3, 109.9, 104.1, 56.2, 43.5, 33.3, 21.5. IR (thin film): ν_{max} (cm^{-1}) = 2962, 2853, 1575, 1259, 1089, 1016, 795, 757. HRMS (ESI) calcd for $C_{20}H_{25}N_2O_4S$ $[M+NH_4]^+$: 389.1530, Found: 389.1529.

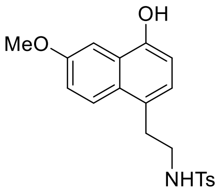

1l, gray solid, m.p. = 136-138 °C. Analytical data: ^{1}H NMR (400 MHz, d_6-Acetone) δ 8.82 (s, 1H), 7.78 (d, *J* = 8.8 Hz, 1H), 7.69 (d, *J* = 7.2 Hz, 2H), 7.58 (s, 1H), 7.30 (d, *J* = 7.2 Hz, 2H), 7.11 (d, *J* =

8.4 Hz, 1H), 6.97 (d, *J* = 7.2 Hz, 1H), 6.78 (d, *J* = 7.2 Hz, 1H), 6.50 (br, 1H), 3.88 (s, 3H), 3.22-3.02 (m, 4H), 2.34 (s, 3H). ^{13}C NMR (100 MHz, d_6-Acetone) δ 156.8, 151.3, 142.9, 138.0, 129.5, 128.2, 126.9, 126.4, 125.5, 125.0, 124.7, 118.5, 108.2, 101.1, 54.6, 44.1, 32.9, 20.5. IR (thin film): ν_{max} (cm^{-1}) = 2962, 2853, 1575, 1259, 1089, 1016, 795, 757. HRMS (ESI) calcd for $C_{20}H_{25}N_2O_4S$ $[M+NH_4]^+$: 389.1530, Found: 389.1529.

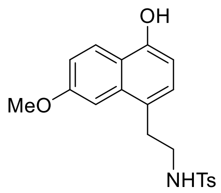

1m, gray solid, m.p. = 155-157 °C. Analytical data: ^{1}H NMR (400 MHz, d_6-Acetone) δ 8.91 (s, 1H), 8.20 (d, *J* = 8.8 Hz, 1H), 7.73 (d, *J* = 8.0 Hz, 2H), 7.33-7.26 (m, 3H), 7.16-7.05 (m, 2H), 6.70 (d, *J* = 7.8 Hz, 1H), 6.61 (t, *J* = 5.2 Hz, 1H), 3.91 (s, 3H), 3.27-3.12 (m, 4H), 2.35 (s, 3H). ^{13}C NMR (100 MHz, d_6-Acetone) δ 158.3, 152.4, 142.9, 138.2, 134.5, 129.5, 128.0, 126.8, 124.5, 124.2, 120.4, 116.5, 105.7, 102.2, 54.8, 43.8, 33.4, 20.6. IR (thin film): ν_{max} (cm^{-1}) = 2962, 2853, 1575, 1259, 1089, 1016, 795, 757. HRMS (ESI) calcd for $C_{20}H_{25}N_2O_4S$ $[M+NH_4]^+$: 389.1530, Found: 389.1529.

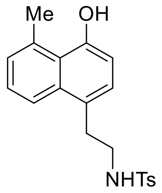

1n, gray solid, m.p. = 160-162 °C. Analytical data: ^{1}H NMR (400 MHz, d_6-Acetone) δ 8.76 (s, 1H), 7.75-7.63 (m, 3H), 7.35-7.23 (m, 3H), 7.13 (d, *J* = 6.4 Hz, 1H), 7.07 (d, *J* = 7.6 Hz, 1H), 6.77 (d, *J* = 7.6 Hz, 1H), 6.51 (s, 1H), 3.19-3.05 (m, 4H), 2.90 (s, 3H), 2.35 (s, 3H). ^{13}C NMR (100 MHz, d_6-Acetone) δ 154.8, 142.8, 138.1, 136.0, 134.3, 129.5, 127.4, 127.2, 126.9, 125.8, 125.5, 124.6,

121.5, 109.1, 43.9, 33.5, 24.7, 20.5. IR (thin film): ν_{max} (cm^{-1}) = 3450, 3275, 2905, 1590, 1417, 1320, 1271, 1154, 1030, 810, 760, 674. HRMS (ESI) calcd for $C_{20}H_{25}N_2O_3S$ $[M+NH_4]^+$: 373.1580, Found: 373.1578.

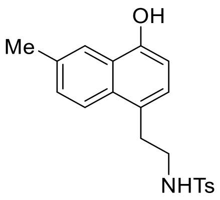

1o, gray solid, m.p. = 132-134 °C. Analytical data: ^{1}H NMR (400 MHz, d_6-Acetone) δ 8.85 (s, 1H), 8.03 (s, 1H), 7.80-7.60 (m, 3H), 7.35-7.20 (m, 3H), 7.03 (d, *J* = 6.4 Hz, 1H), 6.76 (d, *J* = 6.4 Hz, 1H), 6.52 (s, 1H), 3.14 (br s, 4H), 2.46 (s, 3H), 2.35 (s, 3H). ^{13}C NMR (100 MHz, d_6-Aetone) δ 151.8, 142.8, 138.1, 133.7, 131.1, 129.5, 128.3, 126.9, 126.2, 125.5, 125.2, 123.2, 121.8, 107.6, 44.1, 32.8, 21.0, 20.9, 20.5. IR (thin film): ν_{max} (cm^{-1}) = 3455, 3277, 2905, 1587, 1416, 1319, 1271, 1193, 1153, 1048, 909, 812, 755, 673. HRMS (ESI) calcd for $C_{20}H_{25}N_2O_3S$ $[M+NH_4]^+$: 373.1580, Found: 373.1578.

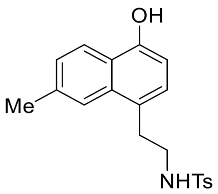

1p, gray solid, m.p. = 168-170 °C. Analytical data: ^{1}H NMR (400 MHz, d_6-Acetone) δ 8.84 (s, 1H), 8.15 (d, *J* = 8.4 Hz, 1H), 7.74-7.68 (m, 3H), 7.34 (d, *J* = 8.0 Hz, 2H), 7.29 (d, *J* = 8.4 Hz, 1H), 7.08 (d, *J* = 7.6 Hz, 1H), 6.72 (d, *J* = 7.6 Hz, 1H), 6.55 (s, 1H), 3.22-3.10 (m, 4H), 2.48 (s, 3H), 2.39 (s, 3H). ^{13}C NMR (100 MHz, d_6-Aetone) δ 152.2, 142.8, 138.2, 135.7, 133.1, 129.5, 127.3, 126.9, 126.3, 124.7, 123.5, 122.7, 122.4, 106.8, 44.0, 32.9, 21.2, 20.5. IR (thin film): ν_{max} (cm^{-1}) = 3336, 3224,

2920, 1598, 1426, 1315, 1222, 1146, 1051, 914, 826, 812, 664. HRMS (ESI) calcd for $C_{20}H_{25}N_2O_3S$ $[M+NH_4]^+$: 373.1580, Found: 373.1578.

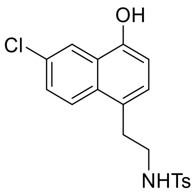

1q, gray solid, m.p. = 149-151 °C. Analytical data: ^1H NMR (400 MHz, d_6-Acetone) δ 9.17 (s, 1H), 8.21 (d, *J* = 2.4 Hz, 1H), 7.92 (d, *J* = 9.2 Hz, 1H), 7.68 (d, *J* = 8.4 Hz, 2H), 7.47 (dd, *J* = 8.8, 2.4 Hz, 1H), 7.32 (d, *J* = 8.0 Hz, 2H), 7.18 (d, *J* = 7.6 Hz, 1H), 6.88 (d, *J* = 8.0 Hz, 1H), 6.54 (br s, 1H), 3.25-3.11 (m, 4H), 2.39 (s, 3H). ^{13}C NMR (100 MHz, d_6-Acetone) δ 151.4, 142.8, 138.0, 131.1, 129.9, 129.5, 127.9, 126.8, 126.6, 126.1, 125.7, 125.7, 121.7, 108.9, 43.9, 32.6, 20.5. IR (thin film): ν_{max} (cm^{-1}) = 3500, 3284, 2834, 1586, 1416, 1307, 1283, 1193, 1158, 1091, 1050, 933, 813, 755, 667. HRMS (ESI) calcd for $C_{19}H_{22}ClN_2O_3S$ $[M+NH_4]^+$: 393.1034, Found: 393.1035.

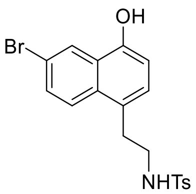

1r, gray solid, m.p. = 161-163 °C. Analytical data: ^1H NMR (400 MHz, d_6-Acetone) δ 9.18 (s, 1H), 8.39 (d, *J* = 2.0 Hz, 1H), 7.84 (d, *J* = 9.2 Hz, 1H), 7.67 (d, *J* = 8.0 Hz, 2H), 7.57 (dd, *J* = 9.2, 2.0 Hz, 1H), 7.31 (d, *J* = 8.0 Hz, 2H), 7.19 (d, *J* = 7.6 Hz, 1H), 6.87 (d, *J* = 7.6 Hz, 1H), 6.53 (s, 1H), 3.23-3.11 (m, 4H), 2.38 (s, 3H). ^{13}C NMR (100 MHz, d_6-Acetone) δ 151.3, 142.8, 138.0, 131.3, 129.4, 129.2, 128.0, 126.8, 126.5, 125.8, 125.7, 125.0, 118.0, 108.8, 43.9, 32.6, 20.5. IR (thin film):

ν_{max} (cm^{-1}) = 3482, 3281, 2924, 1584, 1406, 1305, 1283, 1148, 1085, 1050, 933, 812, 667. HRMS (ESI) calcd for $C_{19}H_{22}BrN_2O_3S$ [M+NH$_4$]$^+$: 437.0529, Found: 437.0530.

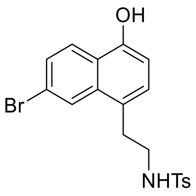

1s, gray solid, m.p. = 149-151 °C. Analytical data: ^{1}H NMR (400 MHz, d$_6$-Acetone) δ 9.17 (s, 1H), 8.19 (d, *J* = 9.2 Hz, 1H), 8.12 (d, *J* = 1.6 Hz, 1H), 7.72 (d, *J* = 8.0 Hz, 2H), 7.56 (dd, *J* = 8.8, 1.6 Hz, 1H), 7.33 (d, *J* = 8.0 Hz, 2H), 7.21 (d, *J* = 8.0 Hz, 1H), 6.87 (d, *J* = 7.6 Hz, 1H), 6.58 (s, 1H), 3.27-3.09 (m, 4H), 2.38 (s, 3H). ^{13}C NMR (100 MHz, d$_6$-Acetone) δ 152.4, 142.9, 138.0, 134.1, 129.5, 128.8, 127.4, 126.9, 125.6, 125.1, 124.8, 123.8, 120.5, 108.3, 43.9, 32.6, 20.5. IR (thin film): ν_{max} (cm^{-1}) = 3321, 3232, 2923, 1584, 1426, 1315, 1268, 1144, 1090, 1045, 825, 810, 662. HRMS (ESI) calcd for $C_{19}H_{22}BrN_2O_3S$ [M+NH$_4$]$^+$: 437.0529, Found: 437.0530.

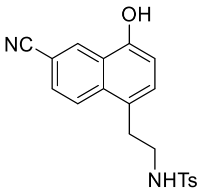

1t, gray solid, m.p. = 158-160 °C. Analytical data: ^{1}H NMR (400 MHz, d$_6$-Acetone) δ 9.54 (s, 1H), 8.63 (s, 1H), 8.06 (d, *J* = 8.8 Hz, 1H), 7.69 (d, *J* = 9.2 Hz, 1H), 7.66 (d, *J* = 8.0 Hz, 2H), 7.36 (d, *J* = 8.0 Hz, 1H), 7.31 (d, *J* = 8.0 Hz, 2H), 6.97 (d, *J* = 7.6 Hz, 1H), 6.56 (s, 1H), 3.23-3.16 (m, 4H), 2.38 (s, 3H). ^{13}C NMR (100 MHz, d$_6$-Acetone) δ 152.5, 142.8, 138.0, 133.9, 131.1, 129.5, 129.1, 126.8, 126.5, 125.8, 125.0, 124.4, 119.1, 109.4, 107.5, 43.8, 32.4, 20.5. IR (thin film): ν_{max} (cm^{-1}) = 3421,

3228, 2923, 2227, 1699, 1622, 1580, 1429, 1316, 1275, 1153, 1071, 1053, 940, 827, 810, 660. HRMS (ESI) calcd for $C_{20}H_{22}N_3O_3S$ $[M+NH_4]^+$: 384.1376, Found: 384.1373.

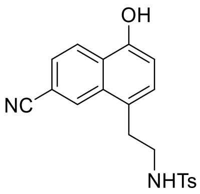

1u, gray solid, m.p. = 207-209 °C. Analytical data: ^{1}H NMR (400 MHz, d_6-Acetone) δ 9.39 (s, 1H), 8.43 (s, 1H), 8.39 (d, *J* = 8.8 Hz, 1H), 7.70-7.63 (m, 3H), 7.36-7.28 (m, 3H), 7.02 (d, *J* = 7.6 Hz, 1H), 6.56 (s, 1H), 3.26-3.20 (m, 4H), 2.38 (s, 3H). ^{13}C NMR (100 MHz, d_6-Acetone) δ 152.2, 142.9, 138.0, 131.8, 129.8, 129.5, 129.4, 126.8, 126.5, 126.4, 124.7, 124.4, 119.1, 110.8, 109.6, 44.1, 32.3, 20.5. IR (thin film): ν_{max} (cm^{-1}) = 3309, 3221, 2920, 2225, 1696, 1599, 1430, 1311, 1283, 1145, 1091, 1064, 940, 834, 812, 662. HRMS (ESI) calcd for $C_{20}H_{22}N_3O_3S$ $[M+NH_4]^+$: 384.1376, Found: 384.1373.

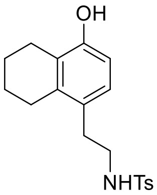

3a, gray solid, m.p. = 162-164 °C. Analytical data: ^{1}H NMR (400 MHz, d_6-Acetone) δ 7.94 (s, 1H), 7.72 (d, *J* = 8.0 Hz, 2H), 7.37 (d, *J* = 8.0 Hz, 2H), 6.73 (d, *J* = 8.0 Hz, 1H), 6.55 (d, *J* = 8.0 Hz, 1H), 6.44 (t, *J* = 5.2 Hz, 1H), 3.04-2.95 (m, 2H), 2.68-2.57 (m, 4H), 2.55-2.48 (m, 2H), 2.40 (s, 3H), 1.72-1.65 (m, 4H). ^{13}C NMR (100 MHz, d_6-Acetone) δ 153.5, 142.8, 138.2, 136.0, 129.5, 127.2, 126.93, 126.86, 124.0, 111.4, 43.7, 32.4, 26.2, 23.4, 22.8, 22.2, 20.5. IR (thin film): ν_{max} (cm^{-1}) =

3336, 3202, 2933, 2863, 1591, 1434, 1313, 1264, 1147, 1091, 1058, 908, 814, 665, 548. HRMS (ESI) calcd for $C_{19}H_{27}N_2O_3S$ $[M+NH_4]^+$: 363.1737, Found: 363.1728.

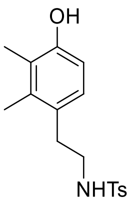

3b, gray solid, m.p. = 149-151 °C. Analytical data: ^{1}H NMR (400 MHz, d_6-Acetone) δ 7.96 (s, 1H), 7.72 (d, *J* = 8.0 Hz, 2H), 7.36 (d, *J* = 8.0 Hz, 2H), 6.74 (d, *J* = 8.0 Hz, 1H), 6.60 (d, *J* = 8.0 Hz, 1H), 6.44 (t, *J* = 5.2 Hz, 1H), 3.00-2.96 (m, 2H), 2.76-2.68 (m, 2H), 2.40 (s, 3H), 2.09 (s, 3H), 2.08 (s, 3H). ^{13}C NMR (100 MHz, d_6-Acetone) δ 153.6, 142.8, 138.2, 135.7, 129.5, 127.5, 127.3, 126.9, 122.9, 112.1, 44.0, 33.9, 20.5, 14.7, 11.3. IR (thin film): ν_{max} (cm^{-1}) = 3347, 3203, 2957, 2881, 1593, 1435, 1314, 1284, 1146, 1090, 1057, 909, 812, 653, 567. HRMS (ESI) calcd for $C_{17}H_{25}N_2O_3S$ $[M+NH_4]^+$: 337.1580, Found: 337.1573.

General procedure for preparation of **2** (and **4**)

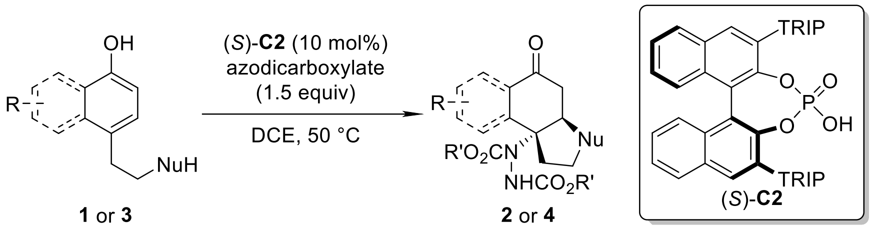

To a flask containing a mixture of **1** (or **3**) (0.1 mmol) and (*S*)-**C2** (7.5 mg, 0.01 mmol) under argon was added a solution of the corresponding azodicarboxylate (0.15 mmol) in anhydrous 1,2-dichloroethane (2 mL). The reaction was stirred at 50 °C until TLC showed complete

consumption of the starting material. The reaction mixture was cooled to room temperature, quenched with $NaHCO_3$ (aq., 10 mL) and extracted with CH_2Cl_2 (3 × 15 mL). The combined organic layer was washed with brine, separated, dried over Na_2SO_4 and filtrated. After the solvent was removed under reduced pressure, the residue was purified by silica gel column chromatography (ethyl acetate/petroleum ether = 1/6 to 1/2) to afford **2** (or **4**).

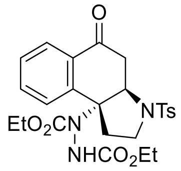

2a, pale yellow oil, 46.0 mg, 90% yield, 99% ee. $[\alpha]_D^{21}$ -173.4 (*c* 2.0, $CHCl_3$). The enantiomeric excess was determined by Daicel Chiralpak AD-H (25 cm), Hexanes/^{i}PrOH = 80/20, 1.0 mL/min^{-1}, λ = 254 nm, t_R (major) = 18.05 min, t_R (minor) = 29.52 min. Analytical data: ^{1}H NMR (600 MHz, $CDCl_3$, 60 °C) δ 8.32 (br s, 1H), 7.84 (d, *J* = 7.8 Hz, 1H), 7.72 (d, *J* = 8.4 Hz, 2H), 7.50 (t, *J* = 7.8 Hz, 1H), 7.37-7.22 (m, 3H), 5.64 (br s, 1H), 4.74 (dd, *J* = 10.2, 5.4 Hz, 1H), 4.29-4.20 (m, 1H), 4.15 (br s, 1H), 3.94-3.79 (m, 2H), 3.66-3.57 (m, 1H), 3.48 (t, *J* = 9.6 Hz, 1H), 3.05 (dd, *J* = 15.6, 6.0 Hz, 1H), 2.67 (dd, *J* = 15.6, 11.4 Hz, 1H), 2.61 (dd, *J* = 14.4, 8.4 Hz, 1H), 2.42 (s, 3H), 2.39-2.30 (m, 1H), 1.29 (t, *J* = 7.2 Hz, 3H), 0.89 (br s, 3H). ^{13}C NMR (150 MHz, $CDCl_3$, 60 °C) δ 194.4, 157.6, 153.9, 144.4, 144.2, 136.0, 134.5, 130.7, 130.1, 127.4, 127.3, 126.9, 126.0, 70.9, 64.2, 62.6, 62.3, 46.4, 44.7, 36.6, 21.3, 14.3, 13.8. IR (thin film): ν_{max} (cm^{-1}) = 3327, 2962, 2921, 1730, 1696, 1514, 1328, 1235, 1159, 1038, 777, 752, 718. HRMS (ESI) calcd for $C_{25}H_{33}N_4O_7S$ $[M+NH_4]^+$: 533.2064, Found: 533.2061.

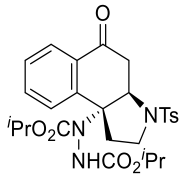

2b, pale yellow oil, 48.0 mg, 89% yield, >99% ee. $[\alpha]_D^{18}$ -146.8 (*c* 2.0, $CHCl_3$). The enantiomeric excess was determined by Daicel Chiralpak IC (25 cm), Hexanes/^{i}PrOH = 60/40, 1.0 mL/min^{-1}, λ = 254 nm, t_R (major) = 17.51 min, t_R (minor) = 21.81 min. Analytical data: ^{1}H NMR (600 MHz, $CDCl_3$, 60 °C) δ 8.38 (br s, 1H), 7.87 (d, *J* = 7.8 Hz, 1H), 7.75 (d, *J* = 8.4 Hz, 2H), 7.53 (t, *J* = 7.2 Hz, 1H), 7.36 (d, *J* = 8.4 Hz, 2H), 7.30 (t, *J* = 7.2 Hz, 1H), 5.40 (br s, 1H), 5.02-4.93 (m, 1H), 4.73 (br s, 1H), 4.66-4.60 (m, 1H), 3.65 (q, *J* = 8.4 Hz, 1H), 3.53 (t, *J* = 9.6 Hz, 1H), 3.13-3.04 (m, 1H), 2.74-2.61 (m, 2H), 2.46 (s, 3H), 2.41-2.34 (m, 1H), 1.30 (d, *J* = 6.4 Hz, 6H), 1.06 (d, *J* = 6.4 Hz, 3H), 0.72 (br s, 3H). ^{13}C NMR (150 MHz, $CDCl_3$, 60 °C) δ 194.5, 157.5, 153.4, 144.8, 144.1, 136.0, 134.6, 130.8, 130.2, 127.4, 127.4, 126.9, 126.0, 70.7, 70.7, 70.2, 64.6, 46.4, 44.9, 36.7, 21.9, 21.9, 21.8, 21.7, 21.4, 21.1. IR (thin film): ν_{max} (cm^{-1}) = 3242, 2980, 2919, 1744, 1703, 1676, 1451, 1373, 1243, 1162, 1106, 770, 719, 666. HRMS (ESI) calcd for $C_{27}H_{37}N_4O_7S$ $[M+NH_4]^+$: 561.2377, Found: 561.2367.

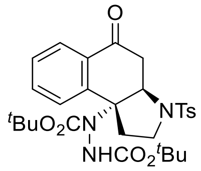

2c, pale yellow oil, 50.0 mg, 88% yield, >99% ee. $[\alpha]_D^{19}$ -136.9 (*c* 2.0, $CHCl_3$). The enantiomeric excess was determined by Daicel Chiralpak IC (25 cm), Hexanes/^{i}PrOH = 60/40, 1.0 mL/min^{-1}, λ = 254 nm, t_R (major) = 14.99 min, t_R (minor) = 17.11 min. Analytical data: ^{1}H NMR (600 MHz, $CDCl_3$, 60 °C) δ 8.42 (br s, 1H), 7.87 (d, *J* = 7.6 Hz, 1H), 7.75 (d, *J* = 8.0 Hz, 2H), 7.55 (s, 1H), 7.35 (d, *J* = 8.0 Hz, 2H), 7.30 (t, *J* = 7.6 Hz, 1H), 4.71 (br s, 1H), 3.65-3.54 (m, 2H), 3.17-3.08 (m, 1H), 2.68 (dd,

J = 15.6, 11.6 Hz, 1H), 2.59 (dd, J = 14.0, 8.0 Hz, 1H), 2.46 (s, 3H), 2.39-2.32 (m, 1H), 1.50 (s, 9H), 1.11 (s, 9H). ^{13}C NMR (150 MHz, $CDCl_3$, 60 °C) δ 194.6, 156.8, 152.5, 145.3, 143.9, 136.1, 134.5, 130.7, 130.2, 127.6, 127.2, 126.8, 126.0, 82.5, 81.4, 70.4, 64.7, 46.5, 45.2, 36.9, 28.2, 27.7, 21.4. IR (thin film): ν_{max} (cm^{-1}) = 3354, 2978, 2930, 1747, 1703, 1691, 1481, 1349, 1244, 1150, 912, 728, 663. HRMS (ESI) calcd for $C_{29}H_{41}N_4O_7S$ [M+NH$_4$]$^+$: 589.2690, Found: 589.2682.

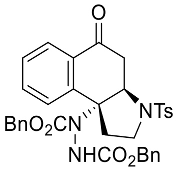

2d, pale yellow oil, 57.0 mg, 89% yield, >99% ee. $[\alpha]_D^{20}$ -121.9 (c 2.0, $CHCl_3$). The enantiomeric excess was determined by Daicel Chiralpak AD-H (25 cm), Hexanes/^{i}PrOH = 60/40, 1.0 mL/min^{-1}, λ = 254 nm, t_R (major) = 20.53 min, t_R (minor) = 28.08 min. Analytical data: ^{1}H NMR (600 MHz, CD_3CN, 80 °C) δ 8.17 (br s, 1H), 7.89-7.81 (m, 1H), 7.76 (d, J = 8.0 Hz, 2H), 7.60-7.30 (m, 12H), 7.13 (d, J = 3.2 Hz, 2H), 5.98 (br s, 1H), 5.23 (d, J = 12.0 Hz, 1H), 5.14 (d, J = 12.0 Hz, 1H), 4.96-4.85 (m, 2H), 4.63 (s, 1H), 3.58-3.45 (m, 1H), 3.36 (br s, 1H), 3.13-2.94 (m, 2H), 2.53 (br s, 1H), 2.45-2.32 (m, 1H), 2.32 (s, 3H). ^{13}C NMR (150 MHz, CD_3CN, 80 °C) δ 193.6, 156.0, 153.0, 143.5, 142.9, 135.0, 134.6, 133.0, 130.5, 129.2, 127.5, 127.3, 127.2, 127.0, 126.6, 126.5, 126.2, 125.9, 124.4, 69.8, 66.9, 66.6, 62.7, 45.3, 42.9, 35.5, 19.3. IR (thin film): ν_{max} (cm^{-1}) = 3342, 2978, 2920, 1717, 1689, 1494, 1336, 1214, 1160, 1026, 737, 696, 663. HRMS (ESI) calcd for $C_{35}H_{37}N_4O_7S$ [M+NH$_4$]$^+$: 657.2377, Found: 657.2375.

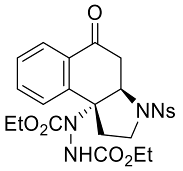

2e, pale yellow oil, 49.0 mg, 89% yield, 98% ee. $[\alpha]_D^{17}$ -112.5 (*c* 2.0, $CHCl_3$). The enantiomeric excess was determined by Daicel Chiralpak AD-H (25 cm), Hexanes/^{i}PrOH = 60/40, 1.0 mL/min^{-1}, λ = 254 nm, t_R (major) = 8.94 min, t_R (minor) = 13.36 min. Analytical data: ^{1}H NMR (600 MHz, CD_3CN, 80 °C) δ 8.42 (br s, 2H), 8.13 (br s, 2H), 7.95-7.86 (m, 1H), 7.70-7.62 (m, 1H), 7.56-7.40 (m, 2H), 6.51 (br, 1H), 4.76-4.67 (m, 1H), 4.34-4.05 (m, 2H), 3.99-3.88 (m, 2H), 3.71-3.04 (m, 4H), 2.64-2.20 (m, 2H), 1.38-1.18 (m, 3H), 1.05-0.96 (m, 3H). ^{13}C NMR (150 MHz, CD_3CN, 80 °C, two rotamers) δ 194.6, 194.4, 155.7, 155.6, 153.7, 153.5, 144.3, 132.9, 131.1, 130.6, 126.3, 126.2, 126.1, 124.8, 124.1, 61.1, 61.0, 60.9, 60.3, 50.6, 50.5, 43.9, 43.6, 41.7, 41.1, 34.8, 34.5, 12.7, 12.6, 12.2. IR (thin film): ν_{max} (cm^{-1}) = 3335, 2981, 2920, 1739, 1709, 1687, 1526, 1345, 1263, 1161, 1093, 739, 627. HRMS (ESI) calcd for $C_{24}H_{27}N_4O_9S$ $[M+H]^+$: 547.1493, Found: 547.1488.

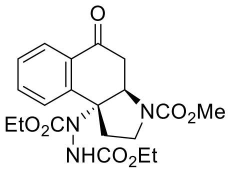

2f, pale yellow oil, 38.0 mg, 90% yield, 99% ee. $[\alpha]_D^{22}$ -227.9 (*c* 2.0, $CHCl_3$). The enantiomeric excess was determined by Daicel Chiralpak IC (25 cm), Hexanes/^{i}PrOH = 70/30, 0.7 mL/min^{-1}, λ = 214 nm, t_R (major) = 38.82 min, t_R (minor) = 45.34 min. Analytical data: ^{1}H NMR (600 MHz, CD_3CN, 80 °C, two rotamers) δ 8.41-7.01 (m, 5H), 4.97-4.70 (m, 1H), 4.34-4.05 (m, 2H), 3.93-3.32 (m, 6H), 3.22-3.05 (m, 1H), 2.90-2.66 (m, 2H), 2.47-2.33 (m, 1H), 1.44-0.81 (m, 6H). ^{13}C NMR (150 MHz, CD_3CN, 80 °C, two rotamers) δ 194.6, 194.4, 156.8, 155.6, 153.7, 153.5, 144.3, 132.9, 132.9,

131.1, 130.6, 126.3, 126.2, 126.1, 124.8, 124.1, 61.1, 61.0, 60.9, 60.8, 60.3, 60.3, 50.6, 50.5, 43.9, 43.6, 41.7, 41.1, 34.8, 34.5, 12.7, 12.6, 12.6, 12.2. IR (thin film): ν_{max} (cm^{-1}) = 3244, 2987, 2959, 1730, 1714, 1693, 1531, 1542, 1392, 1246, 1161, 1066, 766, 733. HRMS (ESI) calcd for $C_{20}H_{29}N_4O_7$ $[M+NH_4]^+$: 437.2031, Found: 437.2024.

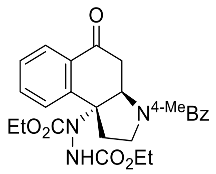

2g, pale yellow oil, 44.0 mg, 92% yield, 98% ee. $[\alpha]_D^{21}$ -175.9 (*c* 2.0, $CHCl_3$). The enantiomeric excess was determined by Daicel Chiralpak IG (25 cm), Hexanes/^{i}PrOH = 50/50, 0.7 mL/min^{-1}, λ = 214 nm, t_R (major) = 59.23 min, t_R (minor) = 42.09 min. Analytical data: ^{1}H NMR (600 MHz, CD_3CN, 80 °C, two rotamers) δ 8.45-7.75 (m, 2H), 7.69-7.50 (m, 2H), 7.45-7.36 (m, 3H), 7.28-7.22 (m, 2H), 5.27-4.85 (m, 1H), 4.34-4.19 (m, 2H), 3.94-2.79 (m, 8H), 2.49-2.41 (m, 1H), 2.40 (s, 3H), 1.38-1.26 (m, 3H), 1.10-0.86 (m, 3H). ^{13}C NMR (150 MHz, CD_3CN, 80 °C, two rotamers) δ 194.2, 168.8, 168.4, 156.7, 155.7, 153.8, 153.6, 144.2, 144.0, 139.2, 138.7, 133.0, 127.7, 127.6, 126.4, 126.3, 126.2, 125.9, 125.8, 124.8, 124.3, 61.1, 60.9, 45.5, 40.6, 35.3, 19.2, 12.7, 12.2. IR (thin film): ν_{max} (cm^{-1}) = 3290, 2923, 2854, 1748, 1690, 1599, 1449, 1209, 1154, 1045, 765, 698; HRMS (ESI) calcd for $C_{26}H_{30}N_3O_6$ $[M+H]^+$: 480.2129, Found: 480.2125.

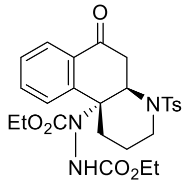

2h, pale yellow oil, 49.0 mg, 93% yield, 98% ee. $[\alpha]_D^{17}$ +12.8 (*c* 2.0, $CHCl_3$). The enantiomeric excess was determined by Daicel Chiralpak OJ-H (25 cm), CO_2/MeOH = 90/10, λ = 214 nm, background press = 2000 psi, T = 40 °C, t_R (major) = 6.08 min, t_R (minor) = 6.59 min. Analytical data: ^{1}H NMR (600 MHz, $CDCl_3$, 60 °C, two rotamers) δ 8.11-8.02 (m, 1H), 7.60-7.22 (m, 6H), 6.74-6.33 (m, 1H), 4.85-4.71 (m, 1H), 4.33-4.12 (m, 3H), 3.89-3.74 (m, 2H), 2.77-2.66 (m, 2H), 2.40-2.36 (m, 3H), 2.25-1.78 (m, 3H), 1.39-1.09 (m, 6H), 0.83-0.67 (m, 3H). ^{13}C NMR (150 MHz, $CDCl_3$, 60 °C, two rotamers) δ 184.1, 157.5, 156.6, 154.9, 154.7, 150.5, 148.0, 145.7, 143.2, 143.1, 142.9, 137.1, 133.2, 132.9, 131.6, 131.2, 129.5, 129.4, 129.4, 129.2, 128.5, 127.3, 127.2, 126.8, 126.8, 126.3, 126.1, 124.2, 64.3, 64.0, 62.4, 62.3, 62.3, 62.0, 42.6, 35.9, 35.8, 23.6, 23.5, 21.2, 21.2, 14.3, 14.2, 14.2, 13.6, 13.5. IR (thin film): ν_{max} (cm^{-1}) = 3327, 2962, 2921, 1730, 1696, 1514, 1328, 1235, 1159, 1038, 777, 752, 718. HRMS (ESI) calcd for $C_{26}H_{35}N_4O_7S$ $[M+NH_4]^+$: 547.2221, Found: 547.2216.

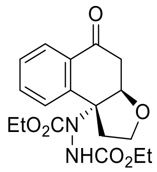

2i, pale yellow oil, 31.0 mg, 87% yield, >99% ee. $[\alpha]_D^{21}$ -222.7 (*c* 2.0, $CHCl_3$). The enantiomeric excess was determined by Daicel Chiralpak IC (25 cm), Hexanes/^{i}PrOH = 70/30, 0.7 mL/min^{-1}, λ = 214 nm, t_R (major) = 33.83 min, t_R (minor) = 20.90 min. ^{1}H NMR (600 MHz, CD_3CN, 80 °C, two rotamers) δ 8.16, 7.74 (br s, 1H), 7.89, 7.05 (d, *J* = 7.8 Hz, 1H), 7.69-7.60 (m, 1H), 7.64, 7.45 (br s, 1H), 7.41 (t, *J* = 7.8 Hz, 1H), 4.81, 4.58 (t, *J* = 4.8 Hz, 1H), 4.31-4.16 (m, 2H), 4.04-3.94 (m, 2H), 3.91, 3.79 (dd, *J* = 15.0, 7.8 Hz, 1H), 3.71, 3.58 (dd, *J* = 15.0, 7.2 Hz, 1H), 3.34, 3.15 (dd, *J* = 16.2,

4.8 Hz, 1H), 3.03, 2.94 (dt, J = 13.2, 6.6 Hz, 1H), 2.87-2.82 (m, 1H), 2.39-2.26 (m, 1H), 1.34-1.25 (m, 3H), 1.09-1.02 (m, 3H). ^{13}C NMR (150 MHz, CD_3CN, 80 °C, two rotamers) δ 194.7, 194.5, 156.2, 155.8, 154.3, 154.2, 144.0, 132.9, 132.8, 131.0, 130.4, 126.7, 126.1, 126.0, 125.6, 124.2, 124.1, 79.7, 79.5, 68.4, 67.7, 64.7, 64.2, 61.0, 60.8, 60.3, 40.6, 40.2, 39.1, 12.7, 12.6, 12.3, 12.2. IR (thin film): ν_{max} (cm^{-1}) = 3233, 2984, 2890, 1737, 1713, 1676, 1598, 1534, 1241, 1054, 763, 698. HRMS (ESI) calcd for $C_{18}H_{26}N_3O_6$ $[M+NH_4]^+$: 380.1816, Found: 380.1809.

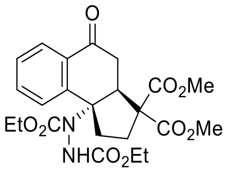

2j, pale yellow oil, 39.0 mg, 81% yield, 99% ee. $[\alpha]_D^{18}$ +18.3 (c 2.0, $CHCl_3$). The enantiomeric excess was determined by Daicel Chiralpak IC (25 cm), Hexanes/^{i}PrOH = 60/40, 0.7 mL/min^{-1}, λ = 214 nm, t_R (major) = 43.73 min, t_R (minor) = 31.30 min. ^1H NMR (600 MHz, CD_3CN, 80 °C, two rotamers) δ 7.98 (br s), 7.47 (d, J = 8.0 Hz) (1H), 7.92-7.84 (m, 1H), 7.65-7.55 (m, 1H), 7.37 (t, J = 7.6 Hz, 1H), 7.07, 7.01 (br s, 1H), 4.21-4.10 (m, 2H), 4.11-4.07, 3.85-3.81 (m, 1H), 4.02-3.92 (m, 2H), 3.75, 3.73 (s, 3H), 3.48, 3.44 (s, 3H), 3.24-3.13 (m, 1H), 2.88-2.79, 2.71-2.66 (m, 2H), 2.38-2.15 (m, 3H), 1.27-1.22 (m, 3H), 1.07-1.02 (m, 3H). ^{13}C NMR (150 MHz, CD_3CN, 80 °C, two rotamers) δ 194.38, 194.35, 171.2, 170.8, 170.2, 169.8, 156.7, 156.1, 154.9, 154.7, 145.6, 133.1, 133.0, 131.4, 131.2, 127.5, 126.65, 126.56, 126.3, 124.6, 124.5, 70.0, 69.3, 67.5, 65.7, 63.0, 62.8, 61.50, 61.46, 61.3, 60.9, 51.9, 51.6, 51.1, 50.9, 48.6, 48.1, 38.1, 37.6, 36.6, 36.5, 31.3, 13.2, 12.91, 12.87. IR (thin film): ν_{max} (cm^{-1}) = 3299, 2984, 2955, 1722, 1663, 1300, 1237, 1154, 1056, 763, 733, 702. HRMS (ESI) calcd for $C_{23}H_{32}N_3O_9$ $[M+NH_4]^+$: 494.2133, Found: 494.2126.

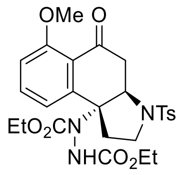

2k, pale yellow oil, 50.0 mg, 91% yield, 98% ee. $[\alpha]_D^{19}$ -144.0 (*c* 2.0, $CHCl_3$). The enantiomeric excess was determined by Daicel Chiralpak IC (25 cm), Hexanes/^{i}PrOH/Et_2NH = 50/50/0.1, 0.7 mL/min^{-1}, λ = 254 nm, t_R (major) = 53.01 min, t_R (minor) = 79.74 min. Analytical data: ^{1}H NMR (600 MHz, $CDCl_3$, 60 °C) δ 7.89 (br s, 1H), 7.75 (d, *J* = 7.8 Hz, 2H), 7.42 (t, *J* = 7.8 Hz, 1H), 7.36 (d, *J* = 8.4 Hz, 2H), 6.83 (d, *J* = 8.4 Hz, 1H), 5.43 (br s, 1H), 4.71-4.65 (m, 1H), 4.28-4.13 (m, 2H), 3.96-3.87 (m, 2H), 3.85 (s, 3H), 3.60-3.49 (m, 2H), 2.99 (dd, *J* = 13.8, 5.7 Hz, 1H), 2.75 (t, *J* = 12.4 Hz, 1H), 2.60 (dd, *J* = 14.2, 7.8 Hz, 1H), 2.45 (s, 3H), 2.41-2.35 (m, 1H), 1.35-1.25 (m, 3H), 1.05-0.85 (m, 3H). ^{13}C NMR (150 MHz, $CDCl_3$, 60 °C) δ 193.5, 158.2, 157.6, 153.8, 146.4, 144.0, 135.8, 134.4, 130.1, 126.9, 121.4, 119.3, 111.0, 71.3, 64.8, 62.6, 62.2, 56.1, 47.5, 46.3, 37.1, 21.3, 14.2, 13.8. IR (thin film): ν_{max} (cm^{-1}) = 3342, 2981, 2926, 1760, 1707, 1594, 1375, 1227, 1159, 1093, 749, 663. HRMS (ESI) calcd for $C_{26}H_{35}N_4O_8S$ $[M+NH_4]^+$: 563.2170, Found: 563.2166.

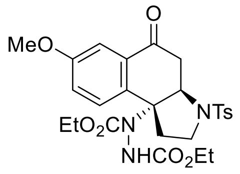

2l, pale yellow oil, 50.0 mg, 91% yield, >99% ee. $[\alpha]_D^{16}$ -158.1 (*c* 2.0, $CHCl_3$). The enantiomeric excess was determined by Daicel Chiralpak IC (25 cm), Hexanes/^{i}PrOH/Et_2NH = 60/40/0.1, 0.7 mL/min^{-1}, λ = 254 nm, t_R (major) = 53.03 min, t_R (minor) = 71.66 min. Analytical data: ^{1}H NMR (600 MHz, $CDCl_3$, 60 °C) δ 8.24 (br s, 1H), 7.75 (d, *J* = 8.4 Hz, 2H), 7.35 (d, *J* = 8.4 Hz, 3H), 7.10

(d, J = 6.6 Hz, 1H), 5.66 (br s, 1H), 4.79-4.73 (m, 1H), 4.30-4.23 (m, 1H), 4.20-4.13 (m, 1H), 3.96-3.85 (m, 2H), 3.80 (s, 3H), 3.67-3.60 (m, 1H), 3.49 (t, J = 9.0 Hz, 1H), 3.07 (dd, J = 15.6, 6.0 Hz, 1H), 2.71-2.59 (m, 2H), 2.45 (s, 3H), 2.38-2.30 (m, 1H), 1.31 (t, J = 7.2 Hz, 3H), 0.97 (s, 3H). ^{13}C NMR (150 MHz, $CDCl_3$, 60 °C) δ 194.1, 158.9, 157.6, 153.9, 144.0, 136.8, 136.0, 131.7, 130.0, 128.7, 126.9, 122.3, 108.6, 70.7, 64.1, 62.5, 62.2, 55.3, 46.4, 44.6, 36.7, 21.3, 14.2, 13.8. IR (thin film): ν_{max} (cm^{-1}) = 3331, 2986, 2912, 1745, 1712, 1693, 1596, 1292, 1239, 1163, 1028, 741, 662. HRMS (ESI) calcd for $C_{26}H_{35}N_4O_8S$ $[M+NH_4]^+$: 563.2170, Found: 563.2166.

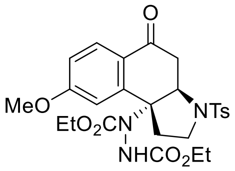

2m, pale yellow oil, 50.0 mg, 91% yield, 99% ee. $[\alpha]_D^{20}$ -111.2 (*c* 2.0, $CHCl_3$). The enantiomeric excess was determined by Daicel Chiralpak IC (25 cm), Hexanes/^{i}PrOH/Et_2NH = 60/40/0.1, 0.7 mL/min^{-1}, λ = 254 nm, t_R (major) = 58.27 min, t_R (minor) = 76.07 min. Analytical data: ^{1}H NMR (600 MHz, $CDCl_3$, 60 °C) δ 8.05 (br s, 1H), 7.85 (d, J = 8.4 Hz, 1H), 7.74 (d, J = 8.4 Hz, 2H), 7.34 (d, J = 7.8 Hz, 2H), 6.81 (dd, J = 8.4, 1.9 Hz, 1H), 5.78 (br s, 1H), 4.79 (br s, 1H), 4.30-4.24 (m, 1H), 4.17 (br s, 1H), 3.92 (br s, 2H), 3.84 (s, 3H), 3.72-3.64 (m, 1H), 3.50 (t, J = 9.6 Hz, 1H), 3.00 (dd, J = 15.6, 5.4 Hz, 1H), 2.65-2.55 (m, 2H), 2.44 (s, 3H), 2.39-2.32 (m, 1H), 1.31 (t, J = 6.6 Hz, 3H), 0.95 (br s, 3H). ^{13}C NMR (150 MHz, $CDCl_3$, 60 °C) δ 192.5, 165.0, 157.6, 153.8, 147.0, 144.0, 136.1, 130.0, 128.5, 126.9, 123.9, 114.9, 110.9, 71.1, 64.1, 62.5, 62.2, 55.6, 46.0, 44.1, 36.6, 21.3, 14.2, 13.8. IR (thin film): ν_{max} (cm^{-1}) = 3338, 2978, 2920, 1758, 1714, 1688, 1599, 1489, 1225, 1160, 1018, 821, 760, 663. HRMS (ESI) calcd for $C_{26}H_{35}N_4O_8S$ $[M+NH_4]^+$: 563.2170, Found: 563.2166.

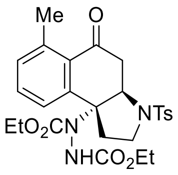

2n, pale yellow oil, 49.0 mg, 92% yield, 99% ee. $[\alpha]_D^{18}$ -151.1 (*c* 2.0, $CHCl_3$). The enantiomeric excess was determined by Daicel Chiralpak IC (25 cm), Hexanes/^{i}PrOH/Et_2NH = 50/50/0.1, 0.7 mL/min^{-1}, λ = 254 nm, t_R (major) = 31.87 min, t_R (minor) = 45.77 min. Analytical data: ^{1}H NMR (600 MHz, $CDCl_3$, 60 °C) δ 8.15 (br s, 1H), 7.77 (d, *J* = 8.4 Hz, 2H), 7.36 (t, *J* = 7.2 Hz, 3H), 7.09 (d, *J* = 7.2 Hz, 1H), 5.45 (br s, 1H), 4.71 (dd, *J* = 11.4, 5.4 Hz, 1H), 4.31-4.24 (m, 1H), 4.20-4.13 (m, 1H), 3.97-3.81 (m, 2H), 3.60 (dd, *J* = 18.0, 9.0 Hz, 1H), 3.53 (t, *J* = 9.0 Hz, 1H), 3.01 (dd, *J* = 14.4, 5.4 Hz, 1H), 2.78-2.71 (m, 1H), 2.64 (dd, *J* = 14.4, 8.4 Hz, 1H), 2.55 (s, 3H), 2.45 (s, 3H), 2.43-2.36 (m, 1H), 1.32 (t, *J* = 7.2 Hz, 3H), 0.91 (br s, 3H). ^{13}C NMR (150 MHz, $CDCl_3$, 60 °C) δ 196.6, 157.6, 153.9, 145.3, 144.0, 138.8, 135.8, 132.9, 130.8, 130.1, 130.0, 126.9, 125.2, 71.5, 64.5, 62.5, 62.2, 47.3, 46.4, 37.0, 21.8, 21.3, 14.2, 13.6. IR (thin film): ν_{max} (cm^{-1}) = 3335, 2979, 2920, 1720, 1691, 1450, 1354, 1215, 1170, 1039, 756, 669. HRMS (ESI) calcd for $C_{26}H_{35}N_4O_7S$ $[M+NH_4]^+$: 547.2221, Found: 547.2216.

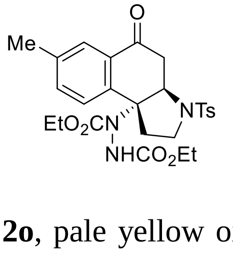

2o, pale yellow oil, 51.0 mg, 93% yield, >99% ee. $[\alpha]_D^{18}$ -154.2 (*c* 2.0, $CHCl_3$). The enantiomeric excess was determined by Daicel Chiralpak IC (25 cm), Hexanes/^{i}PrOH/Et_2NH = 60/40/0.1, 0.7 mL/min^{-1}, λ = 254 nm, t_R (major) = 49.64 min, t_R (minor) = 72.23 min. Analytical data: ^{1}H NMR

(600 MHz, $CDCl_3$, 60 °C) δ 8.21 (br s, 1H), 7.75 (d, *J* = 8.4 Hz, 2H), 7.69 (s, 1H), 7.35 (d, *J* = 7.8 Hz, 3H), 5.69 (br s, 1H), 4.77 (dd, *J* = 10.8, 5.4 Hz, 1H), 4.32-4.24 (m, 1H), 4.18 (br s, 1H), 3.96-3.84 (m, 2H), 3.65 (dd, *J* = 18.0, 9.0 Hz, 1H), 3.49 (t, *J* = 9.0 Hz, 1H), 3.05 (dd, *J* = 16.2, 6.0 Hz, 1H), 2.71-2.60 (m, 2H), 2.45 (s, 3H), 2.39-2.30 (m, 1H), 2.33 (s, 3H), 1.32 (t, *J* = 7.2 Hz, 3H), 0.95 (br s, 3H). ^{13}C NMR (150 MHz, $CDCl_3$, 60 °C) δ 194.4, 157.6, 153.9, 144.0, 141.5, 137.2, 136.0, 135.5, 130.4, 130.0, 127.1, 126.9, 126.2, 70.8, 64.1, 62.5, 62.2, 46.3, 44.6, 36.6, 21.3, 20.6, 14.2, 13.8. IR (thin film): ν_{max} (cm^{-1}) = 3341, 2981, 2923, 1717, 1689, 1493, 1374, 1235, 1160, 1044, 751, 664. HRMS (ESI) calcd for $C_{26}H_{35}N_4O_7S$ $[M+NH_4]^+$: 547.2221, Found: 547.2216.

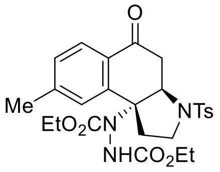

2p, pale yellow oil, 49.0 mg, 92% yield, 98% ee. $[\alpha]_D^{27}$ -155.2 (*c* 2.0, $CHCl_3$). The enantiomeric excess was determined by Daicel Chiralpak IC (25 cm), Hexanes/^{i}PrOH = 60/40, 0.7 mL/min^{-1}, λ = 214 nm, t_R (major) = 58.29 min, t_R (minor) = 73.75 min. Analytical data: ^1H NMR (600 MHz, $CDCl_3$, 60 °C) δ 8.14 (s, 1H), 7.78 (d, *J* = 7.8 Hz, 1H), 7.74 (d, *J* = 8.4 Hz, 2H), 7.34 (d, *J* = 8.4 Hz, 2H), 7.10 (d, *J* = 7.8 Hz, 1H), 5.72 (br s, 1H), 4.76 (dd, *J* = 10.8, 6.0 Hz, 1H), 4.32-4.24 (m, 1H), 4.23-4.14 (m, 1H), 3.90 (q, *J* = 6.6 Hz, 2H), 3.66 (q, *J* = 9.0 Hz, 1H), 3.49 (t, *J* = 9.6 Hz, 1H), 3.02 (dd, *J* = 16.2, 6.0 Hz, 1H), 2.68-2.59 (m, 2H), 2.46 (s, 3H), 2.40-2.32 (m, 1H), 2.37 (s, 3H), 1.31 (t, *J* = 6.6 Hz, 3H), 0.93 (br s, 3H). ^{13}C NMR (150 MHz, $CDCl_3$, 60 °C) δ 193.8, 157.5, 153.9, 145.5, 144.3, 144.0, 136.1, 130.0, 128.4, 128.3, 127.4, 126.9, 126.2, 70.9, 64.1, 62.4, 62.1, 46.2, 44.4, 36.6, 21.7, 21.3, 14.3, 13.7. IR (thin film): ν_{max} (cm^{-1}) = 3342, 2982, 2924, 1744, 1717, 1685, 1607, 1492,

1330, 1228, 1159, 1091, 725, 664. HRMS (ESI) calcd for $C_{26}H_{35}N_4O_7S$ $[M+NH_4]^+$: 547.2221, Found: 547.2216.

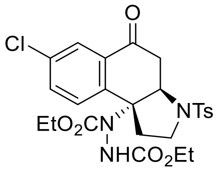

2q, pale yellow oil, 51.0 mg, 93% yield, >99% ee. $[\alpha]_D^{19}$ -150.9 (*c* 2.0, $CHCl_3$). The enantiomeric excess was determined by Daicel Chiralpak IC (25 cm), Hexanes/^{i}PrOH = 50/50, 0.7 mL/min^{-1}, λ = 254 nm, t_R (major) = 22.61 min, t_R (minor) = 29.24 min. Analytical data: ^{1}H NMR (600 MHz, $CDCl_3$, 60 °C) δ 8.35 (br s, 1H), 7.84 (d, *J* = 1.2 Hz, 1H), 7.75 (d, *J* = 8.4 Hz, 2H), 7.48 (dd, *J* = 8.4, 1.8 Hz, 1H), 7.36 (d, *J* = 7.8 Hz, 2H), 5.76 (br s, 1H), 4.80-4.74 (m, 1H), 4.34-4.26 (m, 1H), 4.18 (br s, 1H), 3.99-3.87 (m, 2H), 3.68 (dd, *J* = 18.0, 9.0 Hz, 1H), 3.51 (t, *J* = 9.6 Hz, 1H), 3.07 (dd, *J* = 16.2, 5.4 Hz, 1H), 2.70-2.59 (m, 2H), 2.45 (s, 3H), 2.37-2.30 (m, 1H), 1.32 (t, *J* = 7.2 Hz, 3H), 1.00 (br s, 3H). ^{13}C NMR (150 MHz, $CDCl_3$, 60 °C) δ 193.1, 157.6, 153.7, 144.2, 142.7, 135.9, 134.4, 133.9, 131.9, 130.0, 129.1, 126.9, 125.8, 70.5, 63.9, 62.7, 62.4, 46.3, 44.5, 36.5, 21.3, 14.2, 13.8. IR (thin film): ν_{max} (cm^{-1}) = 3348, 2978, 2916, 1749, 1724, 1703, 1501, 1332, 1217, 1165, 1060, 826, 664. HRMS (ESI) calcd for $C_{25}H_{32}ClN_4O_7S$ $[M+NH_4]^+$: 567.1675, Found: 567.1669.

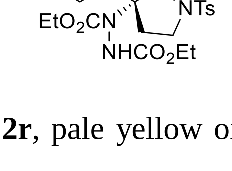

2r, pale yellow oil, 53.0 mg, 90% yield, >99% ee. $[\alpha]_D^{28}$ -181.5 (*c* 2.0, $CHCl_3$). The enantiomeric excess was determined by Daicel Chiralpak IC (25 cm), Hexanes/^{i}PrOH = 60/40, 0.7 mL/min^{-1}, λ =

214 nm, t_R (major) = 30.67 min, t_R (minor) = 41.59 min. Analytical data: ^{1}H NMR (600 MHz, $CDCl_3$, 60 °C) δ 8.28 (br s, 1H), 7.99 (d, *J* = 1.8 Hz, 1H), 7.74 (d, *J* = 8.4 Hz, 2H), 7.63 (dd, *J* = 8.4, 1.8 Hz, 1H), 7.35 (d, *J* = 7.8 Hz, 2H), 5.74 (br s, 1H), 4.80-4.73 (m, 1H), 4.31-4.24 (m, 1H), 4.22-4.14 (m, 1H), 3.99-3.88 (m, 2H), 3.67 (q, *J* = 9.0 Hz, 1H), 3.50 (t, *J* = 9.6 Hz, 1H), 3.06 (dd, *J* = 16.2, 6.0 Hz, 1H), 2.69-2.60 (m, 2H), 2.45 (s, 3H), 2.37-2.29 (m, 1H), 1.32 (t, *J* = 7.2 Hz, 3H), 1.00 (br s, 3H). ^{13}C NMR (150 MHz, $CDCl_3$, 60 °C) δ 193.0, 157.6, 153.7, 144.2, 143.2, 137.3, 135.9, 132.1, 130.0, 129.3, 128.9, 126.9, 121.8, 70.5, 63.8, 62.7, 62.3, 46.2, 44.4, 36.4, 21.3, 14.2, 13.8. IR (thin film): ν_{max} (cm^{-1}) = 3344, 2982, 2906, 1746, 1715, 1693, 1590, 1476, 1331, 1235, 1160, 1091, 914, 815, 727, 664. HRMS (ESI) calcd for $C_{25}H_{32}BrN_4O_7S$ $[M+NH_4]^+$: 611.1170, Found: 611.1170.

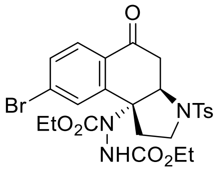

2s, pale yellow oil, 54.0 mg, 92% yield, 98% ee. $[\alpha]_D^{28}$ -119.4 (*c* 2.0, $CHCl_3$). The enantiomeric excess was determined by Daicel Chiralpak IA (25 cm), Hexanes/^{i}PrOH/CH_3CN = 80/18/2, 0.7 mL/min^{-1}, λ = 214 nm, t_R (major) = 37.00 min, t_R (minor) = 55.38 min. Analytical data: ^{1}H NMR (600 MHz, $CDCl_3$, 60 °C) δ 8.62 (br s, 1H), 7.74 (d, *J* = 7.8 Hz, 3H), 7.45 (d, *J* = 8.4 Hz, 1H), 7.35 (d, *J* = 7.8 Hz, 2H), 5.74 (br s, 1H), 4.80-4.74 (m, 1H), 4.34-4.27 (m, 1H), 4.26-4.15 (m, 1H), 3.95 (q, *J* = 6.6 Hz, 2H), 3.68 (q, *J* = 9.0 Hz, 1H), 3.51 (t, *J* = 9.6 Hz, 1H), 3.05 (dd, *J* = 16.2, 6.0 Hz, 1H), 2.68-2.59 (m, 2H), 2.45 (s, 3H), 2.39-2.31 (m, 1H), 1.33 (br s, 3H), 1.00 (br s, 3H). ^{13}C NMR (150 MHz, $CDCl_3$, 60 °C) δ 193.3, 157.4, 153.7, 146.0, 144.2, 135.9, 130.9, 130.3, 130.0, 129.7, 129.4, 127.7, 126.9, 70.4, 63.8, 62.7, 62.4, 46.2, 44.4, 36.5, 21.3, 14.3, 13.8. IR (thin film): ν_{max} (cm^{-1}) =

3344, 2982, 2927, 1747, 1716, 1692, 1586, 1491, 1334, 1234, 1160, 1088, 913, 815, 728, 664. HRMS (ESI) calcd for $C_{25}H_{32}BrN_4O_7S$ $[M+NH_4]^+$: 611.1170, Found: 611.1168.

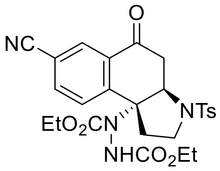

2t, pale yellow oil, 50.0 mg, 93% yield, >99% ee. $[\alpha]_D^{28}$ -178.0 (*c* 2.0, $CHCl_3$). The enantiomeric excess was determined by Daicel Chiralpak IC (25 cm), Hexanes/^{i}PrOH = 50/50, 0.7 mL/min^{-1}, λ = 214 nm, t_R (major) = 78.73 min, t_R (minor) = 64.39 min. Analytical data: ^{1}H NMR (600 MHz, CD_3CN, 80 °C, two rotamers) δ 8.43 (br s, 1H), 8.16 (s, 1H), 7.90 (d, *J* = 8.4 Hz, 1H), 7.80 (d, *J* = 5.4 Hz, 2H), 7.48 (d, *J* = 5.4 Hz, 2H), 7.02, 5.91 (s, 1H), 4.67-4.53 (m, 1H), 4.33-4.17 (m, 2H), 3.97-3.87 (m, 2H), 3.64-3.54 (m, 1H), 3.45-3.30 (m, 1H), 3.16-2.97 (m, 2H), 2.64-2.45 (m, 1H), 2.49 (s, 3H), 2.43-2.35 (m, 1H), 1.31 (br s, 3H), 1.01 (br s, 3H). ^{13}C NMR (150 MHz, CD_3CN, 80 °C, two rotamers) δ 193.0, 157.0, 156.2, 153.7, 148.5, 144.3, 136.2, 135.1, 131.8, 129.9, 129.2, 128.3, 126.6, 117.6, 111.2, 69.9, 63.3, 62.3, 61.9, 61.0, 45.9, 43.4, 35.7, 20.1, 13.33, 13.30, 12.9. IR (thin film): ν_{max} (cm^{-1}) = 3343, 2920, 2849, 2232, 1747, 1716, 1699, 1490, 1333, 1236, 1160, 1091, 817, 728, 663. HRMS (ESI) calcd for $C_{26}H_{32}N_5O_7S$ $[M+NH_4]^+$: 558.2017, Found: 558.2014.

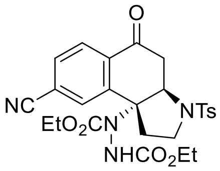

2u, pale yellow oil, 49.0 mg, 91% yield, 98% ee. $[\alpha]_D^{28}$ -150.3 (*c* 2.0, $CHCl_3$). The enantiomeric excess was determined by Daicel Chiralpak IA (25 cm), Hexanes/^{i}PrOH = 60/40, 0.7 mL/min^{-1}, λ =

214 nm, t_R (major) = 9.03 min, t_R (minor) = 10.19 min. Analytical data: ^{1}H NMR (600 MHz, $CDCl_3$, 60 °C) δ 8.80 (br s, 1H), 7.95 (d, *J* = 7.8 Hz, 1H), 7.74 (d, *J* = 7.8 Hz, 2H), 7.57 (d, *J* = 7.8 Hz, 1H), 7.36 (d, *J* = 7.8 Hz, 2H), 5.76 (br s, 1H), 4.81-4.75 (m, 1H), 4.35-4.27 (m, 1H), 4.24-4.17 (m, 1H), 3.99-3.91 (m, 2H), 3.73-3.65 (m, 1H), 3.52 (t, *J* = 9.6 Hz, 1H), 3.12 (dd, *J* = 16.2, 5.4 Hz, 1H), 2.72-2.62 (m, 2H), 2.46 (s, 3H), 2.38-2.30 (m, 1H), 1.33 (br s, 3H), 1.04 (br s, 3H). ^{13}C NMR (150 MHz, $CDCl_3$, 60 °C) δ 193.1, 157.4, 153.6, 145.3, 144.2, 135.7, 133.1, 131.6, 130.6, 130.0, 126.9, 126.7, 117.7, 117.5, 70.1, 63.6, 62.8, 62.5, 46.2, 44.5, 36.3, 21.2, 14.2, 13.8. IR (thin film): ν_{max} (cm^{-1}) = 3347, 2983, 2926, 1747, 1702, 1491, 1336, 1235, 1161, 1091, 912, 815, 728, 664. HRMS (ESI) calcd for $C_{26}H_{32}N_5O_7S$ $[M+NH_4]^+$: 558.2017, Found: 558.2014.

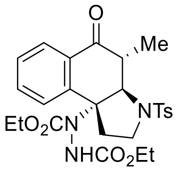

2v, pale yellow oil, 36.0 mg (0.2 mmol scale), 34% yield, 78% ee. $[\alpha]_D^{23}$ +84.4 (*c* 1.0, $CHCl_3$). The enantiomeric excess was determined by Daicel Chiralpak IC (25 cm), Hexanes/^{i}PrOH = 60/40, 1.0 mL/min^{-1}, λ = 254 nm, t_R (major) = 24.05 min, t_R (minor) = 20.87 min. Analytical data: ^{1}H NMR (600 MHz, CD_3CN, 80 °C) δ 8.42 (br s, 1H), 7.88-7.78 (m, 3H), 7.60 (t, *J* = 7.8 Hz, 1H), 7.48 (d, *J* = 7.2 Hz, 2H), 7.38 (t, *J* = 7.2 Hz, 1H), 6.22 (br s, 1H), 4.69 (d, *J* = 11.4 Hz, 1H), 4.33-4.27 (m, 1H), 4.26-4.19 (m, 1H), 3.99-3.85 (m, 2H), 3.75 (dd, *J* = 18.6, 9.6 Hz, 1H), 3.47 (t, *J* = 10.2 Hz, 1H), 2.90-2.83 (m, 1H), 2.77-2.69 (m, 1H), 2.52-2.46 (m, 1H), 2.49 (s, 3H), 1.35 (t, *J* = 6.6 Hz, 3H), 1.30 (dd, *J* = 6.6, 1.2 Hz, 3H), 1.00 (br s, 3H). ^{13}C NMR (150 MHz, CD_3CN, 80 °C) δ 196.0, 156.3, 153.0, 143.5, 143.3, 135.5, 132.7, 130.4, 129.2, 126.2, 126.1, 126.0, 124.6, 70.9, 69.8, 61.2, 61.1, 46.4, 45.4,

35.5, 19.4, 12.7, 12.3, 10.3. IR (thin film): ν_{max} (cm^{-1}) = 3329, 2922, 1747, 1714, 1684, 1506, 1309, 1216, 1090, 822, 760, 672. HRMS (ESI) calcd for $C_{26}H_{35}N_4O_7S$ $[M+NH_4]^+$: 547.2221, Found: 547.2216.

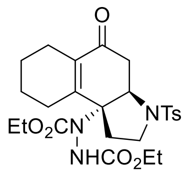

4a, pale yellow oil, 47.0 mg, 90% yield, 96% ee. $[\alpha]_D^{26}$ -84.7 (*c* 2.0, $CHCl_3$). The enantiomeric excess was determined by Daicel Chiralpak IE3 (25 cm), Hexanes/^{i}PrOH = 60/40, 0.7 mL/min^{-1}, λ = 214 nm, t_R (major) = 69.30 min, t_R (minor) = 76.07 min. Analytical data: ^{1}H NMR (600 MHz, $CDCl_3$, 60 °C) δ 7.70 (d, *J* = 8.0 Hz, 2H), 7.31 (d, *J* = 8.0 Hz, 2H), 5.88 (br s, 1H), 4.55 (dd, *J* = 10.0, 6.8 Hz, 1H), 4.23-4.17 (m, 1H), 4.17-4.05 (m, 3H), 3.66-3.60 (m, 1H), 3.45 (t, *J* = 9.2 Hz, 1H), 2.78 (dd, *J* = 16.0, 6.4 Hz, 1H), 2.64-2.50 (m, 2H), 2.42 (s, 3H), 2.40-2.34 (m, 1H), 2.24-2.04 (m, 3H), 1.75-1.42 (m, 5H), 1.26 (t, *J* = 7.2 Hz, 3H), 1.20 (t, *J* = 7.2 Hz, 3H). ^{13}C NMR (150 MHz, $CDCl_3$, 60 °C) δ 194.5, 157.2, 155.1, 154.4, 144.1, 136.4, 132.0, 130.1, 127.0, 72.5, 63.8, 62.8, 62.2, 46.2, 43.1, 34.3, 27.2, 22.8, 22.2, 21.43, 21.40, 14.4, 14.3. IR (thin film): ν_{max} (cm^{-1}) = 3297, 2932, 2862, 1743, 1716, 1664, 1376, 1230, 1160, 1090, 912, 816, 728, 660. HRMS (ESI) calcd for $C_{25}H_{37}N_4O_7S$ $[M+NH_4]^+$: 537.2377, Found: 537.2365.

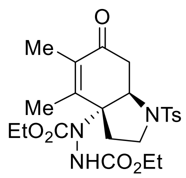

4b, pale yellow oil, 40.0 mg, 82% yield, 53% ee. $[\alpha]_D^{26}$ -28.7 (*c* 2.0, $CHCl_3$). The enantiomeric excess was determined by Daicel Chiralpak IA (25 cm), Hexanes/^{i}PrOH = 70/30, 0.7 mL/min^{-1}, λ = 214 nm, t_R (major) = 9.28 min, t_R (minor) = 10.59 min. Analytical data: ^{1}H NMR (600 MHz, $CDCl_3$, 60 °C) δ 7.72 (d, *J* = 8.0 Hz, 2H), 7.34 (d, *J* = 8.0 Hz, 2H), 5.95 (br s, 1H), 4.54 (dd, *J* = 10.4, 6.4 Hz, 1H), 4.26-4.20 (m, 1H), 4.17-4.09 (m, 3H), 3.70-3.64 (m, 1H), 3.45 (td, *J* = 10.0, 2.8 Hz, 1H), 2.80 (dd, *J* = 16.0, 6.4 Hz, 1H), 2.60-2.55 (m, 1H), 2.44 (s, 3H), 2.43-2.38 (m, 1H), 2.22-2.14 (m, 1H), 2.09 (s, 3H), 1.78 (s, 3H), 1.29 (t, *J* = 7.2 Hz, 3H), 1.21 (t, *J* = 7.2 Hz, 3H). ^{13}C NMR (150 MHz, $CDCl_3$, 60 °C) δ 194.3, 157.3, 154.5, 144.2, 136.4, 131.3, 130.1, 129.9, 127.1, 73.1, 63.7, 62.8, 62.3, 46.3, 42.9, 34.1, 21.4, 16.8, 14.4, 14.2, 11.6. IR (thin film): ν_{max} (cm^{-1}) = 3295, 2982, 2828, 1717, 1666, 1327, 1231, 1160, 1091, 815, 731, 660, 545. HRMS (ESI) calcd for $C_{23}H_{35}N_4O_7S$ $[M+NH_4]^+$: 511.2221, Found: 511.2208.

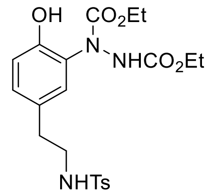

5, light white solid, m.p. = 160-162 °C, 39.0 mg, 84% yield. Analytical data: ^{1}H NMR (600 MHz, $CDCl_3$, 60 °C) δ 8.61 (br s, 1H), 7.68 (d, *J* = 7.8 Hz, 2H), 7.44 (s, 1H), 7.26 (d, *J* = 7.8 Hz, 2H), 6.96-6.89 (m, 2H), 6.87 (d, *J* = 8.4 Hz, 1H), 4.70 (br s, 1H), 4.26 (q, *J* = 7.2 Hz, 2H), 4.20 (q, *J* = 7.2 Hz, 2H), 3.16 (q, *J* = 6.6 Hz, 2H), 2.67 (t, *J* = 6.6 Hz, 2H), 2.41 (s, 3H), 1.29 (t, *J* = 7.2 Hz, 3H), 1.21 (t, *J* = 6.6 Hz, 3H). ^{13}C NMR (150 MHz, $CDCl_3$, 60 °C) δ 159.0, 155.3, 152.4, 143.1, 137.2, 130.4, 129.4, 129.3, 128.8, 128.0, 126.9, 118.2, 63.2, 63.1, 44.0, 34.6, 21.1, 14.1, 14.0. IR (thin film): ν_{max}

(cm^{-1}) = 3268, 2983, 2933, 1717, 1695, 1521, 1317, 1264, 1154, 1060, 822, 759, 661. HRMS (ESI) calcd for $C_{21}H_{31}N_4O_7S$ $[M+NH_4]^+$: 483.1908, Found: 483.1898.

Procedure for the preparation of **6**

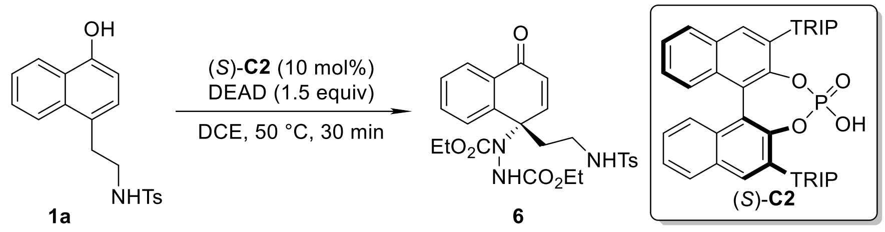

To a flask containing a mixture of **1a** (34 mg, 0.1 mmol) and (*S*)-**C2** (7.5 mg, 0.01 mmol) under argon was added a solution of DEAD (26.0 mg, 0.15 mmol) in anhydrous 1,2-dichloroethane (2 mL). The reaction was stirred at 50 °C for 30 min. The reaction mixture was cooled to room teperature, quenched with $NaHCO_3$ (aq., 10 mL) and extracted with CH_2Cl_2 (3 × 15 mL). The combined organic layer was washed with brine, separated, dried over Na_2SO_4 and filtrated. After the solvent was removed under reduced pressure, the residue was purified by silica gel column chromatography (ethyl acetate/petroleum ether = 1/3 to 1/1) to afford **6**.

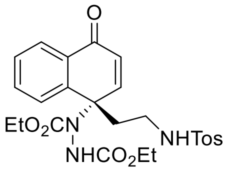

6, pale yellow oil, 45.0 mg, 86% yield, >99% ee. $[\alpha]_D^{20}$ -14.3 (*c* 2.0, $CHCl_3$). The enantiomeric excess was determined by Daicel Chiralpak IC (25 cm), Hexanes/^{i}PrOH = 50/50, 1.0 mL/min^{-1}, λ = 254 nm, t_R (major) = 16.33 min, t_R (minor) = 9.55 min. Analytical data: ^{1}H NMR (600 MHz, CD_3CN, 80 °C, two rotamers) δ 8.14, 7.80 (br s, 1H), 8.02 (t, *J* = 6.6 Hz, 1H), 7.66-7.60 (m, 1H), 7.51 (d, *J* =

7.8 Hz, 2H), 7.48-7.43 (m, 1H), 7.31 (d, *J* = 7.8 Hz, 2H), 7.19, 6.86 (d, *J* = 10.2 Hz, 1H), 6.42-6.36 (m, 1H), 5.21 (br s, 1H), 4.30-4.22 (m, 2H), 3.89-3.83 (m, 2H), 2.68-2.61 (m, 1H), 2.43 (s, 3H), 2.37-2.06 (m, 4H), 1.37-1.31 (m, 3H), 0.95-0.89 (m, 3H). ^{13}C NMR (150 MHz, CD_3CN, 80 °C, two rotamers) δ 182.64, 182.61, 156.21, 153.33, 153.12, 149.14, 147.04, 144.35, 142.35, 136.17, 136.12, 131.92, 131.88, 130.43, 130.20, 128.41, 127.76, 127.25, 126.42, 126.27, 125.53, 125.17, 124.70, 124.64, 124.55, 124.48, 123.80, 61.76, 61.67, 60.96, 60.85, 60.84, 60.81, 37.31, 37.20, 37.17, 37.14, 19.26, 12.69, 12.12, 12.08. IR (thin film): ν_{max} (cm^{-1}) = 3274, 2982, 2932, 1717, 1660, 1598, 1376, 1238, 1155, 1092, 749, 662. HRMS (ESI) calcd for $C_{25}H_{33}N_4O_7S$ $[M+NH_4]^+$: 533.2064, Found: 533.2060.

Procedure for the preparation of **7**

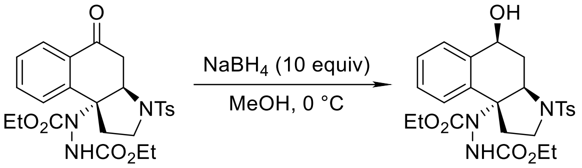

2a **7**

To a flask containing a solution of **2a** (103 mg, 0.2 mmol, 99% ee) in anhydrous MeOH (4 mL) at 0 °C was added $NaBH_4$ (76 mg, 2.0 mmol). The mixture was stirred at 0 °C until TLC showed complete consumption of starting material. Water was added and the mixture was extracted with AcOEt (3 × 20 mL). The combined organic layer was washed with brine, separated, dried over Na_2SO_4 and filtered. After the solvent was removed under reduced pressure, the residue was purified by silica gel column chromatography (ethyl acetate/petroleum ether = 1/4 to 1/2) to afford **7**.

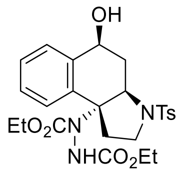

7, colorless oil, 94.0 mg, 91% yield, 98% ee. $[\alpha]_D^{19}$ -76.0 (*c* 4.5, $CHCl_3$). The enantiomeric excess was determined by Daicel Chiralpak IC (25 cm), Hexanes/^{i}PrOH = 50/50, 0.9 mL/min^{-1}, λ = 254 nm, t_R (major) = 24.16 min, t_R (minor) = 16.76 min. Analytical data: ^{1}H NMR (600 MHz, $CDCl_3$, 60 °C) δ 7.95 (br s, 1H), 7.78 (d, *J* = 8.4 Hz, 2H), 7.52-7.48 (m, 1H), 7.36 (d, *J* = 8.4 Hz, 2H), 7.22-7.16 (m, 2H), 5.04 (br s, 1H), 4.92 (dd, *J* = 11.4, 3.6 Hz, 1H), 4.46 (dd, *J* = 12.6, 4.8 Hz, 1H), 4.26-4.20 (m, 1H), 4.12 (br s, 1H), 3.92-3.85 (m, 2H), 3.48 (dt, *J* = 9.6, 4.8 Hz, 1H), 3.41 (dd, *J* = 18.0, 9.6 Hz, 1H), 2.57 (dt, *J* = 12.0, 4.8 Hz, 1H), 2.49 (dd, *J* = 14.4, 7.8 Hz, 1H), 2.45 (s, 3H), 2.22 (dt, *J* = 14.4, 10.2 Hz, 1H), 1.78 (q, *J* = 12.0 Hz, 1H), 1.29 (t, *J* = 7.2 Hz, 3H), 0.96 (br s, 3H). ^{13}C NMR (150 MHz, $CDCl_3$, 60 °C) δ 157.5, 153.9, 143.9, 138.8, 138.2, 135.6, 130.1, 127.8, 127.1, 127.0, 126.8, 123.9, 71.7, 67.2, 64.0, 62.3, 62.1, 46.4, 41.8, 37.4, 21.3, 14.3, 13.9. IR (thin film): ν_{max} (cm^{-1}) = 3511, 3348, 2982, 2903, 1754, 1713, 1491, 1336, 1224, 1161, 1092, 912, 763, 728, 666; HRMS (ESI) calcd for $C_{25}H_{32}N_3O_7S$ $[M+H]^+$: 518.1955, Found: 518.1948.

Procedure for the preparation of **8**

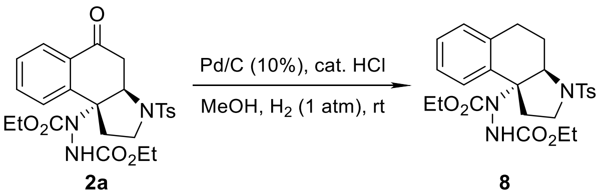

To a flask containing a solution of **2a** (103 mg, 0.2 mmol, 99% ee) in MeOH (4 mL) were added 10% Pd/C (50 mg) and HCl (conc. 20 μL). Then the mixture was stirred under H_2 (1 atm) at room temperature until TLC showed complete consumption of starting material. After the removal of Pd/C,

water was added and the mixture was extracted with AcOEt (3 × 20 mL). The combined organic layer was washed with brine, separated, dried over Na_2SO_4 and filtered. After the solvent was removed under reduced pressure, the residue was purified by silica gel column chromatography (ethyl acetate/petroleum ether = 1/10 to 1/5) to afford **8**.

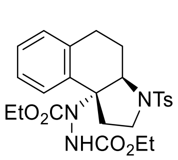

8, colorless oil, 78.0 mg, 78% yield, >99% ee. $[\alpha]_D^{19}$ -57.8 (*c* 2.0, $CHCl_3$). The enantiomeric excess was determined by Daicel Chiralpak AD-H (25 cm), Hexanes/^{i}PrOH = 80/20, 1.0 mL/min^{-1}, λ = 254 nm, t_R (major) = 8.46 min, t_R (minor) = 16.01 min. Analytical data: ^{1}H NMR (600 MHz, $CDCl_3$, 60 °C) δ 7.94 (br s, 1H), 7.78 (d, *J* = 8.4 Hz, 2H), 7.36 (d, *J* = 8.4 Hz, 2H), 7.12 (t, *J* = 7.2 Hz, 1H), 7.06 (t, *J* = 7.2 Hz, 1H), 6.99 (d, *J* = 7.2 Hz, 1H), 4.95 (br s, 1H), 4.34 (dd, *J* = 12.0, 4.8 Hz, 1H), 4.26-4.20 (m, 1H), 4.16-4.08 (m, 1H), 3.95-3.84 (m, 2H), 3.46 (t, *J* = 9.0 Hz, 1H), 3.38 (q, 9.6 Hz, 1H), 3.00-2.91 (m, 1H), 2.69 (dt, *J* = 16.2, 3.6 Hz, 1H), 2.54-2.44 (m, 1H), 2.45 (s, 3H), 2.34-2.27 (m, 1H), 2.20 (dt, *J* = 14.4, 9.6 Hz, 1H), 1.76 (td, *J* = 13.2, 4.2 Hz, 1H), 1.28 (t, *J* = 7.2 Hz, 3H), 0.96 (br s, 3H). ^{13}C NMR (150 MHz, $CDCl_3$, 60 °C) δ 157.4, 153.8, 143.6, 139.7, 135.7, 135.6, 129.9, 127.2, 126.9, 126.8, 126.5, 71.6, 65.5, 62.0, 61.9, 46.0, 37.5, 31.9, 28.2, 21.2, 14.2, 13.7. IR (thin film): ν_{max} (cm^{-1}) = 3348, 2981, 2902, 1756, 1715, 1492, 1337, 1223, 1161, 1092, 911, 761, 728, 663. HRMS (ESI) calcd for $C_{25}H_{35}N_4O_6S$ [M+H]$^+$: 519.2272, Found: 519.2260.

Procedure for the preparation of **9**[12]

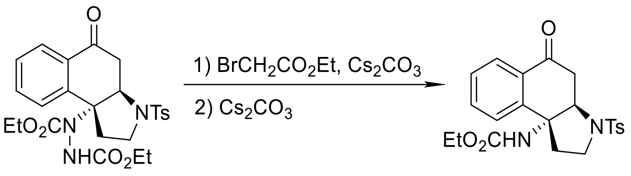

To a flask containing a mixture of **2a** (103 mg, 0.2 mmol, 99% ee) and Cs_2CO_3 (84 mg, 0.26 mmol) was added a solution of $BrCH_2CO_2Et$ (37 mg, 0.22 mmol) in anhydrous CH_3CN (4 mL). The mixture was stirred at 50 °C until TLC showed complete consumption of starting material. Water was added and the mixture was extracted with AcOEt (3 × 20 mL). The combined organic layer was washed with brine, separated, dried over Na_2SO_4 and filtered. After the solvent was removed under reduced pressure, the residue was redissovled in anhydrous CH_3CN (4 mL), and then Cs_2CO_3 (84 mg, 0.26 mmol) was added. The mixture was stirred at 50 °C until TLC showed complete consumption of starting material. Water was added and the mixture was extracted with AcOEt (3 × 20 mL). The combined organic layer was washed with brine, separated, dried over Na_2SO_4 and filtered. After the solvent was removed under reduced pressure, the residue was purified by silica gel column chromatography (ethyl acetate/petroleum ether = 1/3 to 1/2) to afford **9**.

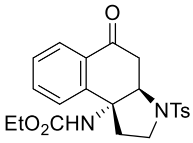

9, pale yellow oil, 45.0 mg, 52% yield, 98% ee. $[\alpha]_D^{20}$ -155.6 (*c* 2.0, $CHCl_3$). The enantiomeric excess was determined by Daicel Chiralpak AD-H (25 cm), Hexanes/^{i}PrOH = 80/20, 1.0 mL/min^{-1}, λ = 254 nm, t_R (major) = 7.56 min, t_R (minor) = 14.73 min. Analytical data: ^{1}H NMR (600 MHz, CD_3CN, 80 °C) δ 7.85 (d, *J* = 8.0 Hz, 1H), 7.75 (d, *J* = 8.0 Hz, 2H), 7.61 (t, *J* = 7.6 Hz, 1H), 7.43 (d, *J* = 8.0 Hz, 1H), 7.42-7.37 (m, 3H), 5.79 (br s, 1H), 4.74 (dd, *J* = 10.8, 6.0 Hz, 1H), 3.84-3.78 (m,

1H), 3.71-3.63 (m, 2H), 3.54-3.49 (m, 1H), 3.14 (dd, *J* = 15.6, 6.0 Hz, 1H), 2.97 (dd, *J* = 15.6, 10.8 Hz, 1H), 2.47-2.41 (m, 1H), 2.45 (s, 3H), 2.30 (dd, *J* = 13.2, 6.0 Hz, 1H), 1.02 (br s, 3H). ^{13}C NMR (150 MHz, CD_3CN, 80 °C) δ 194.5, 154.3, 143.8, 143.2, 134.5, 133.7, 131.5, 129.1, 127.0, 126.8, 125.8, 124.8, 62.2, 61.8, 59.7, 45.9, 44.5, 36.4, 19.9, 13.1. IR (thin film): ν_{max} (cm^{-1}) = 3386, 2920, 2851, 1714, 1690, 1600, 1512, 1309, 1247, 1156, 1092, 822, 779, 669. HRMS (ESI) calcd for $C_{22}H_{28}N_3O_5S$ $[M+NH_4]^+$: 446.1744, Found: 446.1736.

Copies of NMR spectra

Supplementary Figure 1 ^{1}H NMR of compound **1k** ($CDCl_3$, 400 MHz, room temperature)

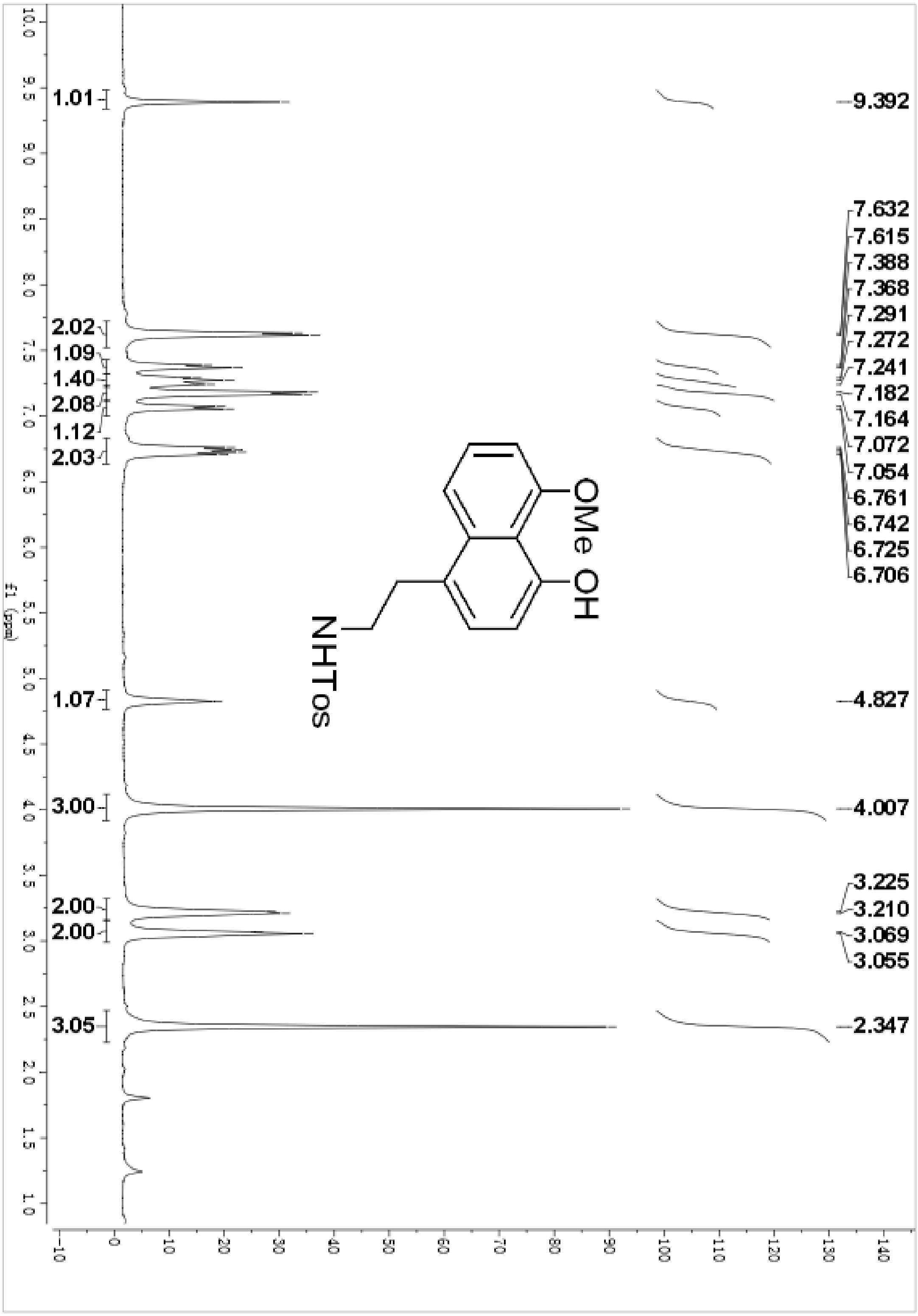

Supplementary Figure 2 ^{13}C NMR of compound **1k** ($CDCl_3$, 100 MHz, room temperature)

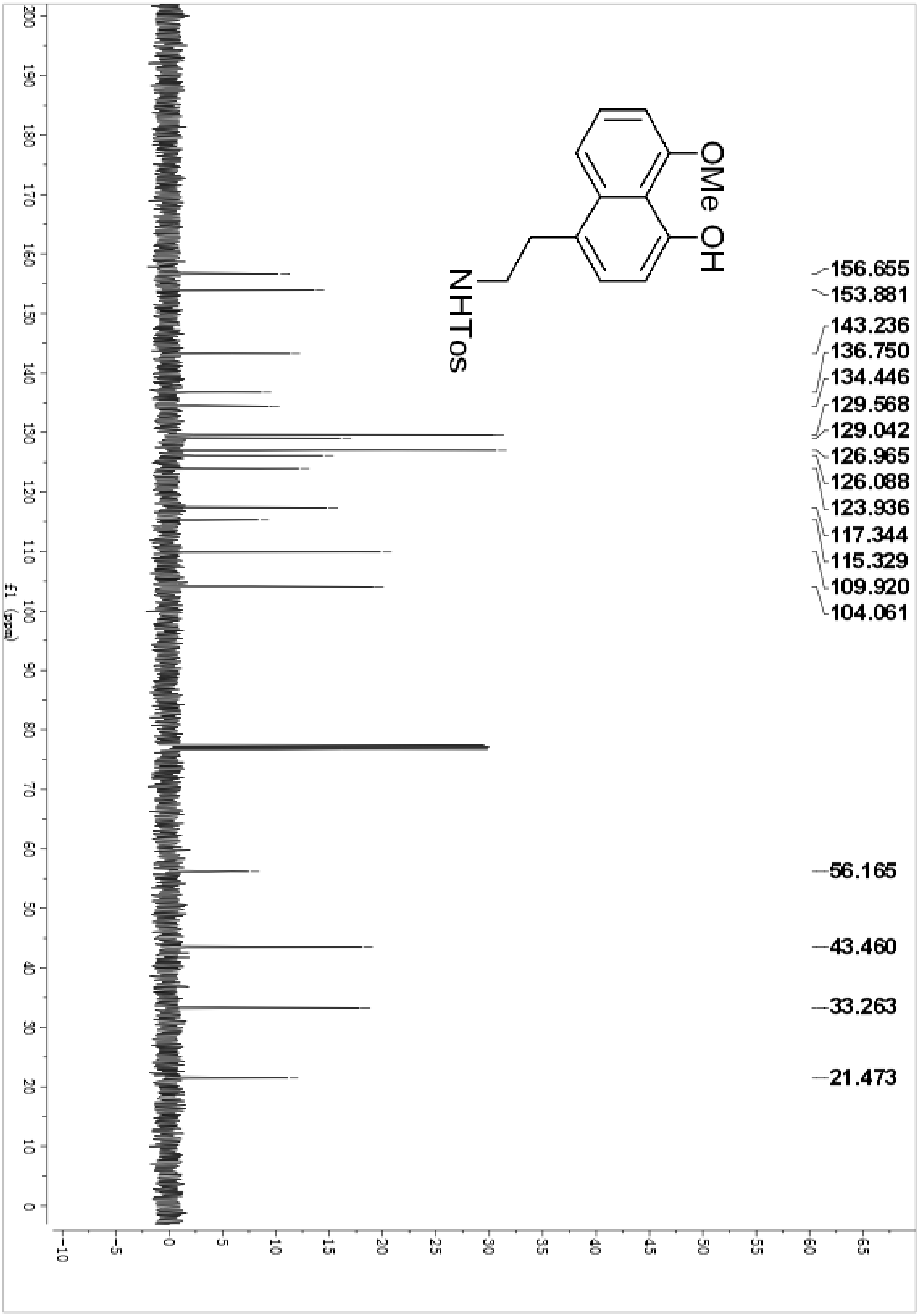

Supplementary Figure 3 ^{1}H NMR of compound **1l** (d_6-Acetone, 400 MHz, room temperature)

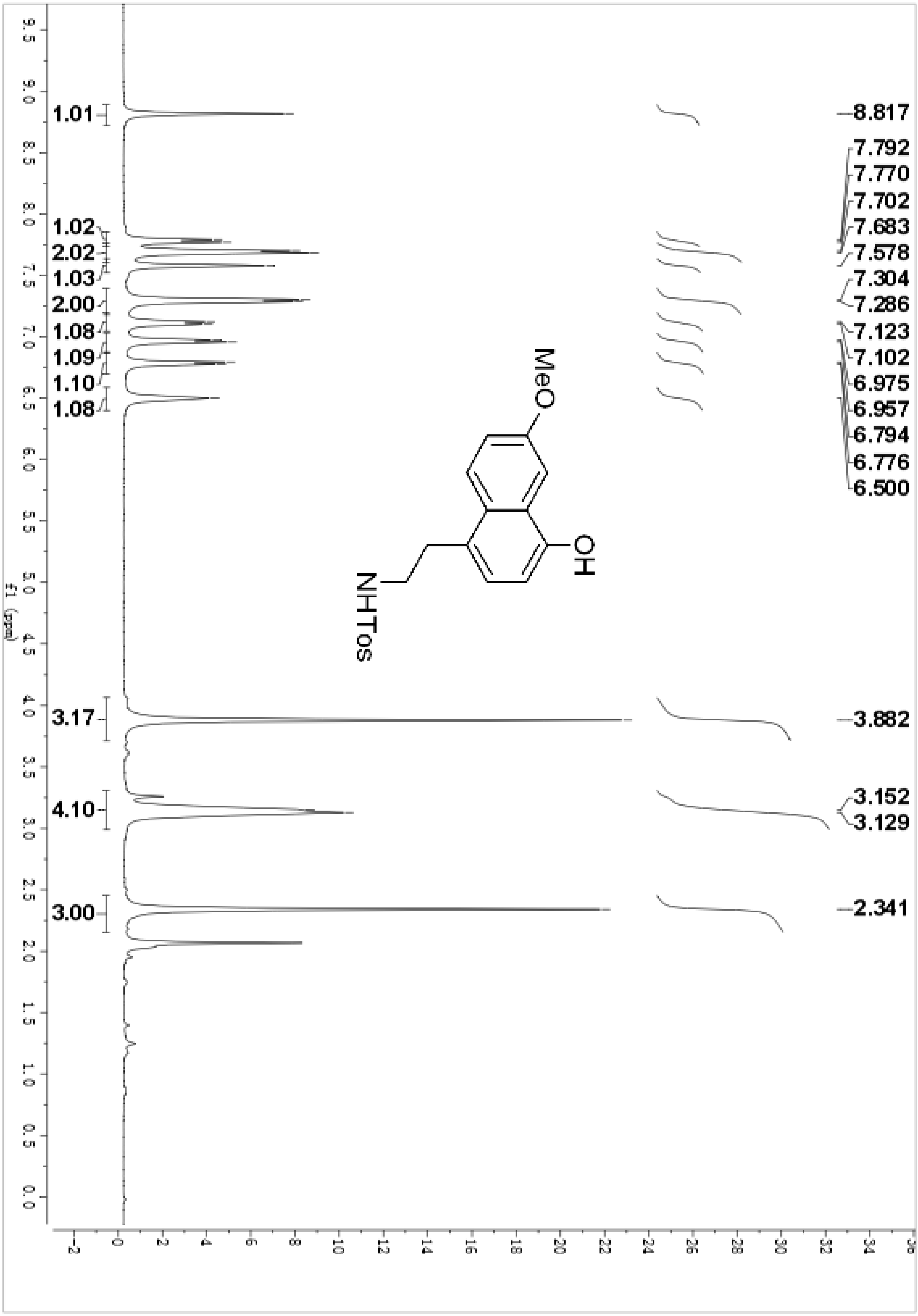

Supplementary Figure 4 ^{13}C NMR of compound **1l** (d_6-Acetone, 100 MHz, room temperature)

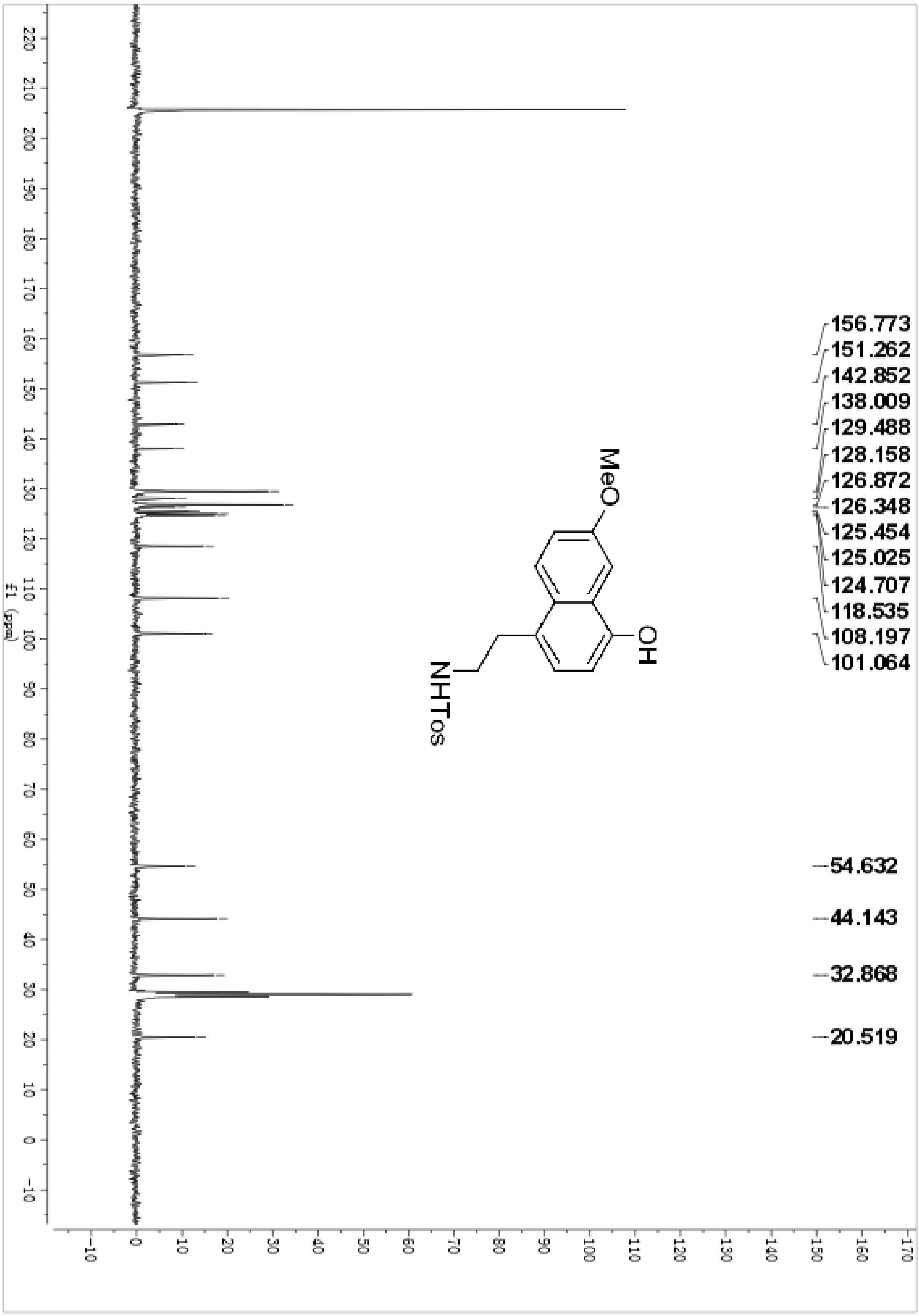

Supplementary Figure 5 ^{1}H NMR of compound **1m** (d_6-Acetone, 400 MHz, room temperature)

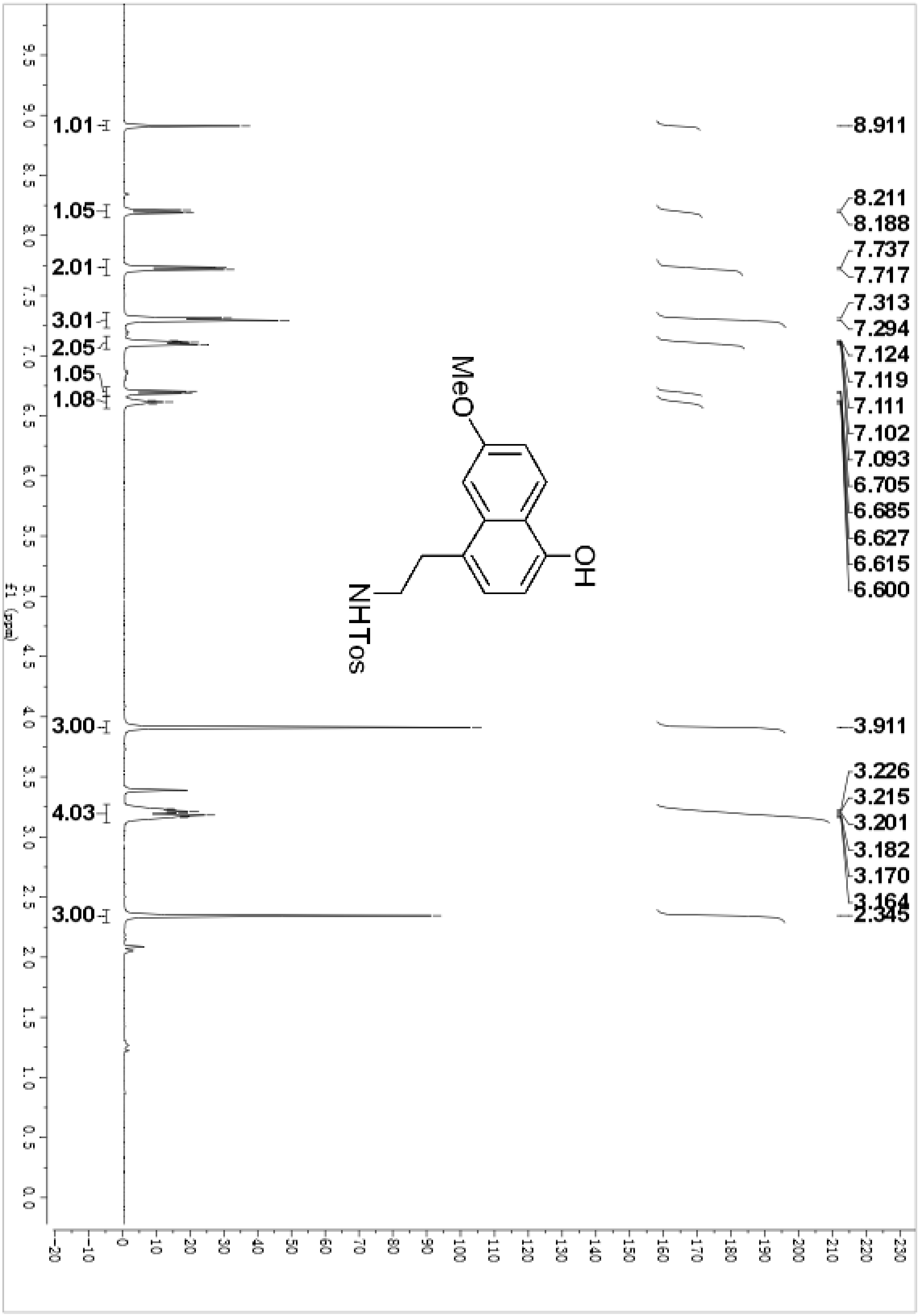

Supplementary Figure 6 ^{13}C NMR of compound **1m** (d_6-Acetone, 100 MHz, room temperature)

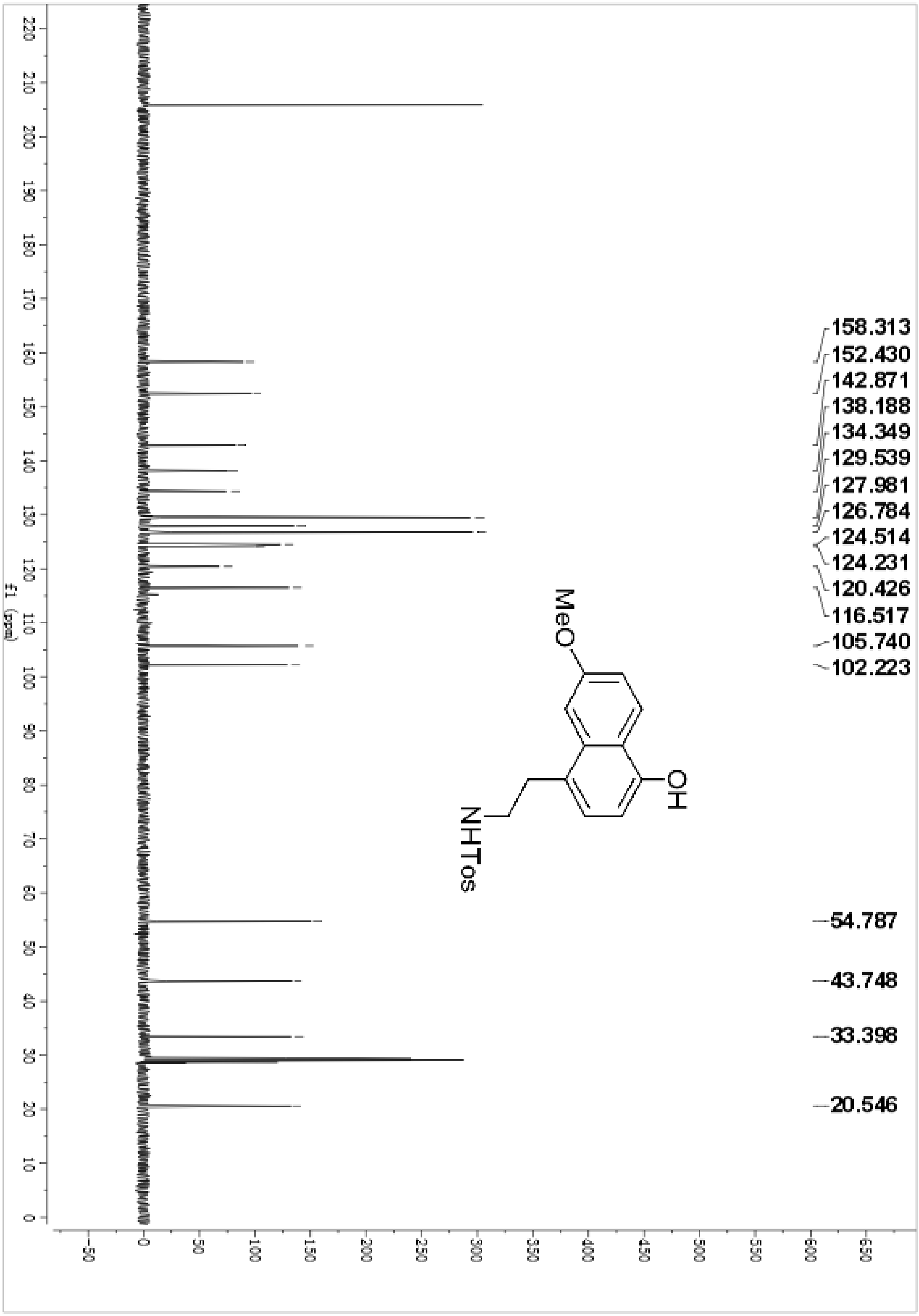

Supplementary Figure 7 ^{1}H NMR of compound **1n** (d_6-Acetone, 400 MHz, room temperature)

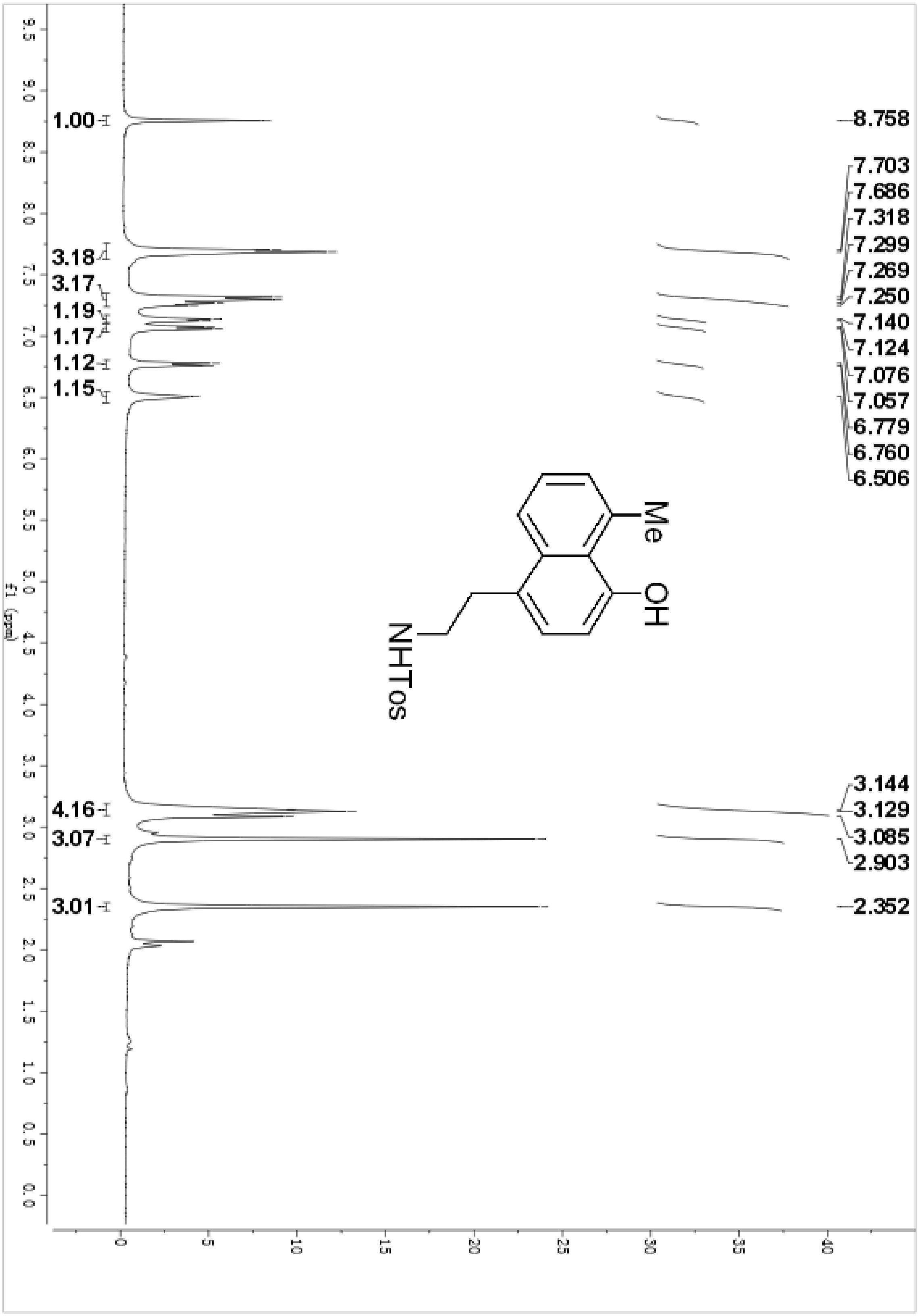

Supplementary Figure 8 ^{13}C NMR of compound **1n** (d_6-Acetone, 100 MHz, room temperature)

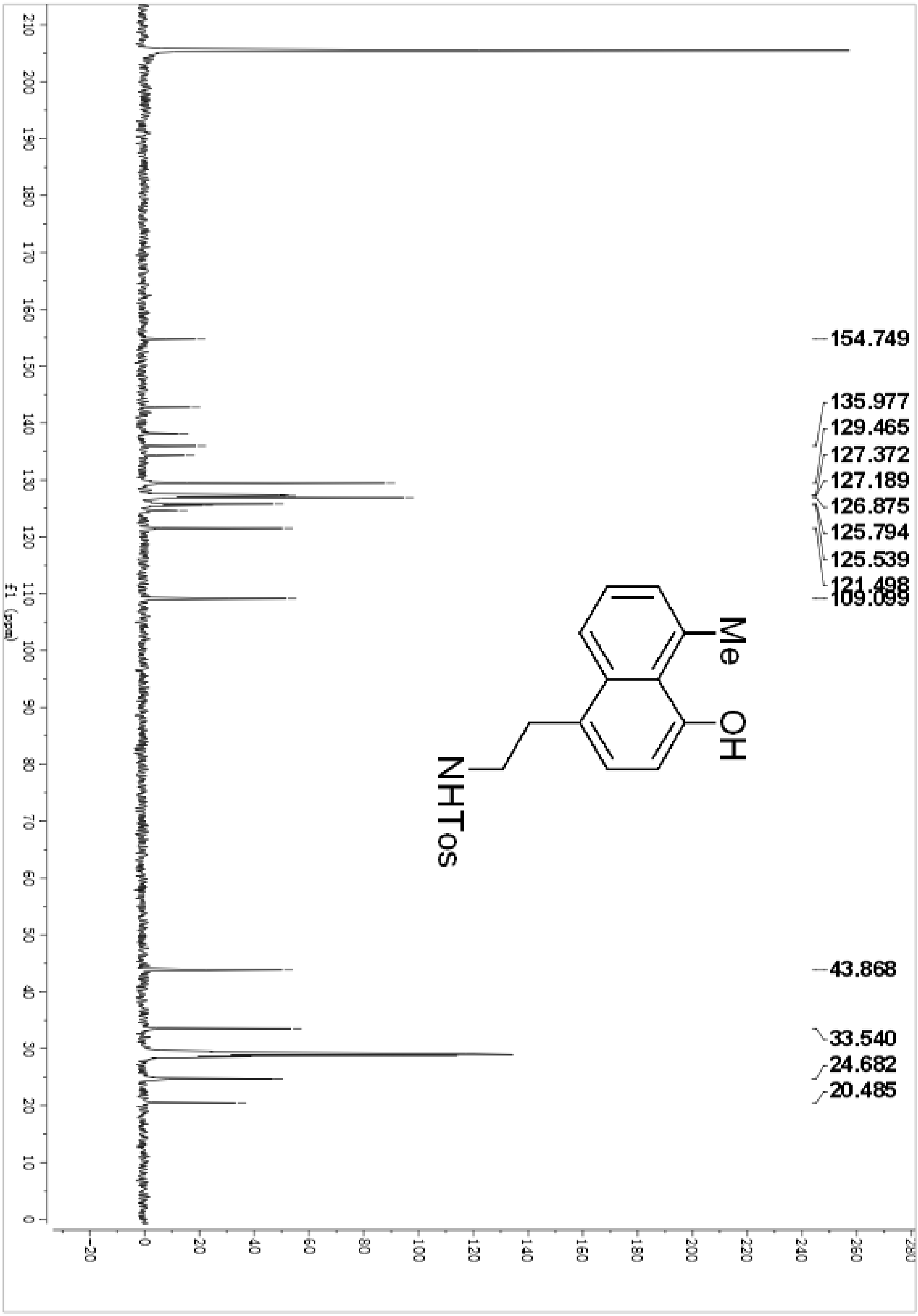

Supplementary Figure 9 ^{1}H NMR of compound **1o** (d_6-Acetone, 400 MHz, room temperature)

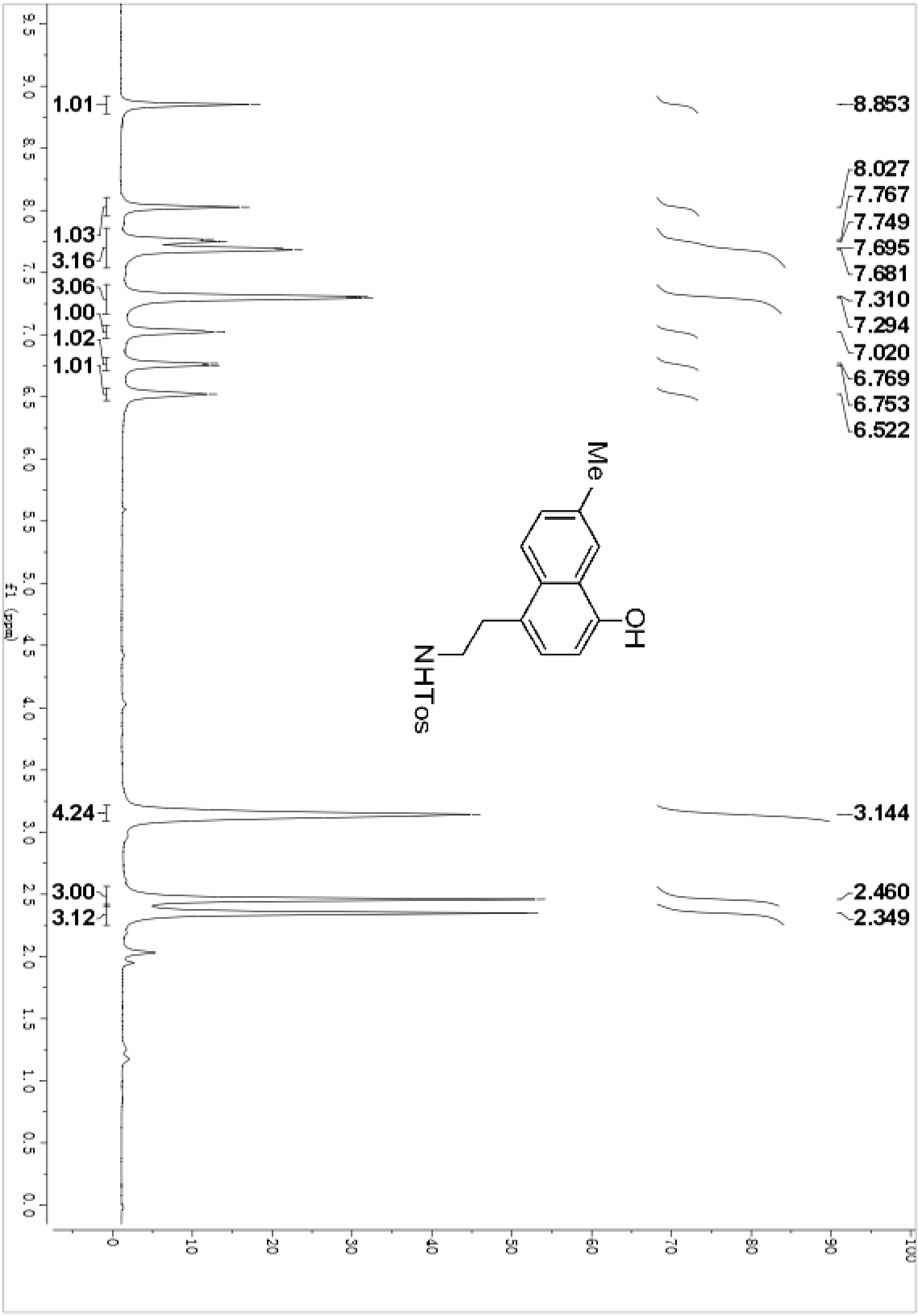

Supplementary Figure 10 ^{13}C NMR of compound **1o** (d_6-Acetone, 100 MHz, room temperature)

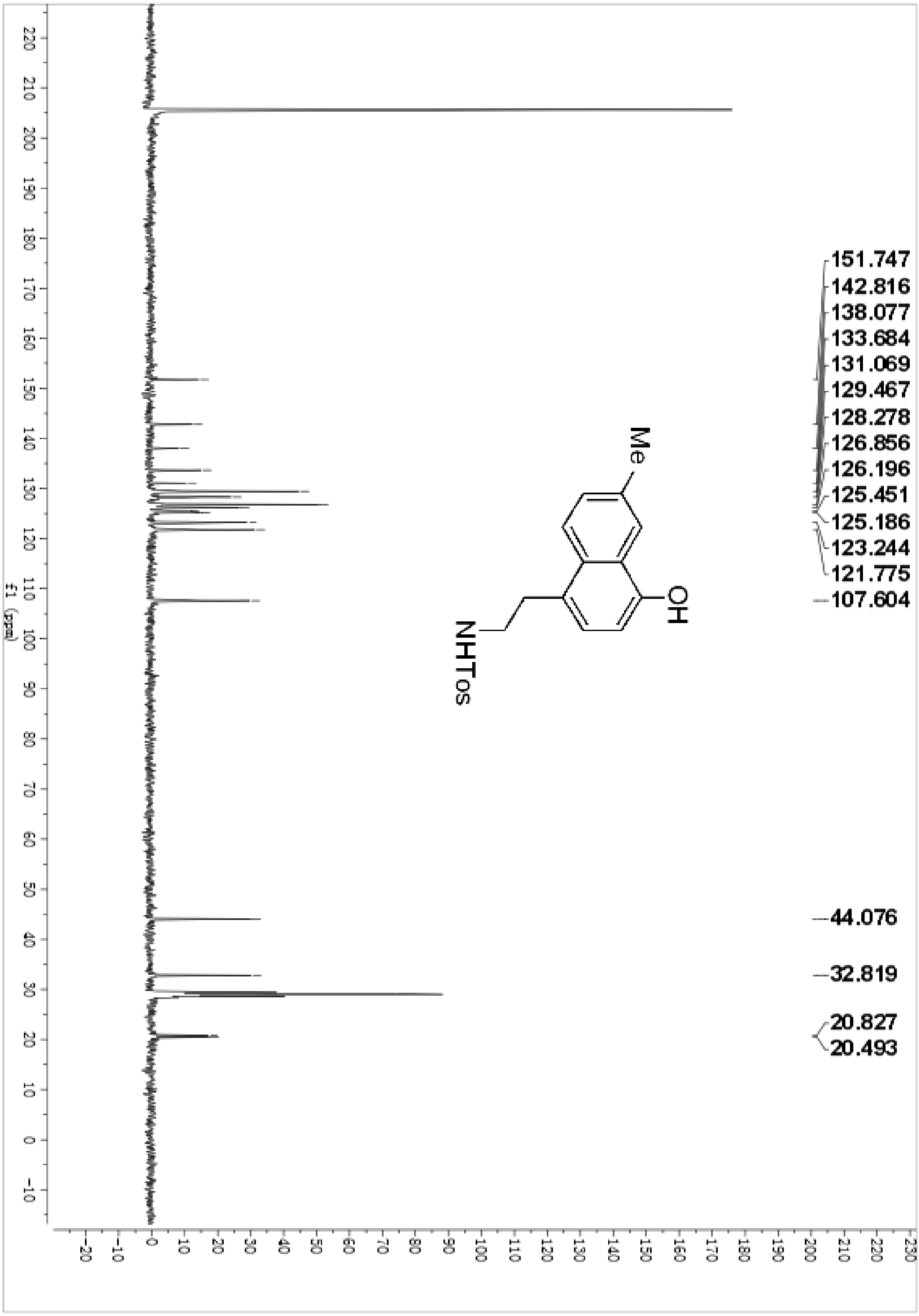

Supplementary Figure 11 ^{1}H NMR of compound **1p** (d_6-Acetone, 400 MHz, room temperature)

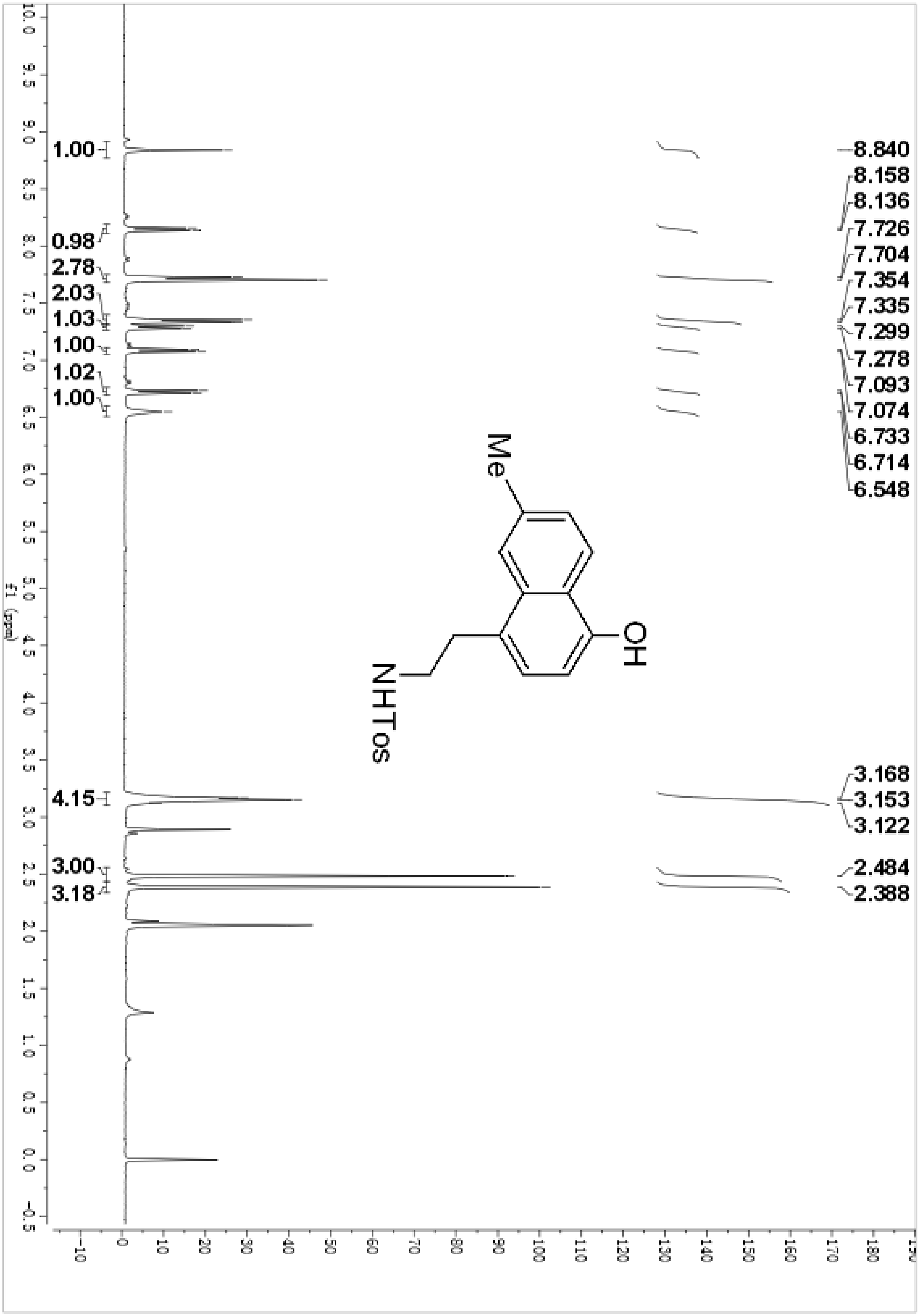

Supplementary Figure 12 ^{13}C NMR of compound **1p** (d_6-Acetone, 100 MHz, room temperature)

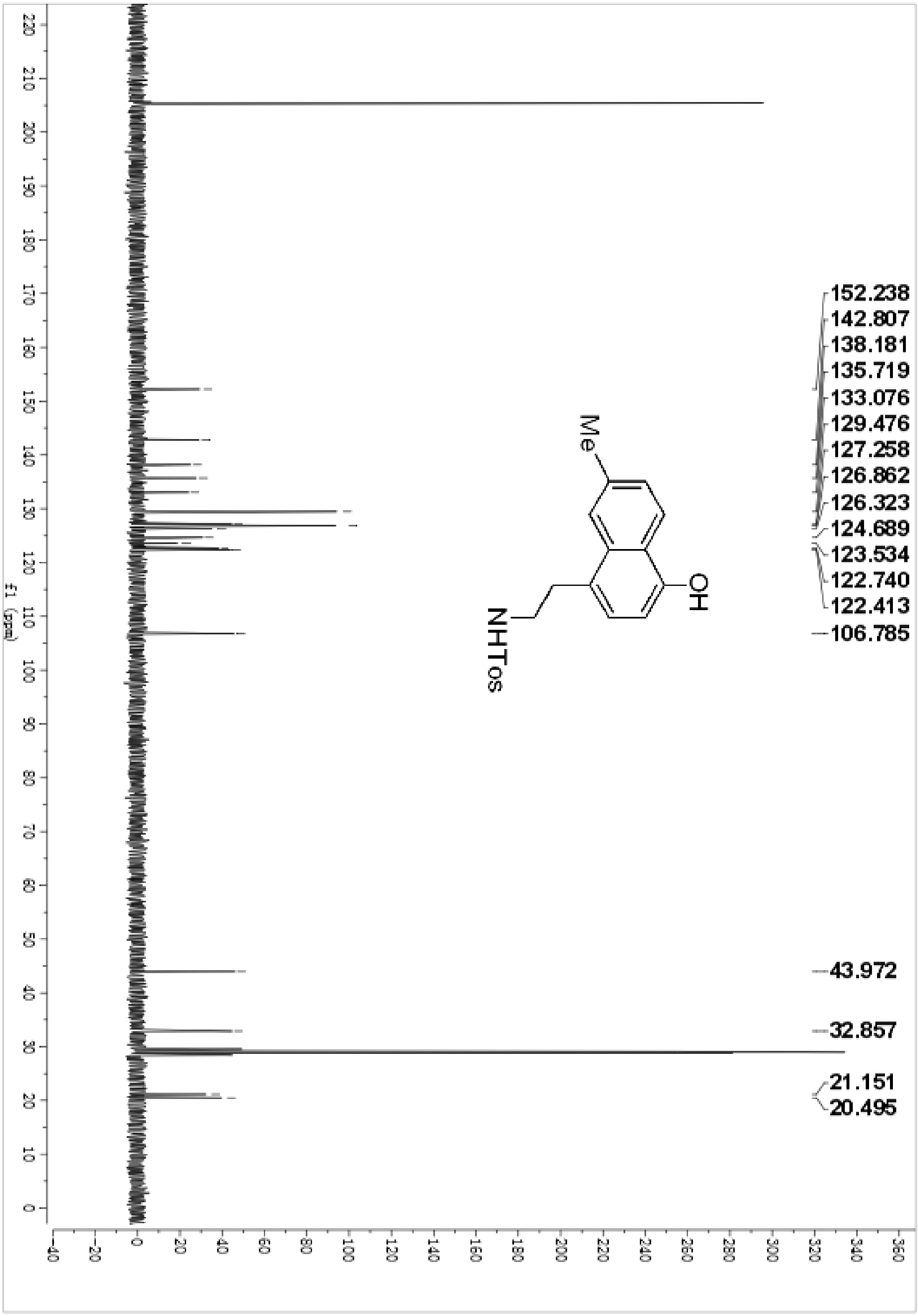

Supplementary Figure 13 ^{1}H NMR of compound **1q**(d_6-Acetone, 400 MHz, room temperature)

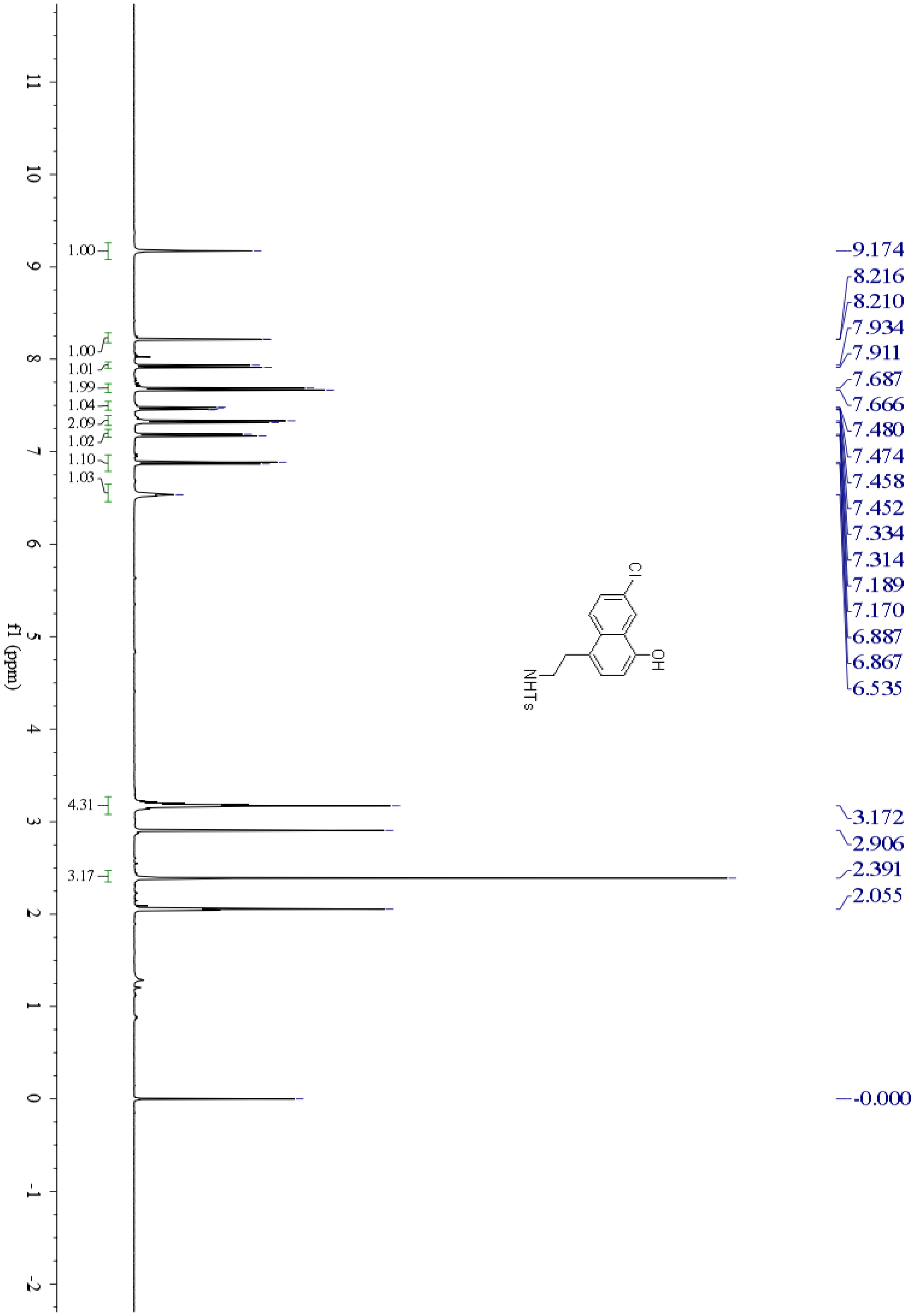

Supplementary Figure 14 ^{13}C NMR of compound **1q** (d_6-Acetone, 100 MHz, room temperature)

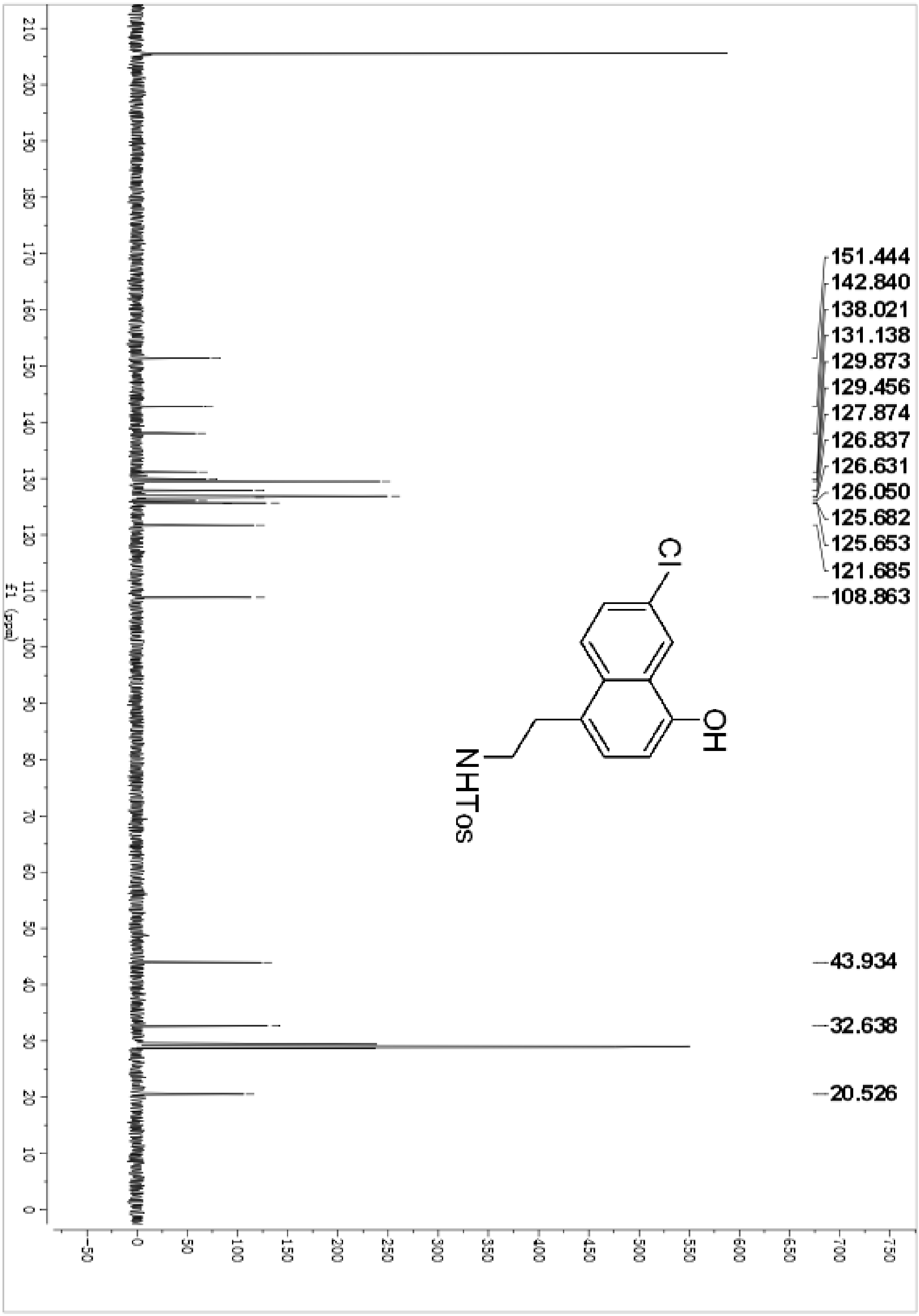

Supplementary Figure 15 ^{1}H NMR of compound **1r** (d_6-Acetone, 400 MHz, room temperature)

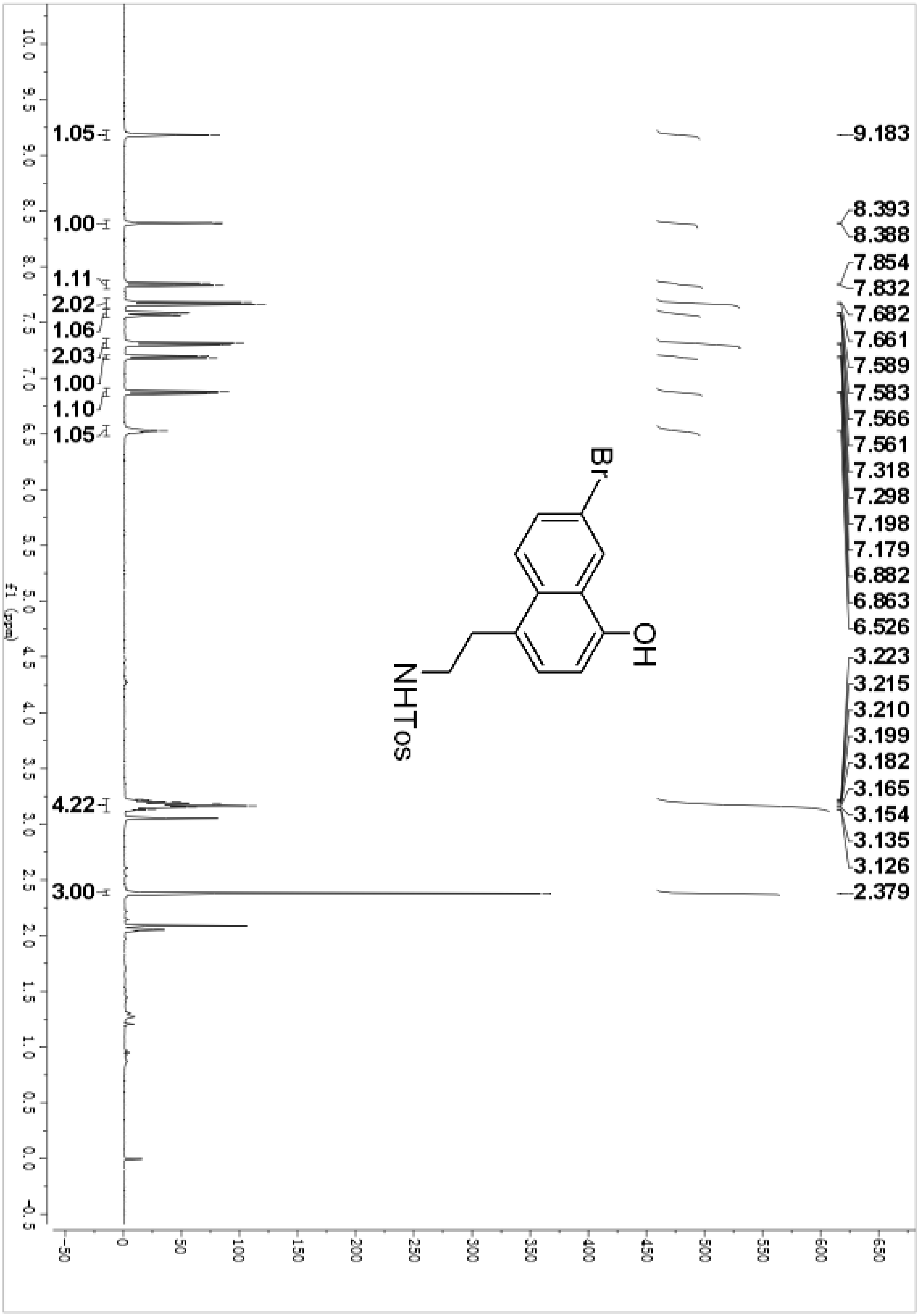

Supplementary Figure 16 ^{13}C NMR of compound **1r** (d_6-Acetone, 100 MHz, room temperature)

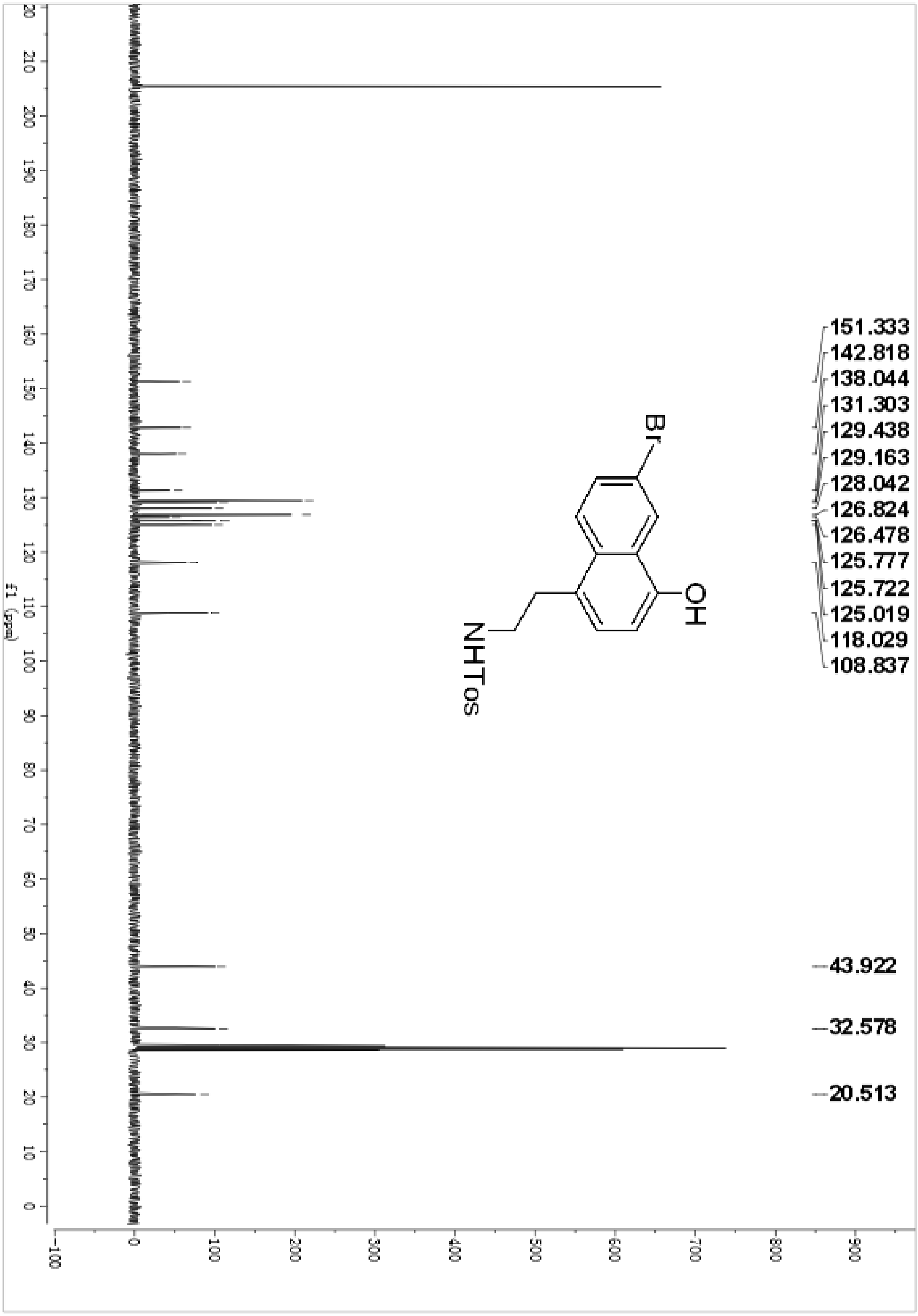

Supplementary Figure 17 ^{1}H NMR of compound **1s** (d_6-Acetone, 400 MHz, room temperature)

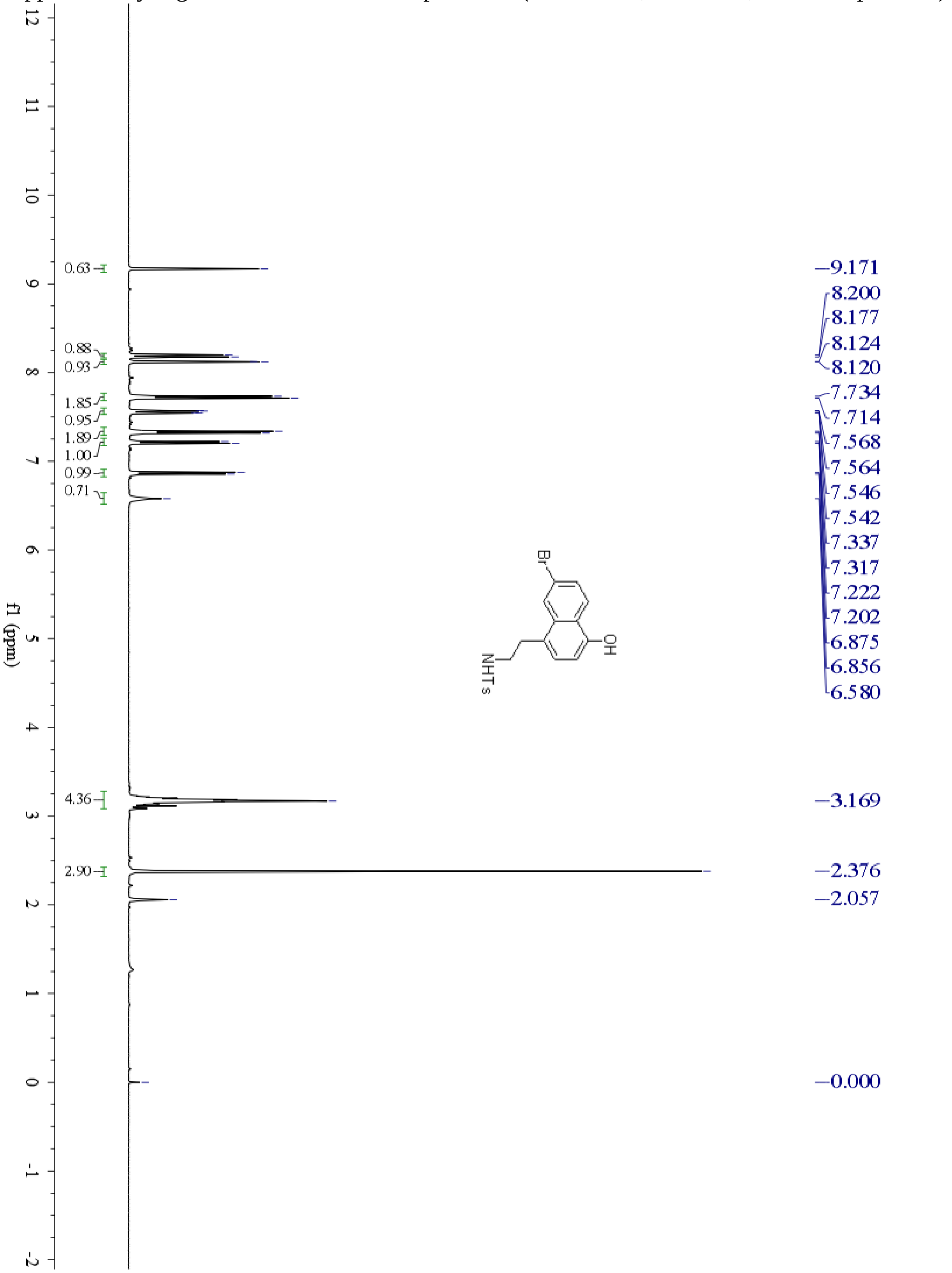

Supplementary Figure 18 ^{13}C NMR of compound **1s** (d_6-Acetone, 100 MHz, room temperature)

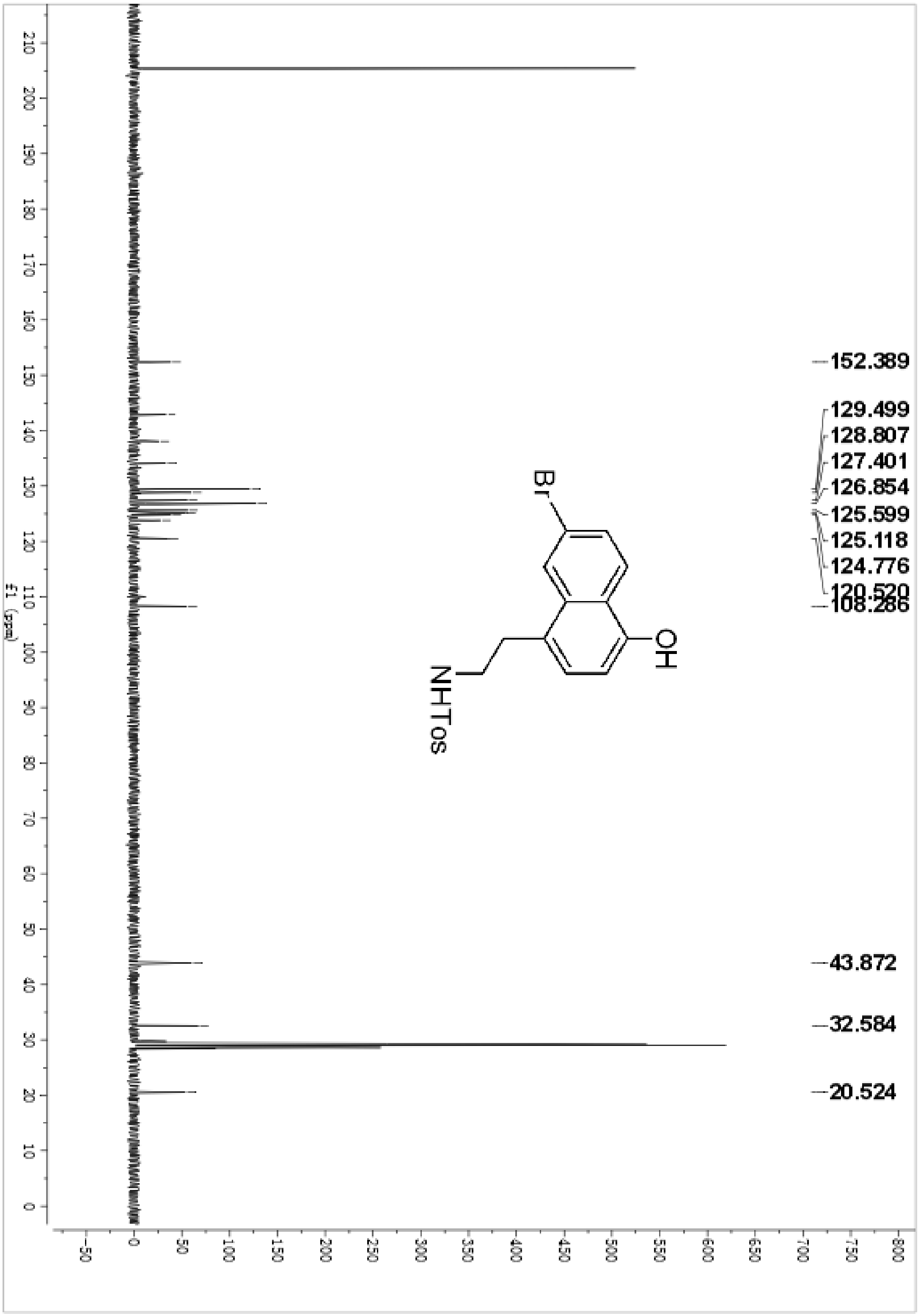

Supplementary Figure 19 ^{1}H NMR of compound **1t** (d_6-Acetone, 400 MHz, room temperature)

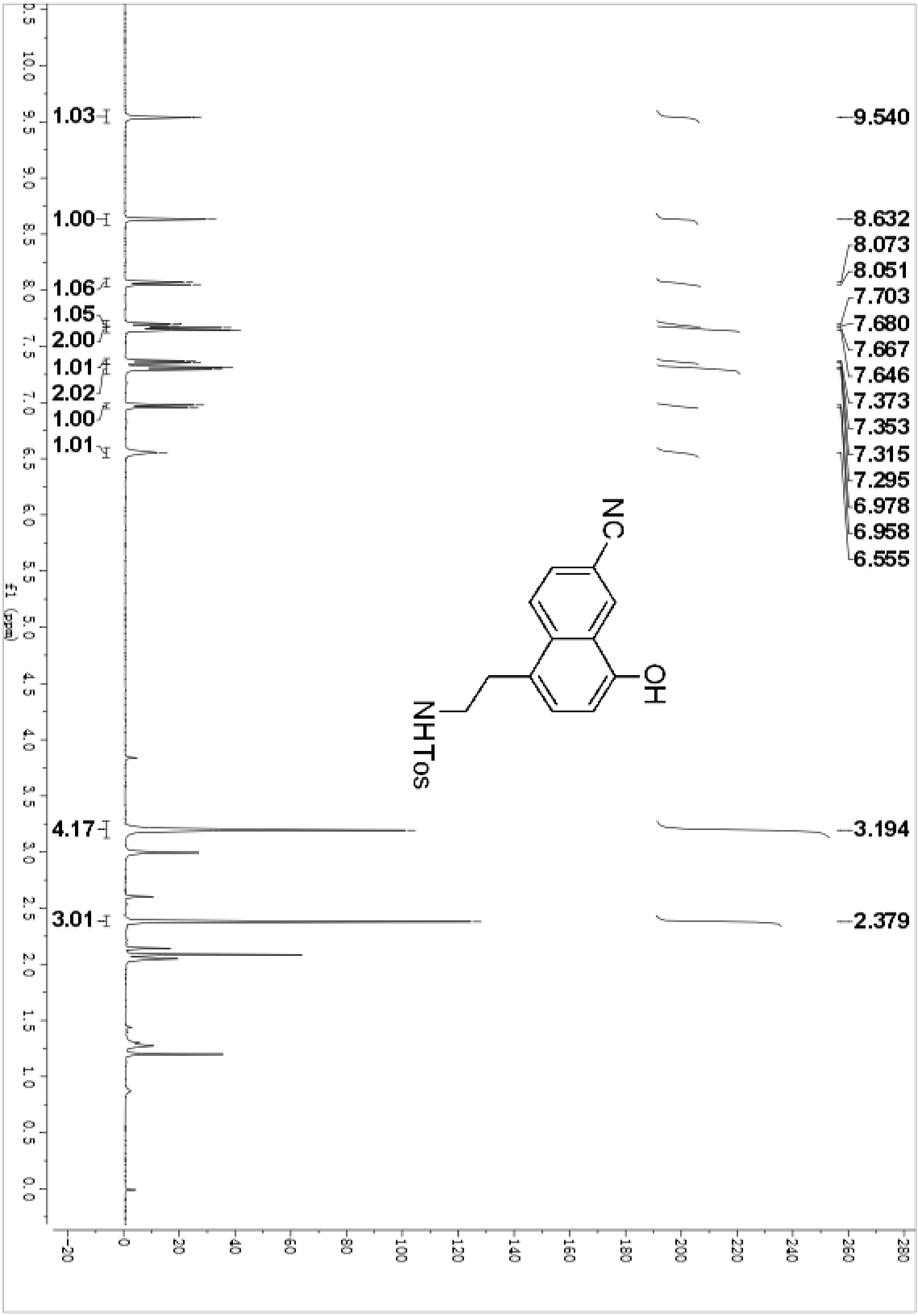

Supplementary Figure 20 ^{13}C NMR of compound **1t** (d_6-Acetone, 100 MHz, room temperature)

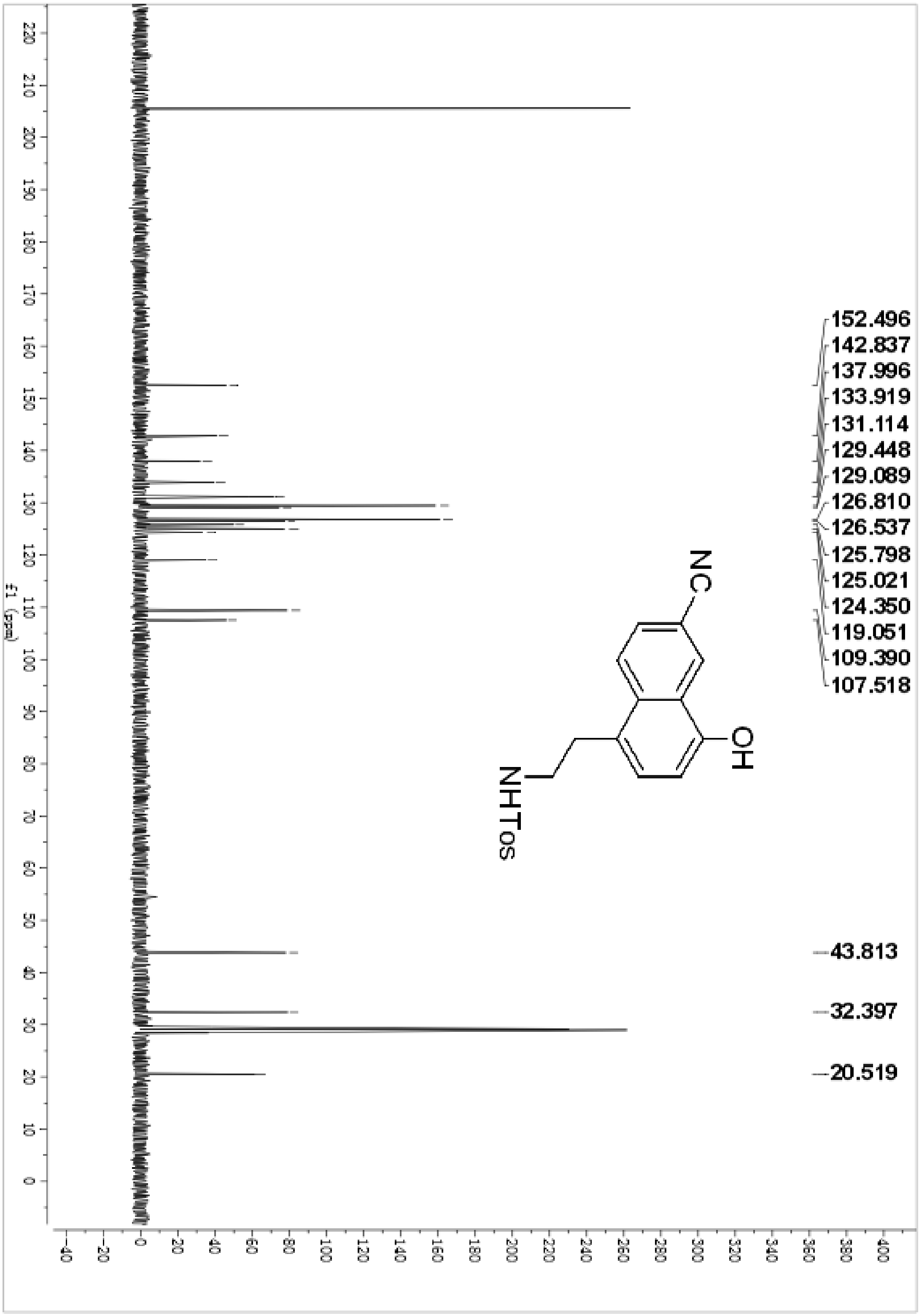

Supplementary Figure 21 ^{1}H NMR of compound **1u** (d_6-Acetone, 400 MHz, room temperature)

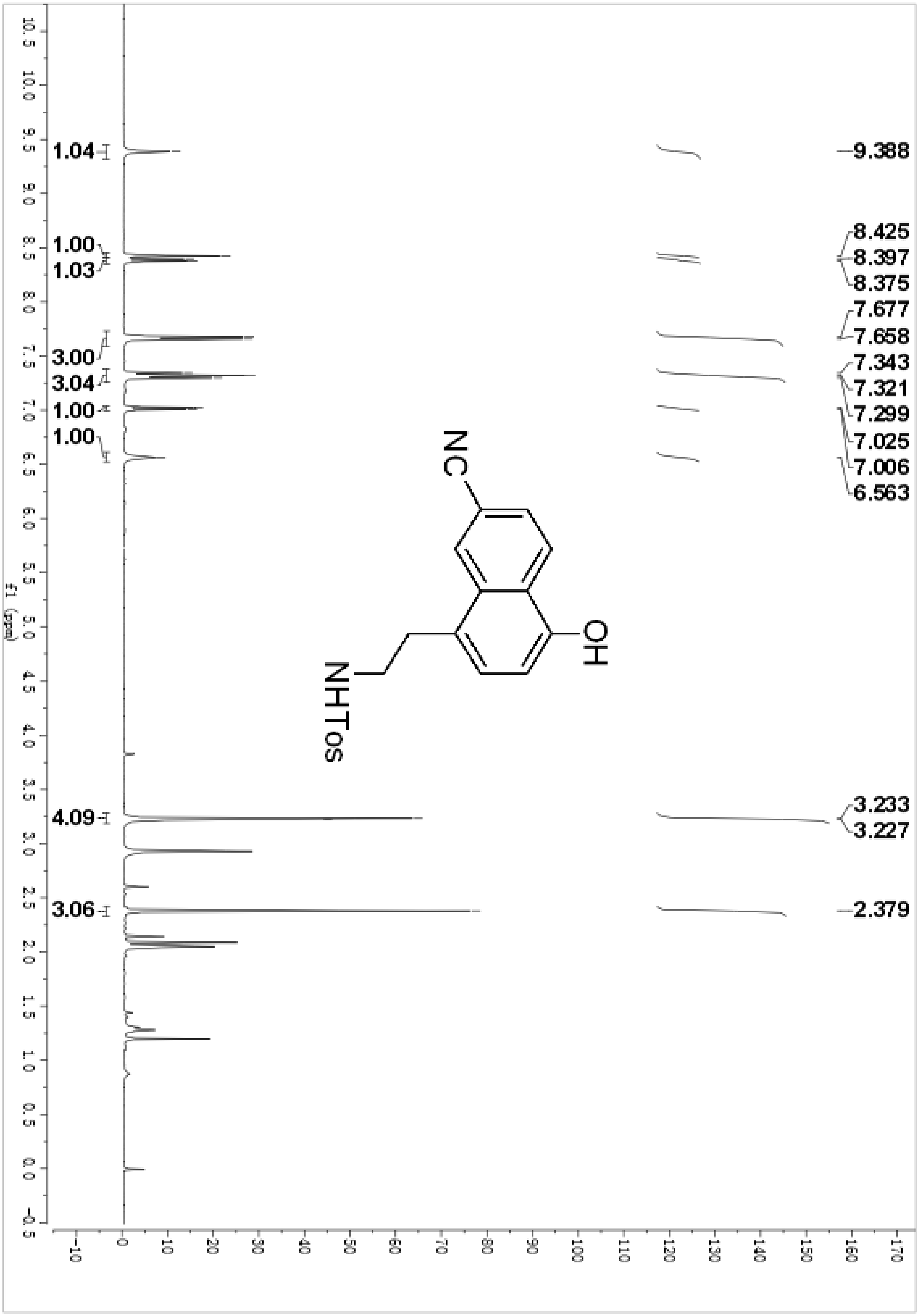

Supplementary Figure 22 ^{13}C NMR of compound **1u** (d_6-Acetone, 100 MHz, room temperature)

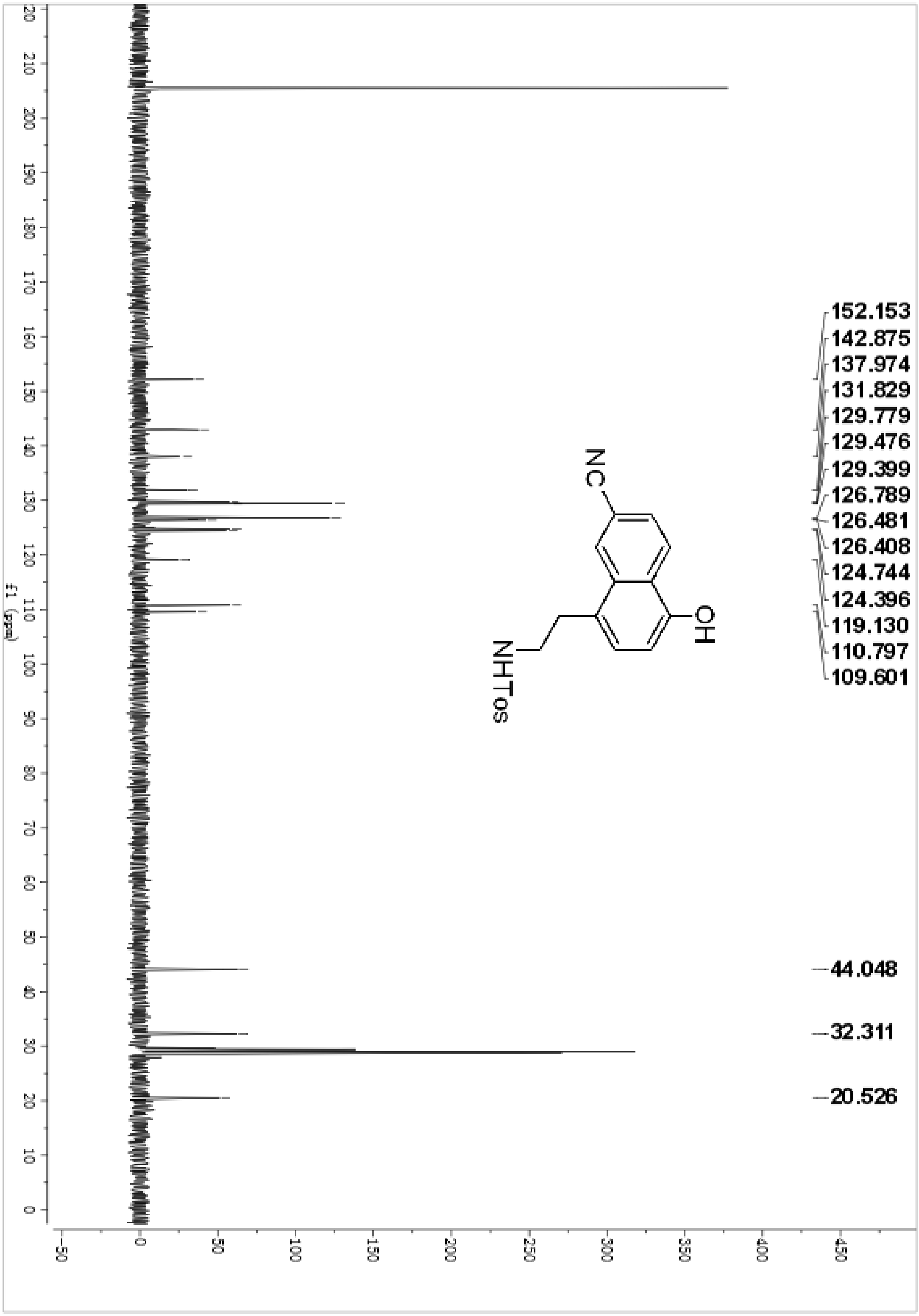

Supplementary Figure 23 ^{1}H NMR of compound **3a** (d_6-Acetone, 400 MHz, room temperature)

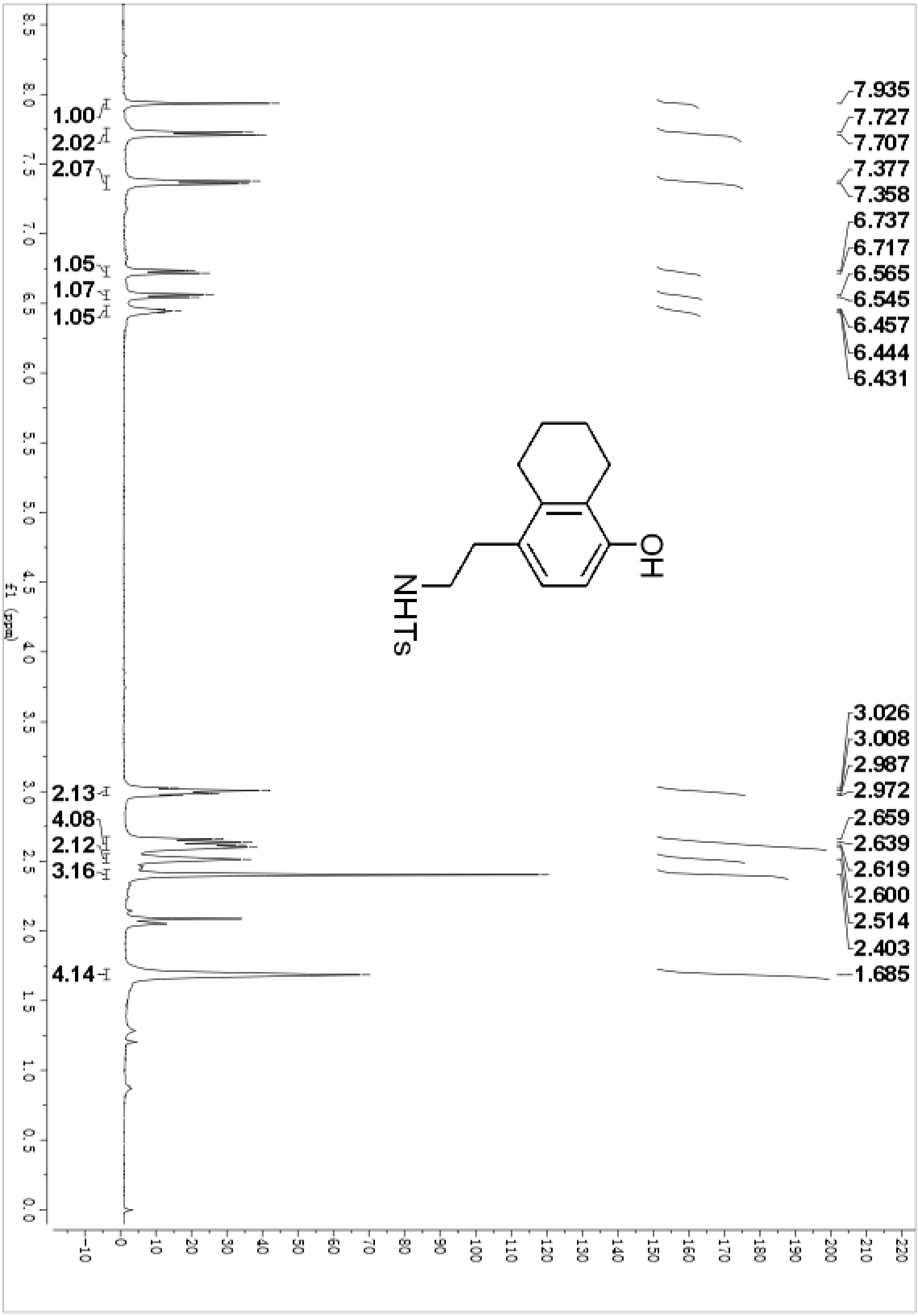

Supplementary Figure 24 ^{13}C NMR of compound **3a** (d_6-Acetone, 100 MHz, room temperature)

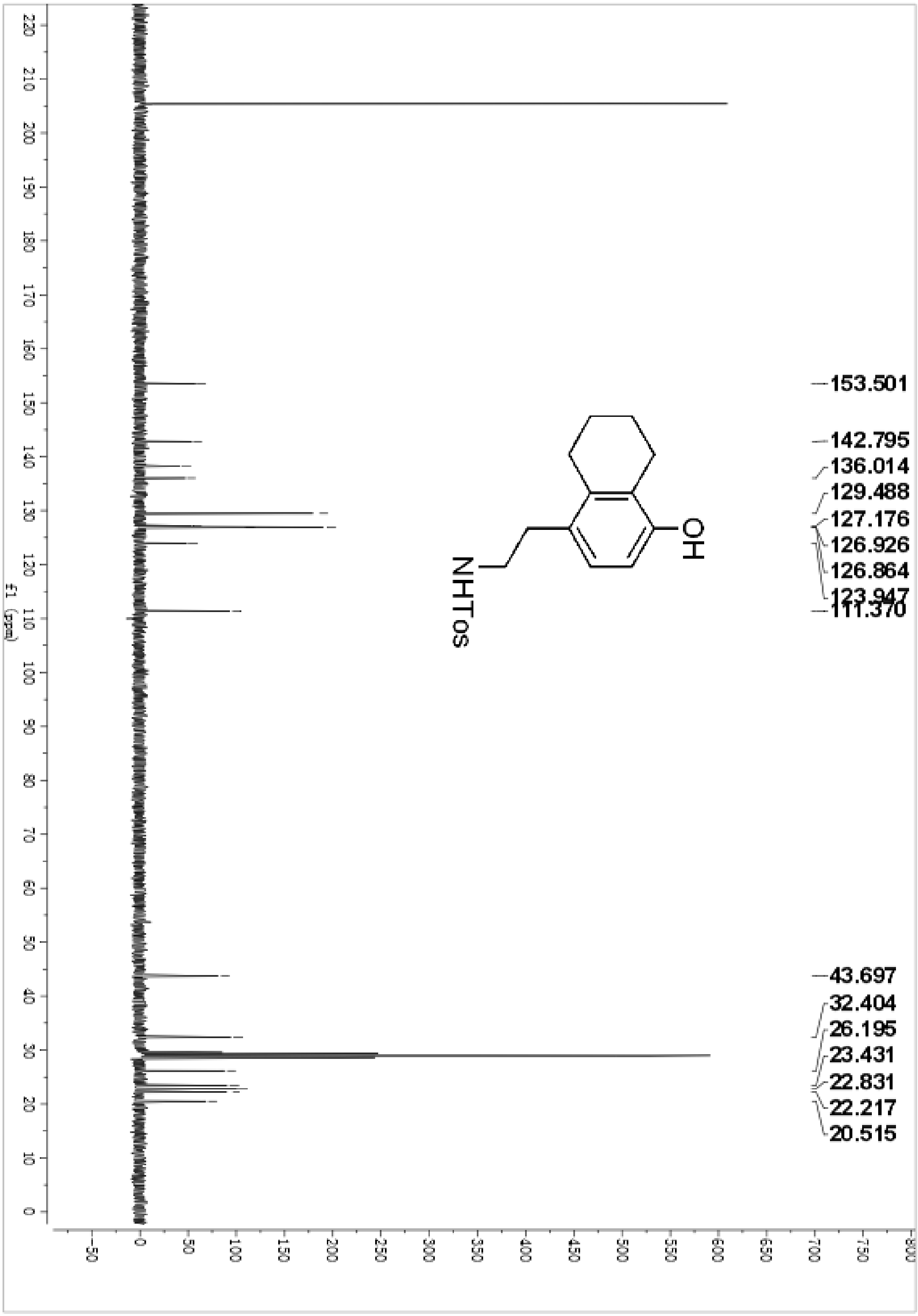

Supplementary Figure 25 ^{1}H NMR of compound **3b** (d_6-Acetone, 400 MHz, room temperature)

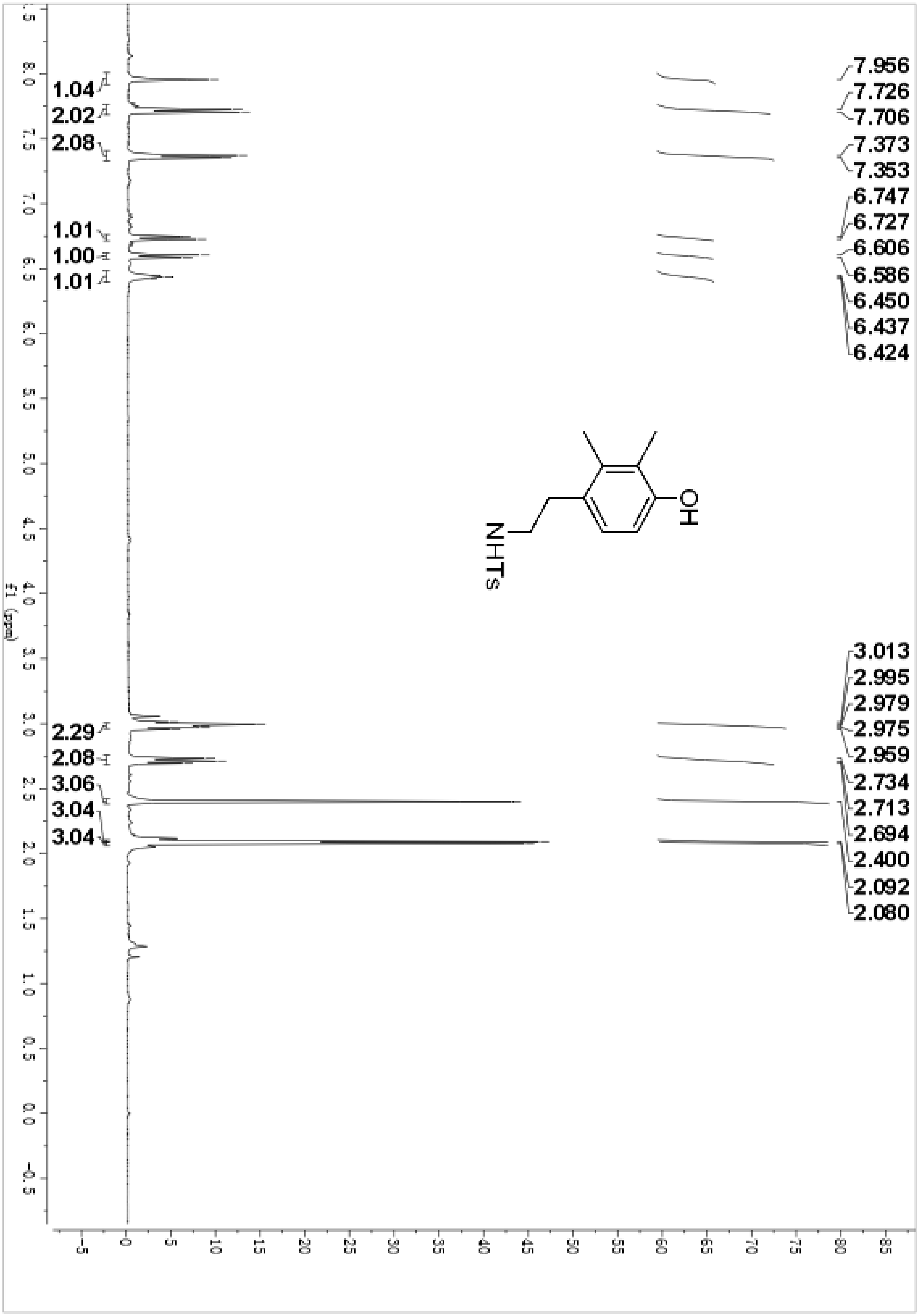

Supplementary Figure 26 ^{13}C NMR of compound **3b** (d_6-Acetone, 100 MHz, room temperature)

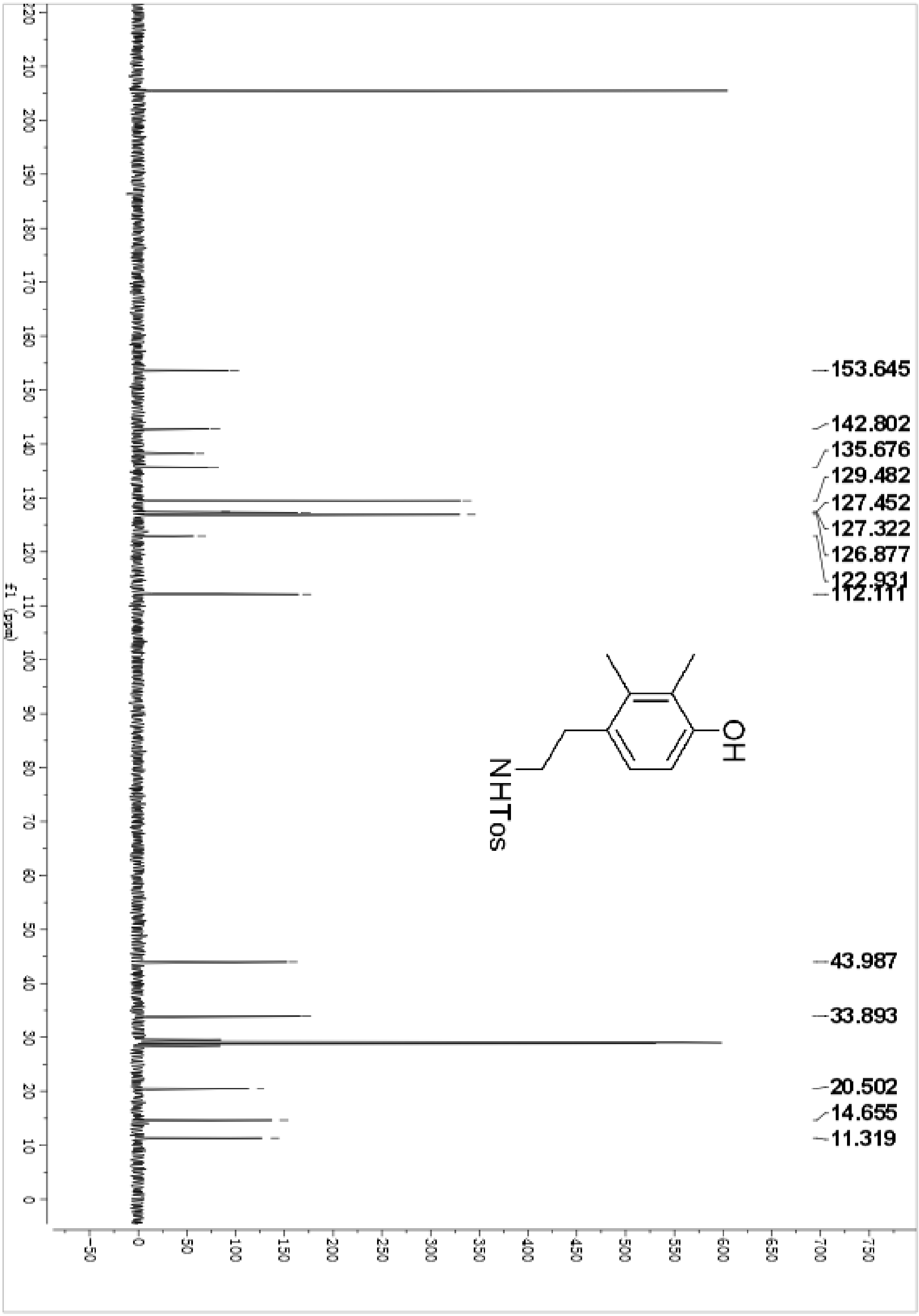

Supplementary Figure 27 ^{1}H NMR of compound **2a** ($CDCl_3$, 600 MHz, 60 °C)

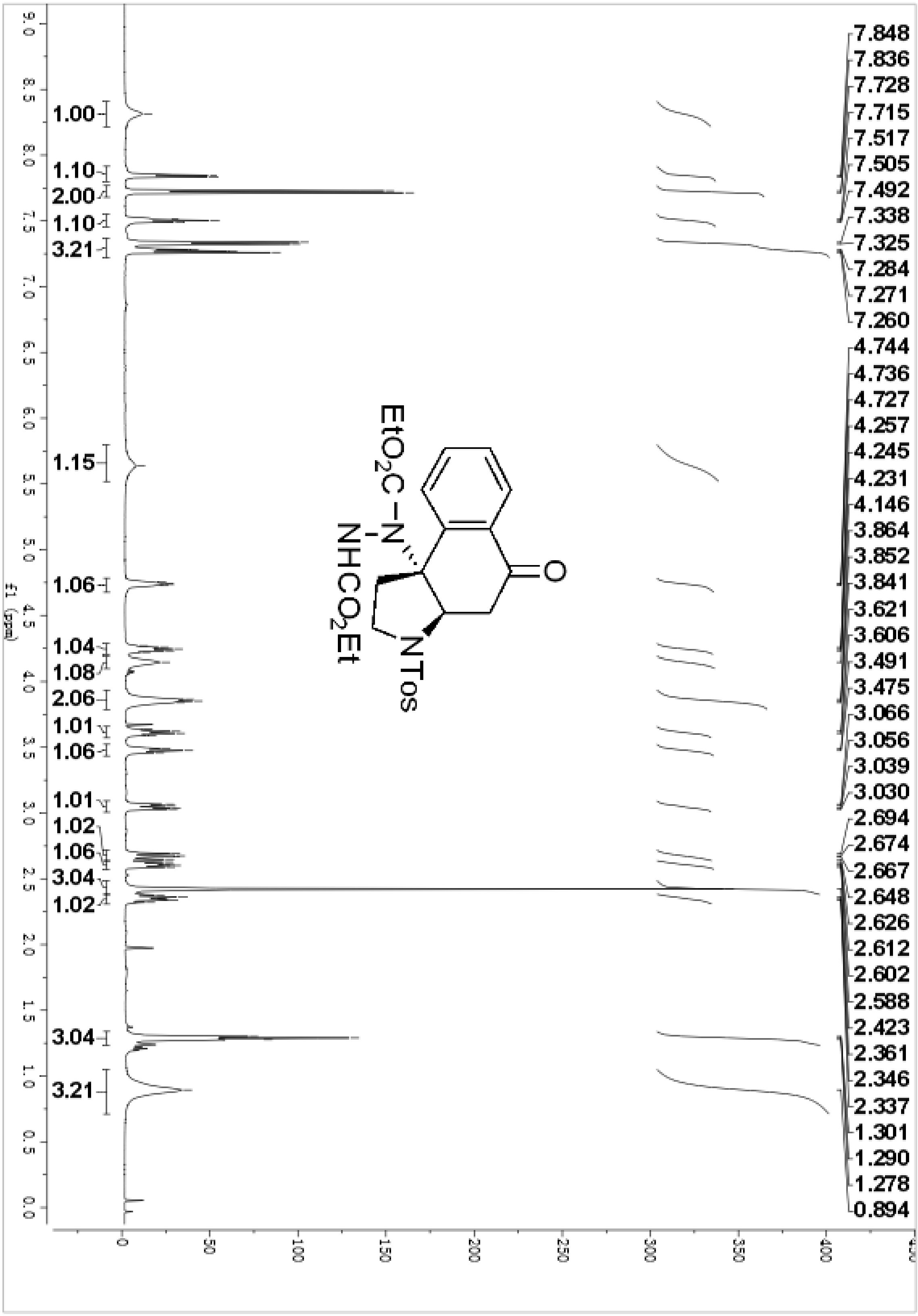

Supplementary Figure 28 ^{13}C NMR of compound **2a** ($CDCl_3$, 150 MHz, 60 °C)

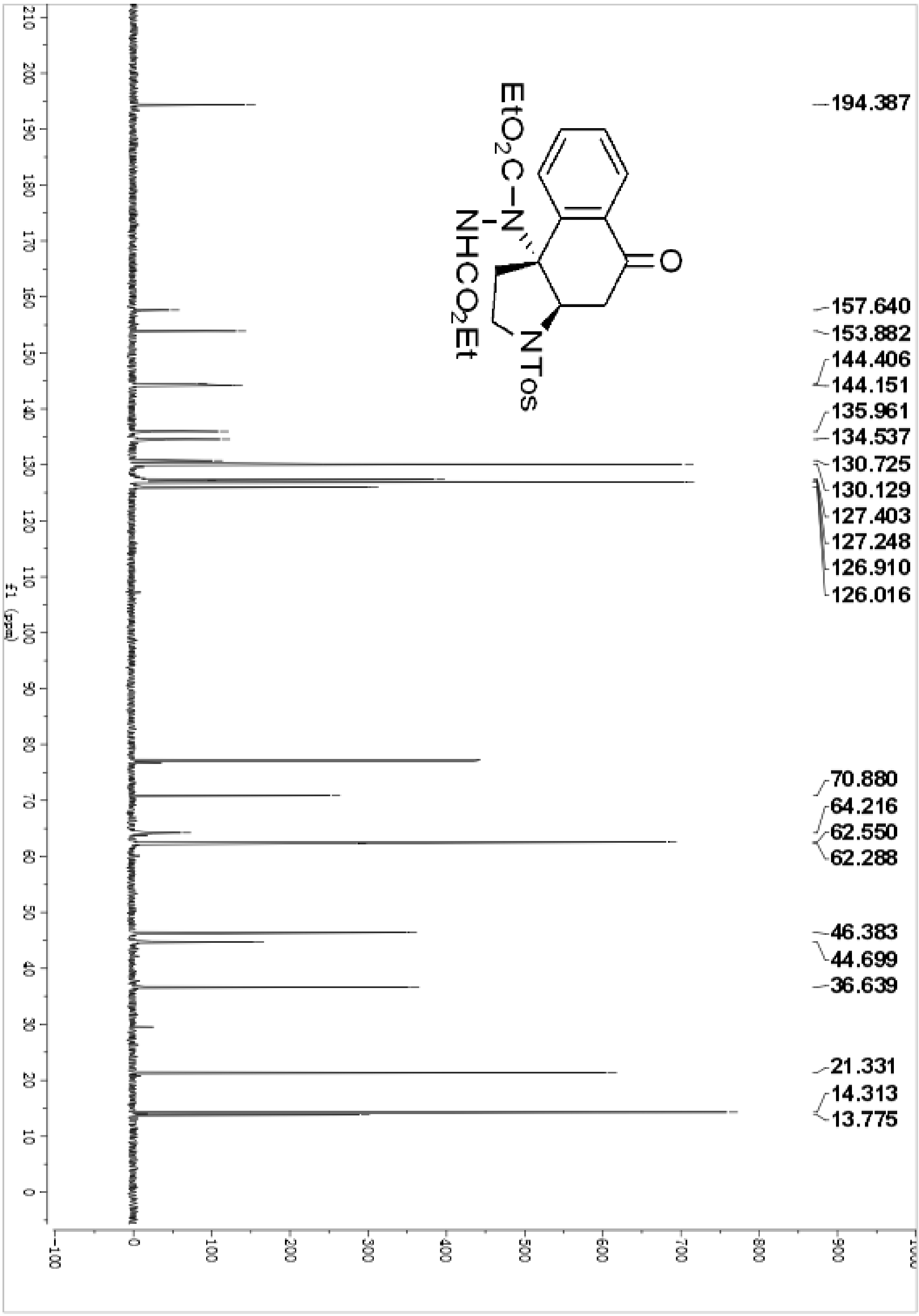

Supplementary Figure 29 ^{1}H NMR of compound **2b** ($CDCl_3$, 600 MHz, 60 °C)

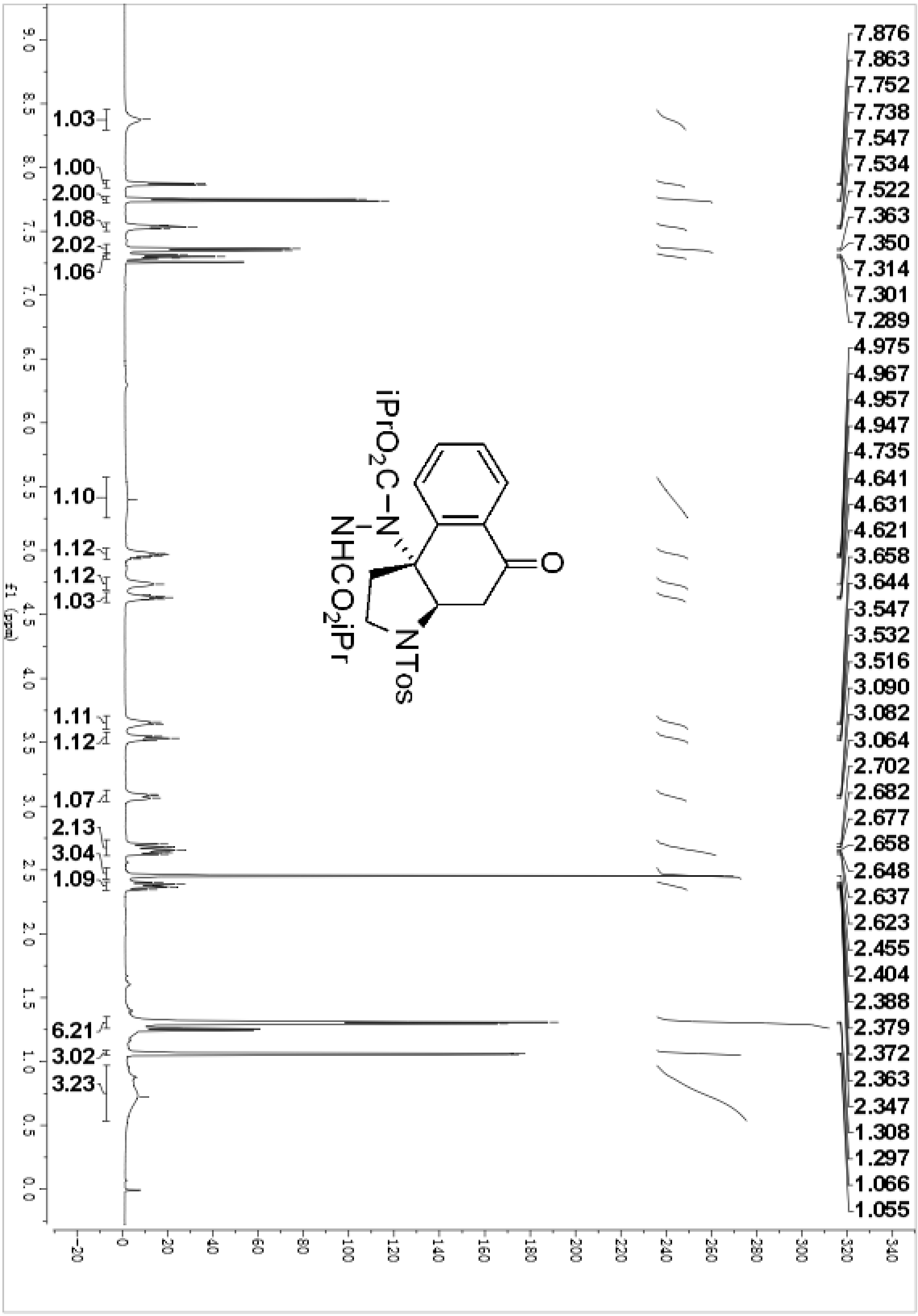

Supplementary Figure 30 ^{13}C NMR of compound **2b** ($CDCl_3$, 150 MHz, 60 °C)

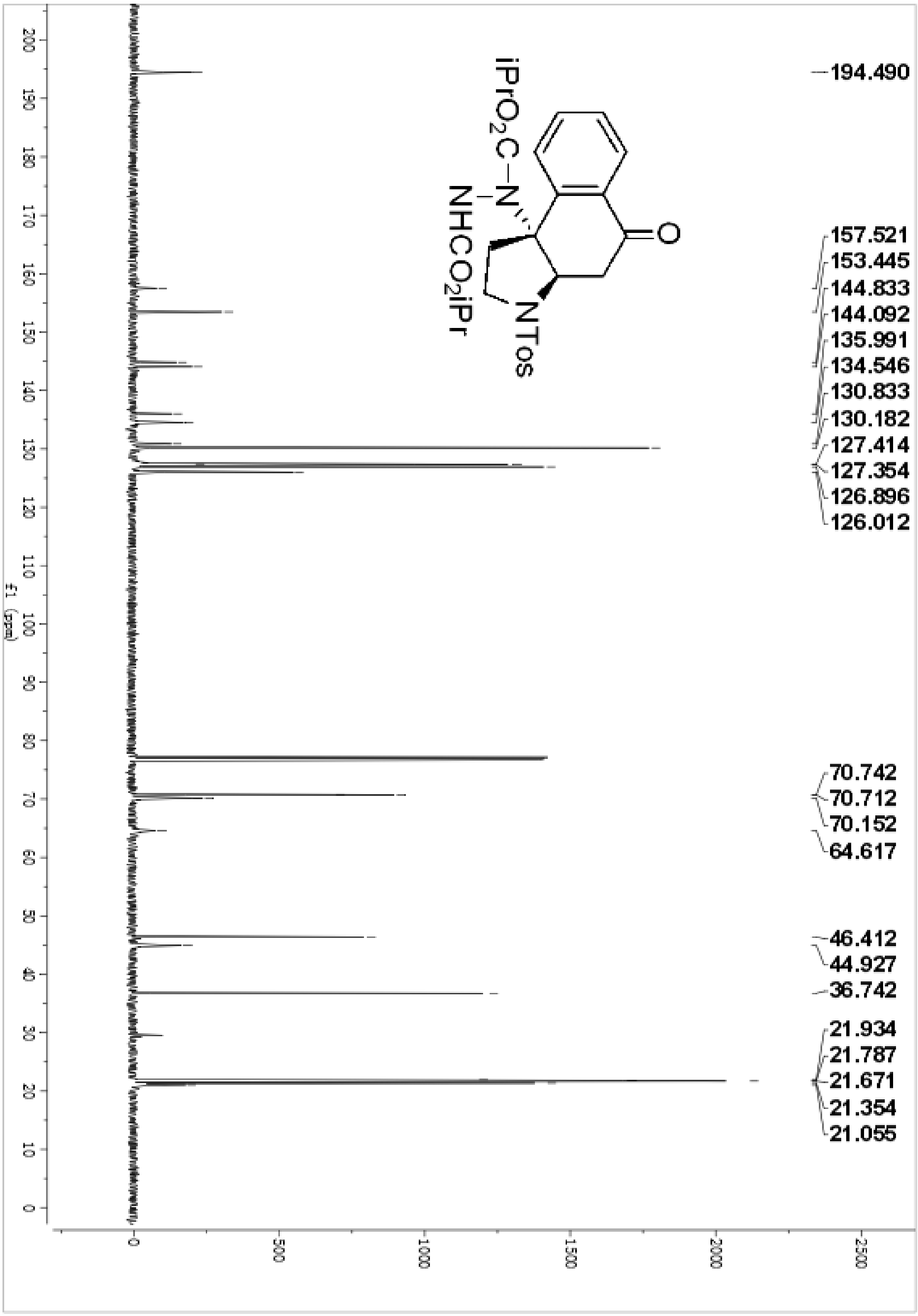

Supplementary Figure 31 ^{1}H NMR of compound **2c** ($CDCl_3$, 600 MHz, 60 °C)

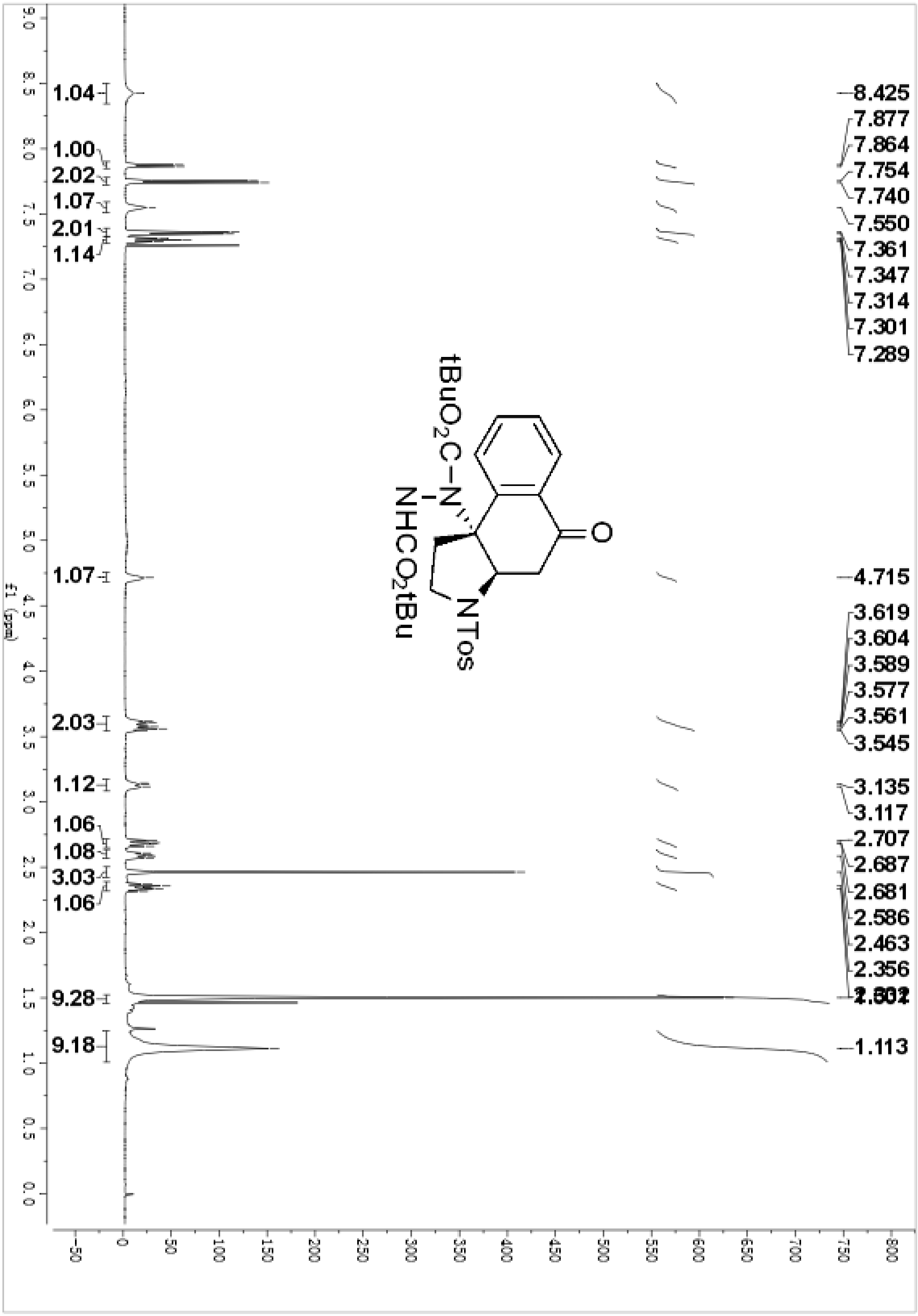

Supplementary Figure 32 ^{13}C NMR of compound **2c** ($CDCl_3$, 150 MHz, 60 °C)

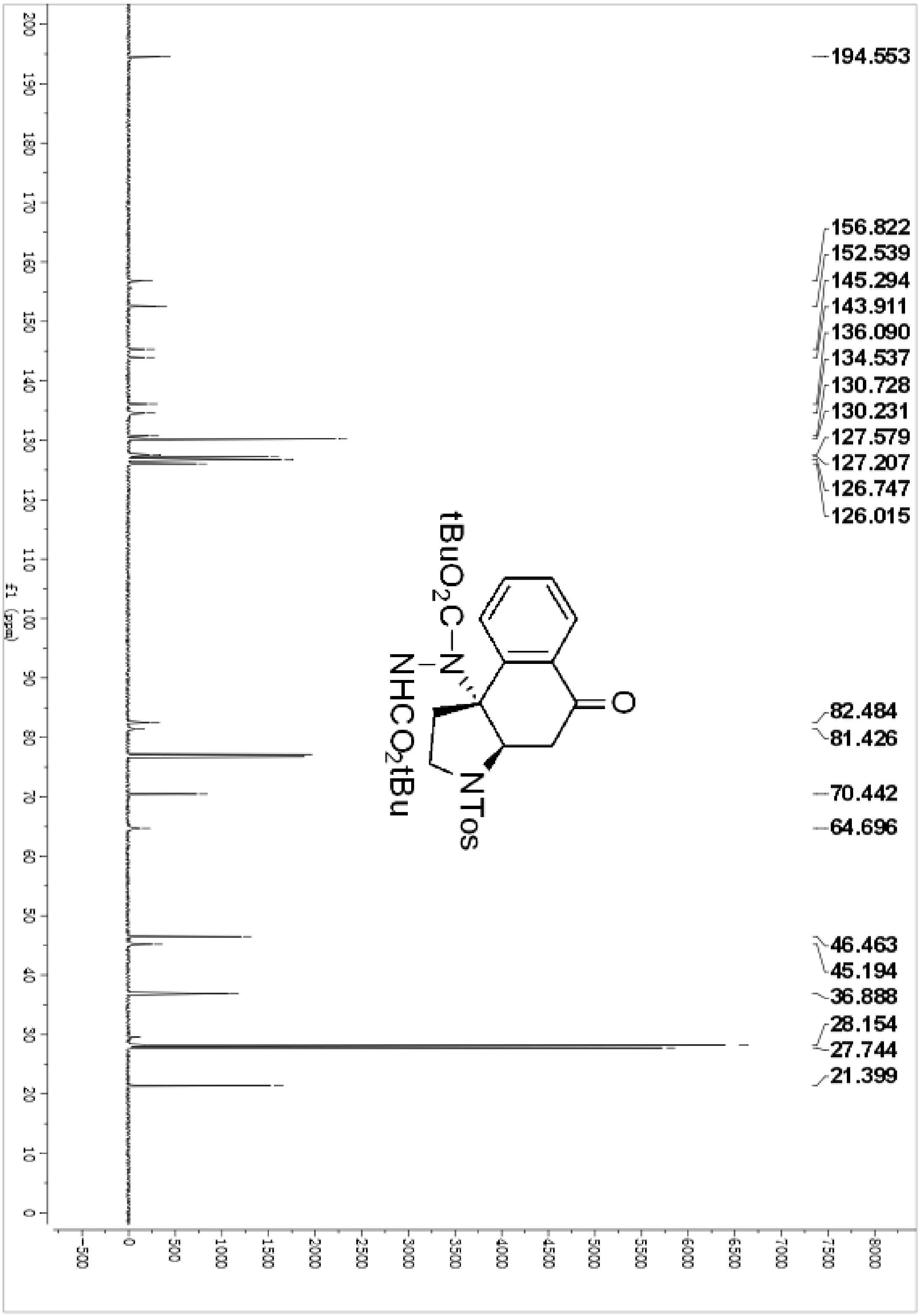

Supplementary Figure 33 ^{1}H NMR of compound **2d** (CD_3CN, 600 MHz, 80 °C)

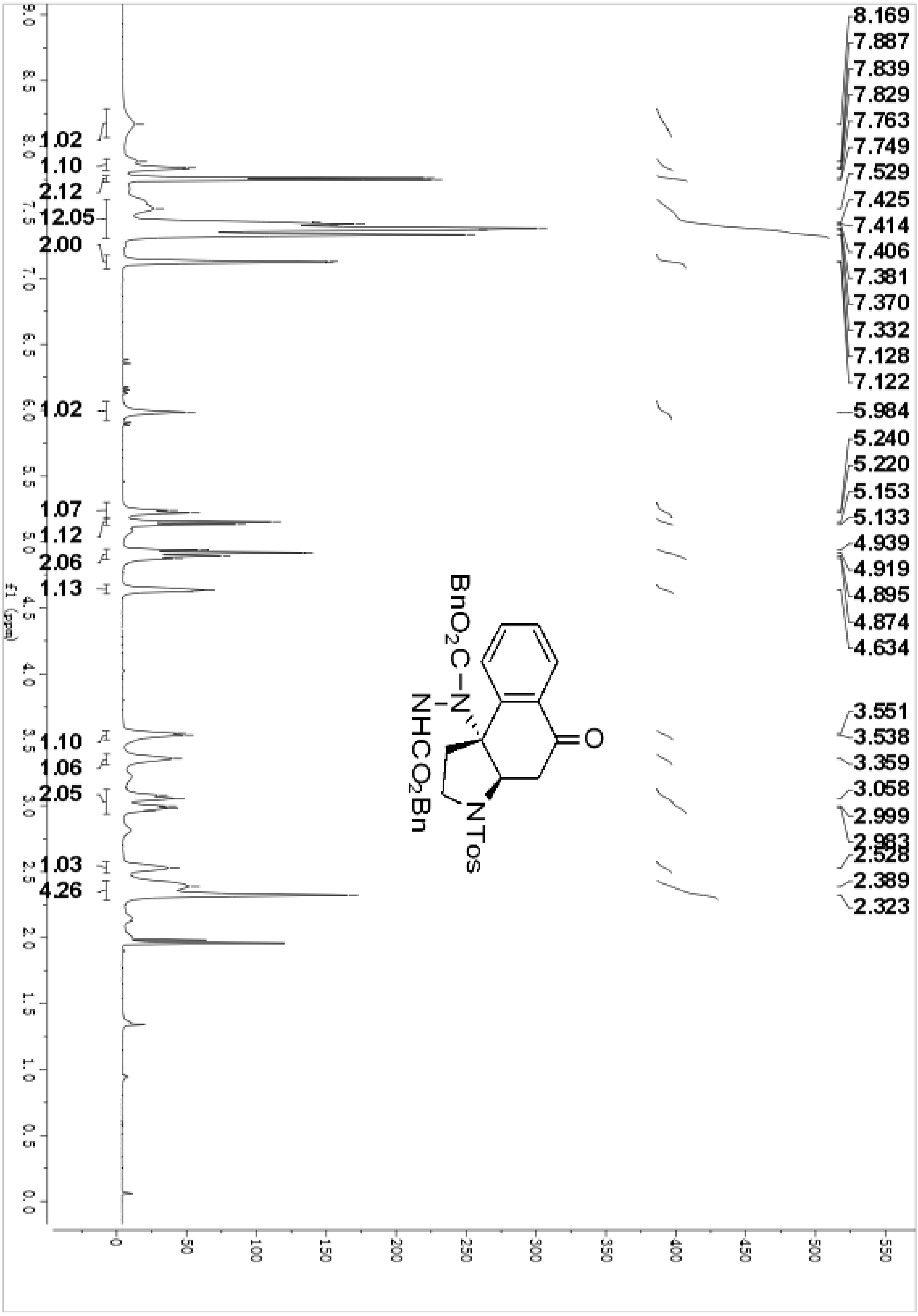

Supplementary Figure 34 ^{13}C NMR of compound **2d** (CD_3CN, 150 MHz, 80 °C)

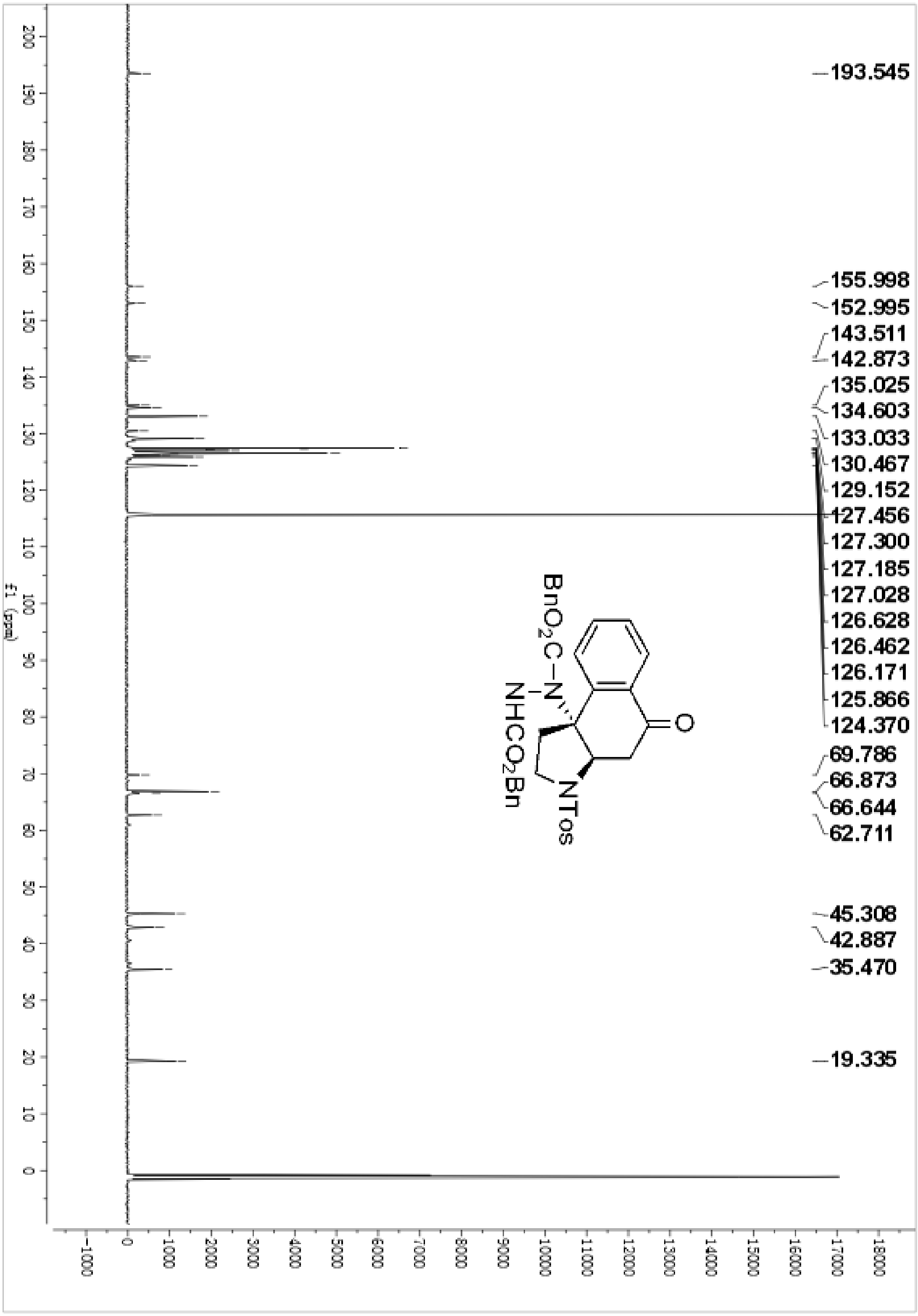

Supplementary Figure 35 ^{1}H NMR of compound **2e** (CD_3CN, 600 MHz, 80 °C)

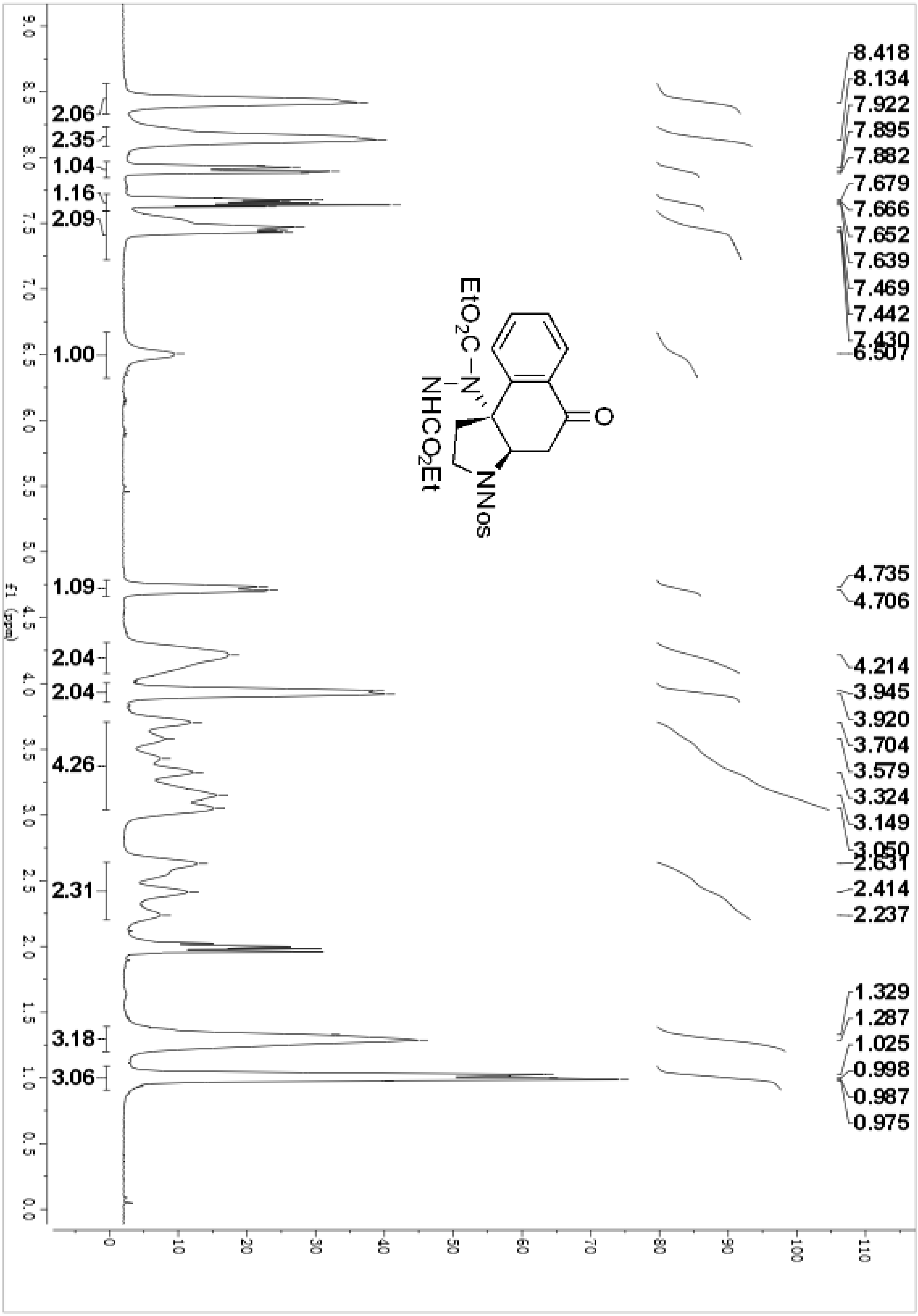

Supplementary Figure 36 ^{13}C NMR of compound **2e** (CD_3CN, 150 MHz, 80 °C)

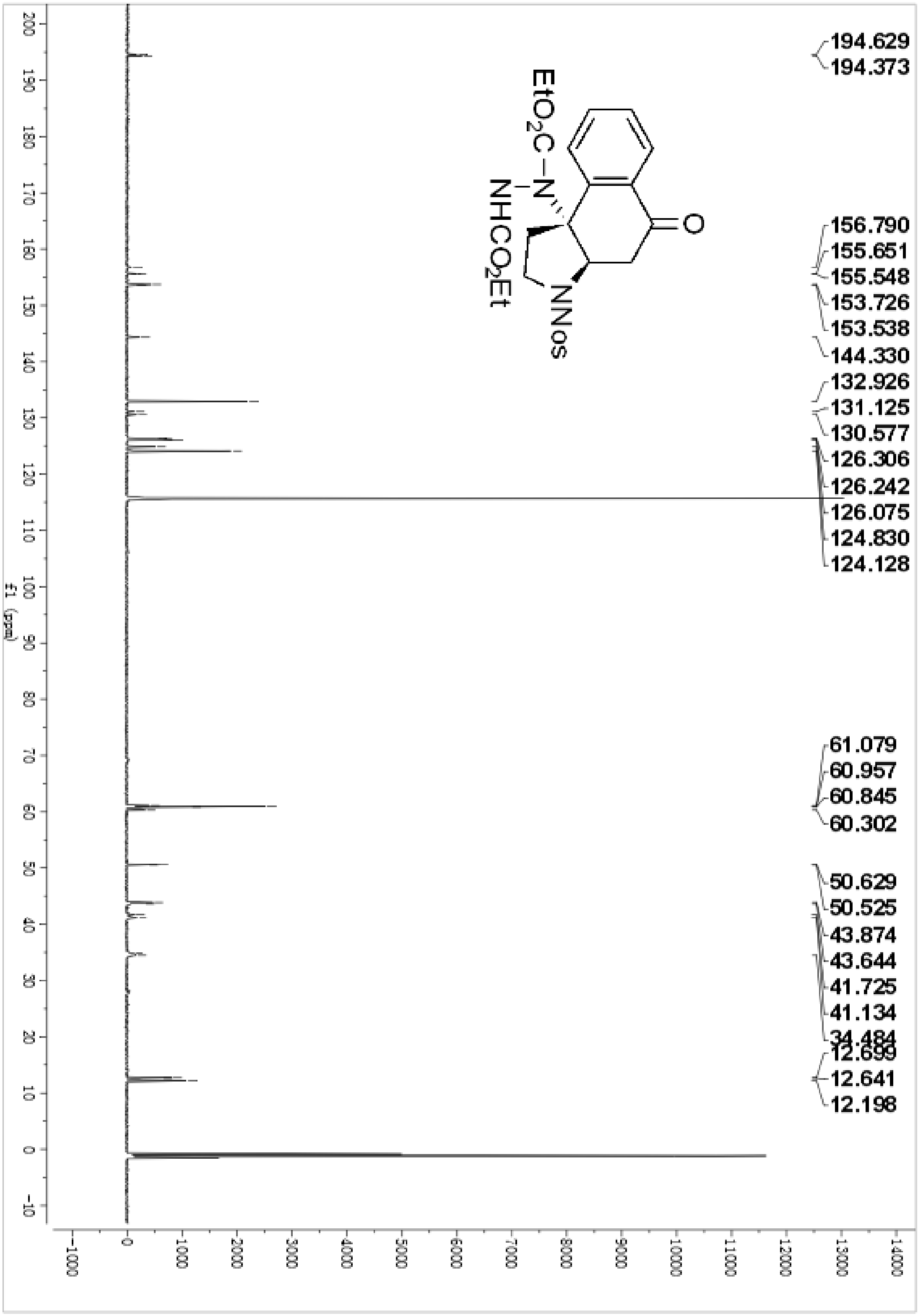

Supplementary Figure 37 ^{1}H NMR of compound **2f** (CD_3CN, 600 MHz, 80 °C)

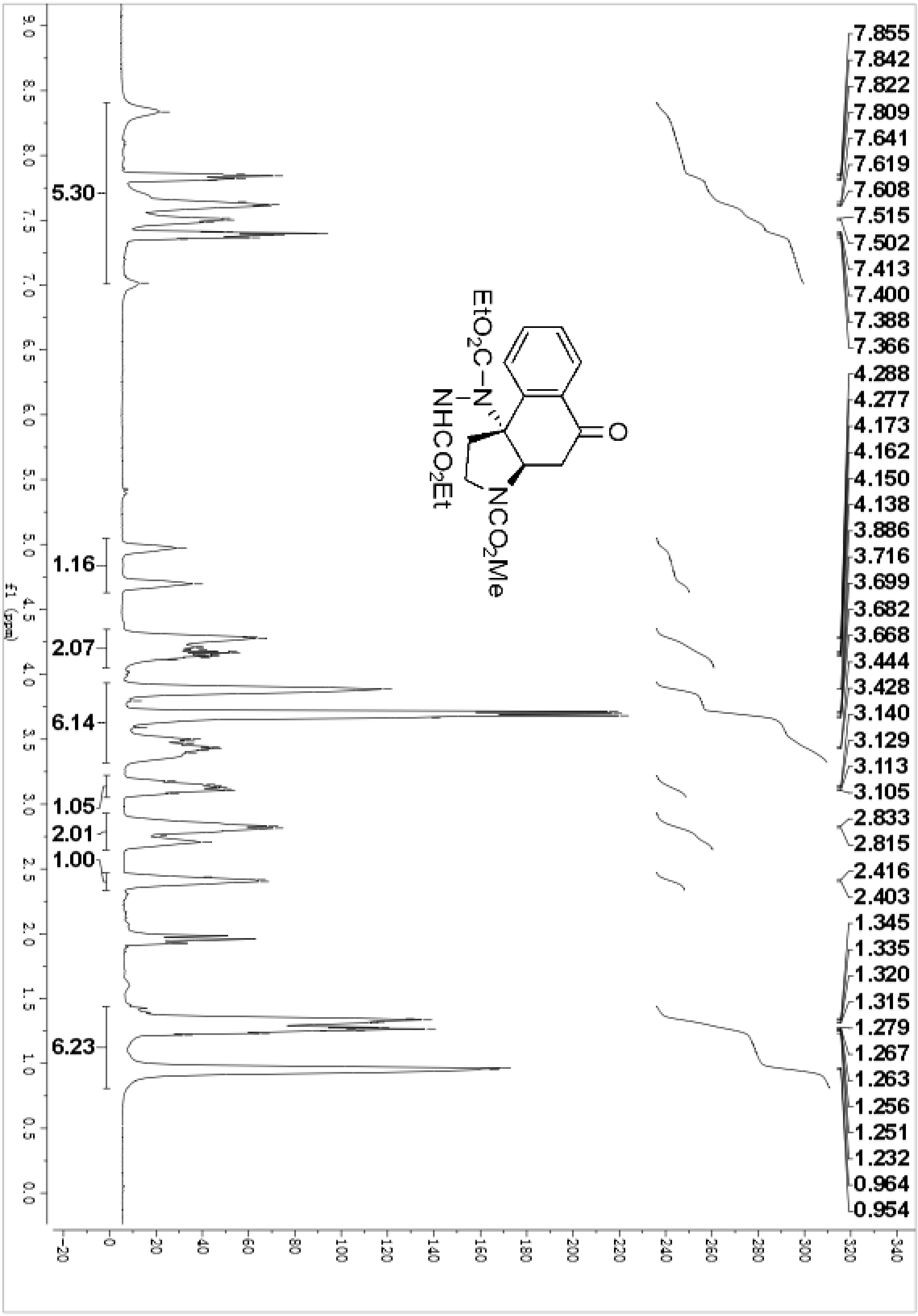

Supplementary Figure 38 ^{13}C NMR of compound **2f** (CD_3CN, 150 MHz, 80 °C)

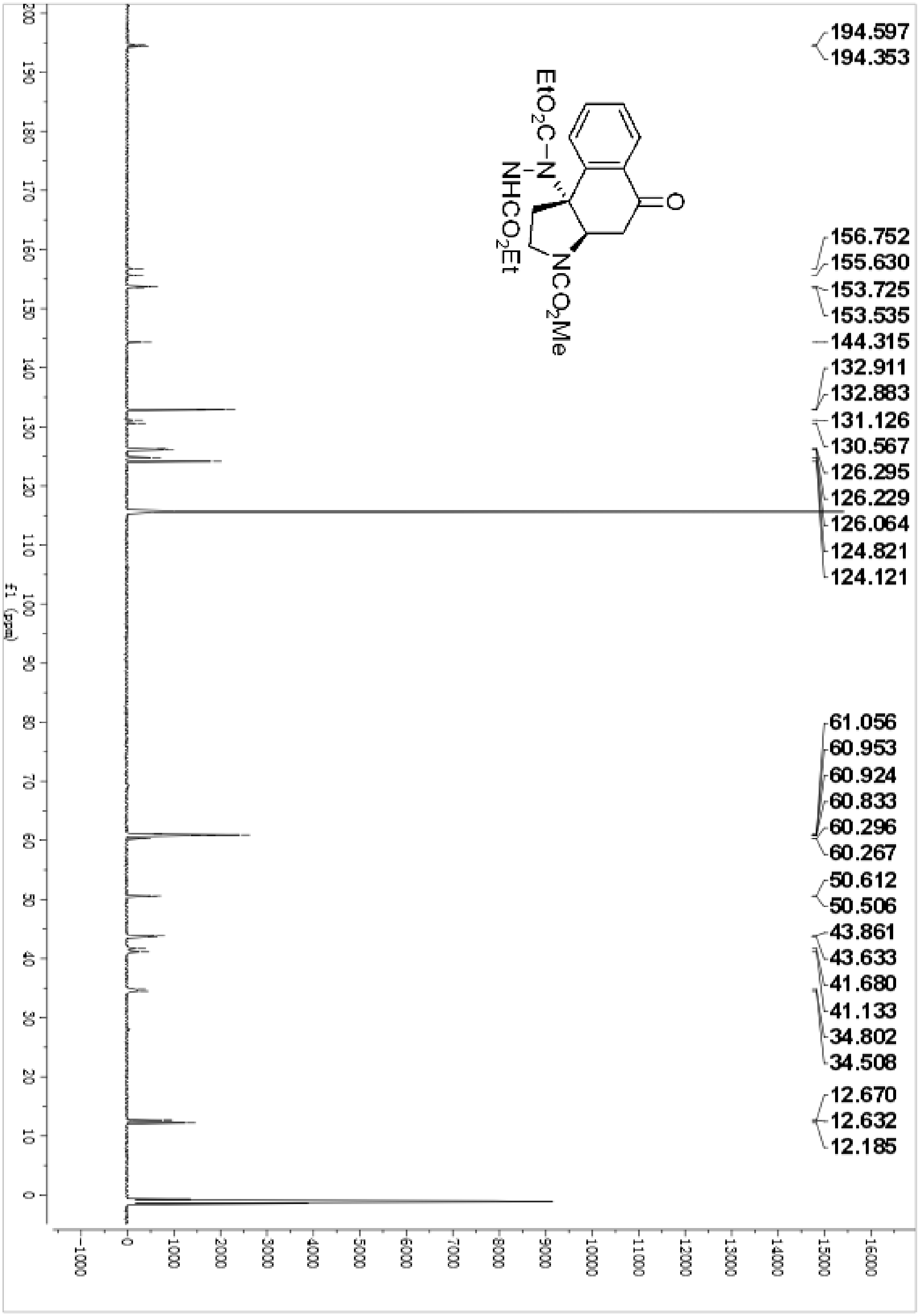

Supplementary Figure 39 ^{1}H NMR of compound **2g** (CD_3CN, 600 MHz, 80 °C)

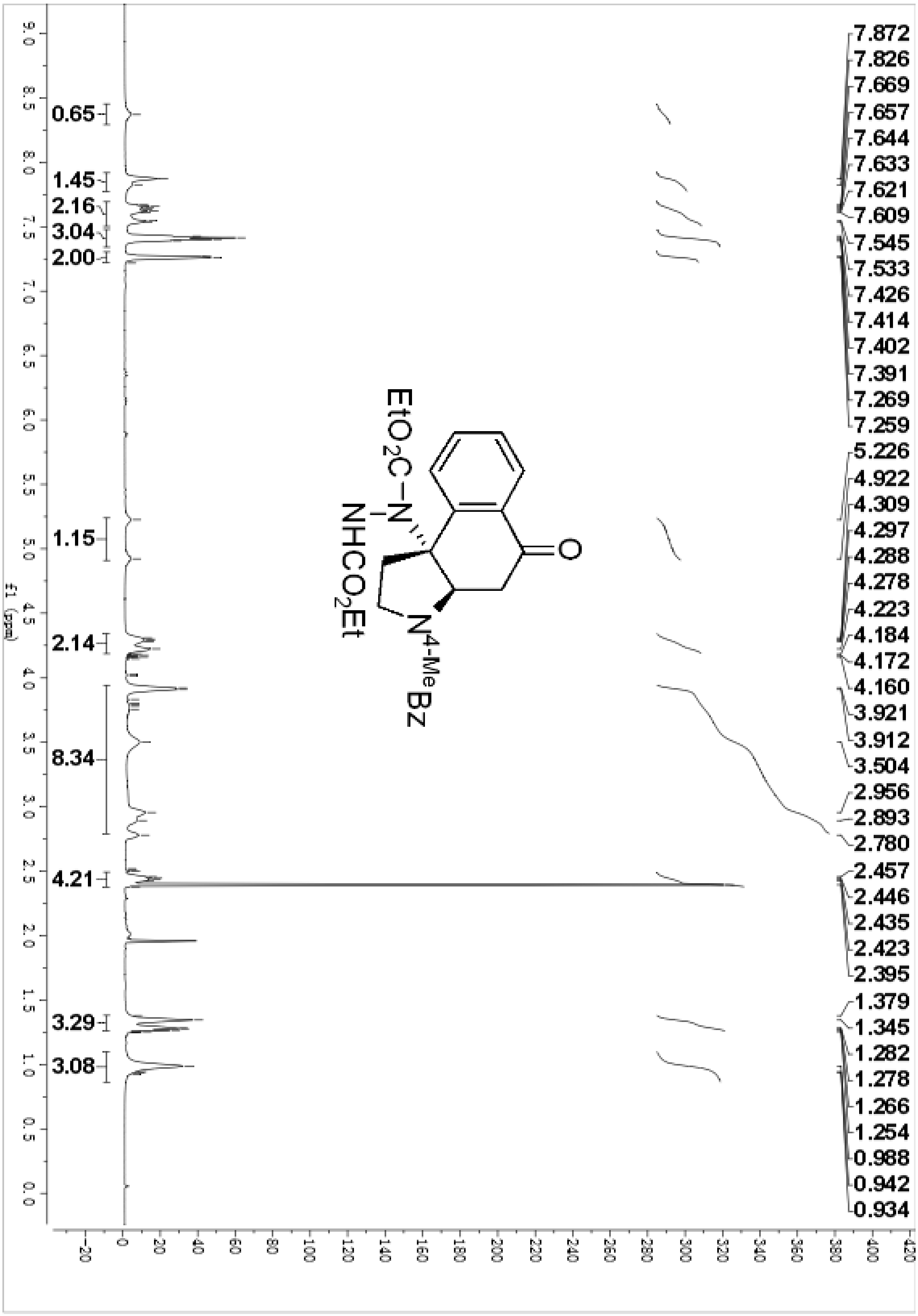

Supplementary Figure 40 ^{13}C NMR of compound **2g** (CD_3CN, 150 MHz, 80 °C)

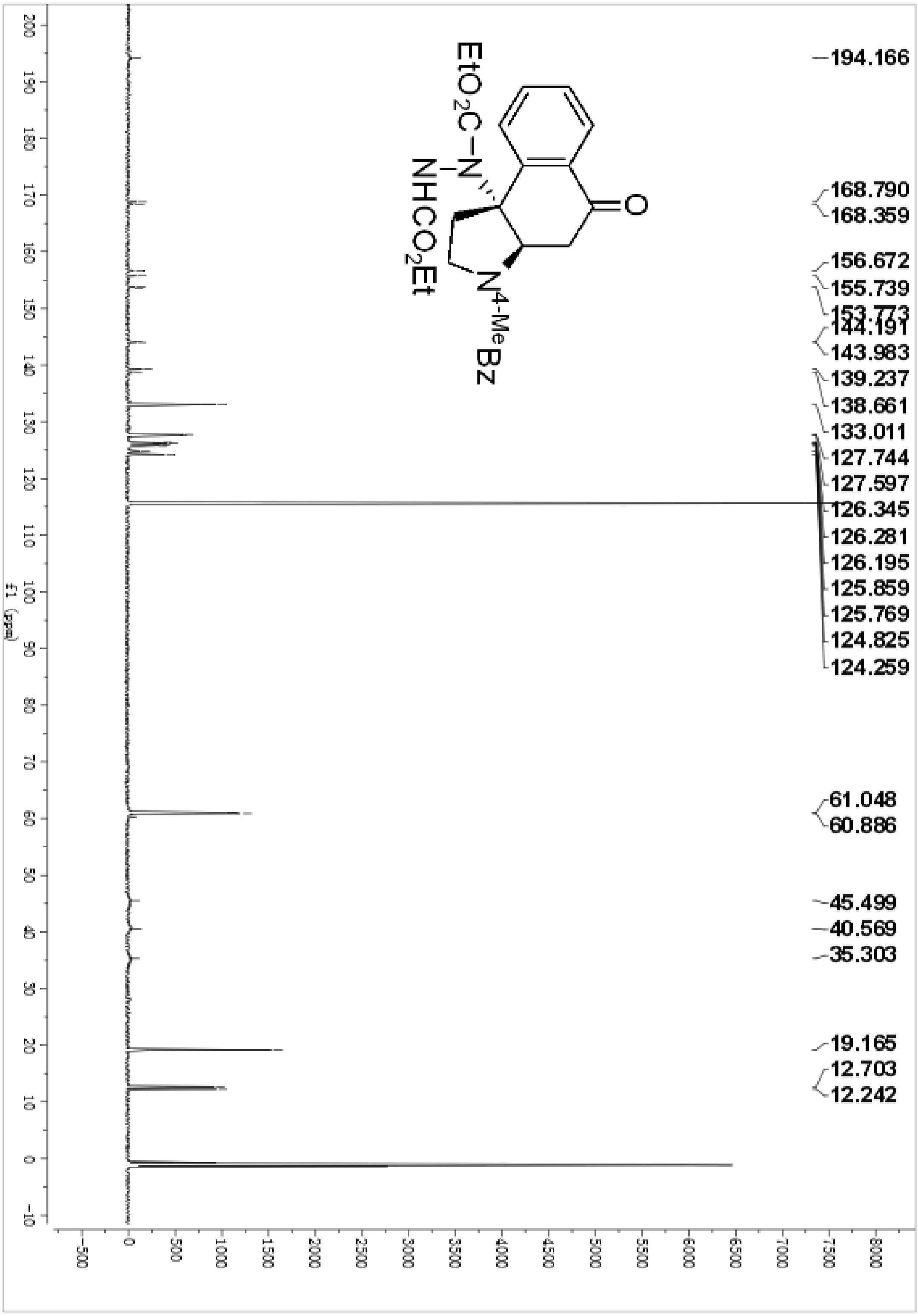

Supplementary Figure 41 ^{1}H NMR of compound **2h** ($CDCl_3$, 600 MHz, 60 °C)

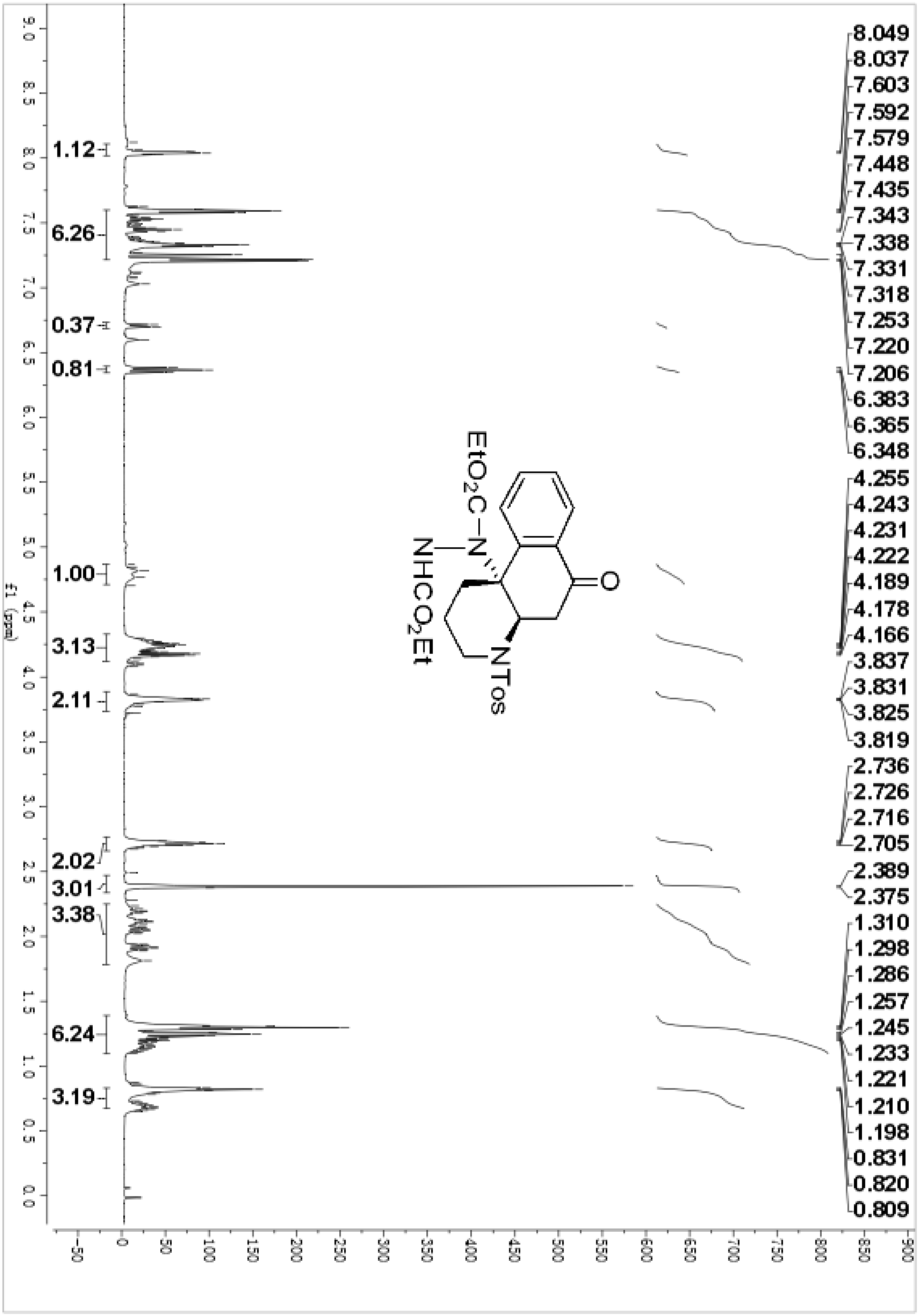

Supplementary Figure 42 ^{13}C NMR of compound **2h** ($CDCl_3$, 150 MHz, 60 °C)

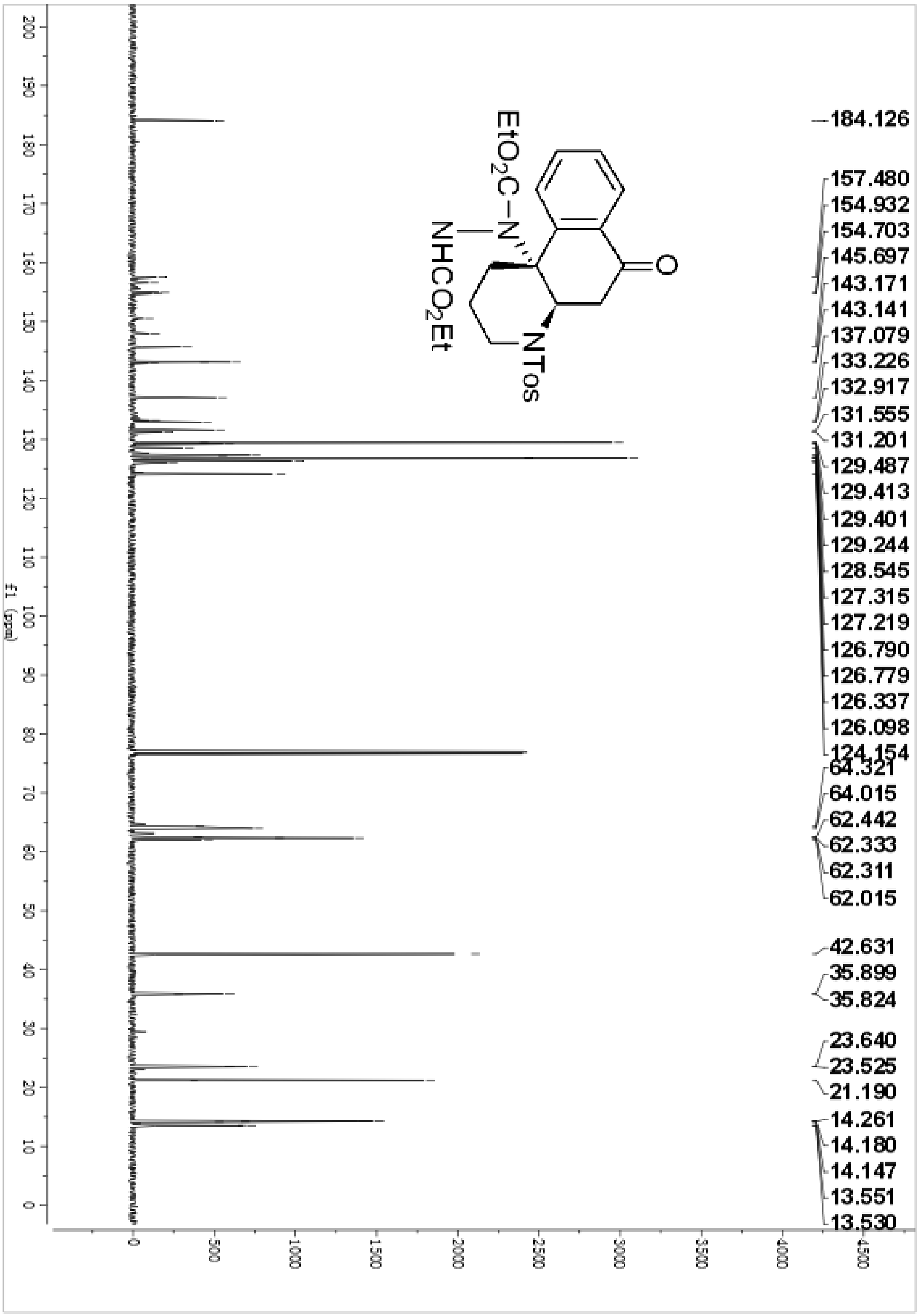

Supplementary Figure 43 ^{1}H NMR of compound **2i** (CD_3CN, 600 MHz, 80 °C)

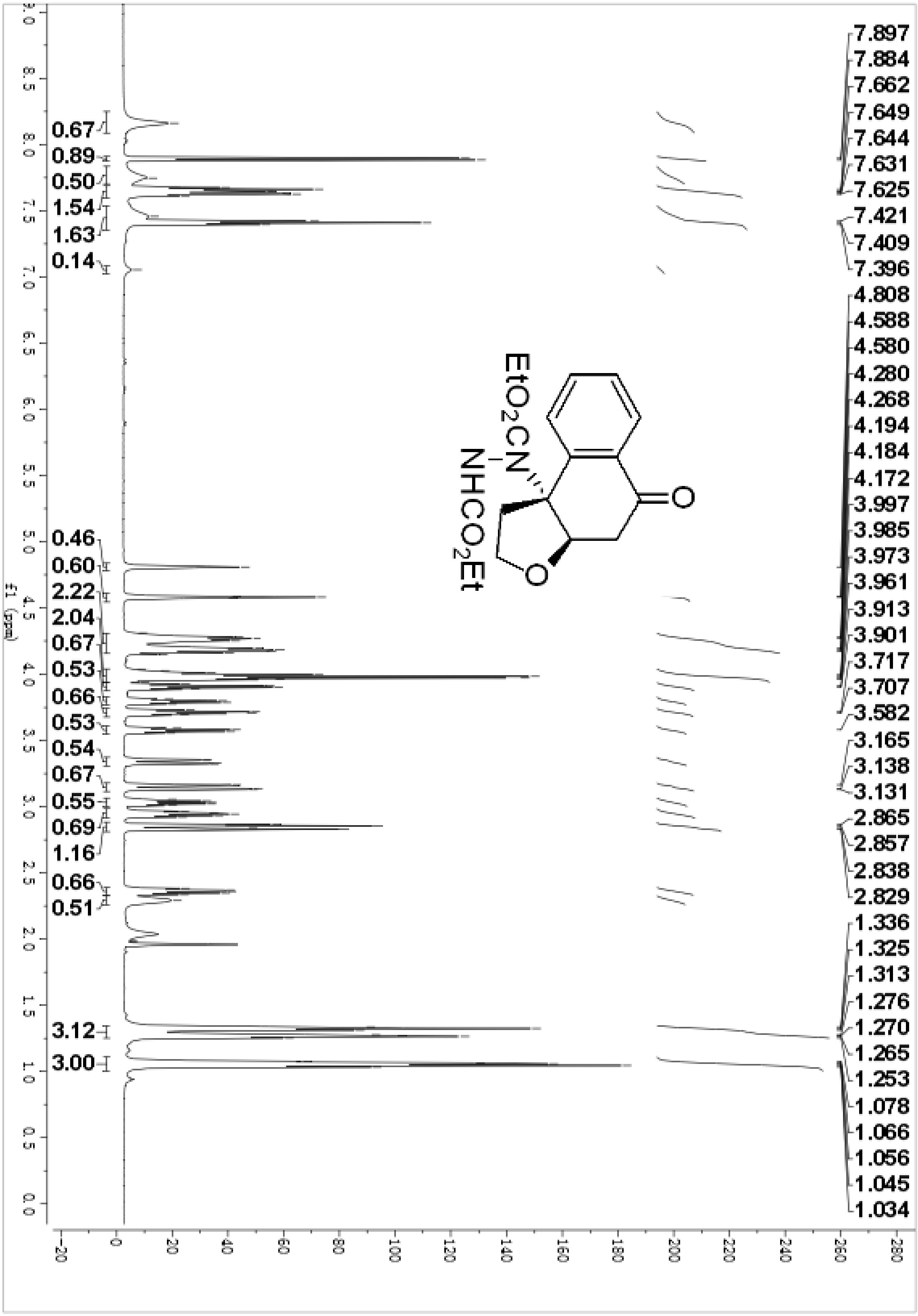

Supplementary Figure 44 ^{13}C NMR of compound **2i** (CD_3CN, 150 MHz, 80 °C)

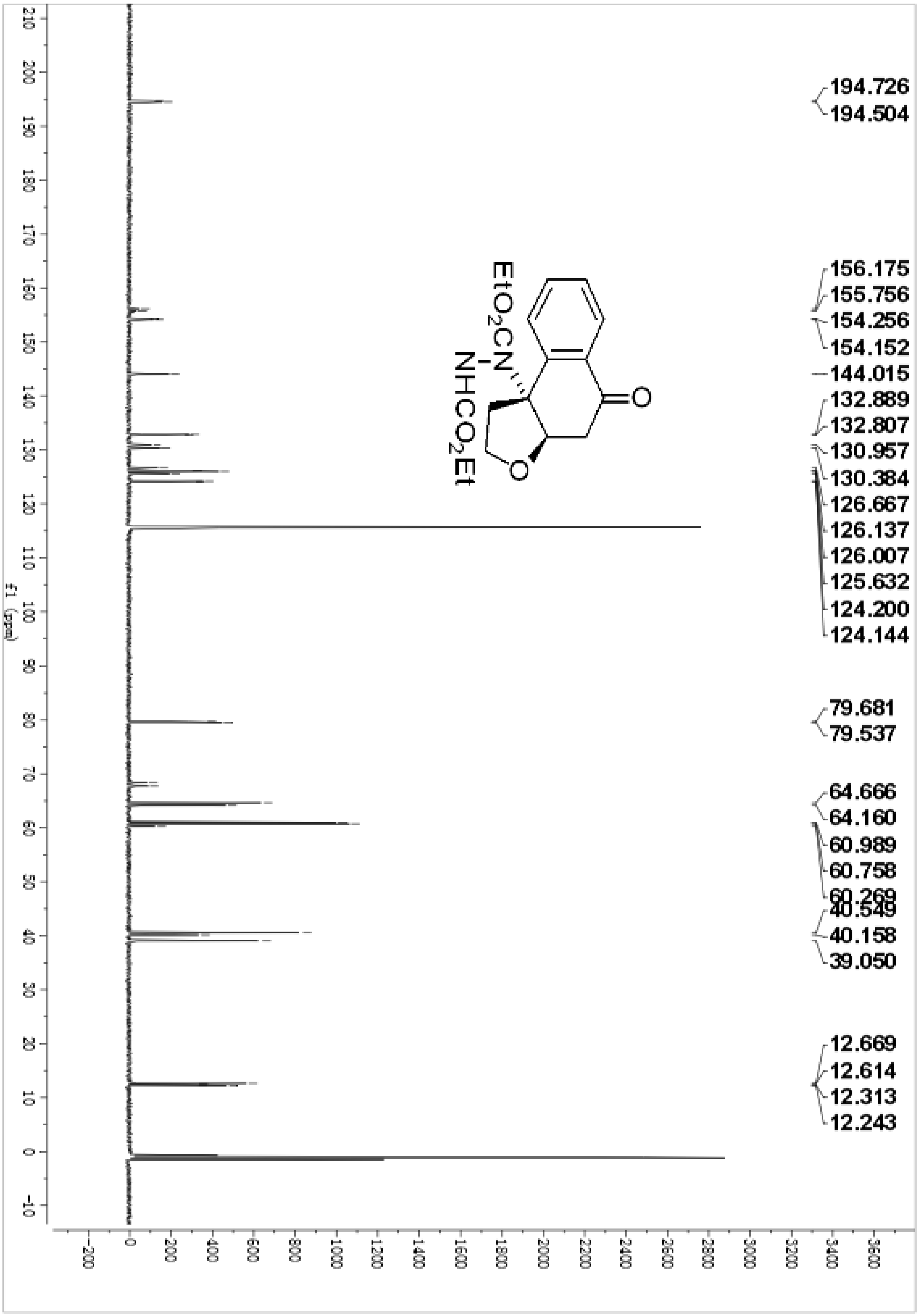

Supplementary Figure 45 ^{1}H NMR of compound **2j** (CD_3CN, 600 MHz, 80 °C)

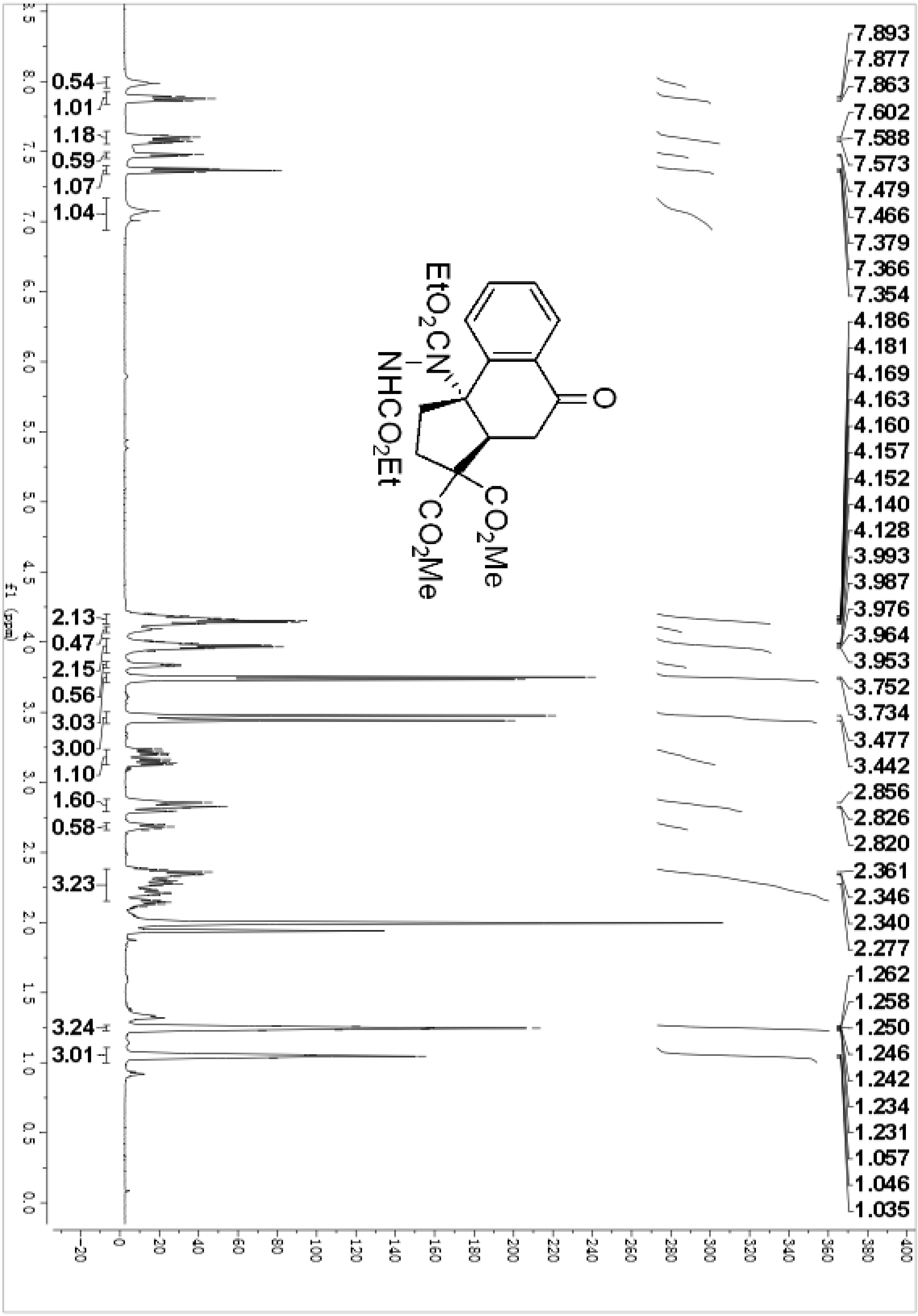

Supplementary Figure 46 ^{13}C NMR of compound **2j** (CD_3CN, 150 MHz, 80 °C)

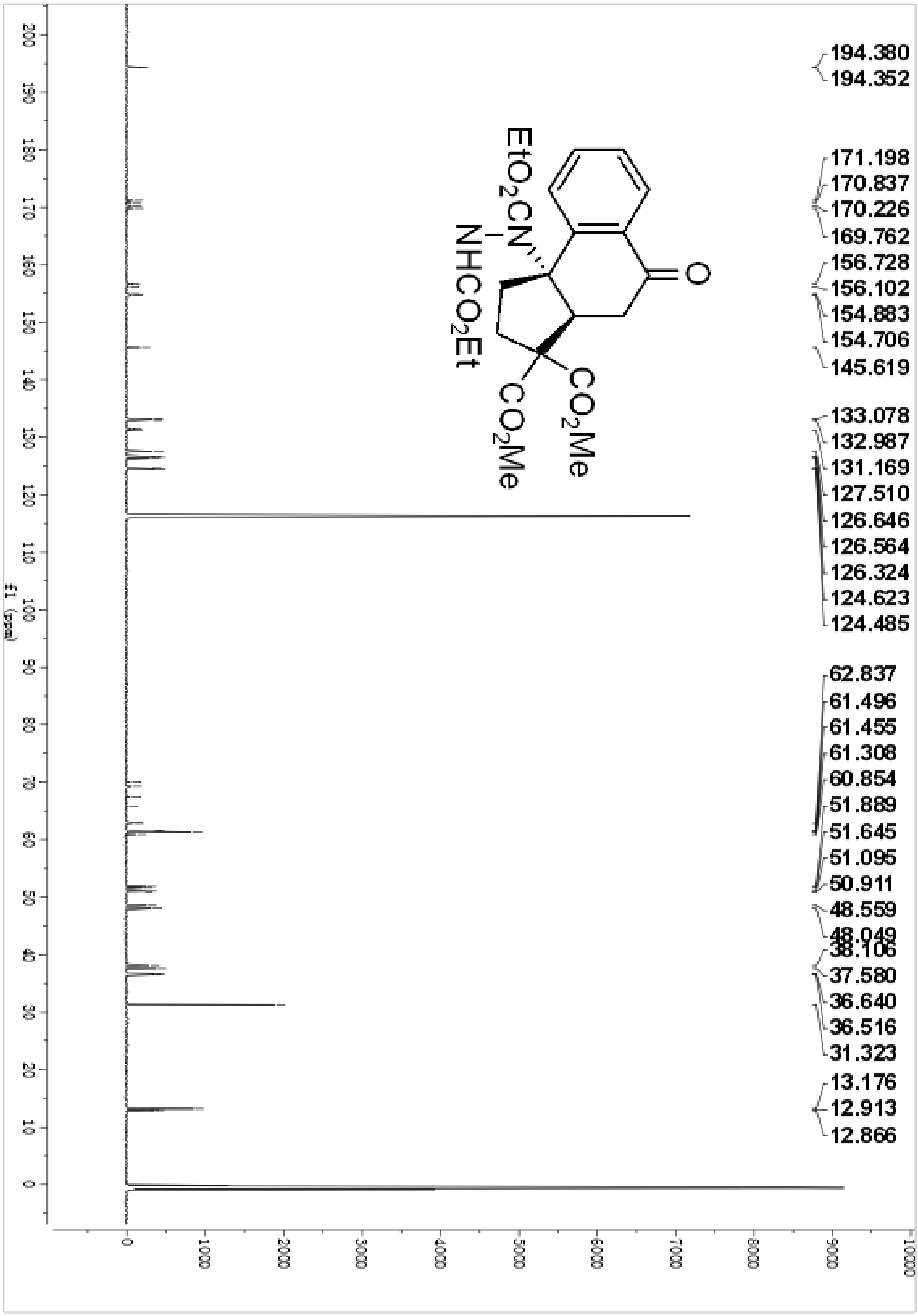

Supplementary Figure 47 ^{1}H NMR of compound **2k** ($CDCl_3$, 600 MHz, 60 °C)

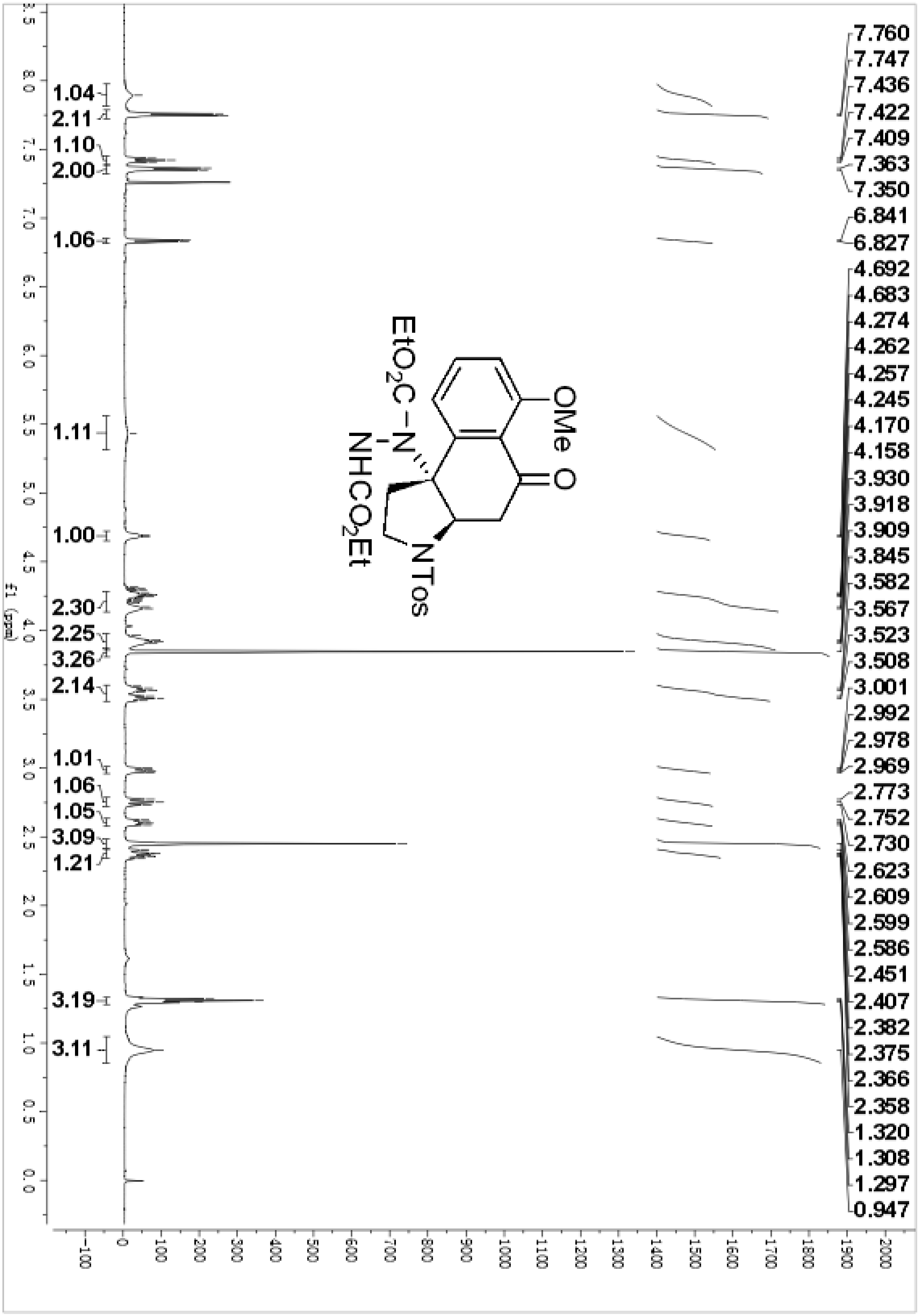

Supplementary Figure 48 ^{13}C NMR of compound **2k** ($CDCl_3$, 100 MHz, 60 °C)

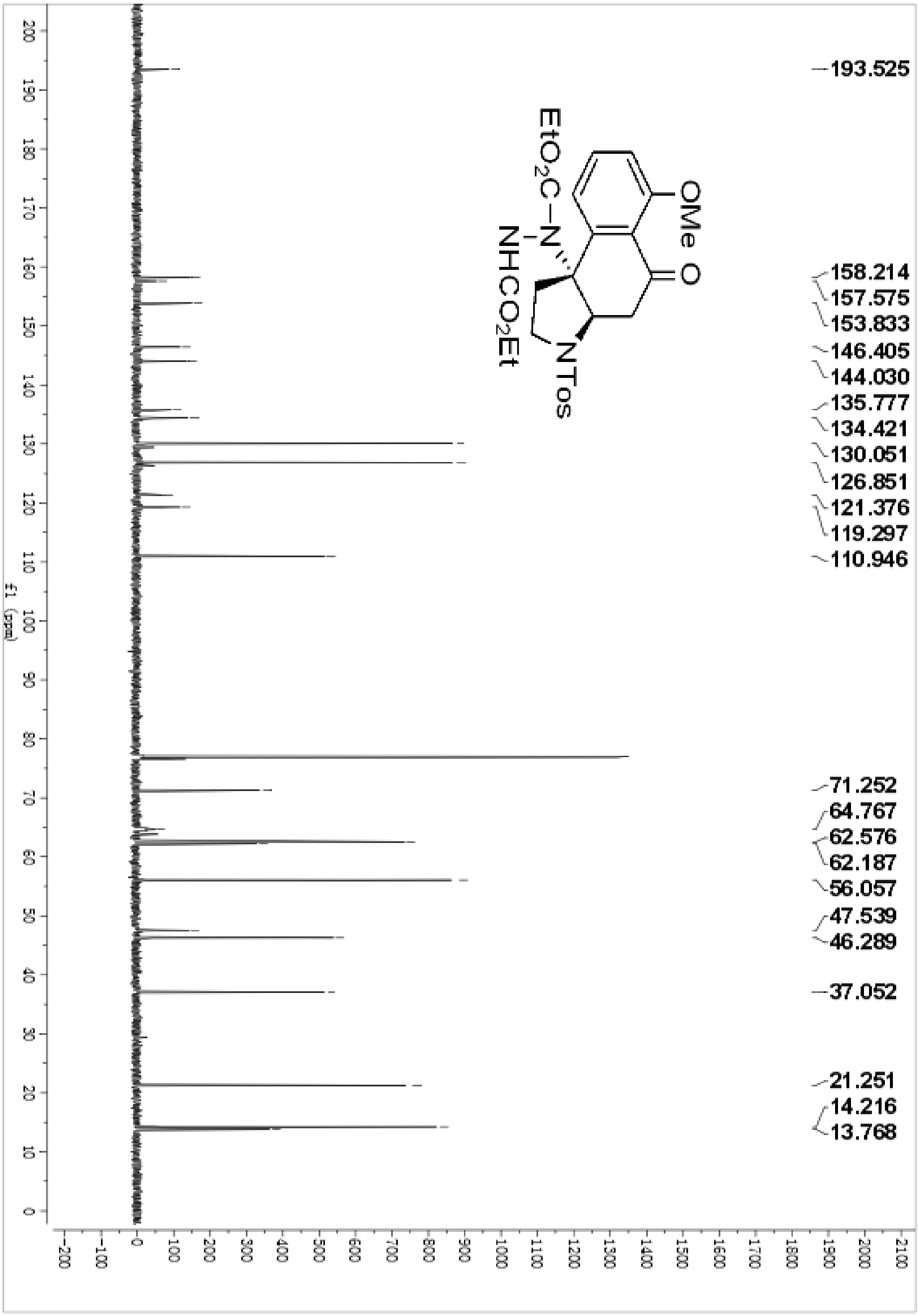

Supplementary Figure 49 ^{1}H NMR of compound **2l** ($CDCl_3$, 600 MHz, 60 °C)

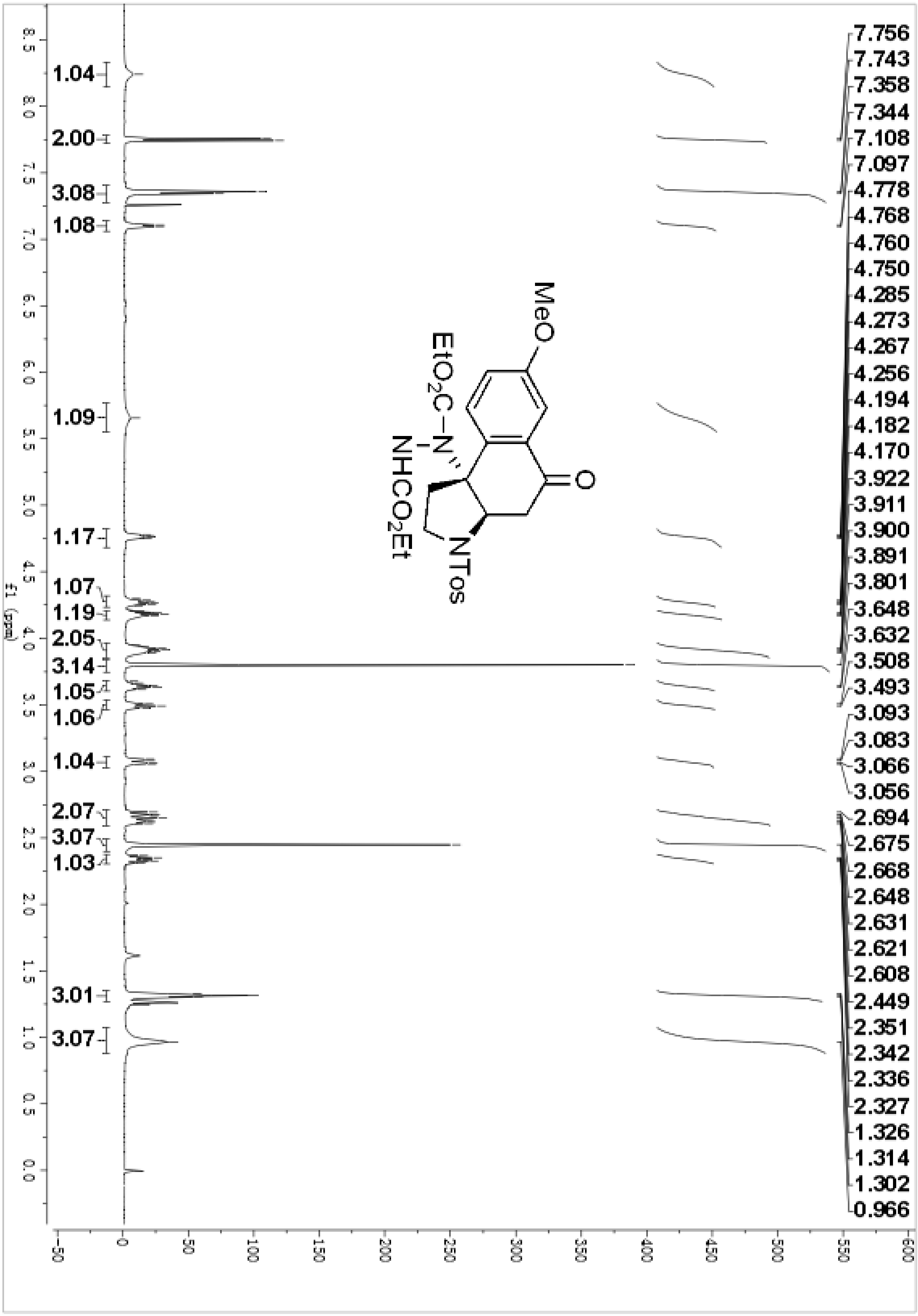

Supplementary Figure 50 ^{13}C NMR of compound **2l** ($CDCl_3$, 150 MHz, 60 °C)

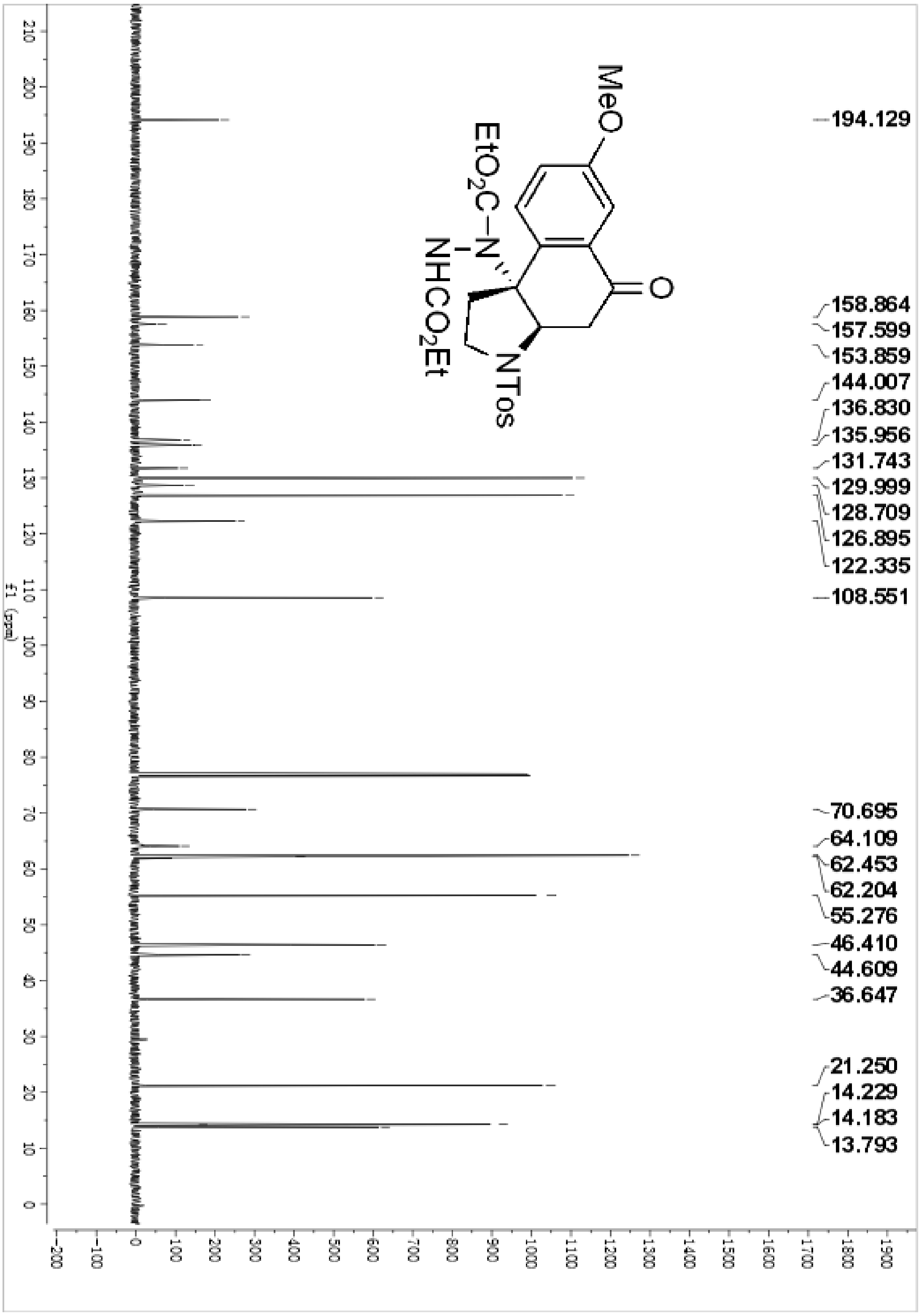

Supplementary Figure 51 ^{1}H NMR of compound **2m** ($CDCl_3$, 600 MHz, 60 °C)

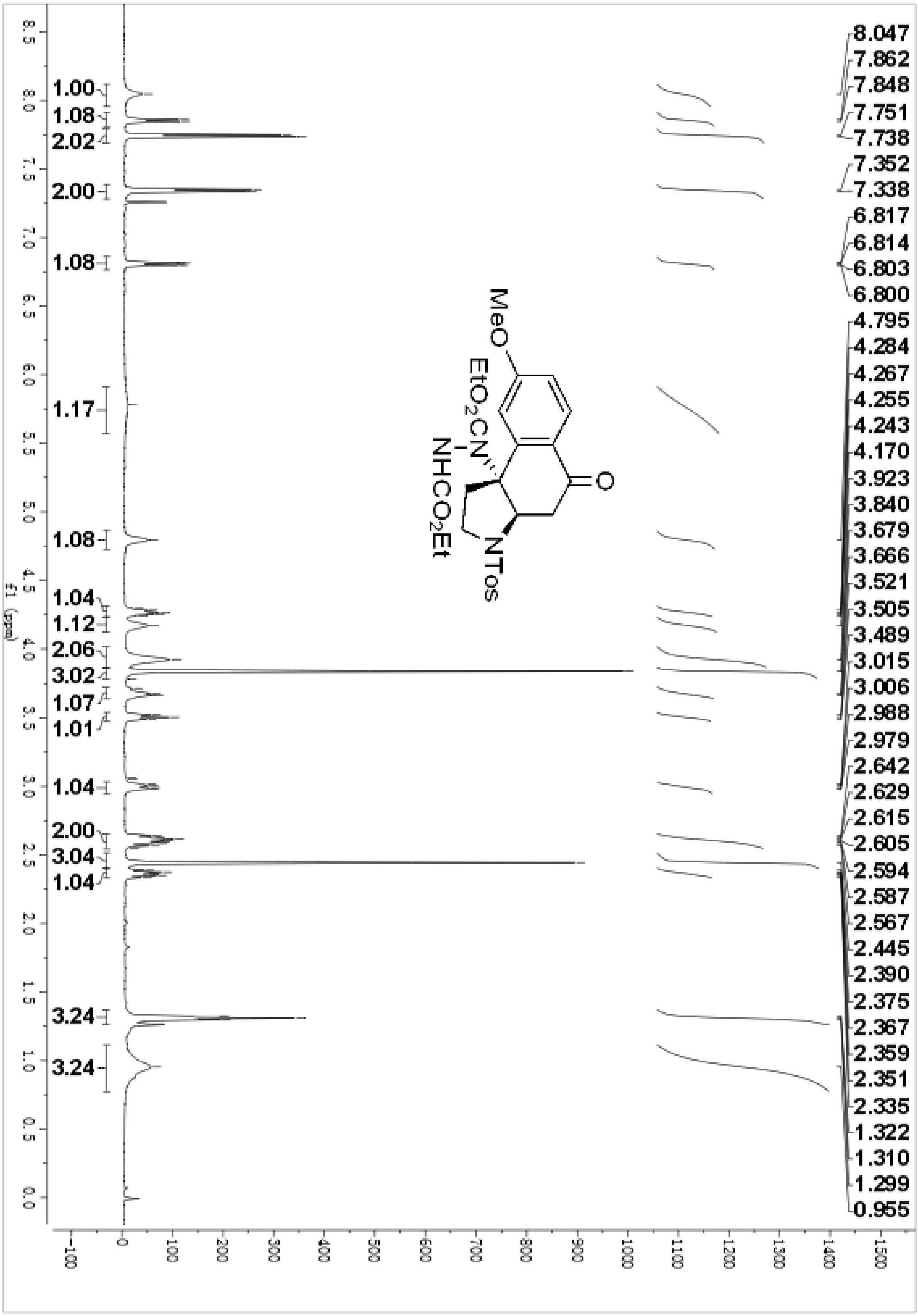

Supplementary Figure 52 ^{13}C NMR of compound **2m** ($CDCl_3$, 150 MHz, 60 °C)

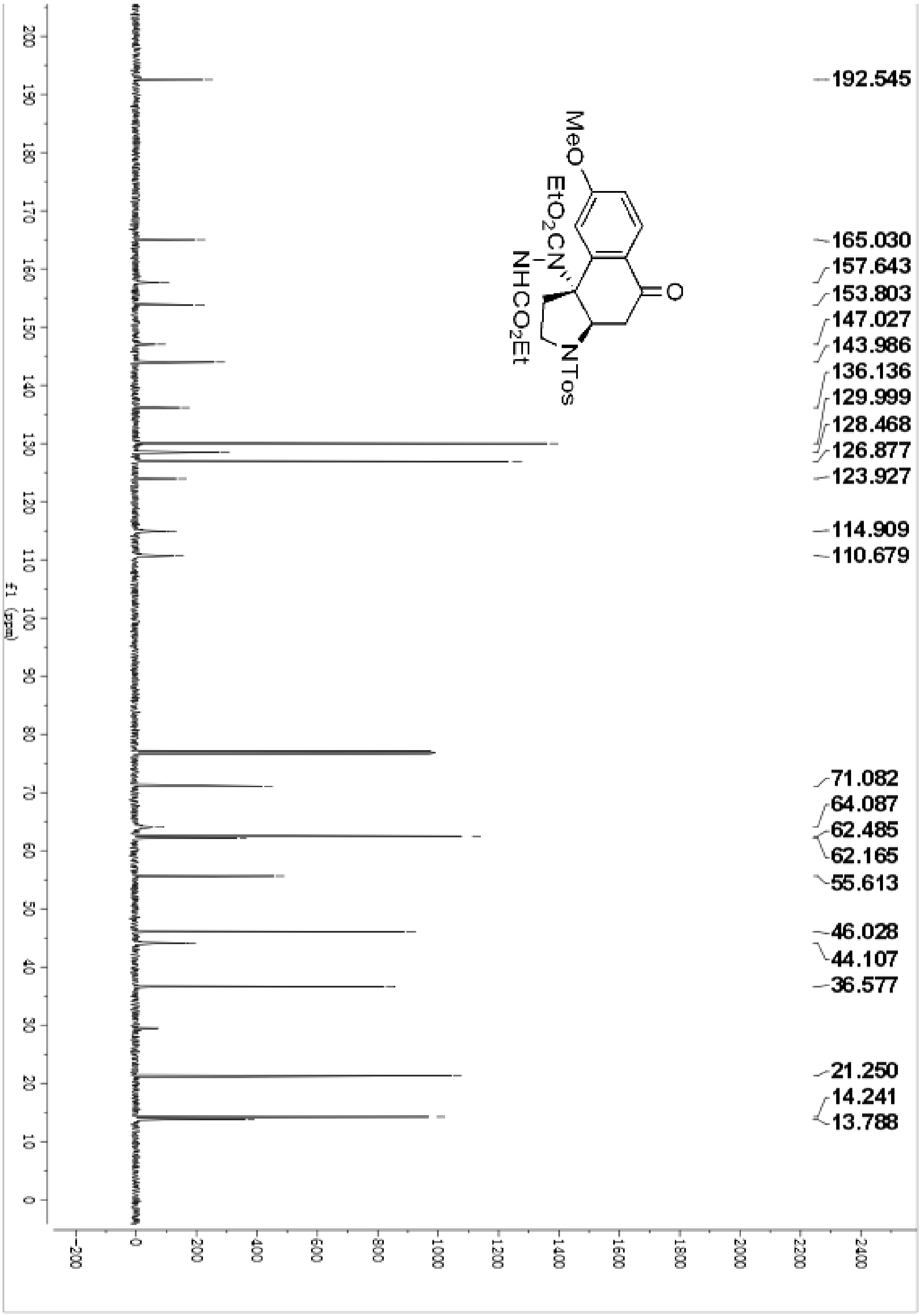

Supplementary Figure 53 ^{1}H NMR of compound **2n** ($CDCl_3$, 600 MHz, 60 °C)

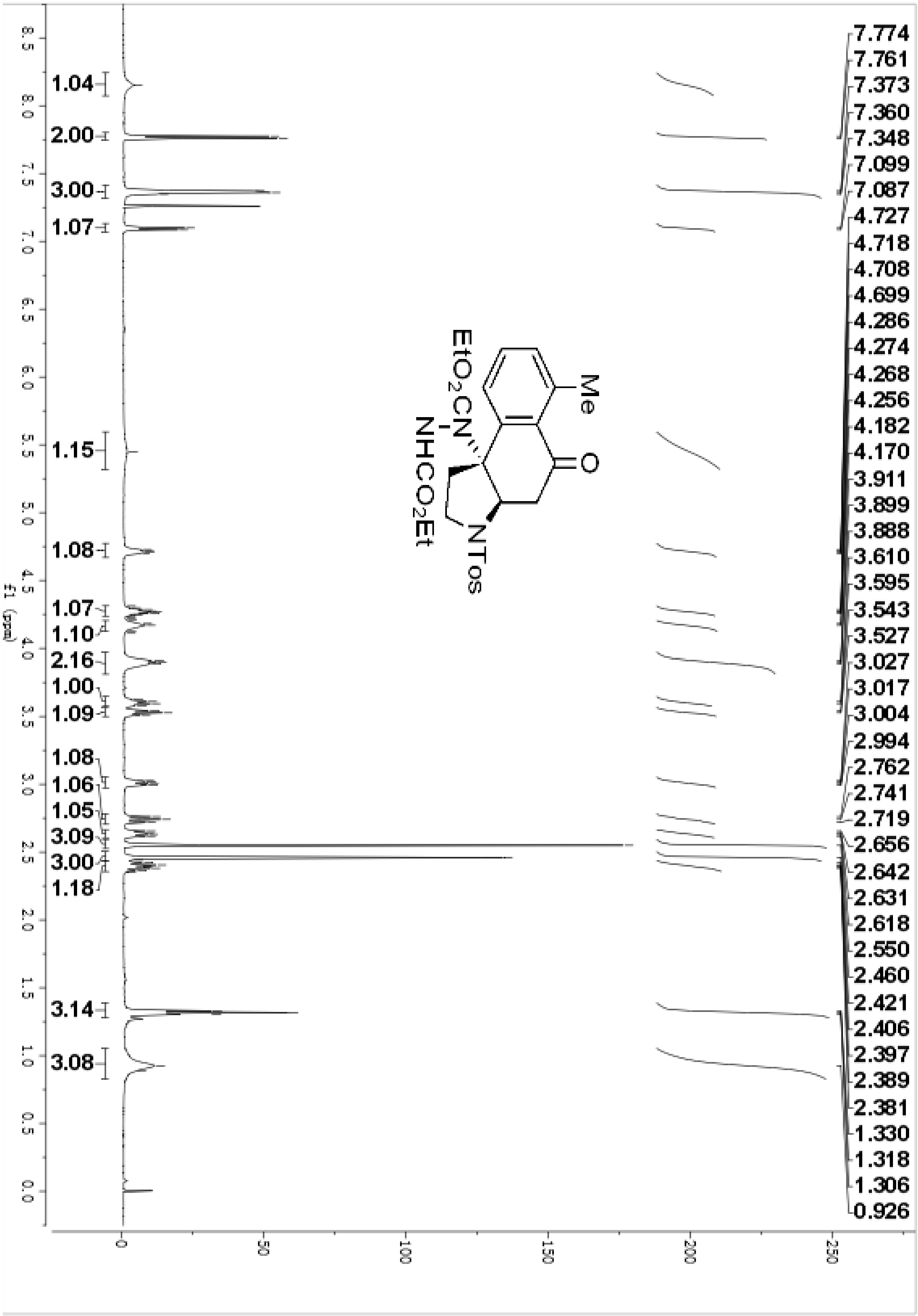

Supplementary Figure 54 ^{13}C NMR of compound **2n** ($CDCl_3$, 150 MHz, 60 °C)

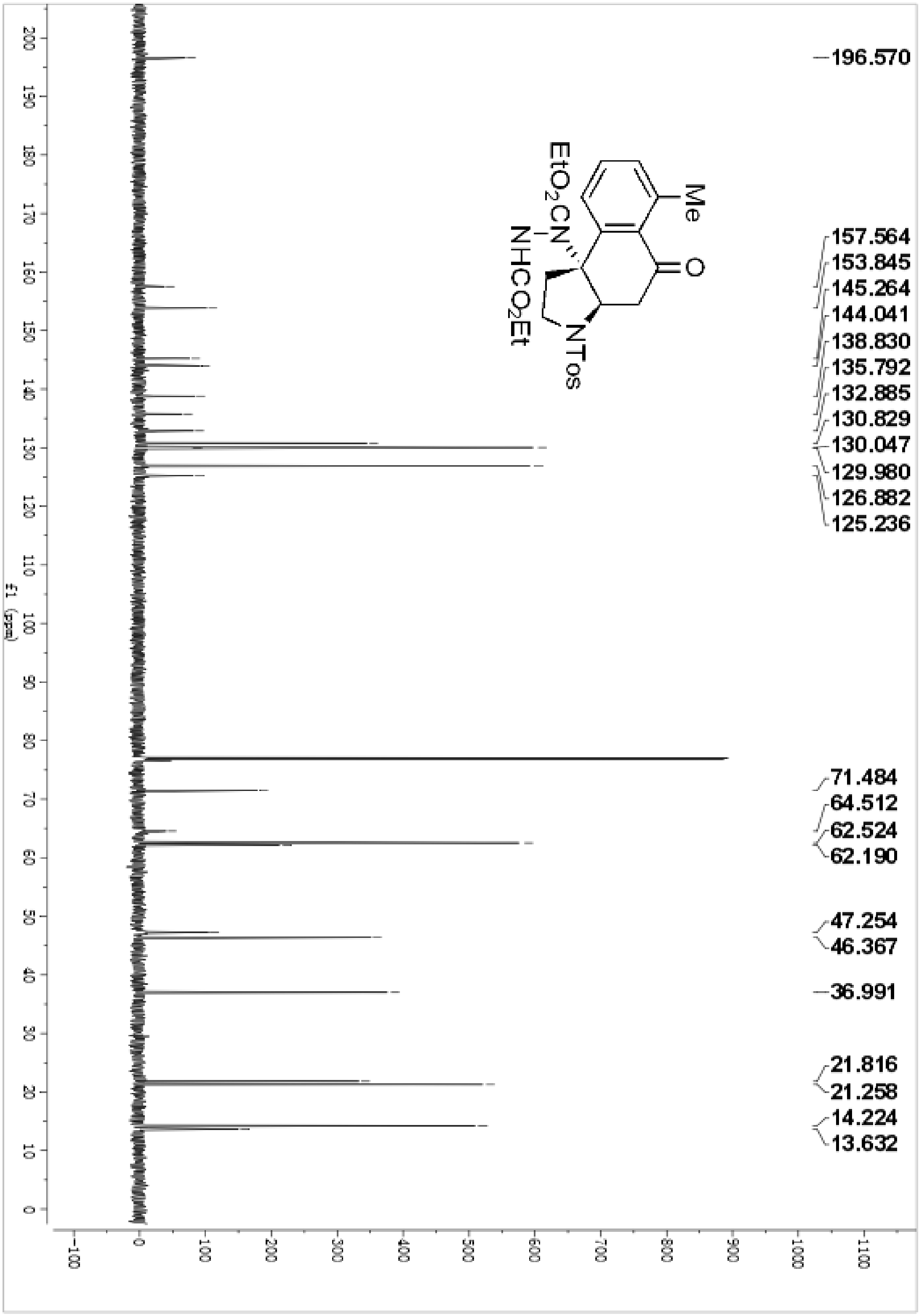

Supplementary Figure 55 ^{1}H NMR of compound **2o** ($CDCl_3$, 600 MHz, 60 °C)

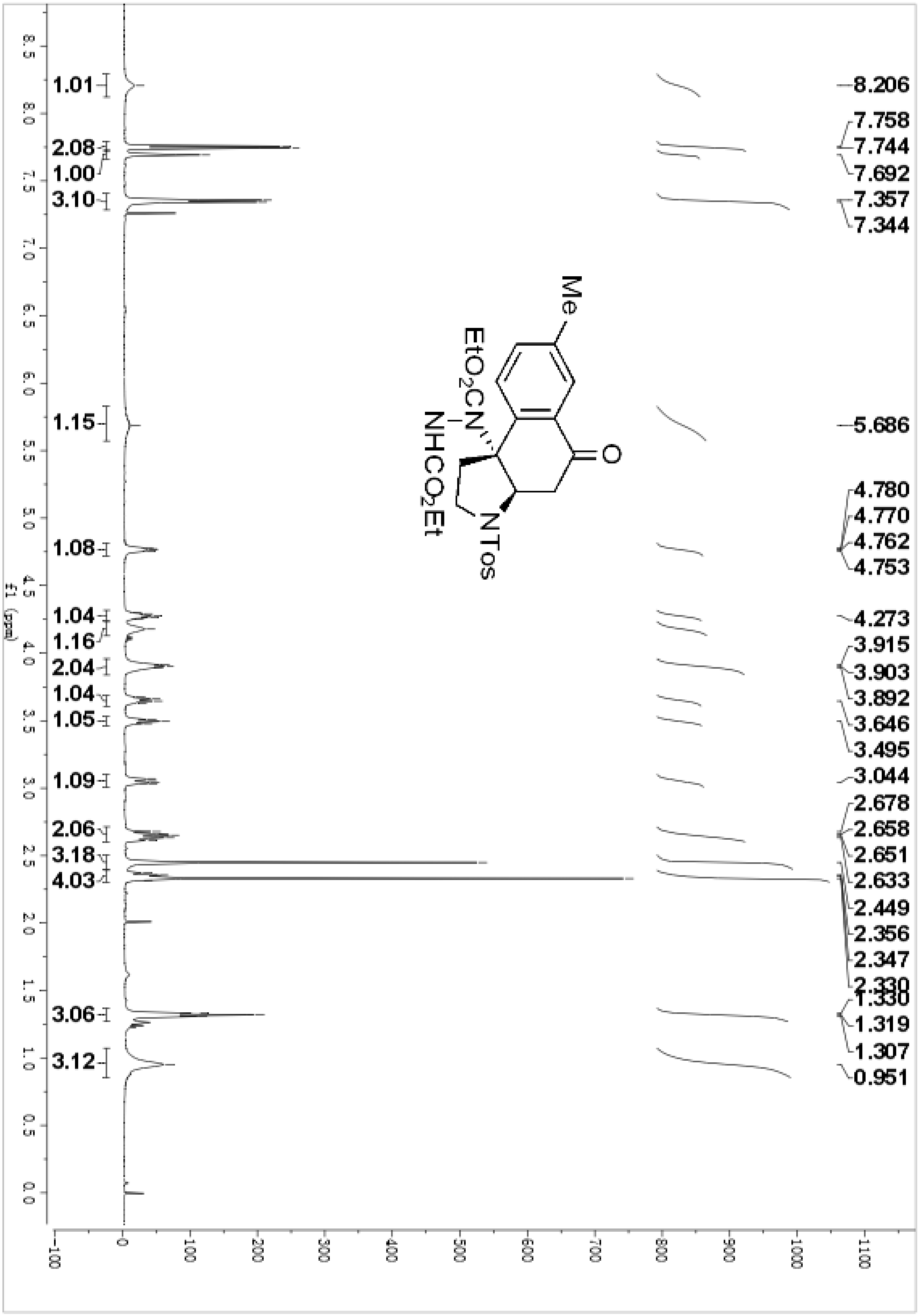

Supplementary Figure 56 ^{13}C NMR of compound **2o** ($CDCl_3$, 150 MHz, 60 °C)

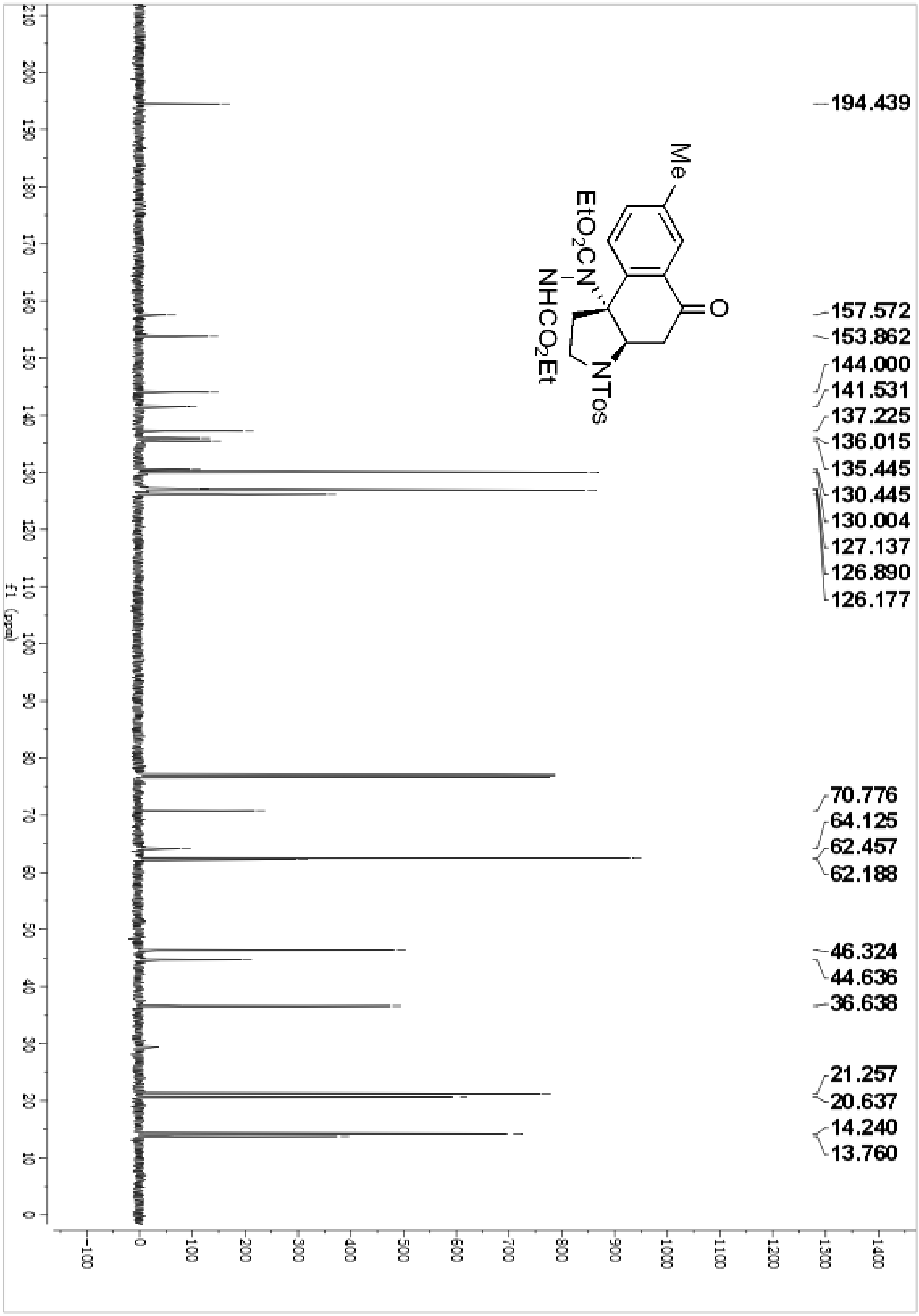

Supplementary Figure 57 ^{1}H NMR of compound **2p** ($CDCl_3$, 600 MHz,60 °C)

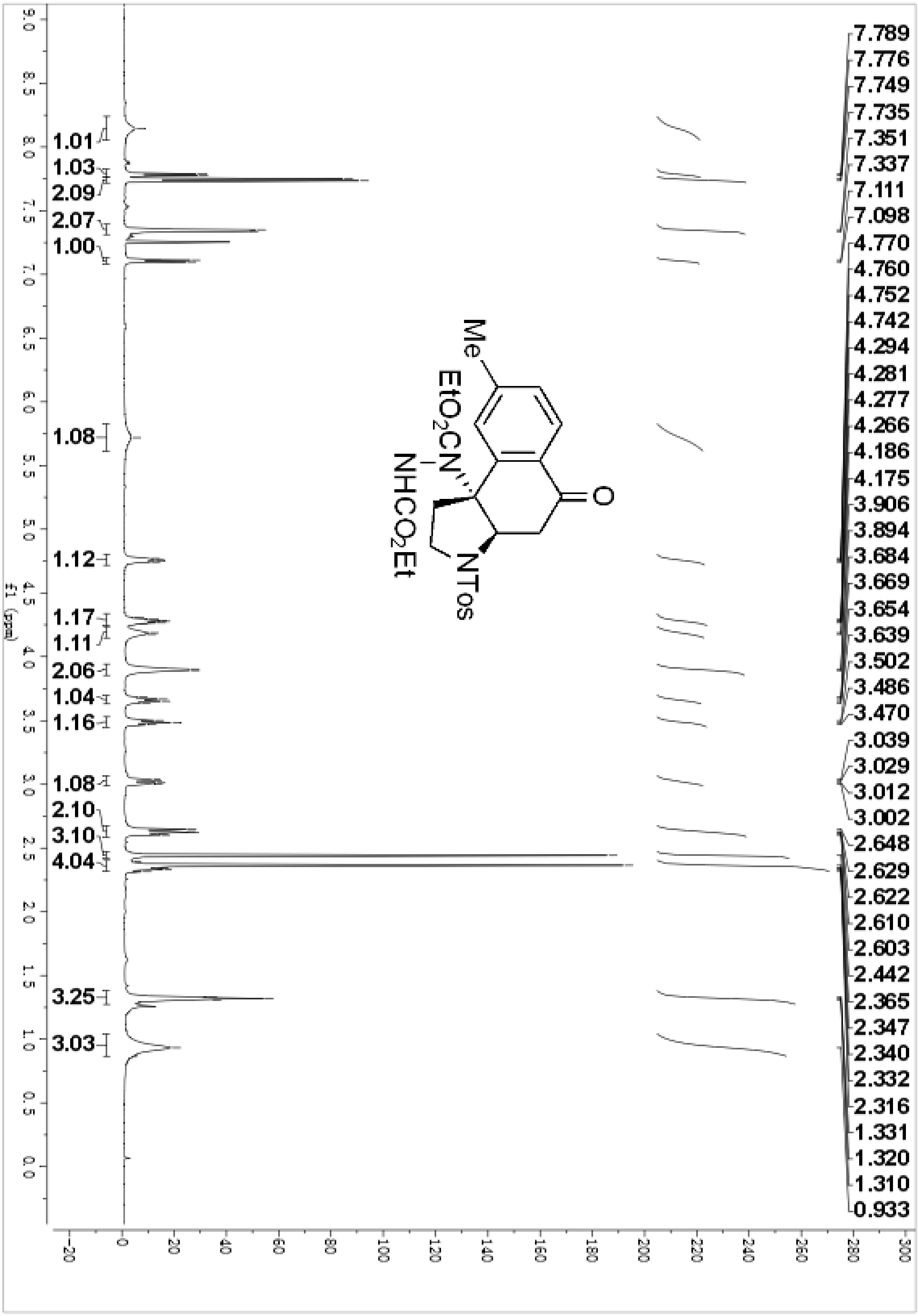

Supplementary Figure 58 ^{13}C NMR of compound **2p** ($CDCl_3$, 150 MHz,60 °C)

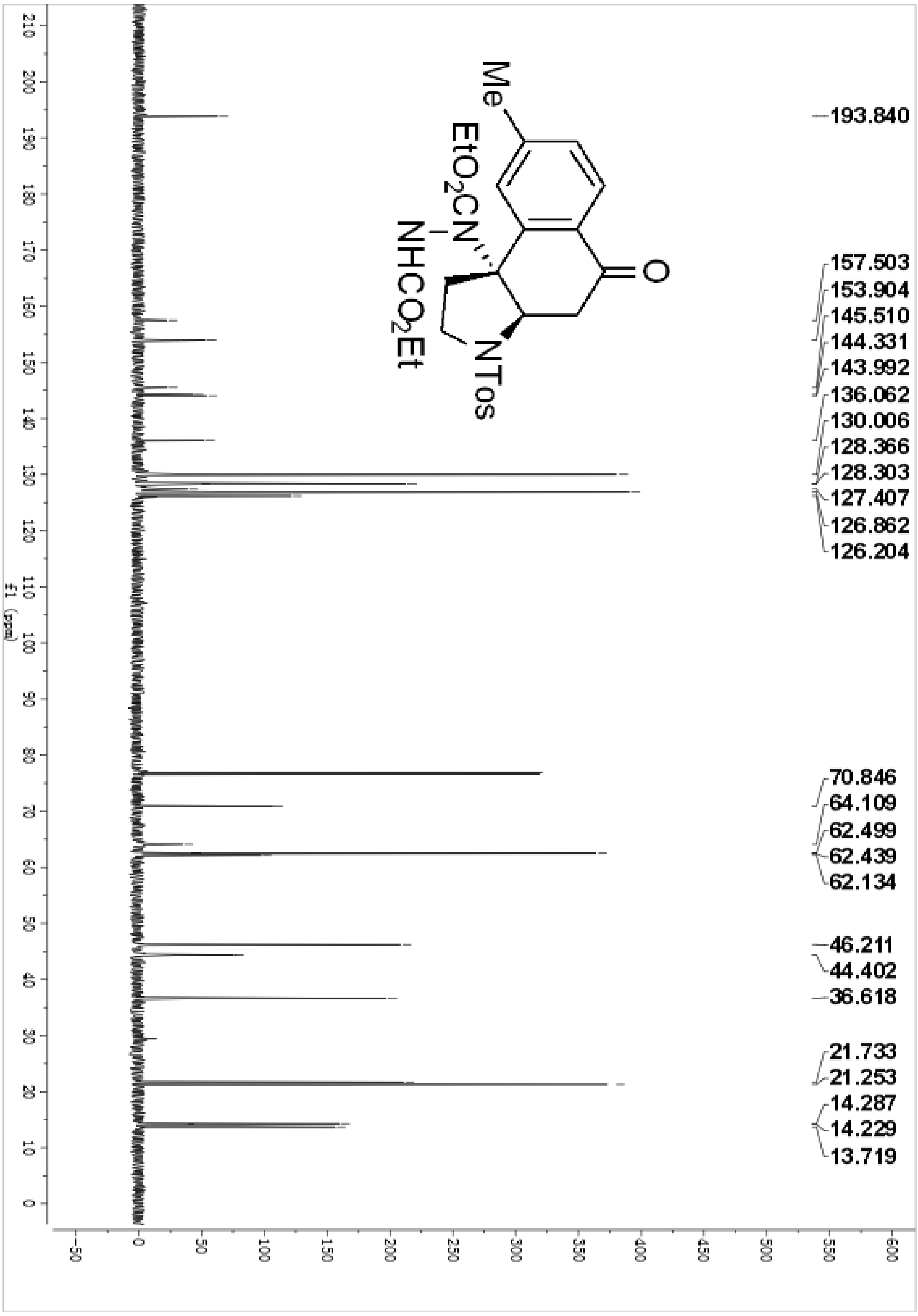

Supplementary Figure 59 ^{1}H NMR of compound **2q** ($CDCl_3$, 600 MHz, 60 °C)

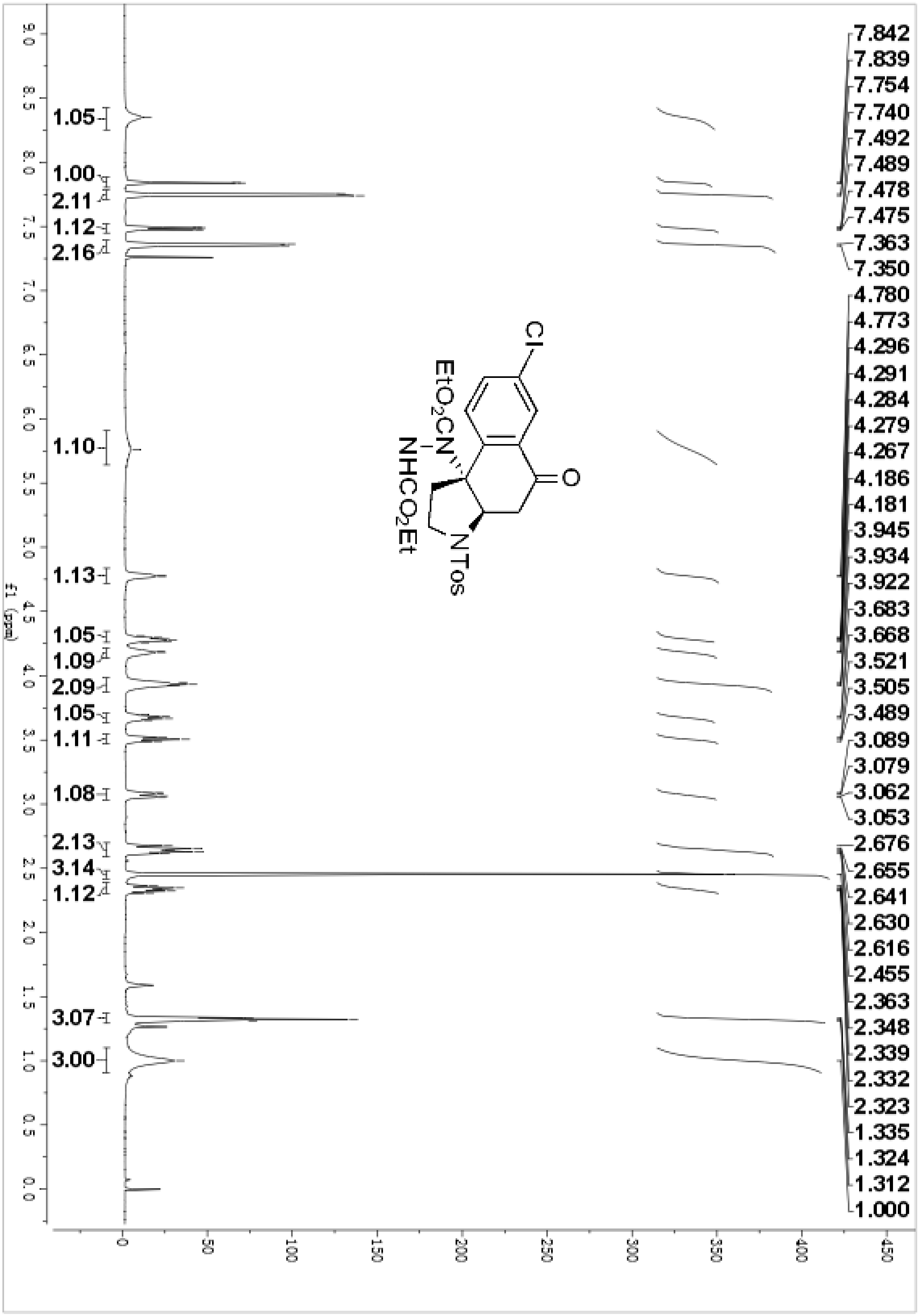

Supplementary Figure 60 ^{13}C NMR of compound **2q** ($CDCl_3$, 150 MHz, 60 °C)

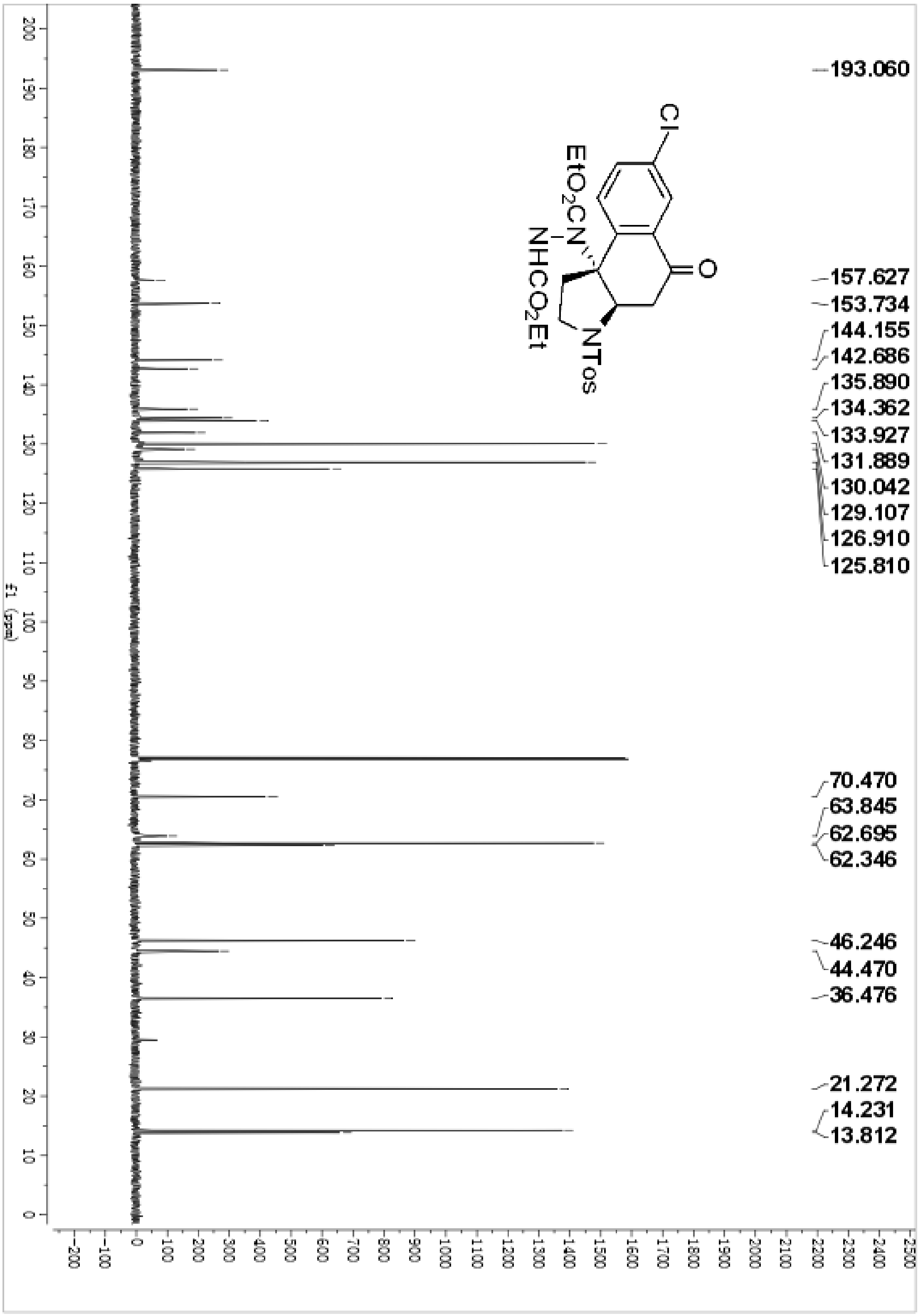

Supplementary Figure 61 ^{1}H NMR of compound **2r** ($CDCl_3$, 600 MHz, 60 °C)

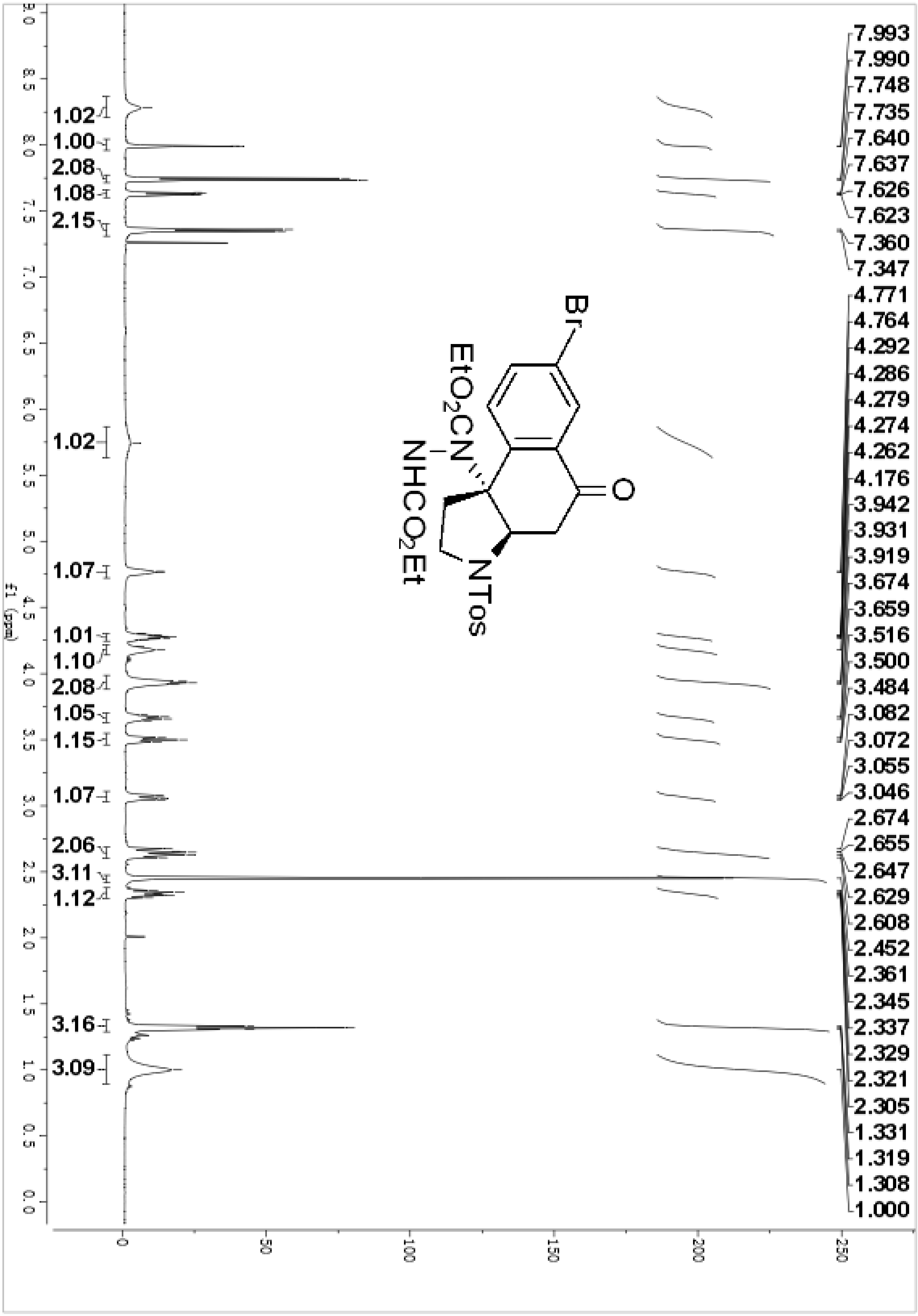

Supplementary Figure 62 ^{13}C NMR of compound **2r** ($CDCl_3$, 150 MHz, 60 °C)

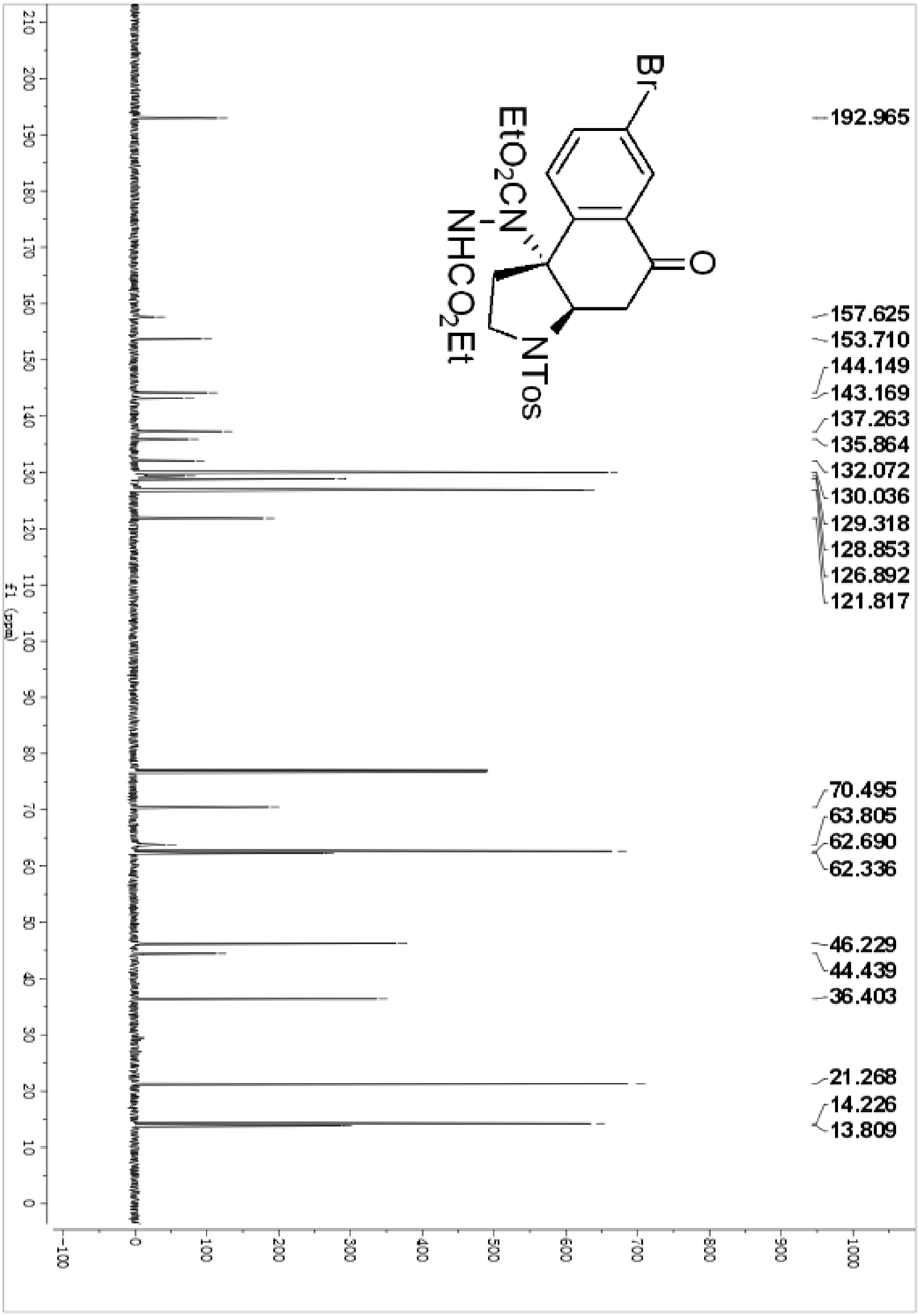

Supplementary Figure 63 ^{1}H NMR of compound **2s** ($CDCl_3$, 600 MHz, 60 °C)

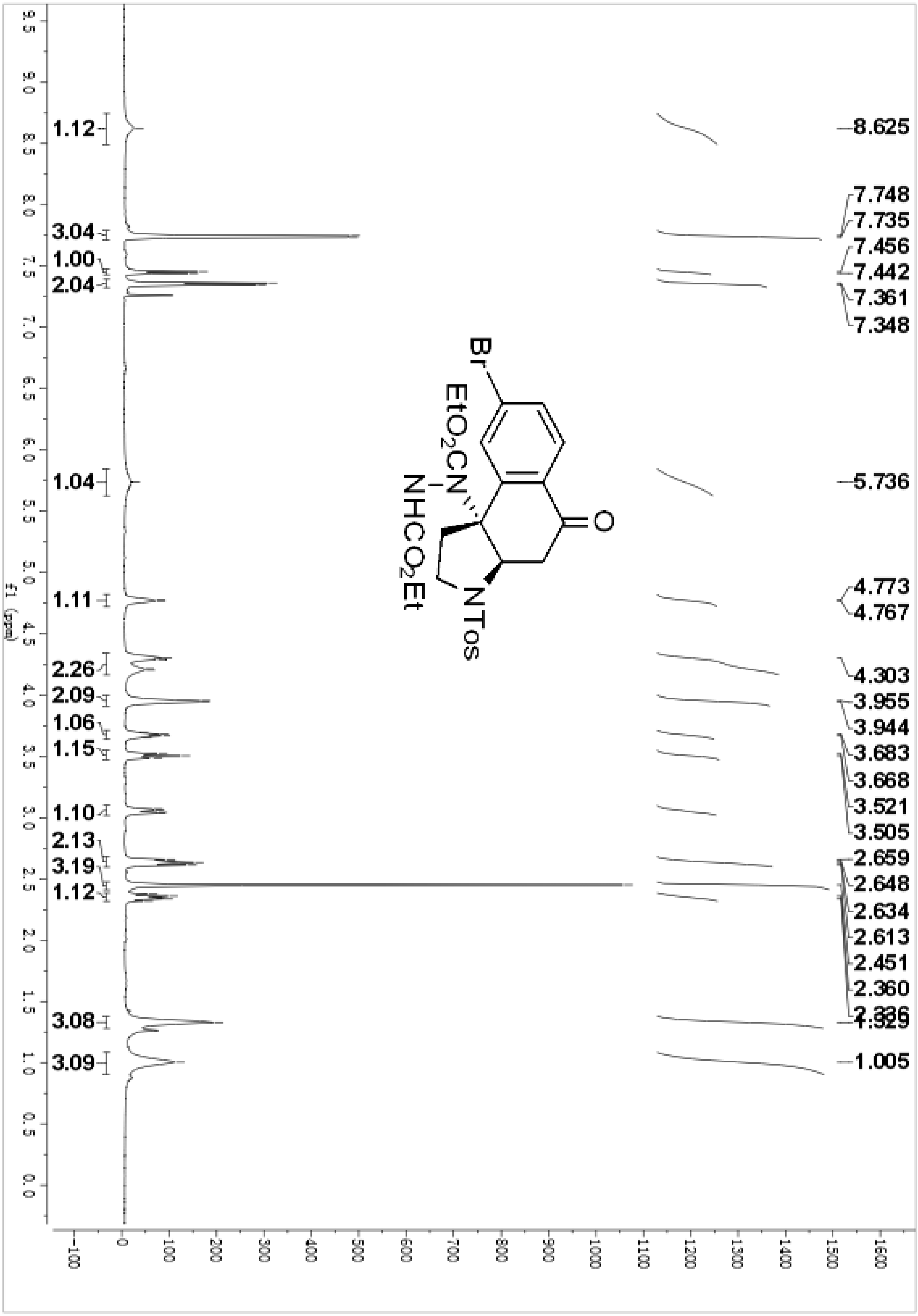

Supplementary Figure 64 ^{13}C NMR of compound **2s** ($CDCl_3$, 150 MHz, 60 °C)

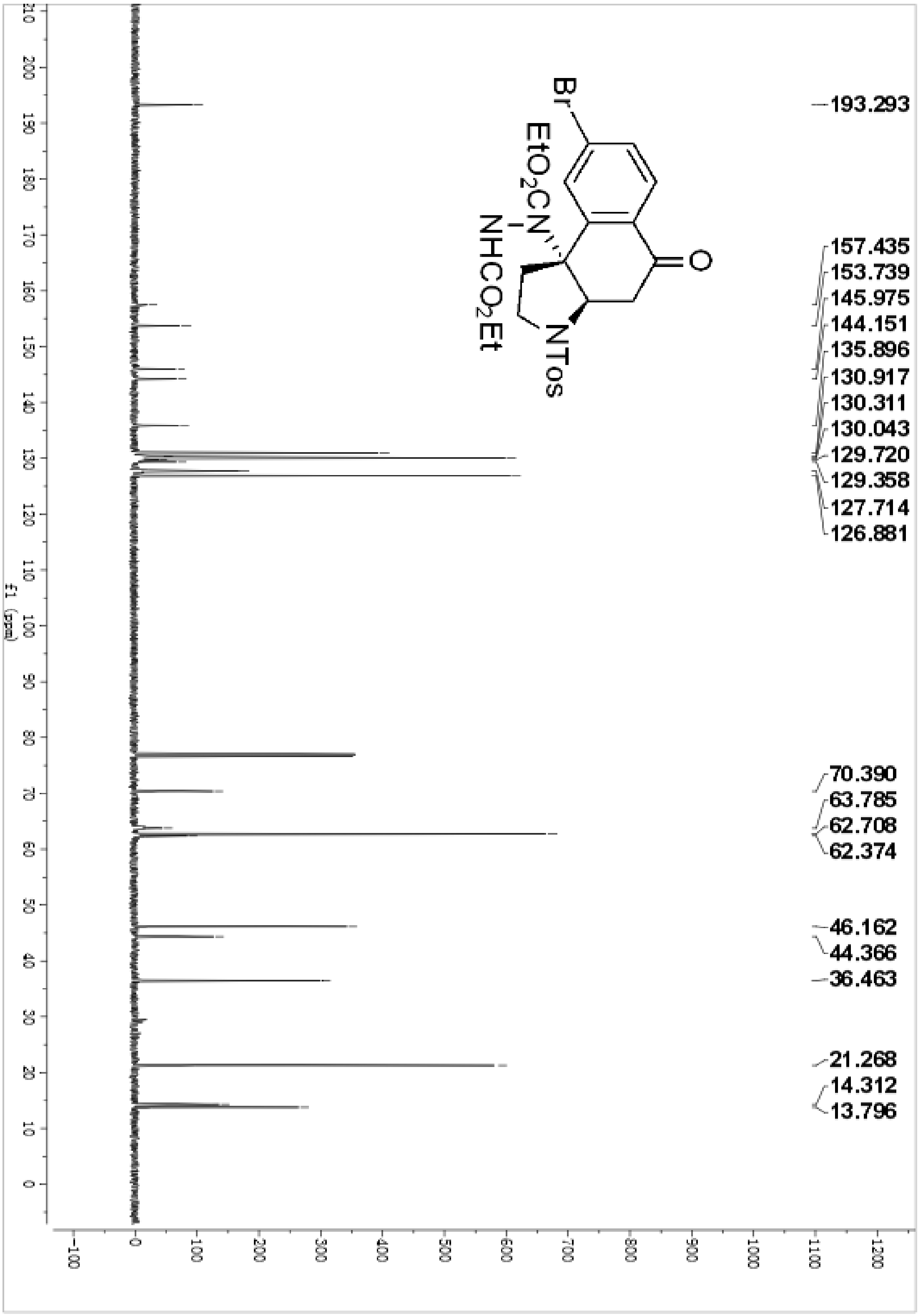

Supplementary Figure 65 ^{1}H NMR of compound **2t** (CD_3CN, 600 MHz, 80 °C)

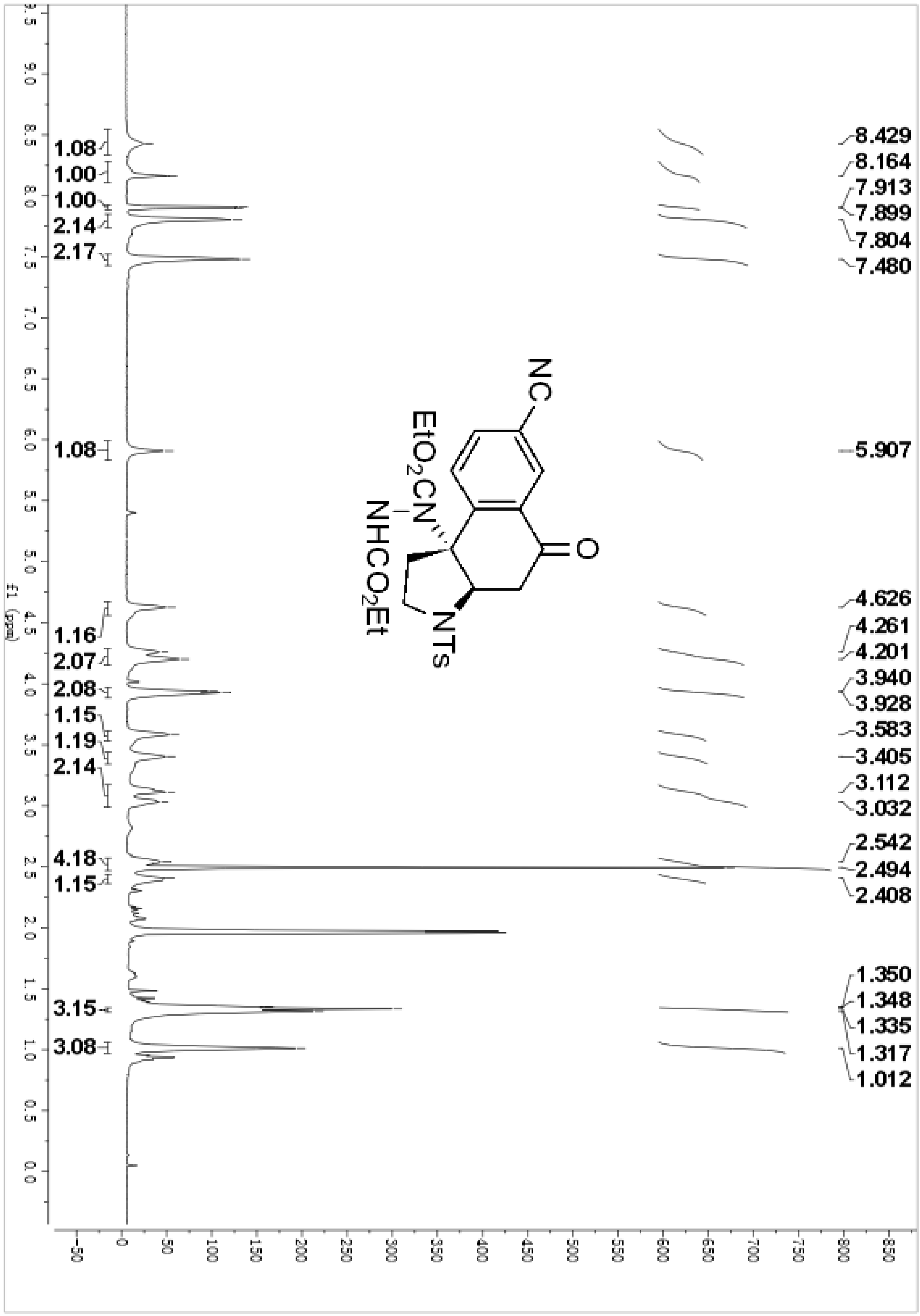

Supplementary Figure 66 ^{13}C NMR of compound **2t** (CD_3CN, 150 MHz, 80 °C)

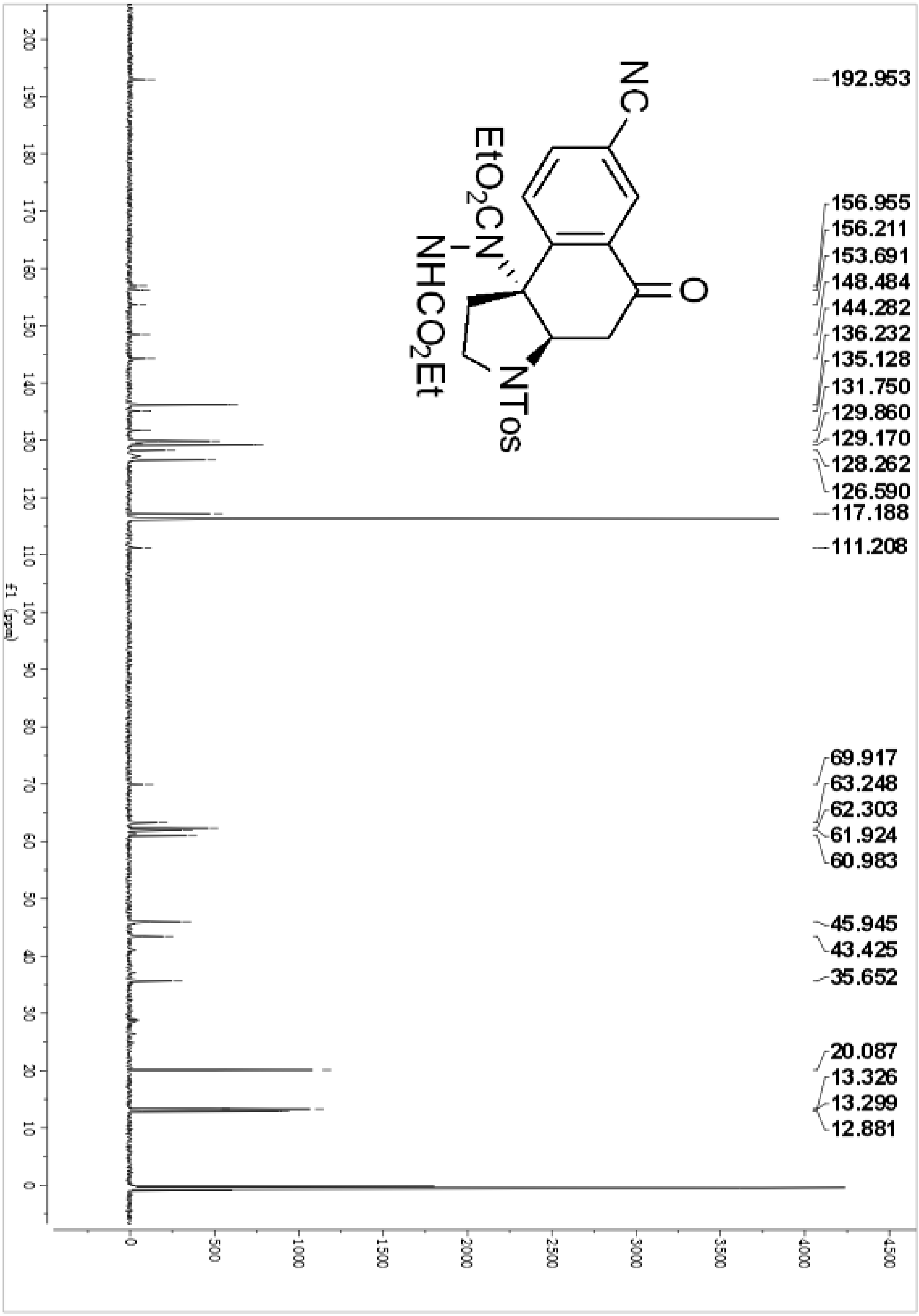

Supplementary Figure 67 ^{1}H NMR of compound **2u** ($CDCl_3$, 600 MHz, 60 °C)

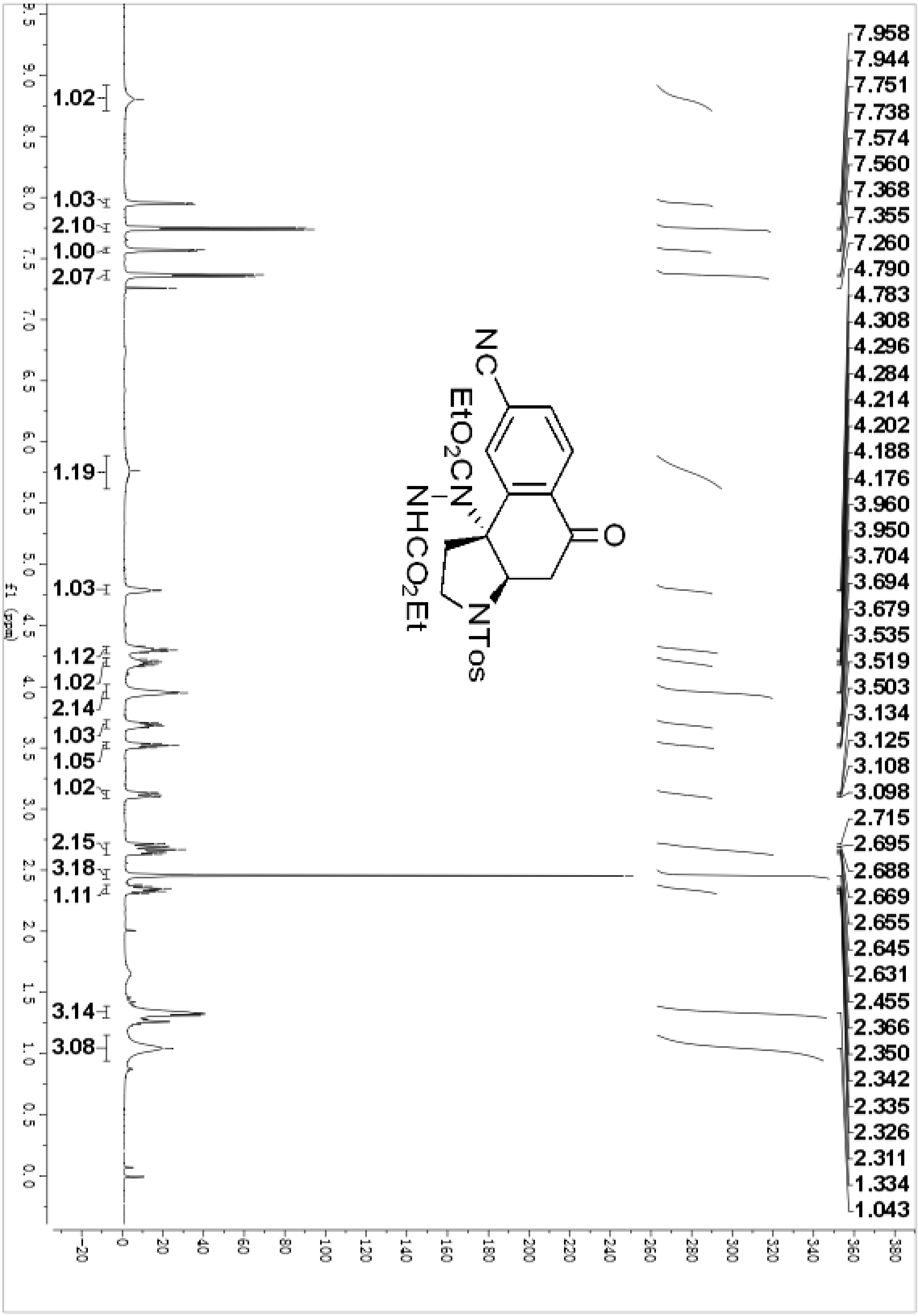

Supplementary Figure 68 ^{13}C NMR of compound **2u** ($CDCl_3$, 150 MHz, 60 °C)

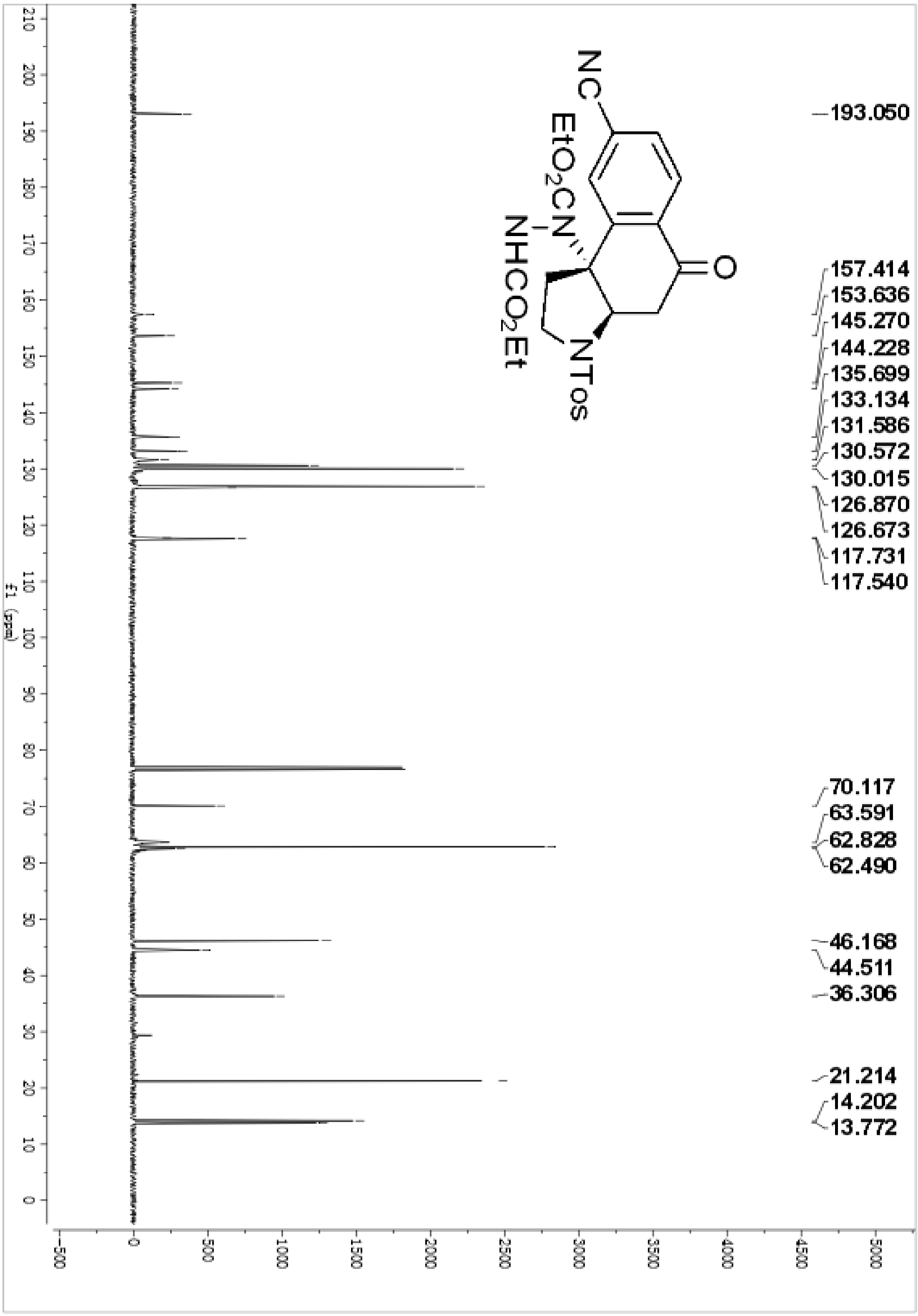

Supplementary Figure 69 ^{1}H NMR of compound **2v** (CD_3CN, 600 MHz, 80 °C)

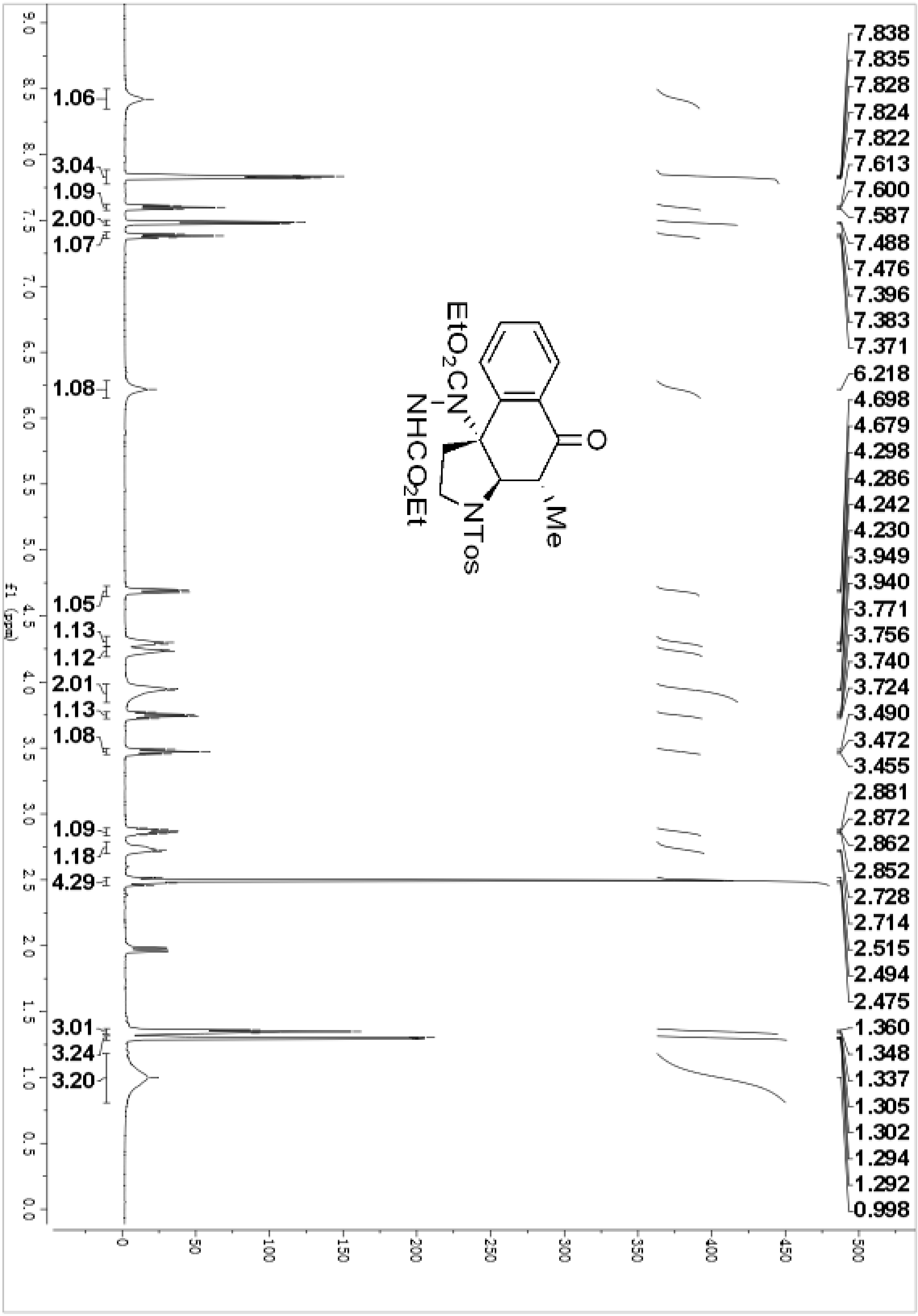

Supplementary Figure 70 ^{13}C NMR of compound **2v** (CD_3CN, 150 MHz, 80 °C)

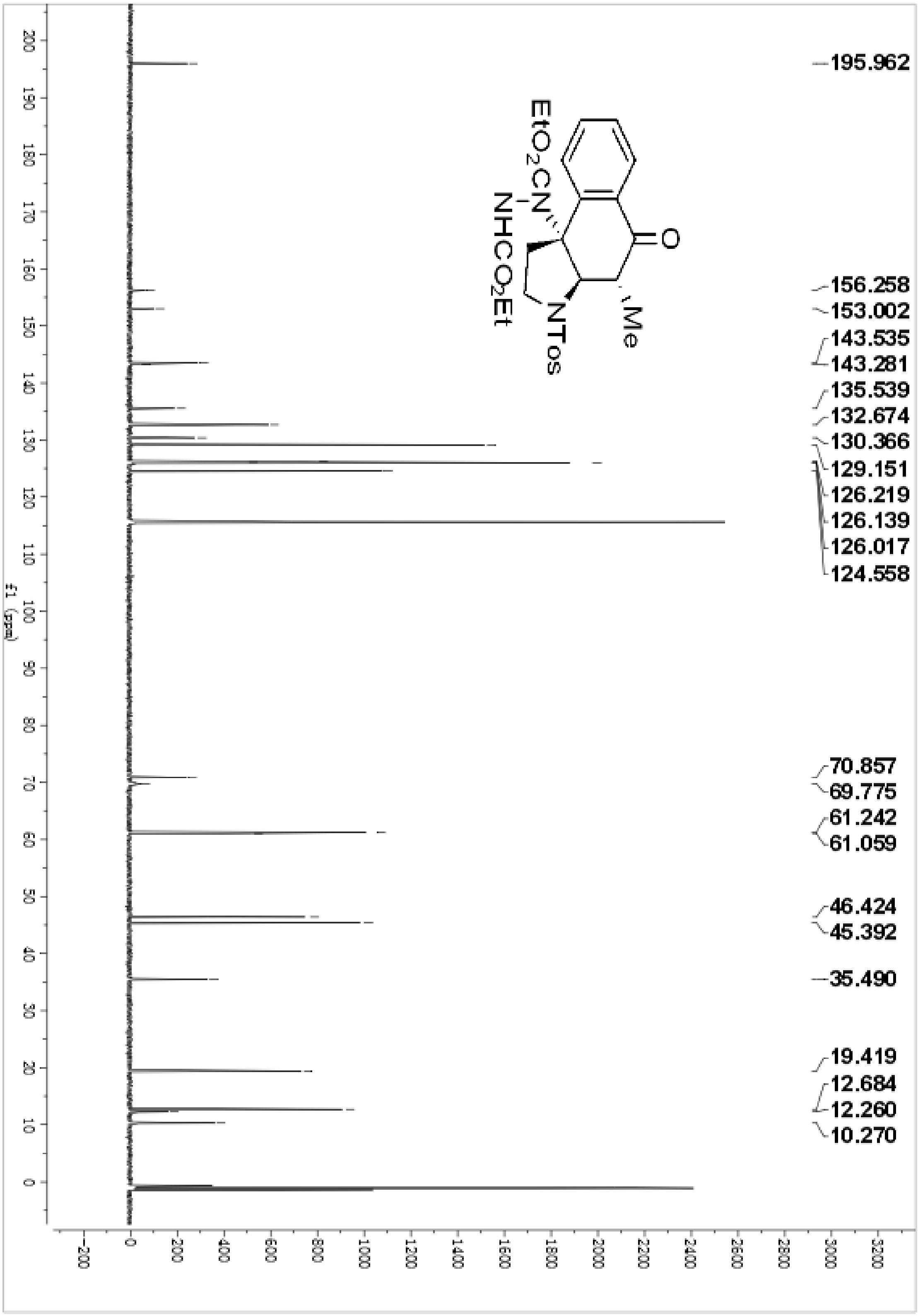

Supplementary Figure 71 COSY of compound **2v** (CD_3CN, 600 MHz, 80 °C)

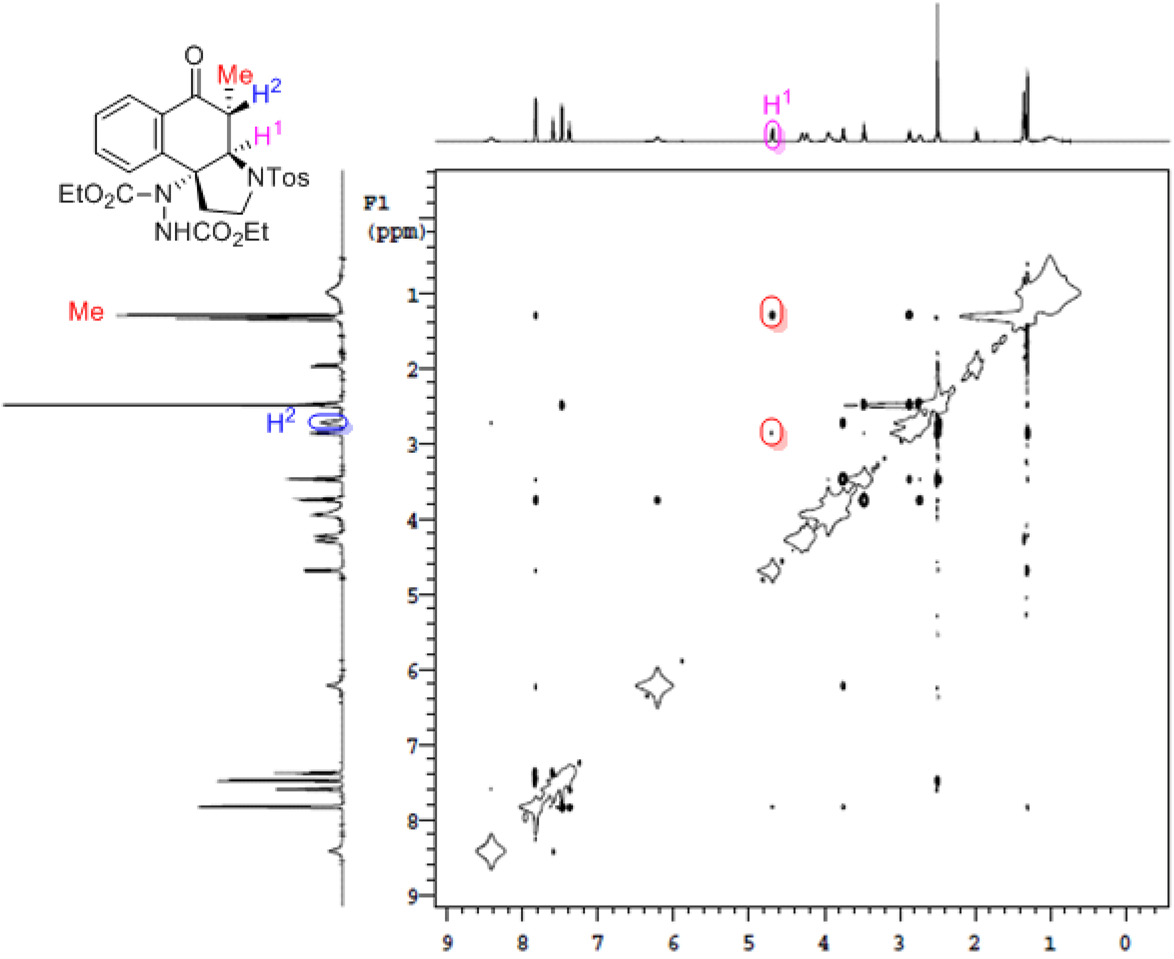

Supplementary Figure 72 ^1H NMR of compound **4a** ($CDCl_3$, 600 MHz, 60 °C)

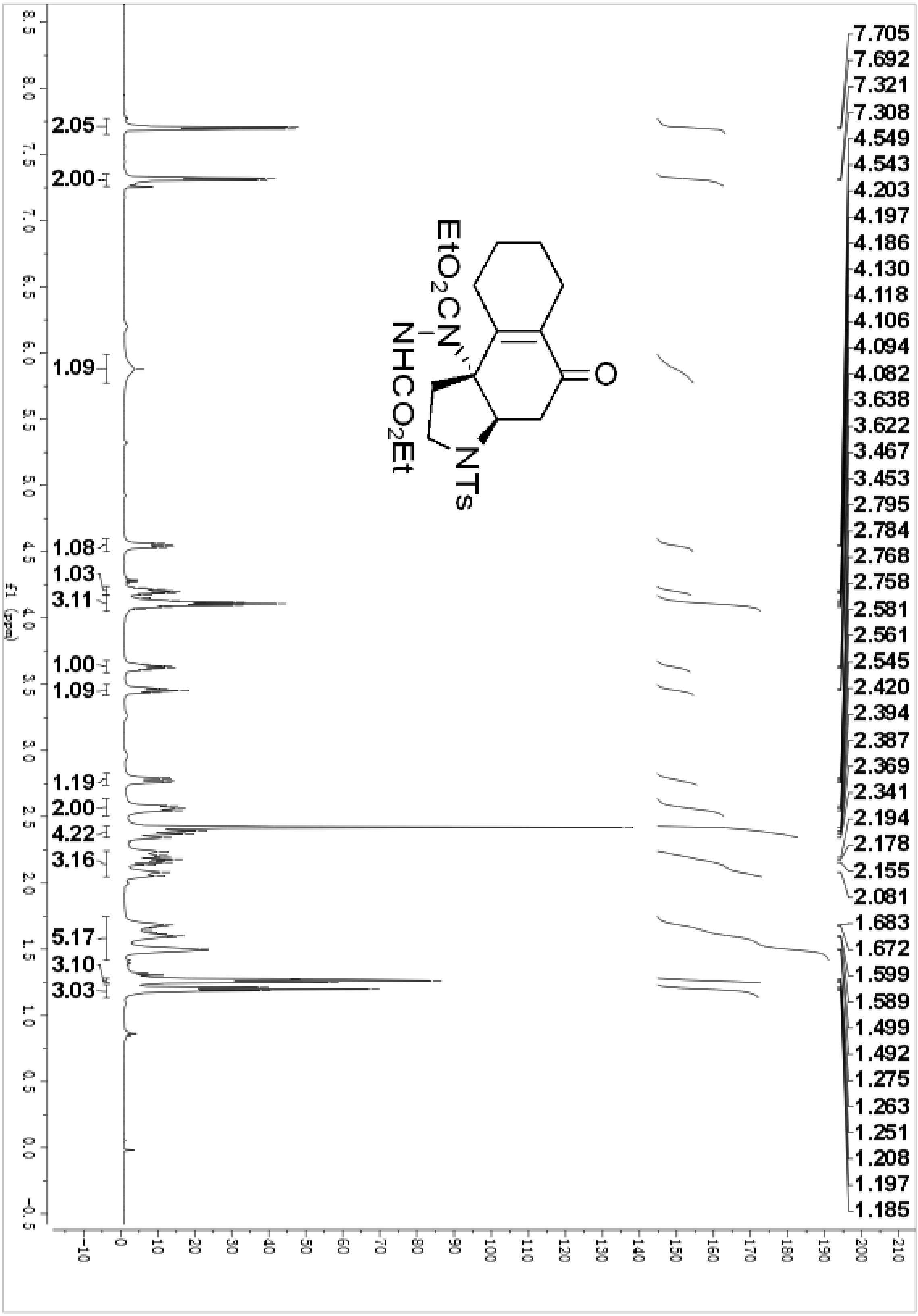

Supplementary Figure 73 ^{13}C NMR of compound **4a** ($CDCl_3$, 150 MHz, 60 °C)

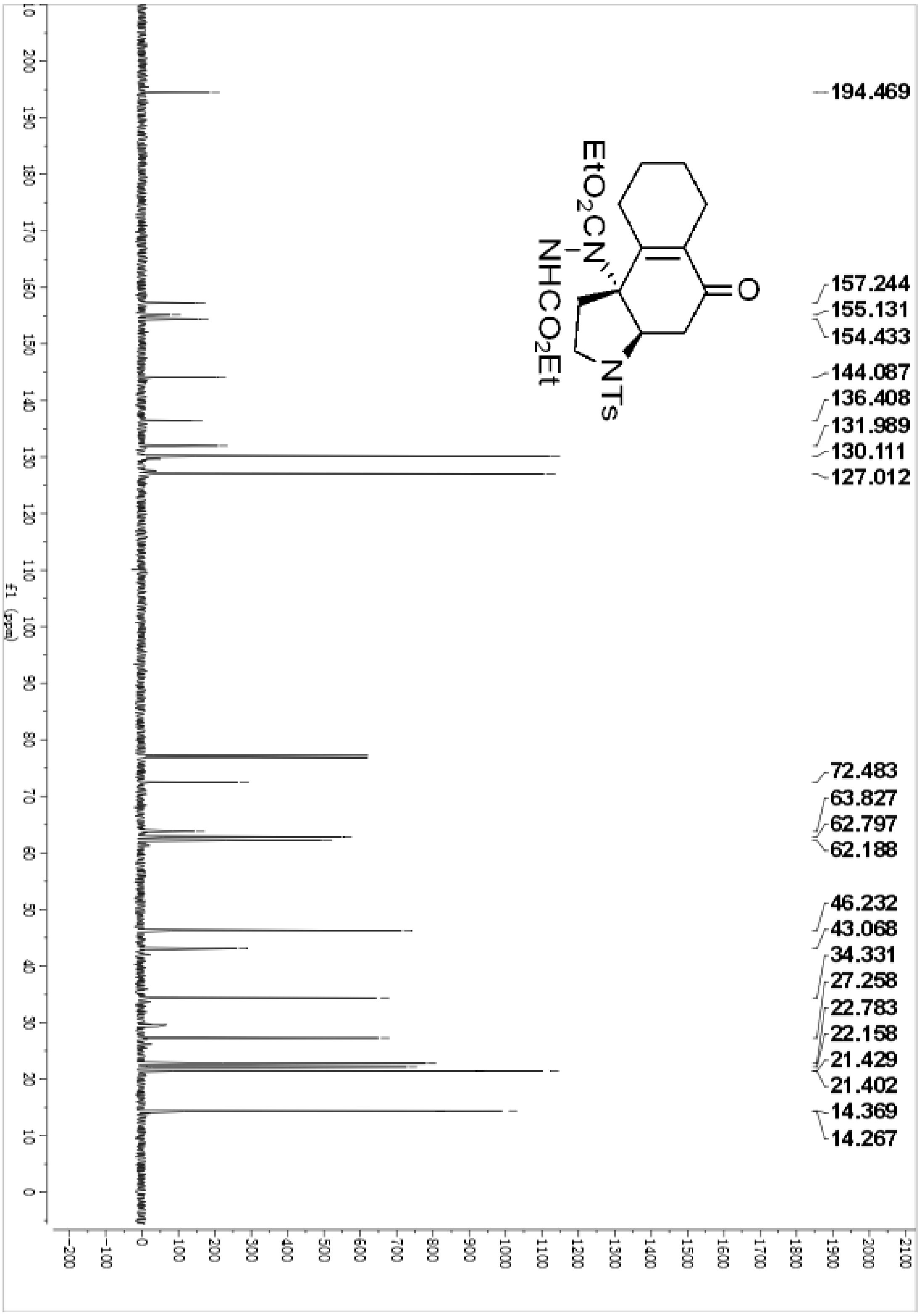

Supplementary Figure 74 ^{1}H NMR of compound **4b** ($CDCl_3$, 600 MHz, 60 °C)

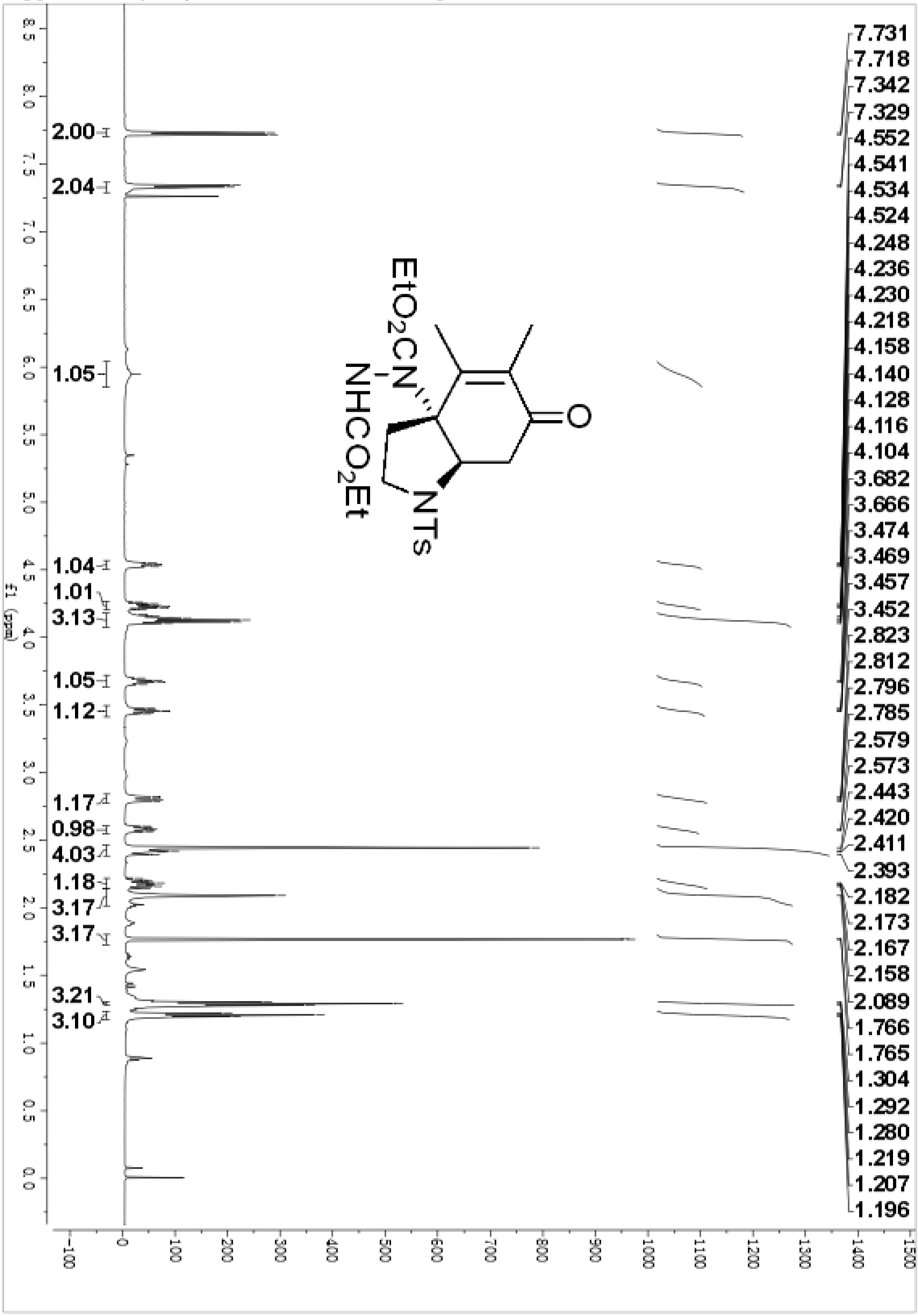

Supplementary Figure 75 ^{13}C NMR of compound **4b** ($CDCl_3$, 150 MHz, 60 °C)

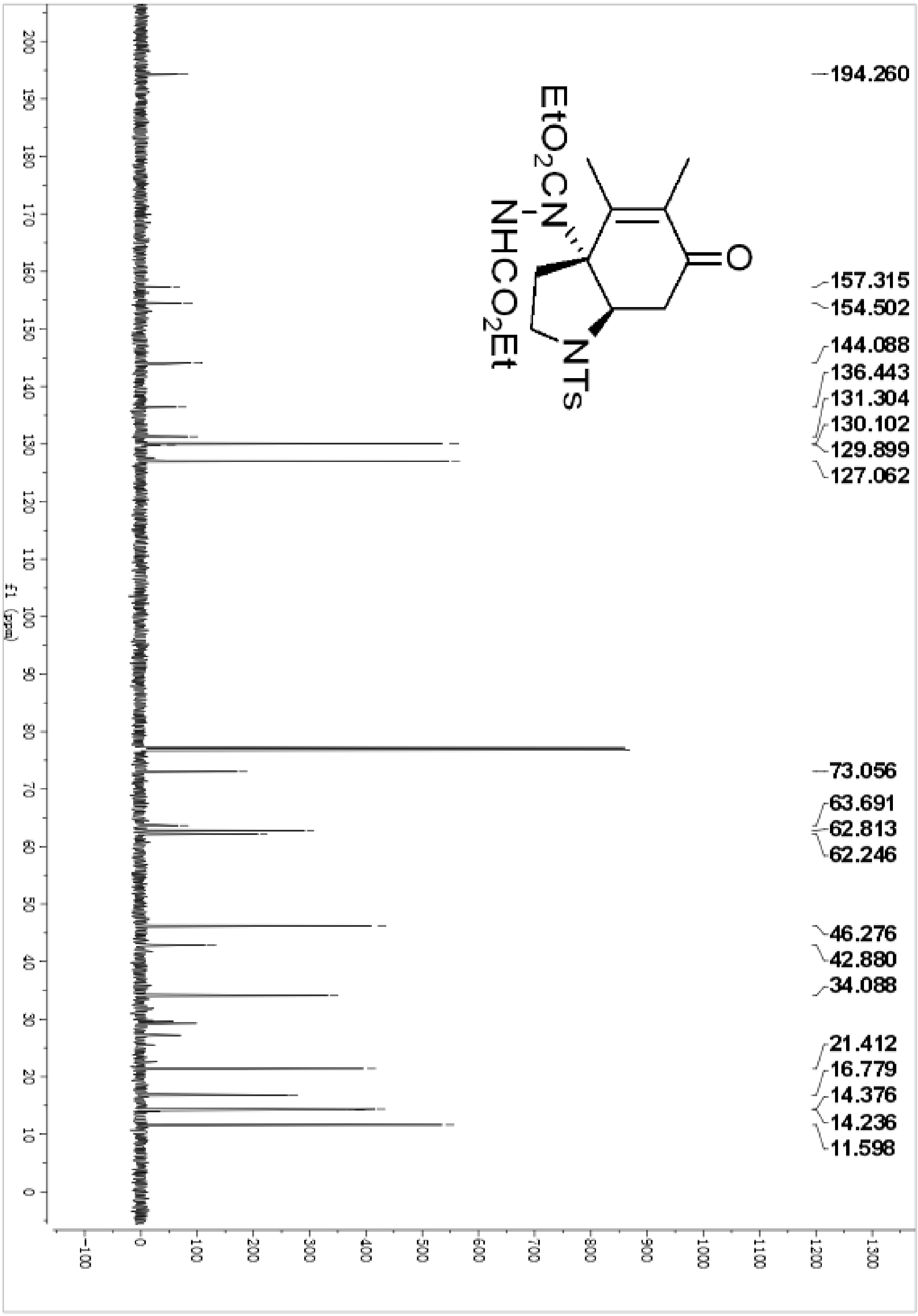

Supplementary Figure 76 ^{1}H NMR of compound **5** ($CDCl_3$, 600 MHz, 60 °C)

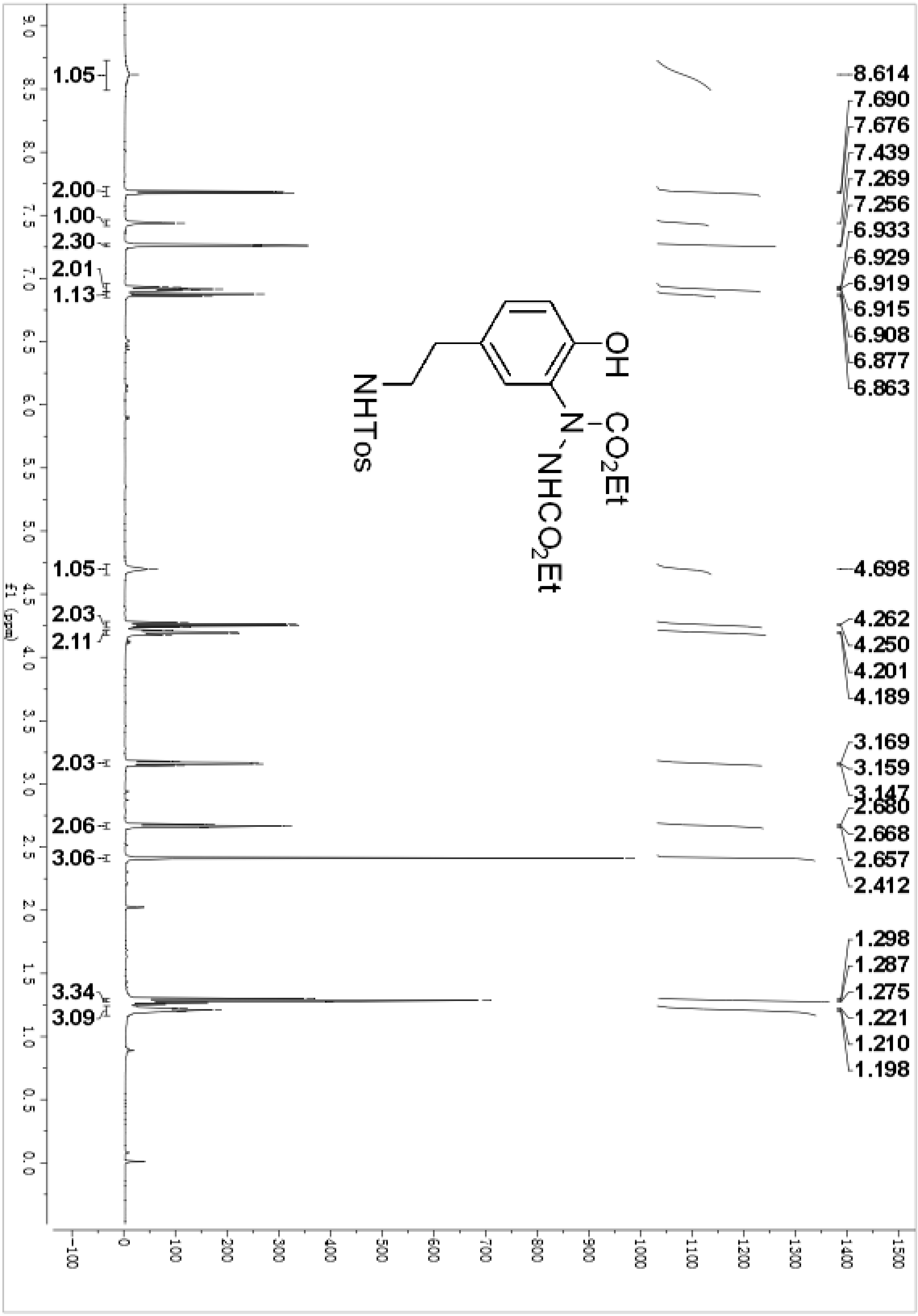

Supplementary Figure 77 ^{13}C NMR of compound **5** ($CDCl_3$, 150 MHz, 60 °C)

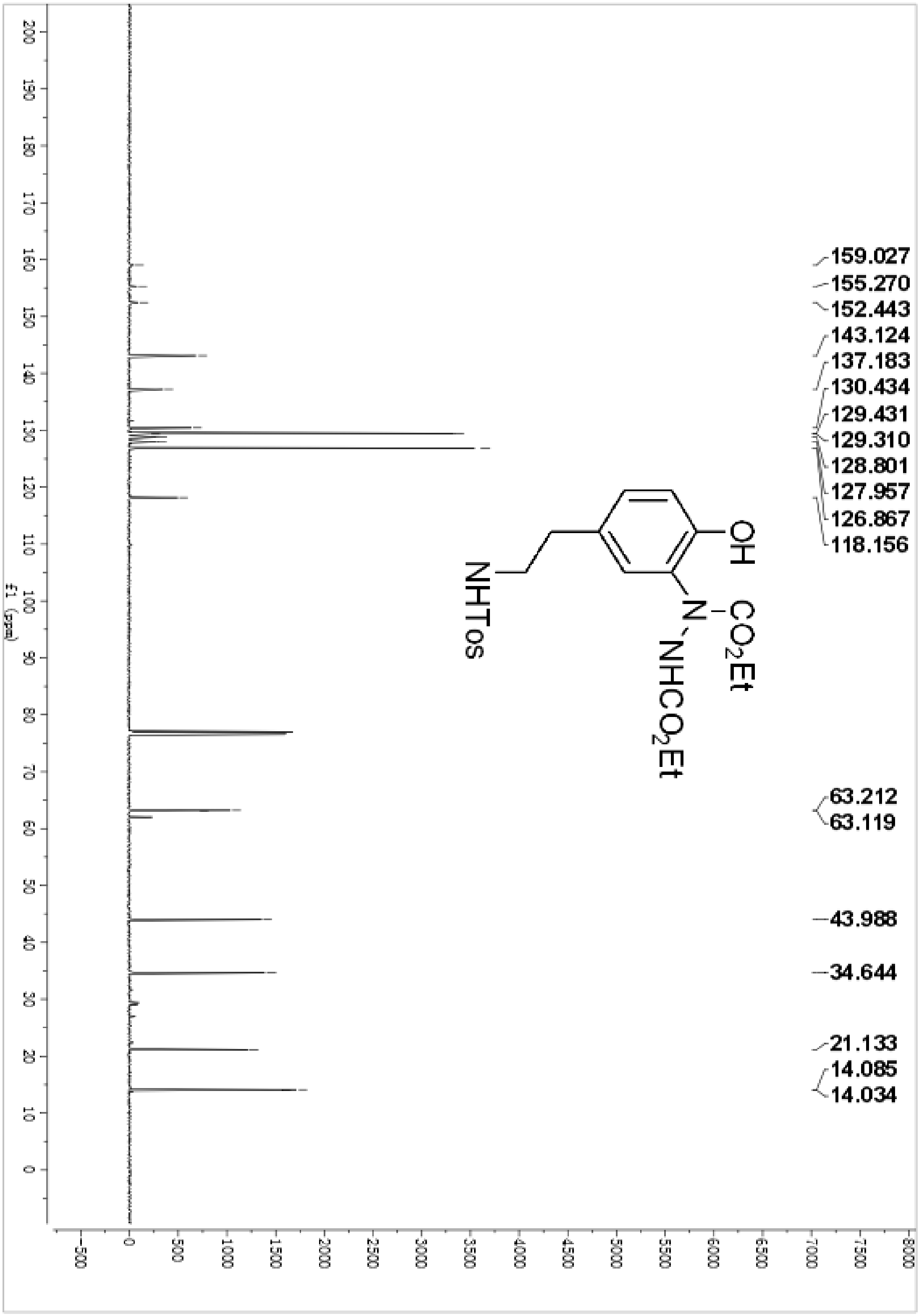

Supplementary Figure 78 ^{1}H NMR of compound **6** (CD_3CN, 600 MHz, 80 °C)

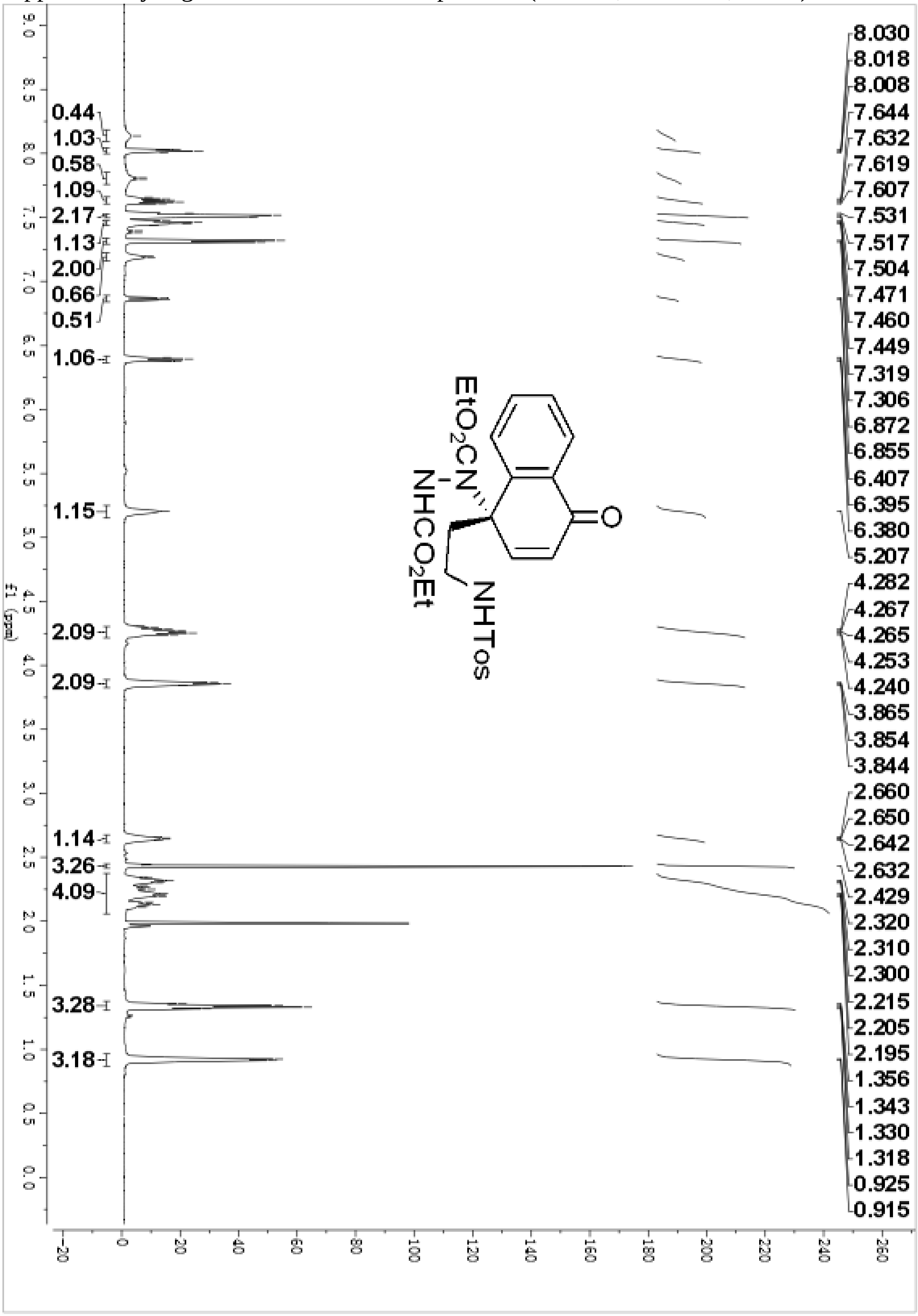

Supplementary Figure 79 ^{13}C NMR of compound **6** (CD_3CN, 150 MHz, 80 °C)

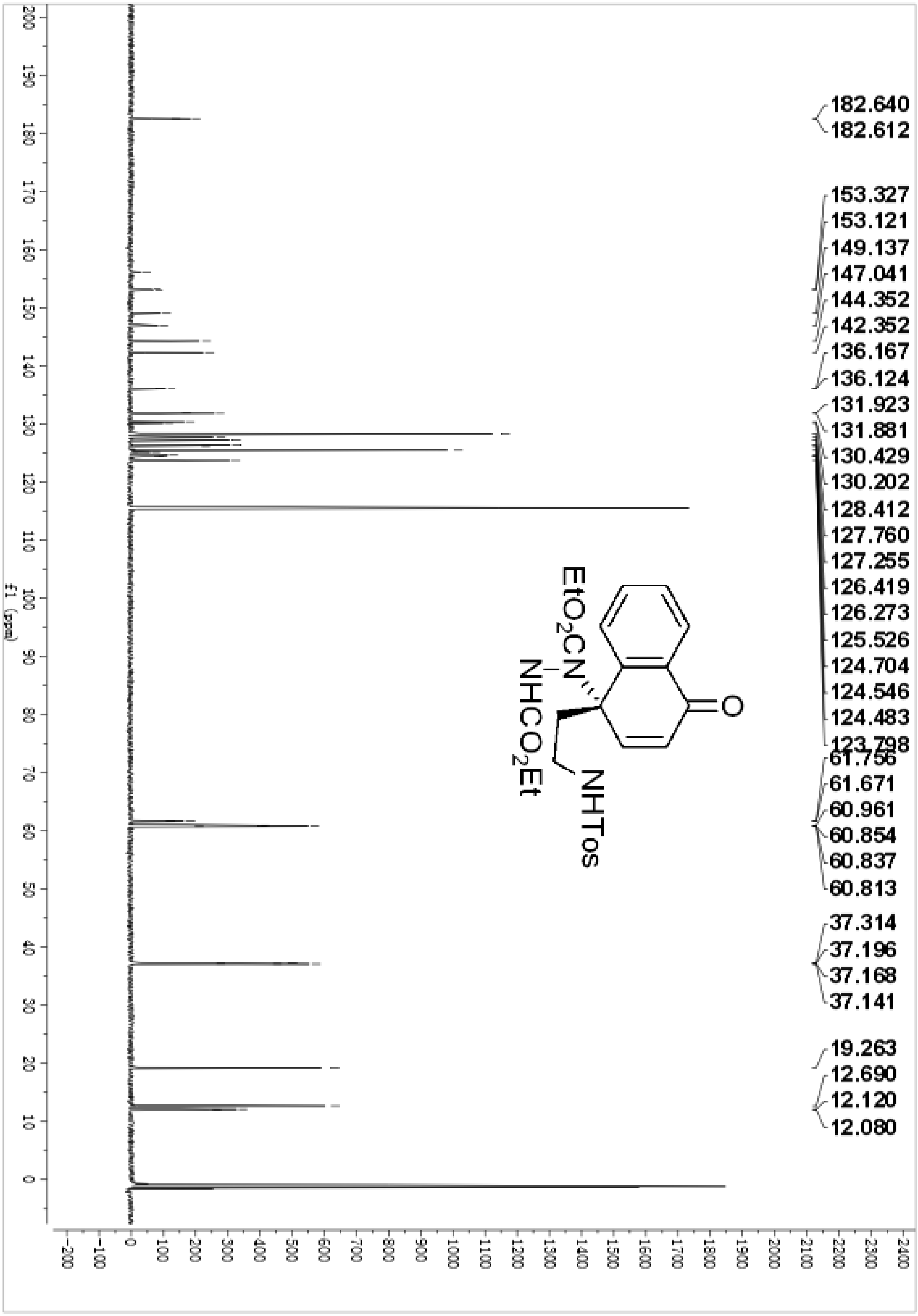

Supplementary Figure 80 ^{1}H NMR of compound **7** ($CDCl_3$, 600 MHz, 60 °C)

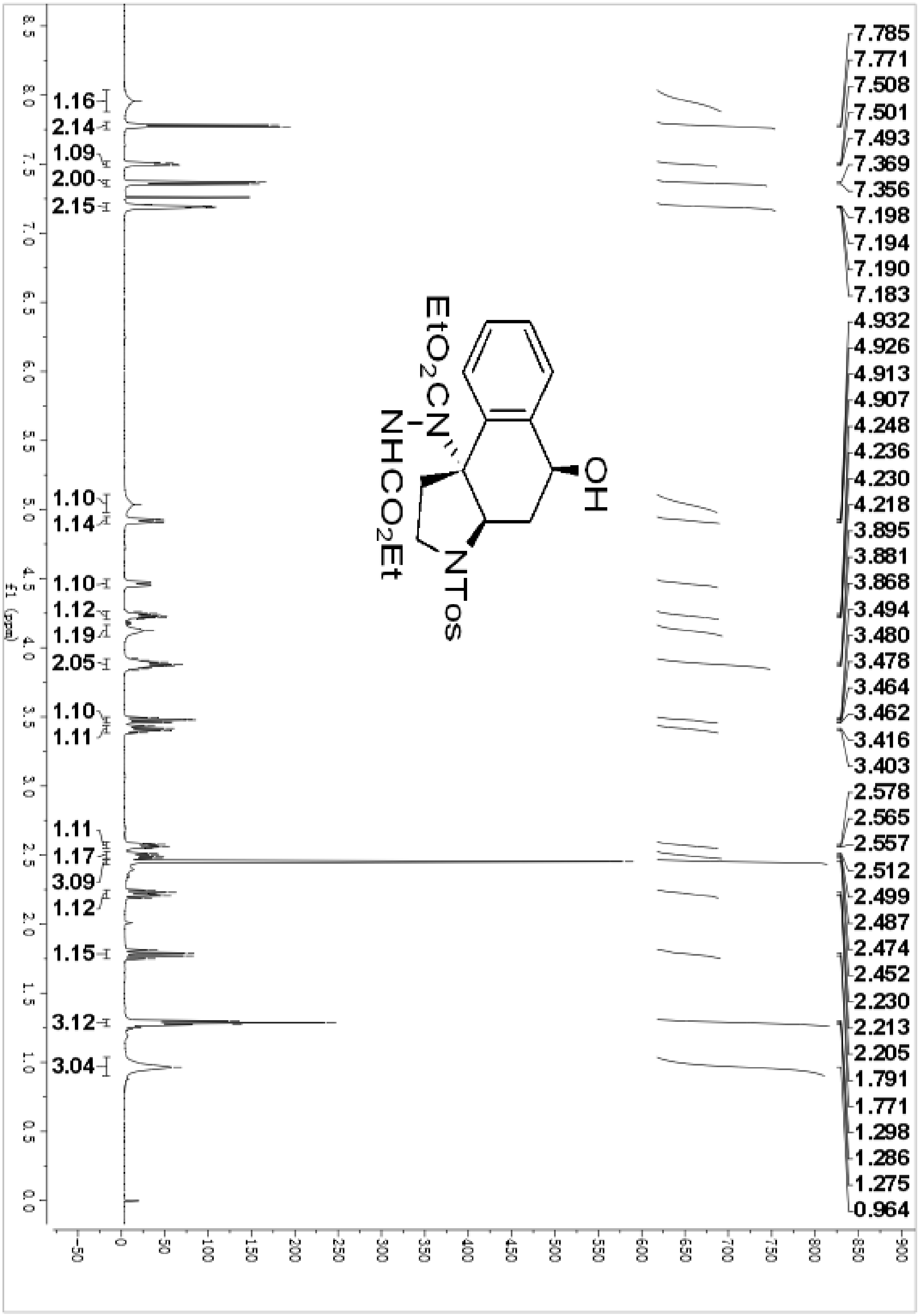

Supplementary Figure 81 ^{13}C NMR of compound **7** ($CDCl_3$, 150 MHz, 60 °C)

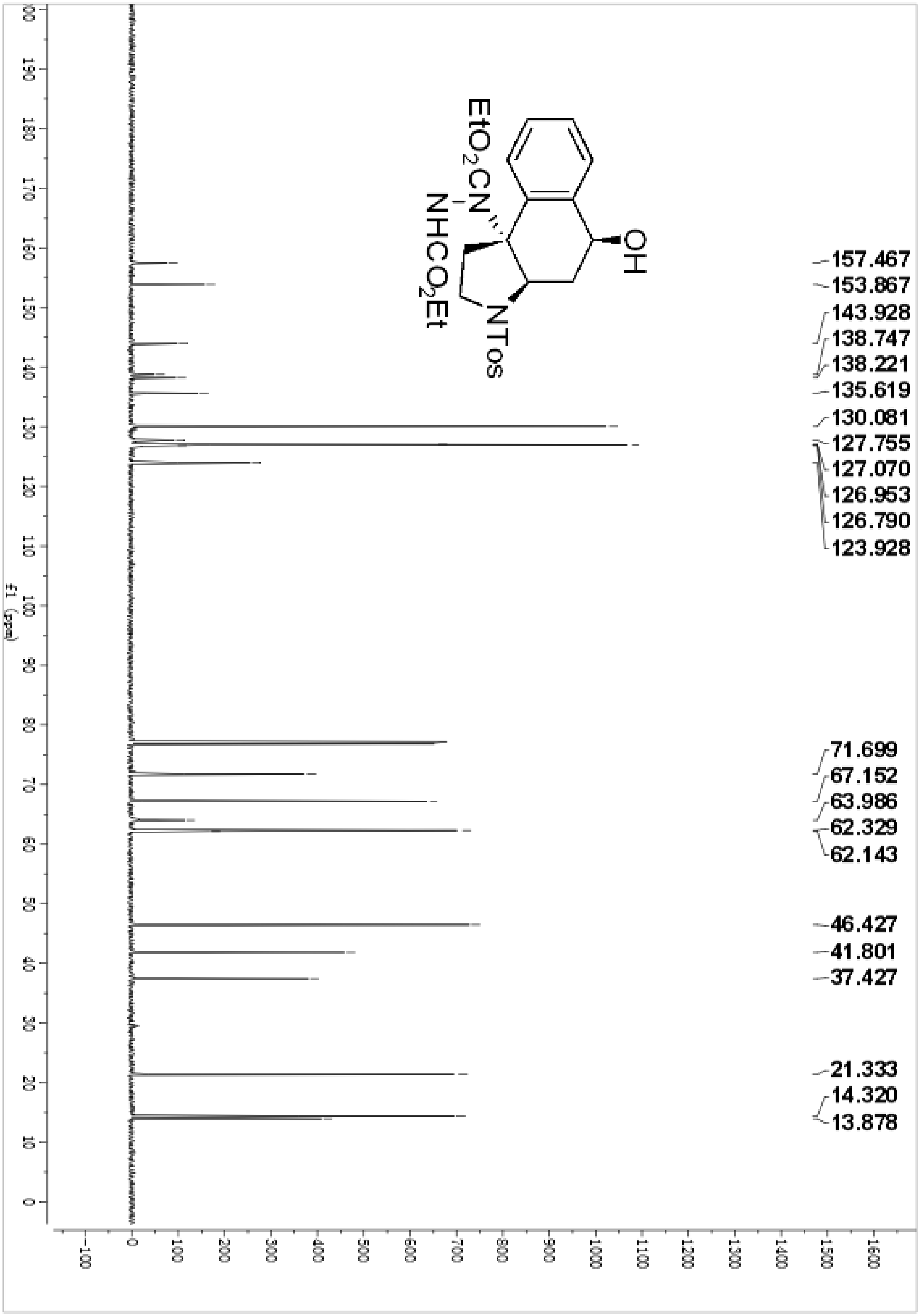

Supplementary Figure 82 DEPT of compound **7** ($CDCl_3$, 150 MHz, 60 °C)

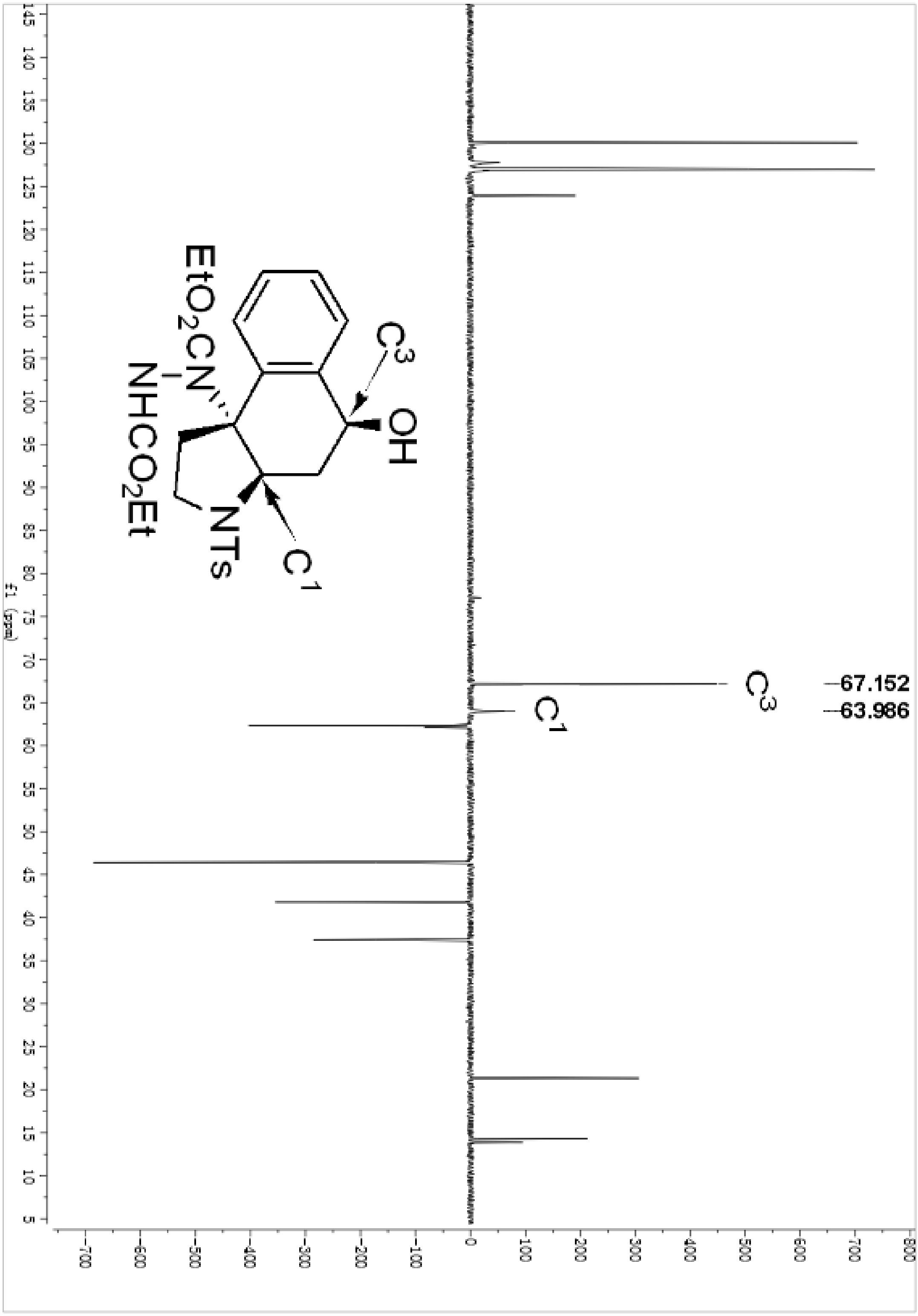

Supplementary Figure 83 HSQC of compound **7** ($CDCl_3$, 150 MHz, 60 °C)

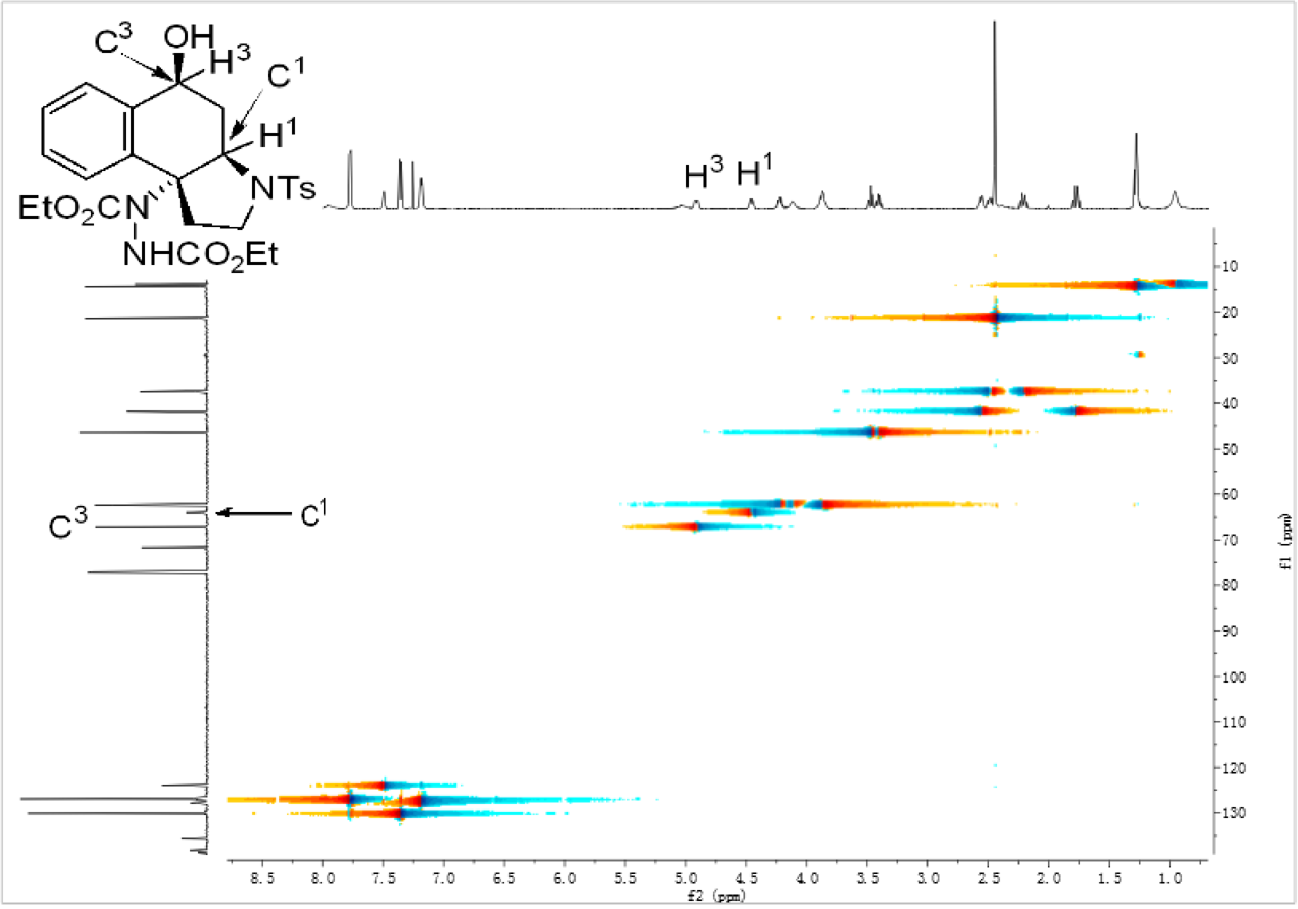

Supplementary Figure 84 COSY of compound **7** ($CDCl_3$, 600 MHz, 60 °C)

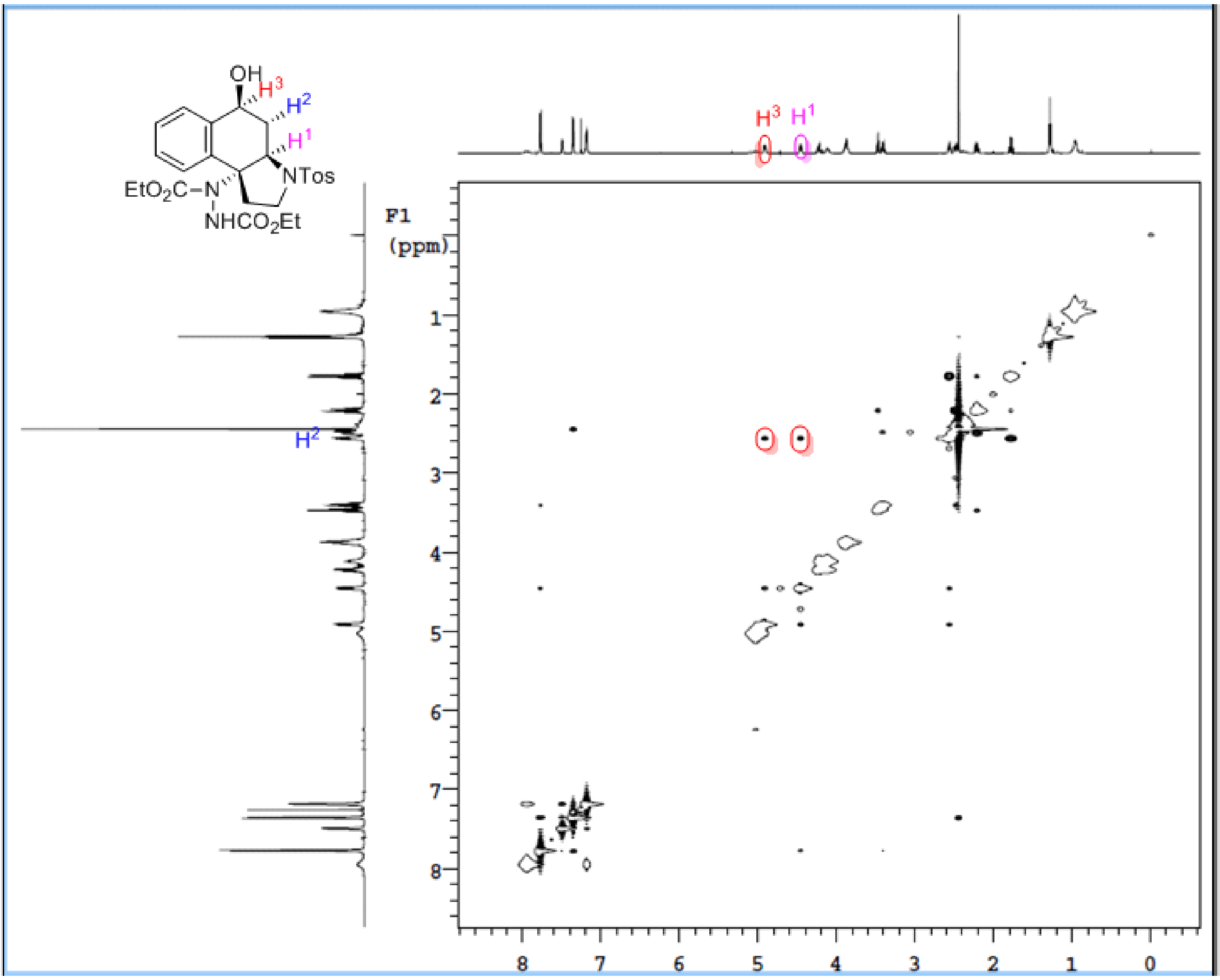

Supplementary Figure 85 ^{1}H NMR of compound **8** ($CDCl_3$, 600 MHz, 60 °C)

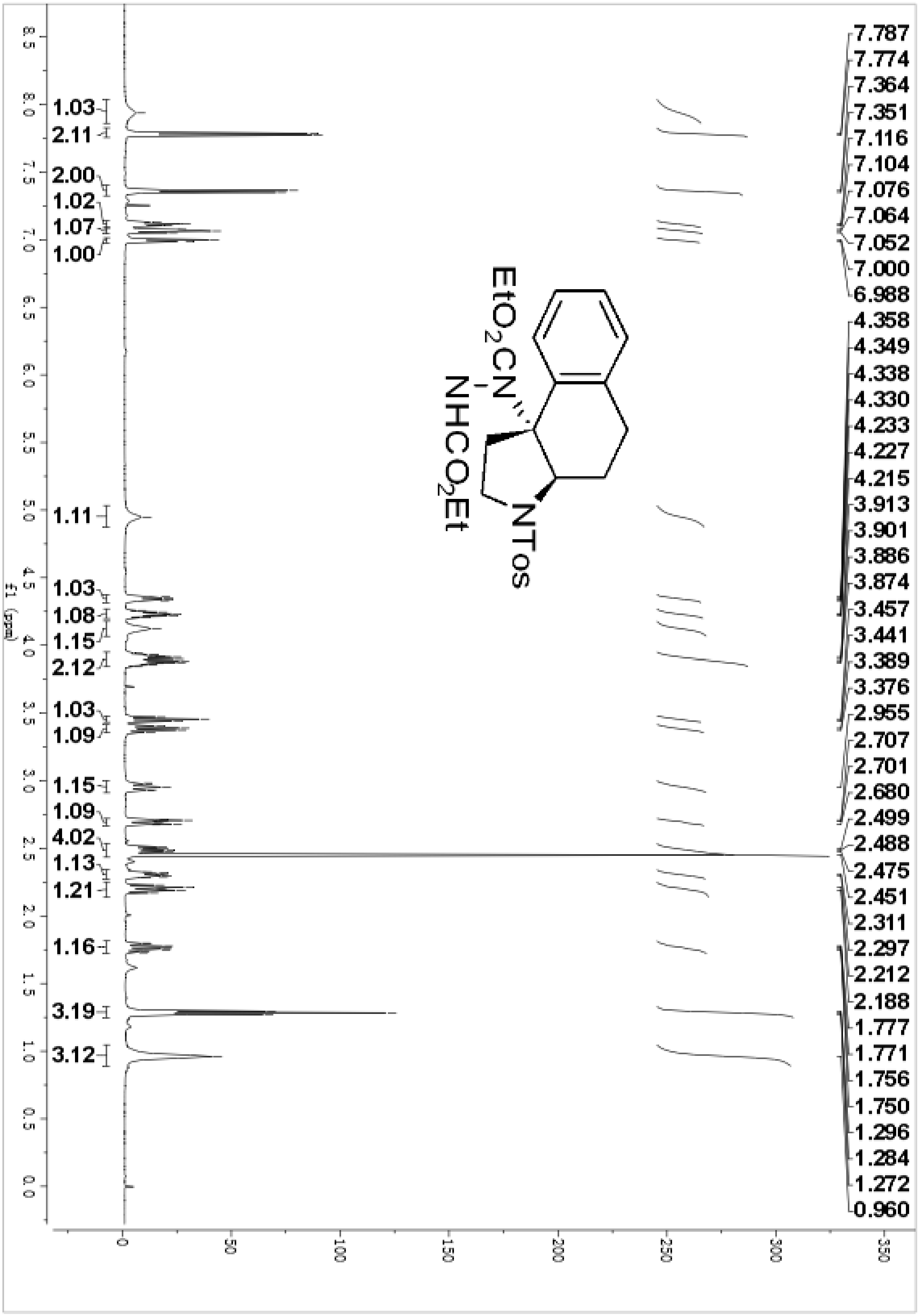

Supplementary Figure 86 ^{13}C NMR of compound **8** ($CDCl_3$, 150 MHz, 60 °C)

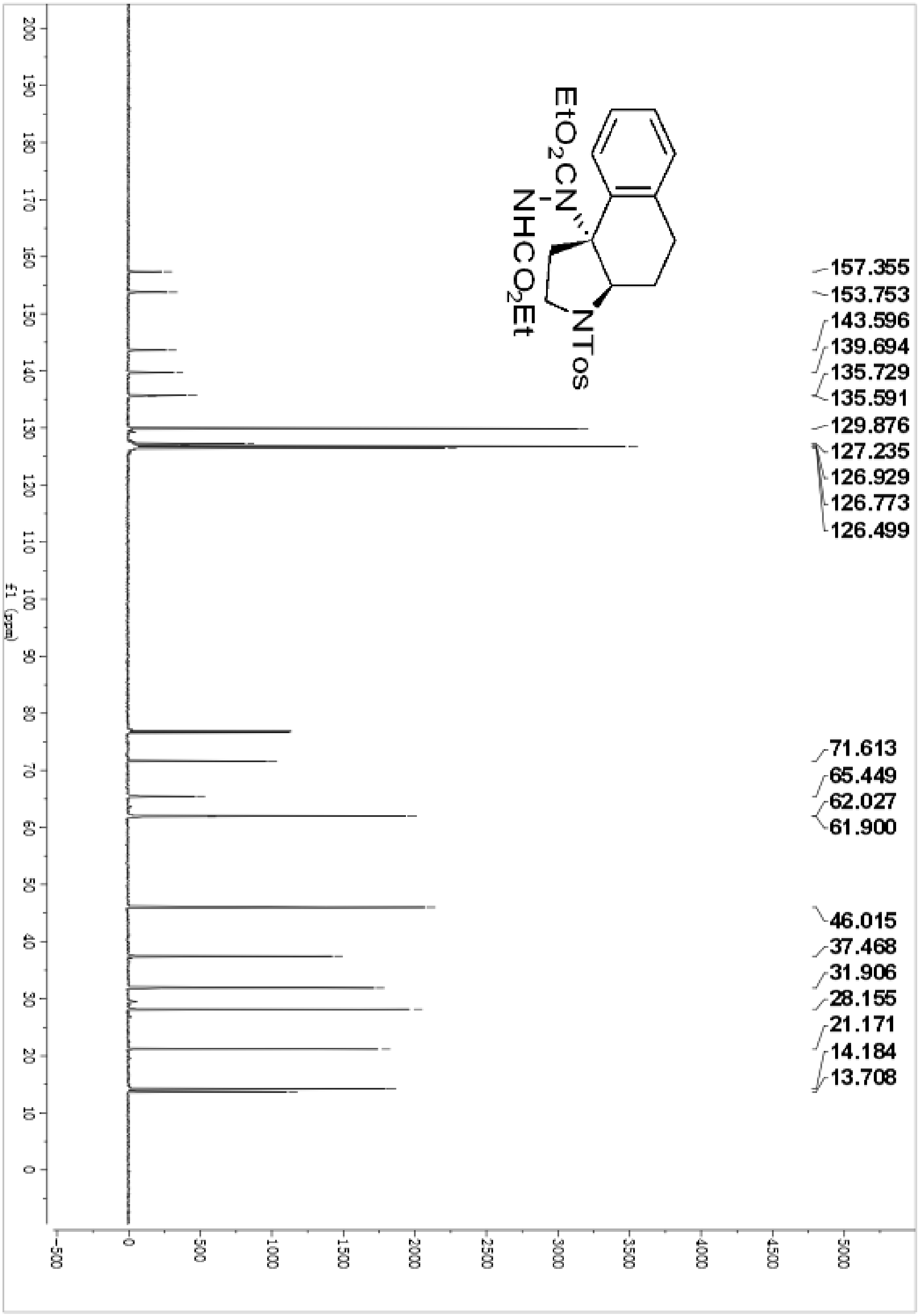

Supplementary Figure 87 ^{1}H NMR of compound **9** (CD_3CN, 600 MHz, 80 °C)

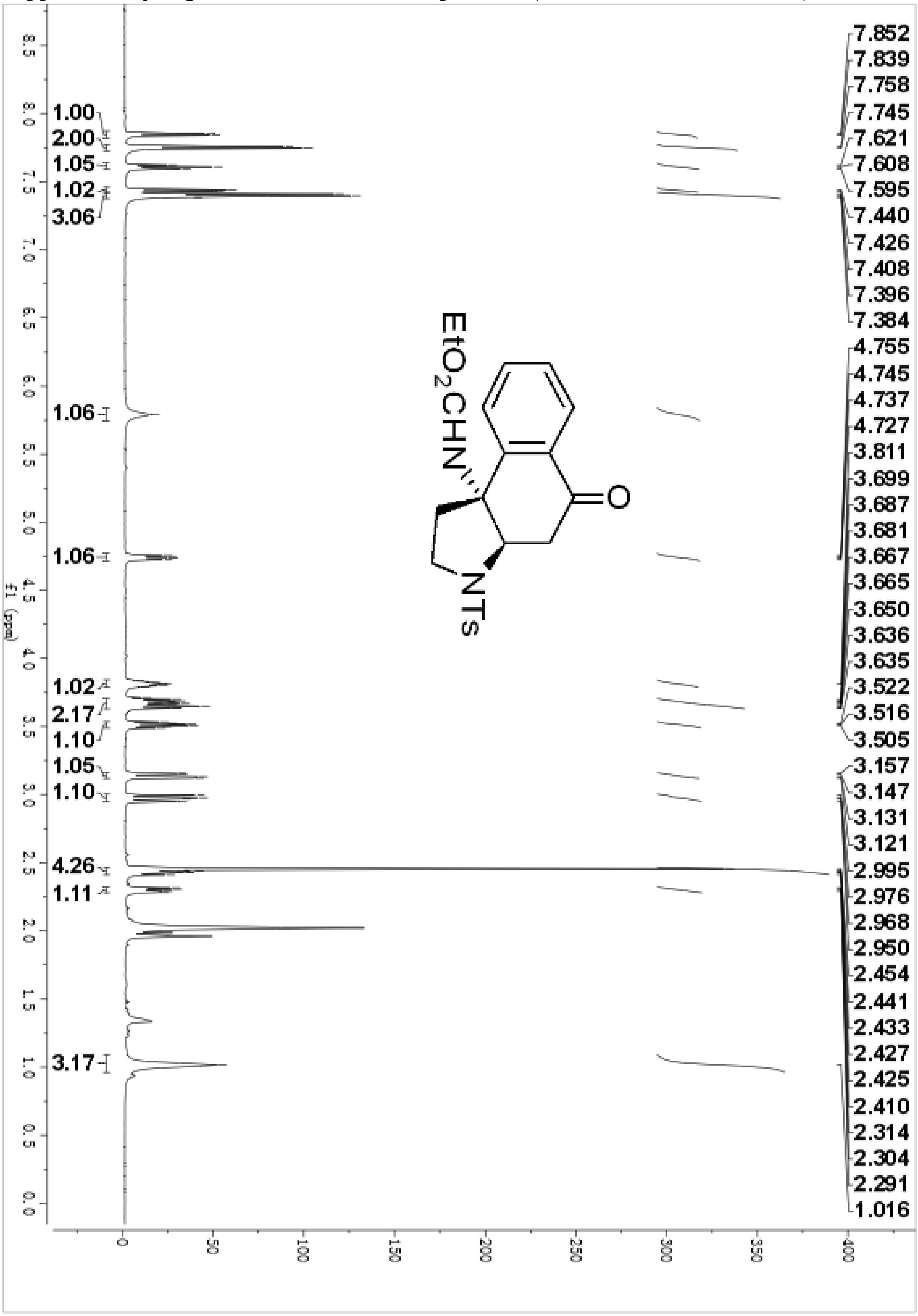

Supplementary Figure 88 ^{13}C NMR of compound **9** (CD_3CN, 150 MHz, 80 °C)

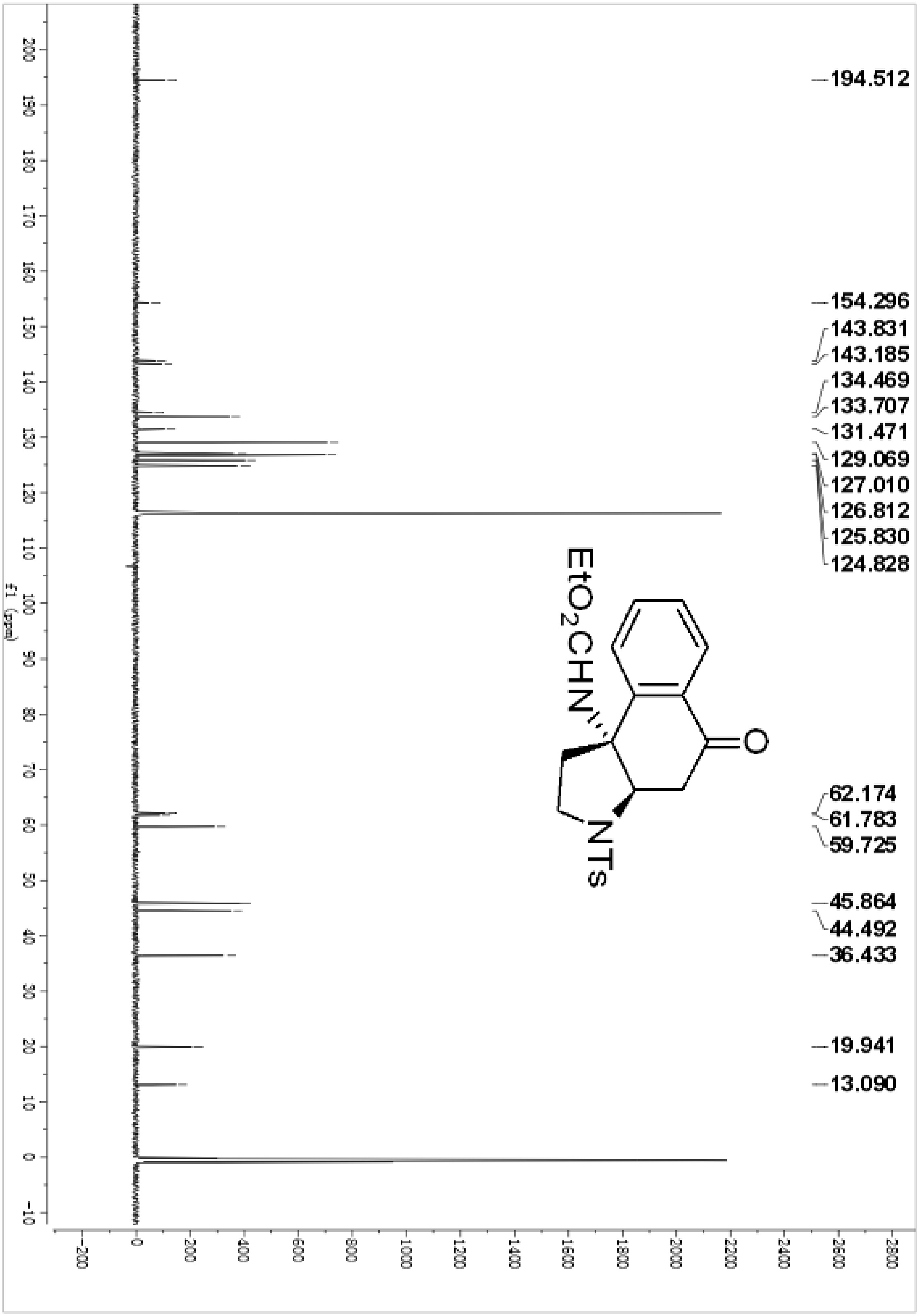

Copies of HPLC analysis

Supplementary Figure 89 HPLC Chromatographs of **2a**

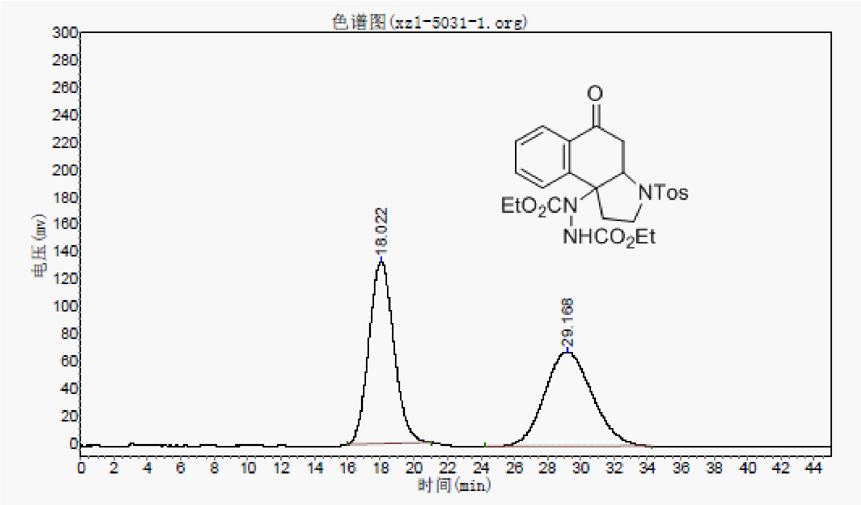

No.	RT/min	Hight	Area	Amount/%
1	18. 022	133482. 672	13866175. 000	50. 4207
2	29. 168	68950. 836	13634768. 000	49. 5793

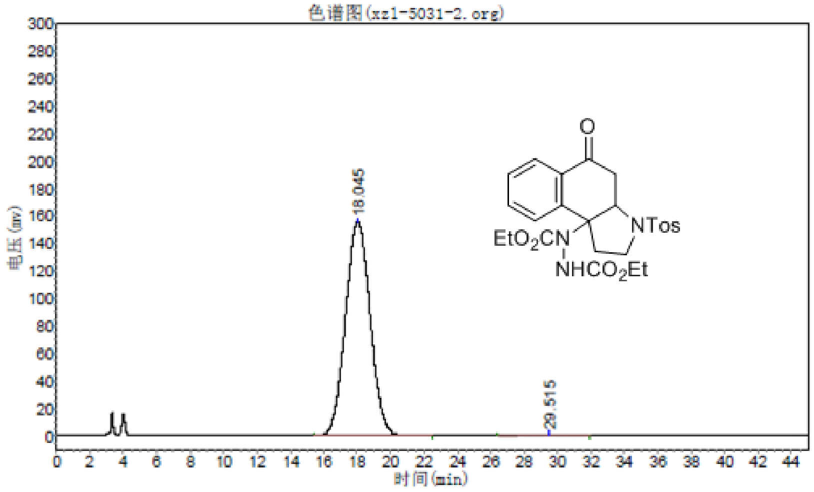

No.	RT/min	Hight	Area	Amount/%
1	18. 045	155409. 422	15963079. 000	99. 4317
2	29. 515	522. 805	91240. 898	0. 5683

Supplementary Figure 90 HPLC Chromatographs of **2b**

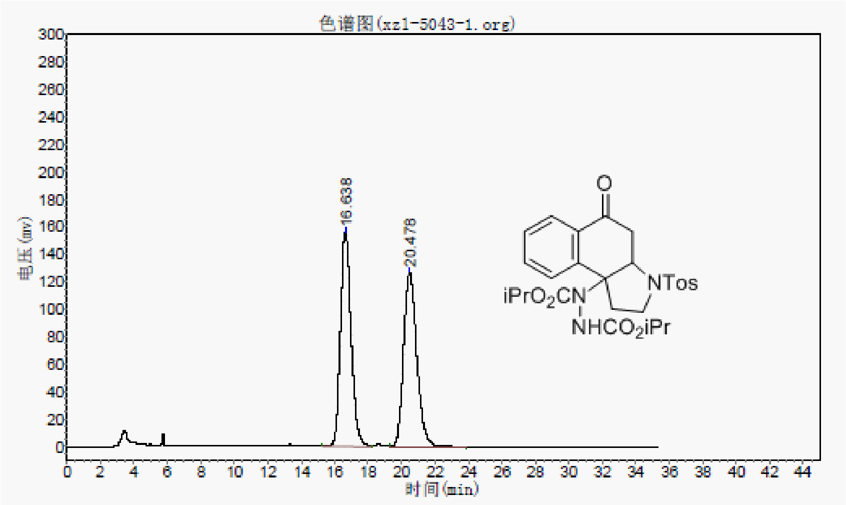

No.	RT/min	Hight	Area	Amount/%
1	16.638	156152.641	6935046.500	50.0029
2	20.478	127313.922	6934249.000	49.9971

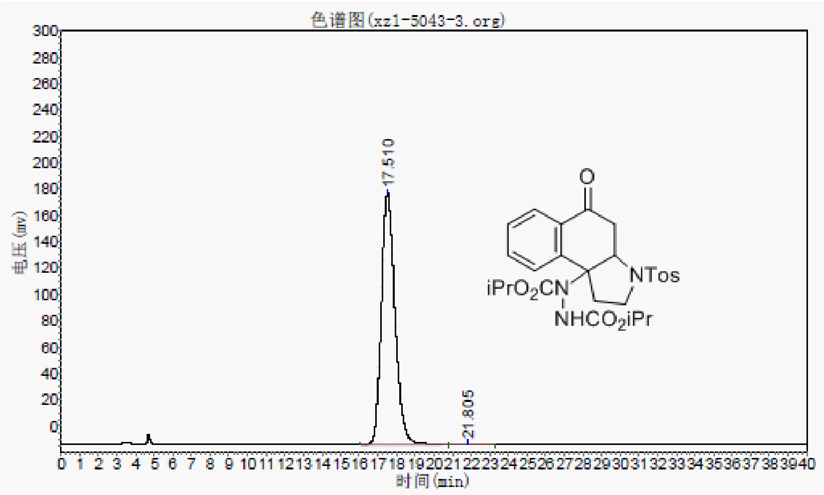

No.	RT/min	Hight	Area	Amount/%
1	17.510	191080.641	9737183.000	99.8645
2	21.805	245.515	13214.150	0.1355

Supplementary Figure 91 HPLC Chromatographs of **2c**

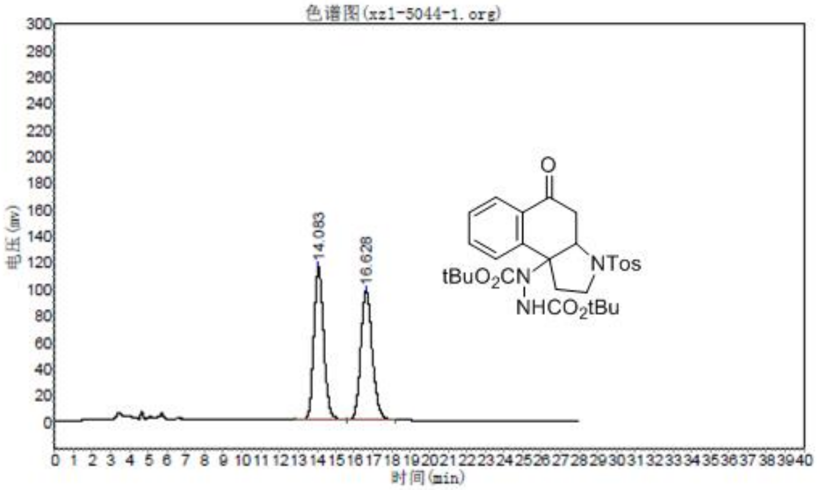

No.	RT/min	Hight	Area	Amount/%
1	14.083	116026.836	4208270.000	50.2001
2	16.628	97751.352	4174715.500	49.7999

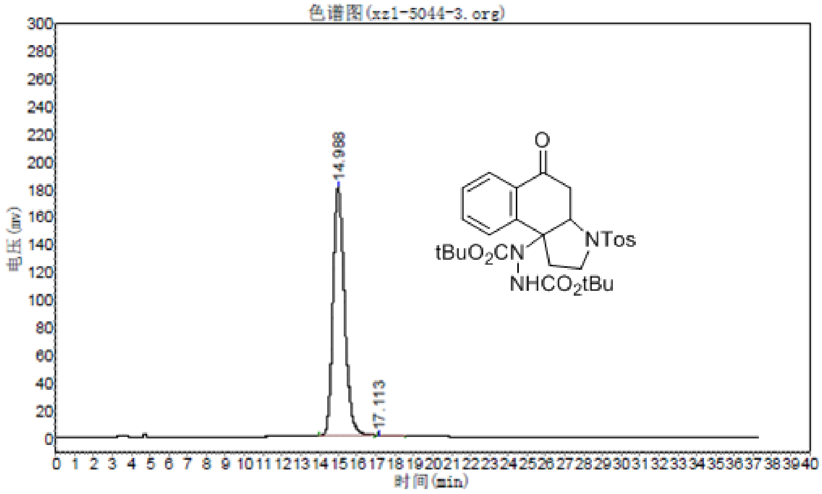

No.	RT/min	Hight	Area	Amount/%
1	14.988	180267.500	7531697.000	99.9307
2	17.113	9.105	5226.645	0.0693

Supplementary Figure 92 HPLC Chromatographs of **2d**

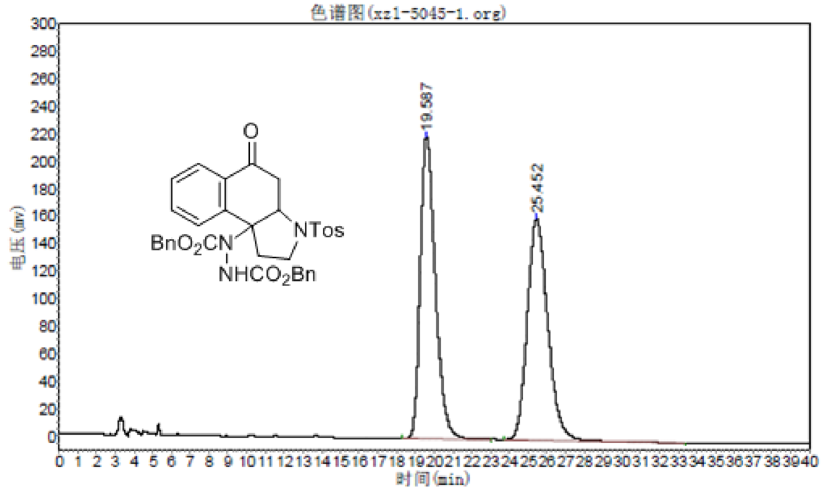

No.	RT/min	Hight	Area	Amount/%
1	19. 587	220056. 672	12335302. 000	50. 0602
2	25. 452	161432. 594	12305645. 000	49. 9398

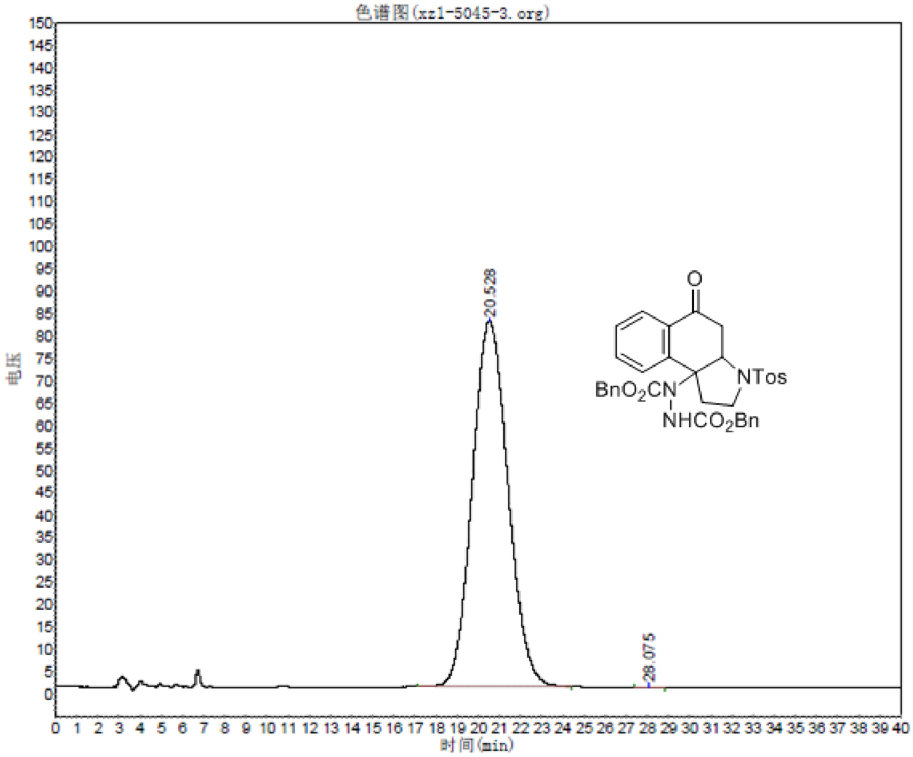

No.	RT/min	Hight	Area	Amount/%
1	20. 528	81635. 219	9573359. 000	99. 9982
2	28. 075	28. 024	175. 499	0. 0018

Supplementary Figure 93 HPLC Chromatographs of **2e**

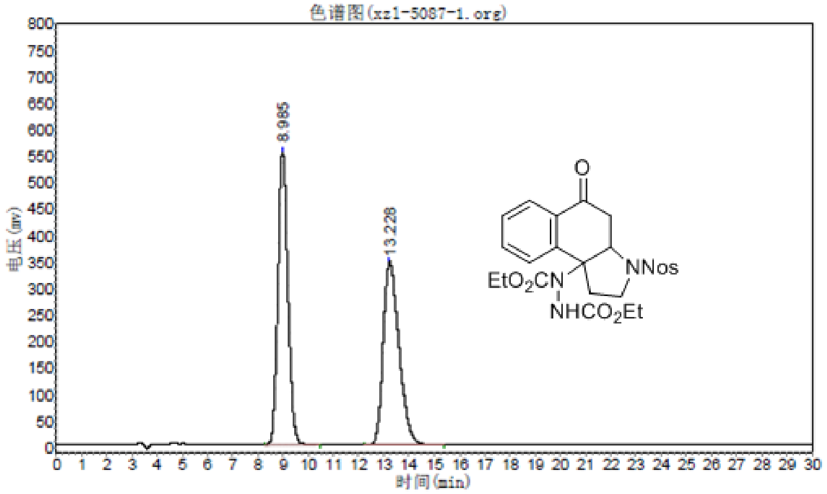

No.	RT/min	Hight	Area	Amount/%
1	8. 985	553079. 438	15237203. 000	50. 1526
2	13. 228	346554. 875	15144496. 000	49. 8474

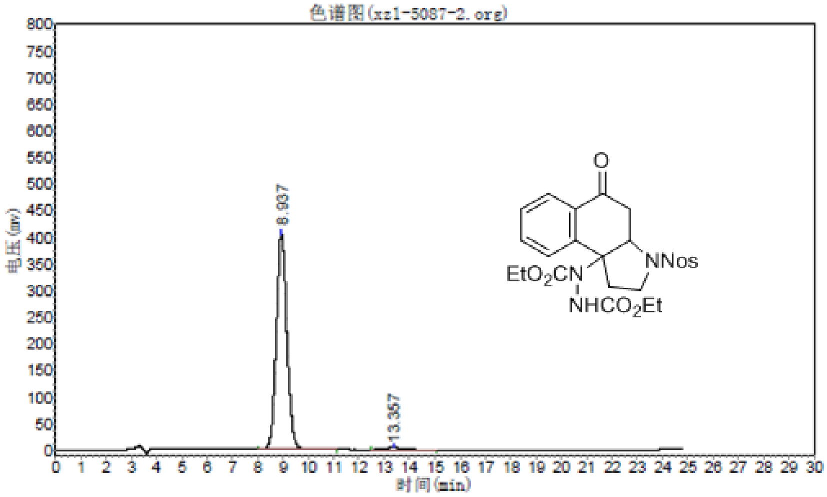

No.	RT/min	Hight	Area	Amount/%
1	8. 937	404373. 750	11748547. 000	98. 7889
2	13. 357	3318. 359	144031. 297	1. 2111

Supplementary Figure 94 HPLC Chromatographs of **2f**

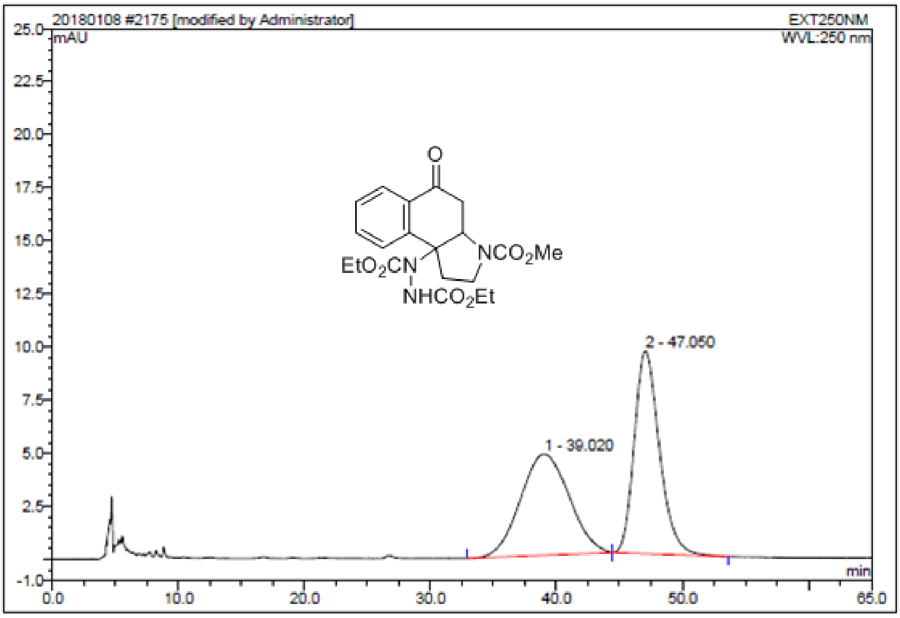

No.	Ret.Time min	Peak Name	Height mAU	Area mAU*min	Rel.Area %	Amount	Type
1	39.02	n.a.	4.778	21.333	49.15	n.a.	BMb*
2	47.05	n.a.	9.540	22.071	50.85	n.a.	bMB*
Total:			14.318	43.404	100.00	0.000	

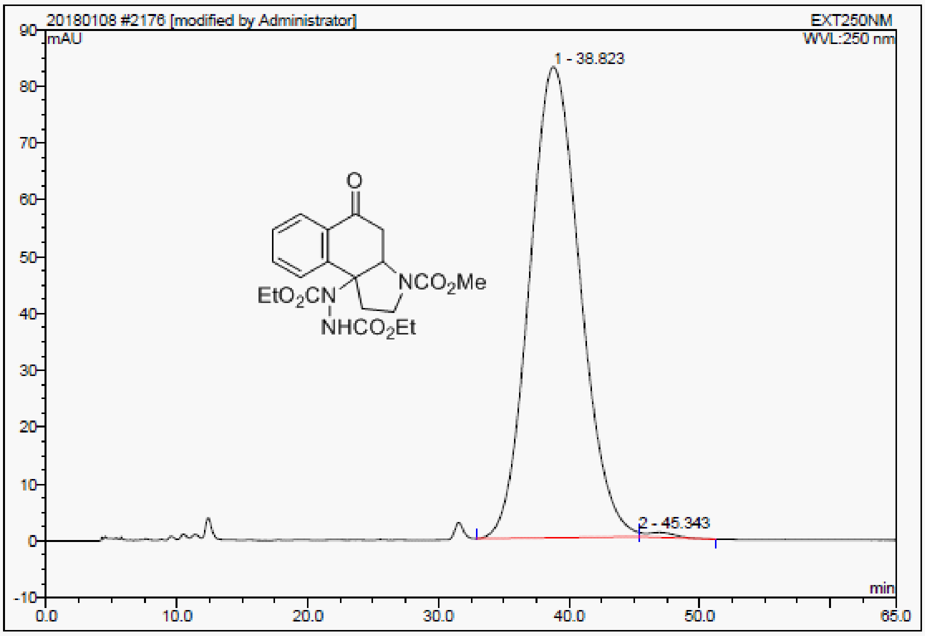

No.	Ret.Time min	Peak Name	Height mAU	Area mAU*min	Rel.Area %	Amount	Type
1	38.82	n.a.	82.819	371.700	99.37	n.a.	BM *
2	45.34	n.a.	0.780	2.358	0.63	n.a.	MB*
Total:			83.599	374.058	100.00	0.000	

Supplementary Figure 95 HPLC Chromatographs of **2g**

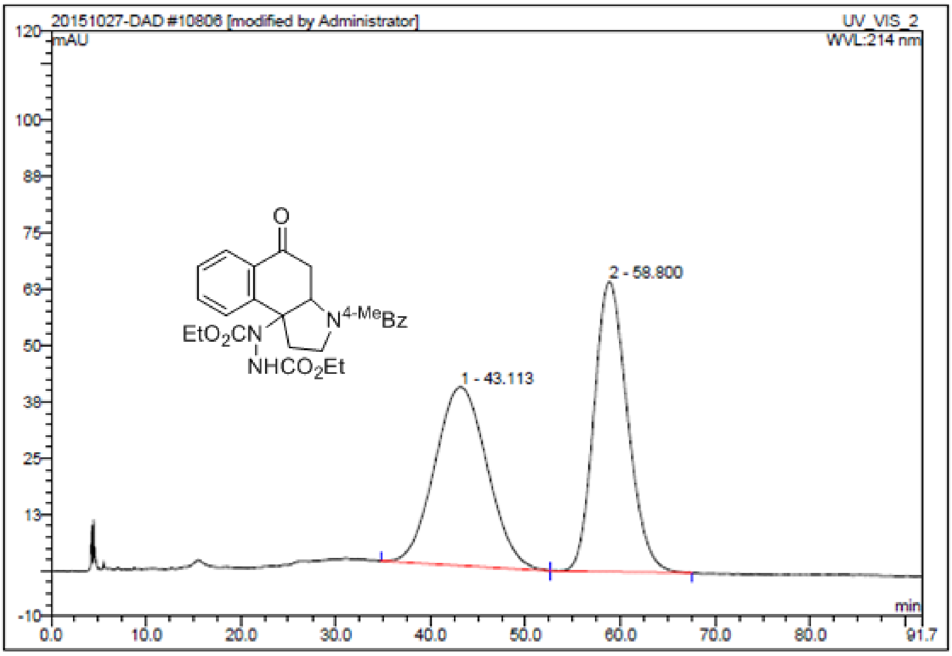

No.	Ret.Time min	Peak Name	Height mAU	Area mAU*min	Rel.Area %	Amount	Type
1	43.11	n.a.	39.762	254.286	49.13	n.a.	BMb*
2	58.80	n.a.	64.367	263.297	50.87	n.a.	bMB*
Total:			104.129	517.583	100.00	0.000	

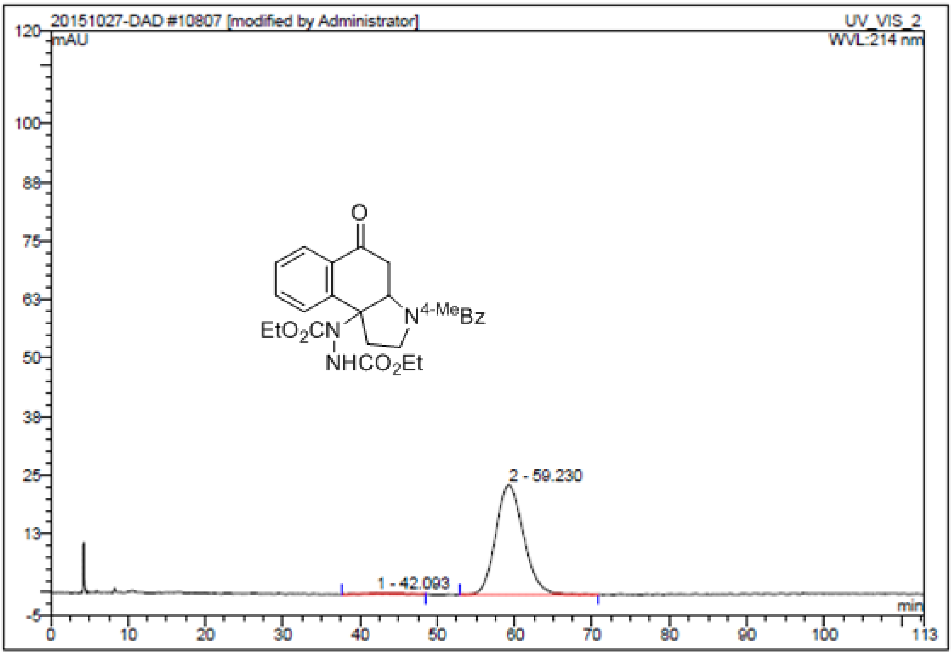

No.	Ret.Time min	Peak Name	Height mAU	Area mAU*min	Rel.Area %	Amount	Type
1	42.09	n.a.	0.266	0.957	0.98	n.a.	BMB*
2	59.23	n.a.	23.413	96.814	99.02	n.a.	BMB*
Total:			23.679	97.772	100.00	0.000	

Supplementary Figure 96 HPLC Chromatographs of **2h**

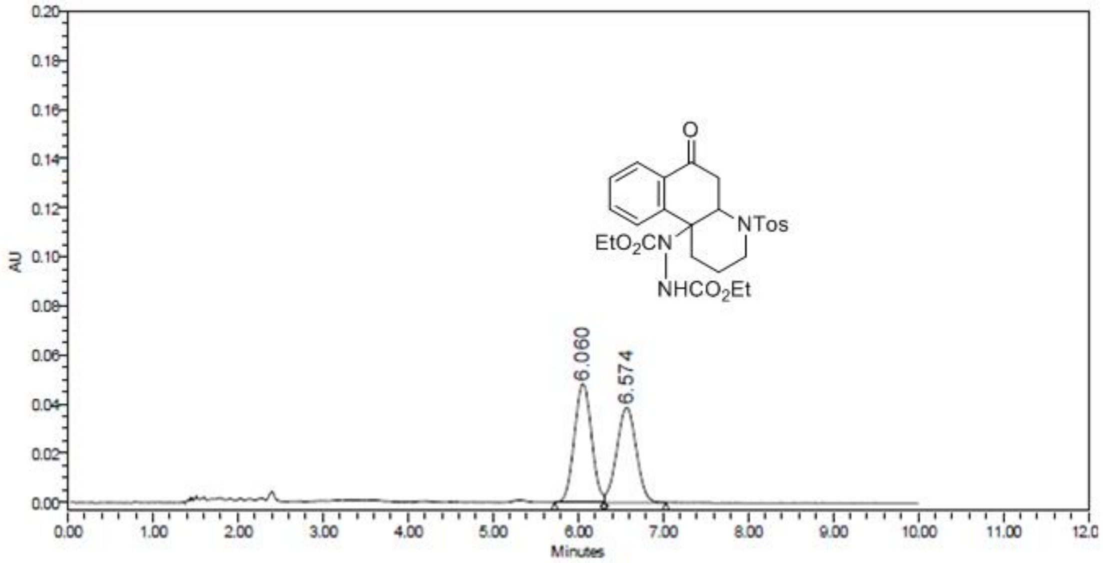

Peak Results

	RT	Area	Height	% Area
1	6.060	679967	48423	52.29
2	6.574	620332	38852	47.71

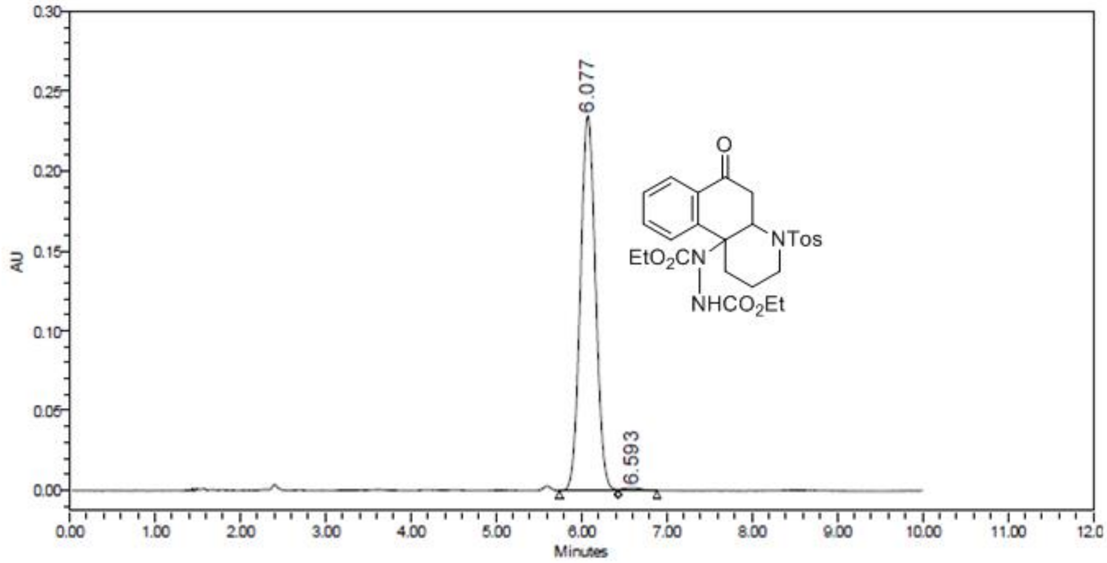

Peak Results

	RT	Area	Height	% Area
1	6.077	2899985	234937	99.05
2	6.593	27934	1928	0.95

Supplementary Figure 97 HPLC Chromatographs of **2i**

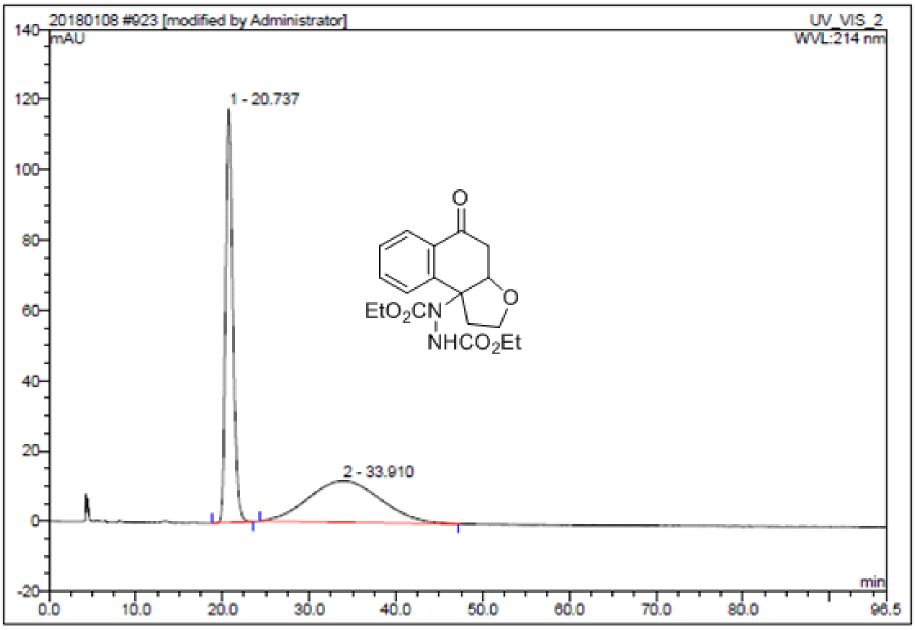

No.	Ret.Time min	Peak Name	Height mAU	Area mAU*min	Rel.Area %	Amount	Type
1	20.74	n.a.	117.789	114.834	50.29	n.a.	BMB*
2	33.91	n.a.	11.783	113.489	49.71	n.a.	BMB*
Total:			129.572	228.323	100.00	0.000	

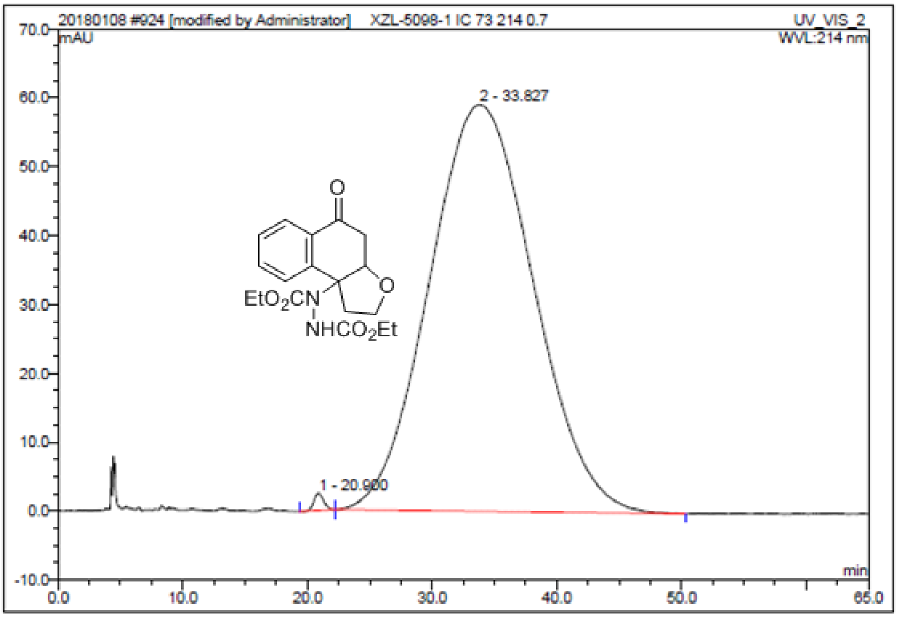

No.	Ret.Time min	Peak Name	Height mAU	Area mAU*min	Rel.Area %	Amount	Type
1	20.90	n.a.	2.512	2.238	0.39	n.a.	BMb*
2	33.83	n.a.	59.062	572.605	99.61	n.a.	bMB*
Total:			61.574	574.843	100.00	0.000	

Supplementary Figure 98 HPLC Chromatographs of **2j**

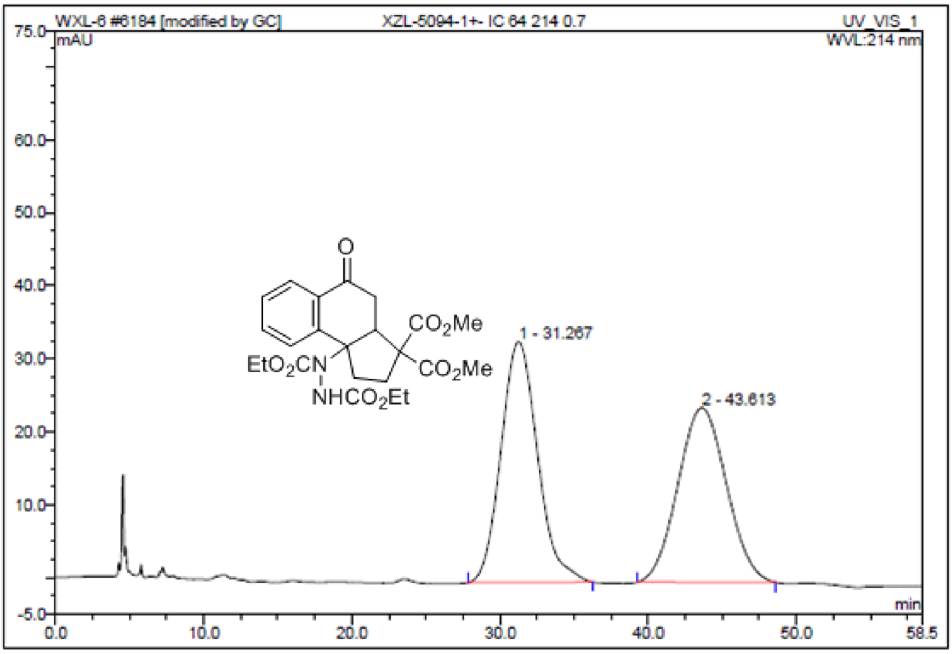

No.	Ret.Time min	Peak Name	Height mAU	Area mAU*min	Rel.Area %	Amount	Type
1	31.27	n.a.	32.994	94.468	50.93	n.a.	BMB
2	43.61	n.a.	23.919	91.001	49.07	n.a.	BMB
Total:			56.913	185.469	100.00	0.000	

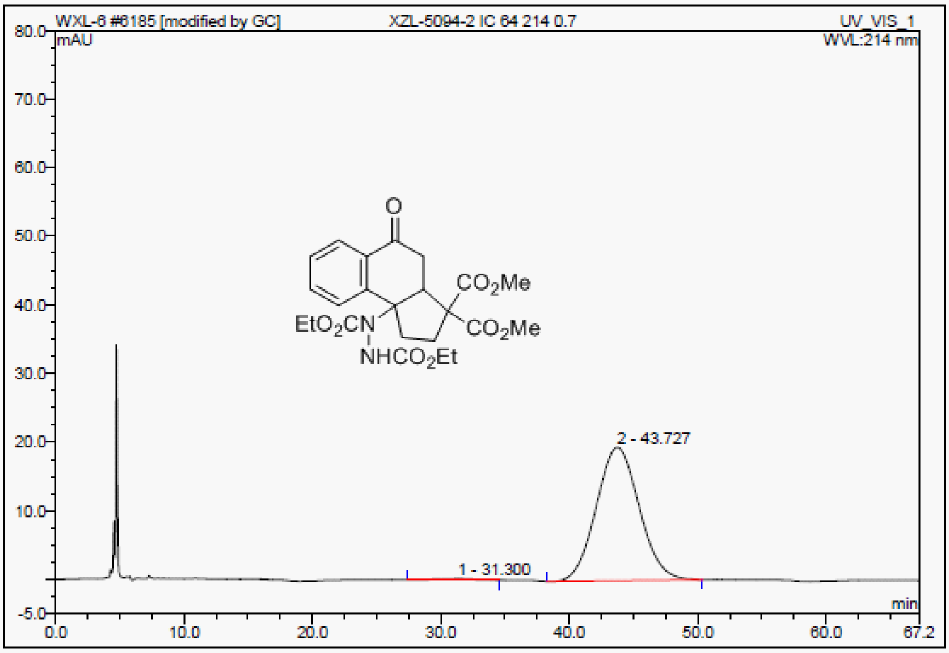

No.	Ret.Time min	Peak Name	Height mAU	Area mAU*min	Rel.Area %	Amount	Type
1	31.30	n.a.	0.188	0.483	0.64	n.a.	BMB*
2	43.73	n.a.	19.427	75.401	99.36	n.a.	BMB*
Total:			19.615	75.883	100.00	0.000	

Supplementary Figure 99 HPLC Chromatographs of **2k**

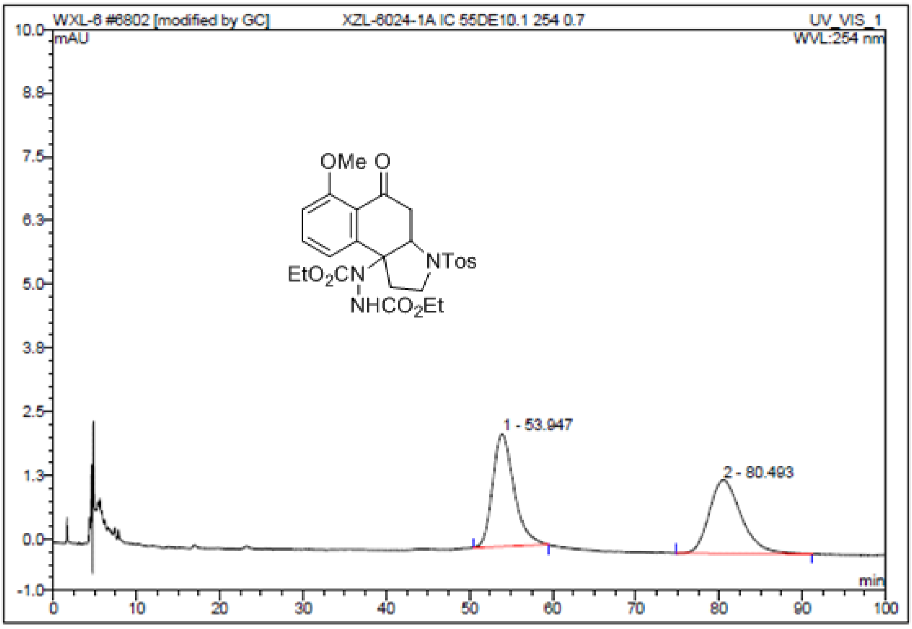

No.	Ret.Time min	Peak Name	Height mAU	Area mAU*min	Rel.Area %	Amount	Type
1	53.95	n.a.	2.204	6.602	50.76	n.a.	BMB*
2	80.49	n.a.	1.438	6.404	49.24	n.a.	BMB*
Total:			3.643	13.007	100.00	0.000	

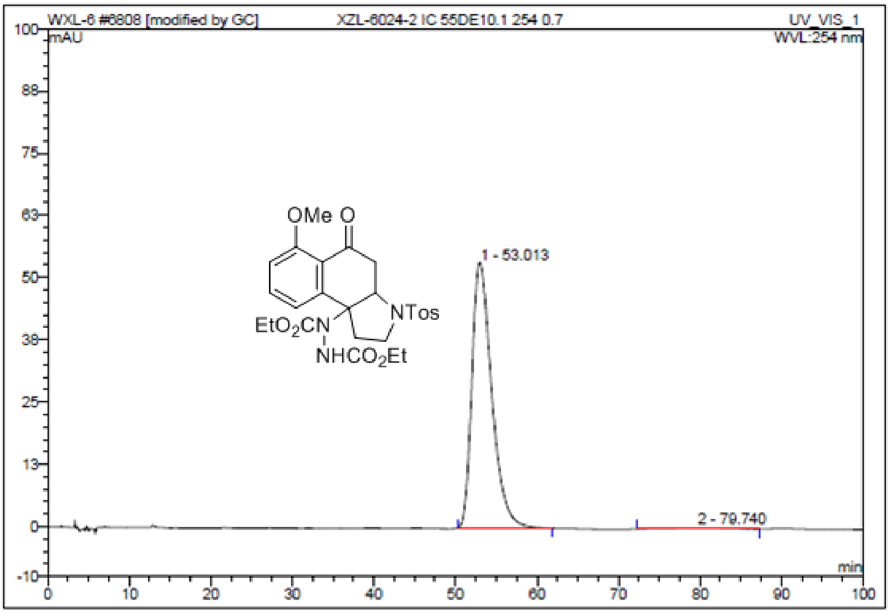

No.	Ret.Time min	Peak Name	Height mAU	Area mAU*min	Rel.Area %	Amount	Type
1	53.01	n.a.	53.300	150.383	99.02	n.a.	BMB*
2	79.74	n.a.	0.210	1.488	0.98	n.a.	BMB*
Total:			53.510	151.871	100.00	0.000	

Supplementary Figure 100 HPLC Chromatographs of **2l**

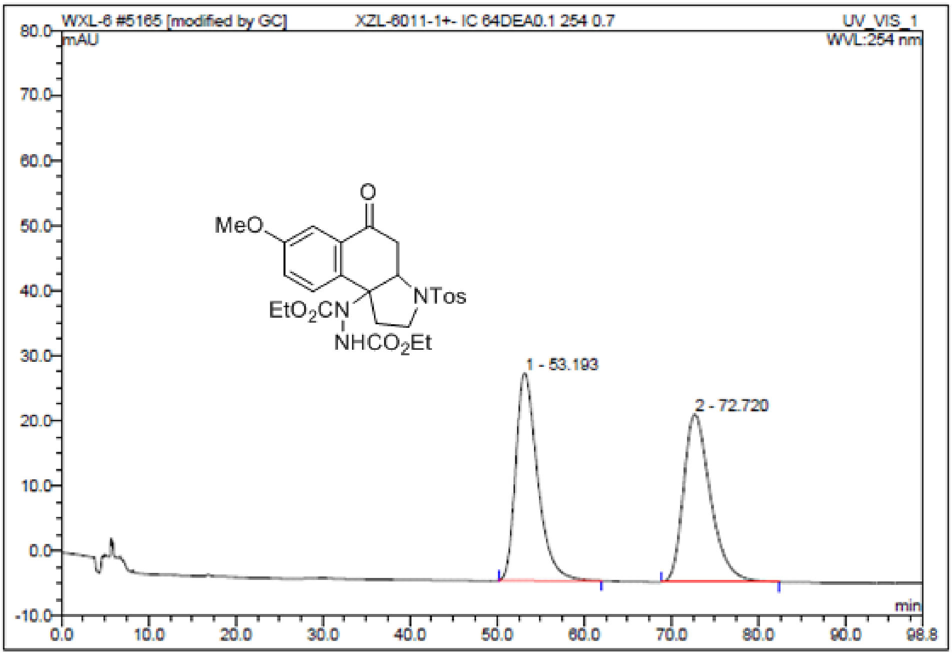

No.	Ret.Time min	Peak Name	Height mAU	Area mAU*min	Rel.Area %	Amount	Type
1	53.19	n.a.	31.927	92.422	49.92	n.a.	BMB*
2	72.72	n.a.	25.782	92.720	50.08	n.a.	BMB*
Total:			57.709	185.142	100.00	0.000	

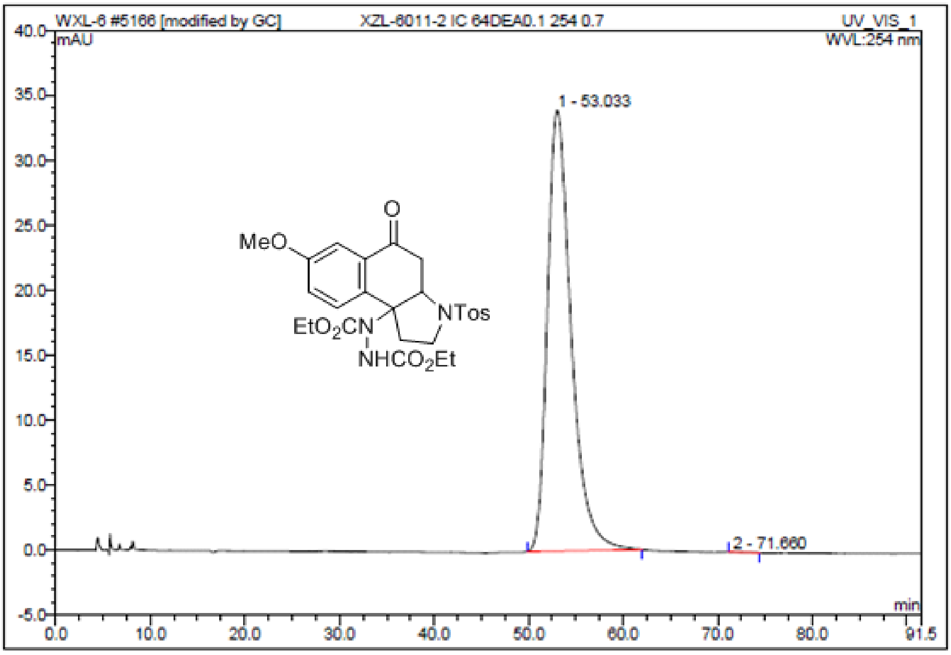

No.	Ret.Time min	Peak Name	Height mAU	Area mAU*min	Rel.Area %	Amount	Type
1	53.03	n.a.	33.904	98.772	99.95	n.a.	BMB*
2	71.66	n.a.	0.017	0.049	0.05	n.a.	BMB*
Total:			33.920	98.821	100.00	0.000	

Supplementary Figure 101 HPLC Chromatographs of **2m**

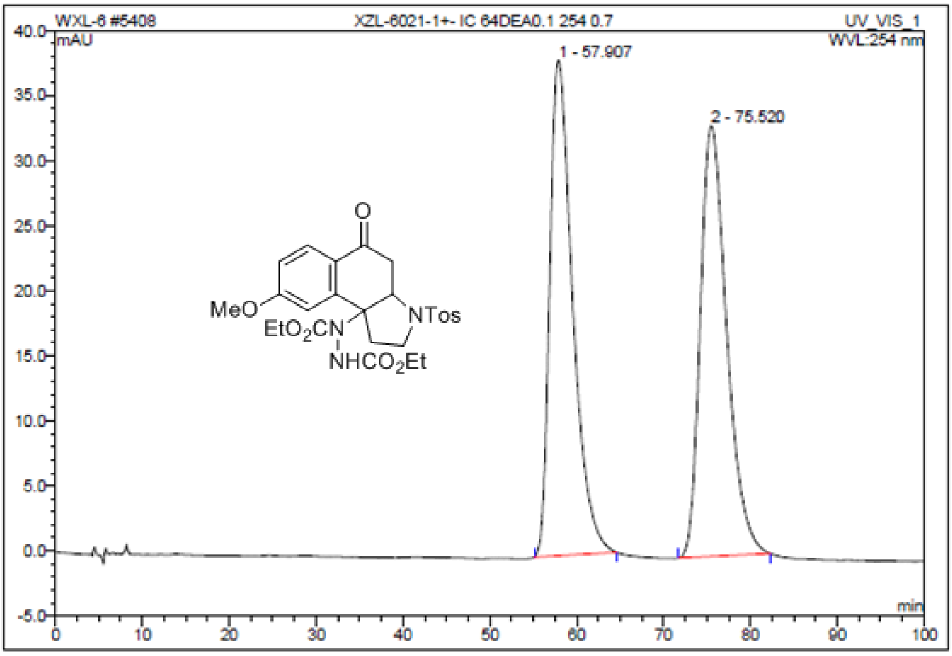

No.	Ret.Time min	Peak Name	Height mAU	Area mAU*min	Rel.Area %	Amount	Type
1	57.91	n.a.	38.077	116.255	50.01	n.a.	BMB
2	75.52	n.a.	33.101	116.198	49.99	n.a.	BMB
Total:			71.178	232.454	100.00	0.000	

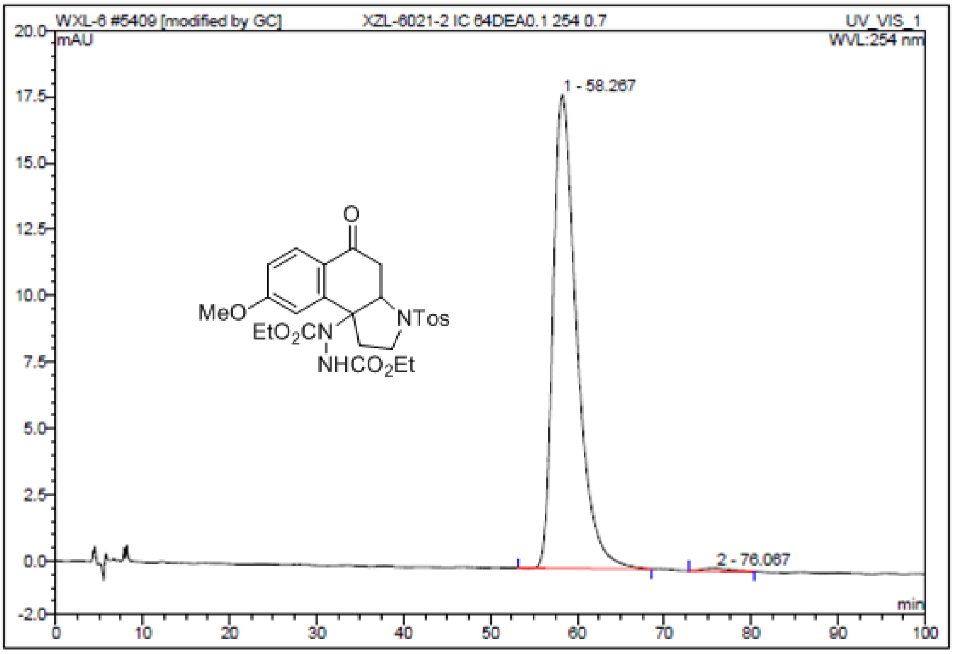

No.	Ret.Time min	Peak Name	Height mAU	Area mAU*min	Rel.Area %	Amount	Type
1	58.27	n.a.	17.838	56.229	99.32	n.a.	BMB*
2	76.07	n.a.	0.109	0.385	0.68	n.a.	BMB*
Total:			17.947	56.614	100.00	0.000	

Supplementary Figure 102 HPLC Chromatographs of **2n**

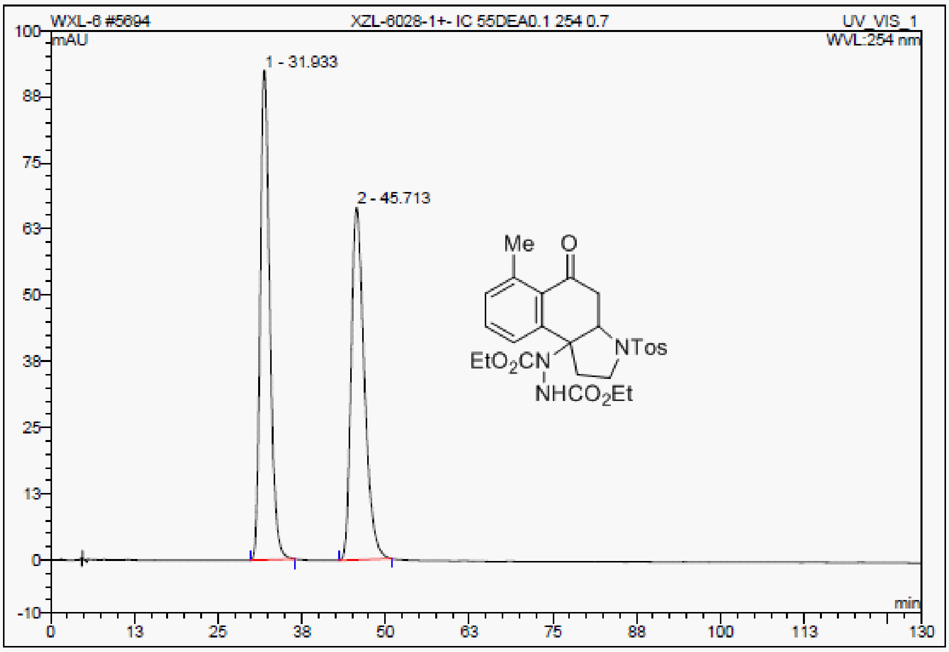

No.	Ret.Time min	Peak Name	Height mAU	Area mAU*min	Rel.Area %	Amount	Type
1	31.93	n.a.	92.476	153.275	50.23	n.a.	BMB
2	45.71	n.a.	66.476	151.861	49.77	n.a.	BMB
Total:			158.952	305.135	100.00	0.000	

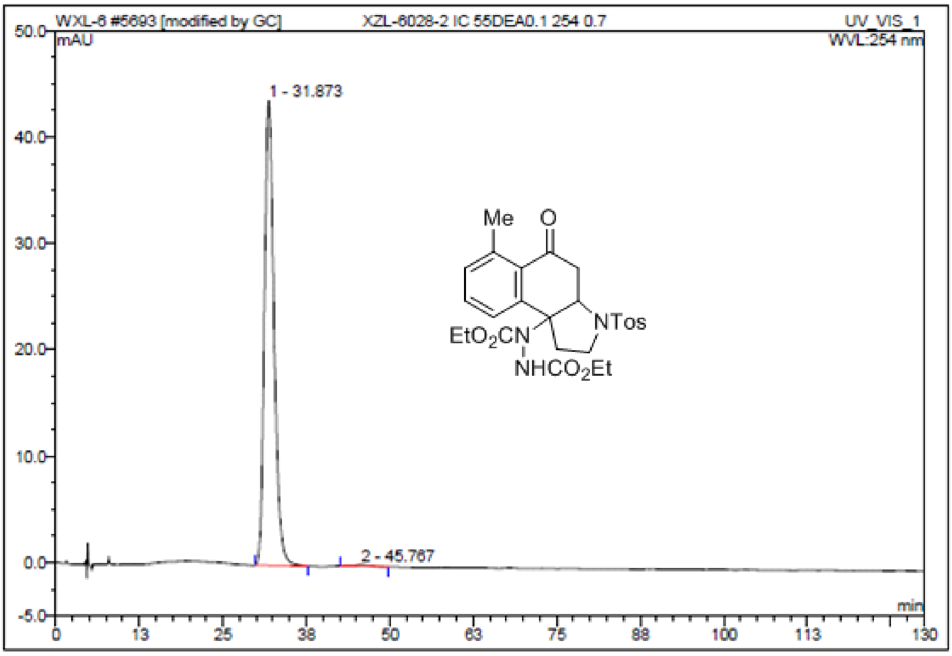

No.	Ret.Time min	Peak Name	Height mAU	Area mAU*min	Rel.Area %	Amount	Type
1	31.87	n.a.	43.666	72.911	99.50	n.a.	BMB*
2	45.77	n.a.	0.139	0.370	0.50	n.a.	BMB*
Total:			43.805	73.281	100.00	0.000	

Supplementary Figure 103 HPLC Chromatographs of **2o**

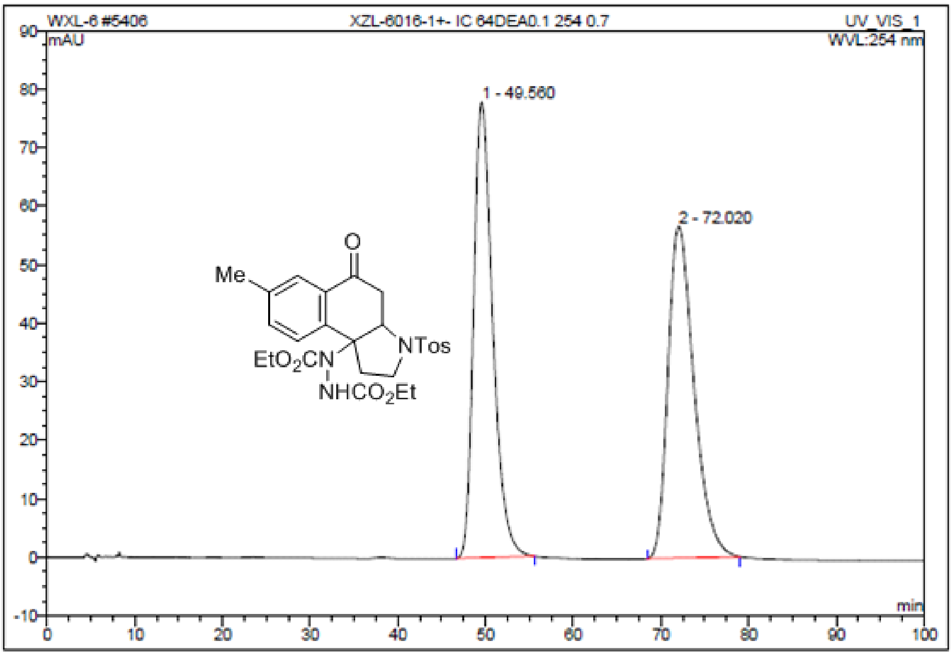

No.	Ret.Time min	Peak Name	Height mAU	Area mAU*min	Rel.Area %	Amount	Type
1	49.56	n.a.	77.839	196.194	50.19	n.a.	BMB
2	72.02	n.a.	56.677	194.714	49.81	n.a.	BMB
Total:			134.516	390.908	100.00	0.000	

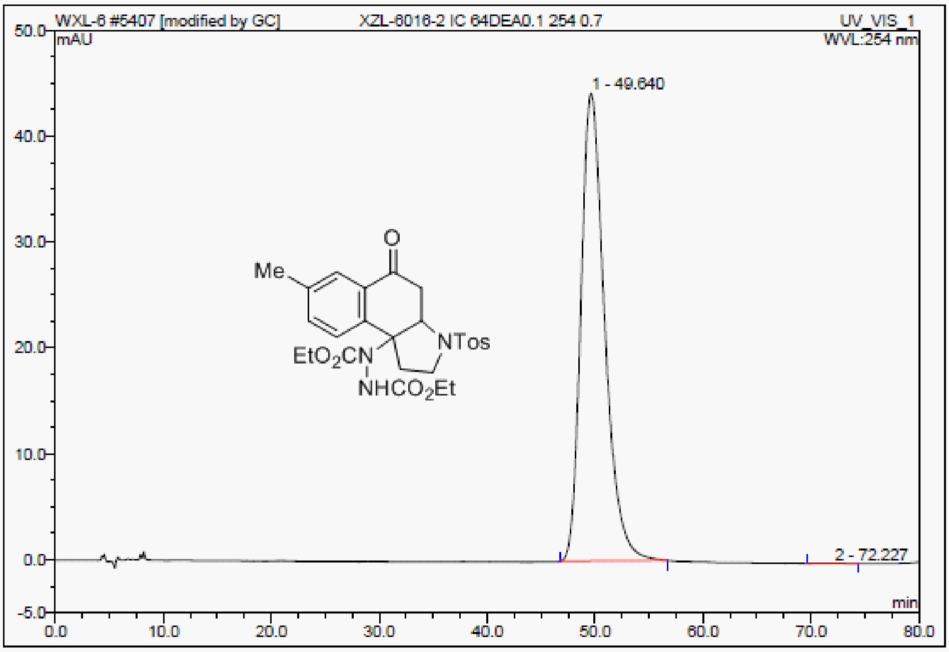

No.	Ret.Time min	Peak Name	Height mAU	Area mAU*min	Rel.Area %	Amount	Type
1	49.64	n.a.	44.140	112.091	99.92	n.a.	BMB*
2	72.23	n.a.	0.038	0.093	0.08	n.a.	BMB*
Total:			44.178	112.184	100.00	0.000	

Supplementary Figure 104 HPLC Chromatographs of **2p**

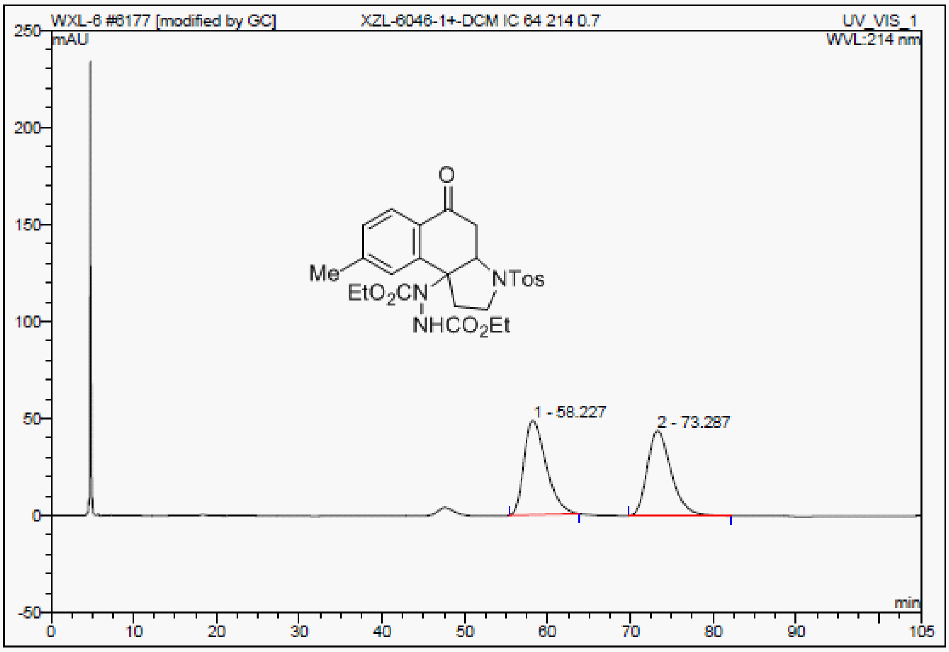

No.	Ret.Time min	Peak Name	Height mAU	Area mAU*min	Rel.Area %	Amount	Type
1	58.23	n.a.	48.510	150.904	50.60	n.a.	BMB*
2	73.29	n.a.	43.861	147.334	49.40	n.a.	BMB*
Total:			92.371	298.238	100.00	0.000	

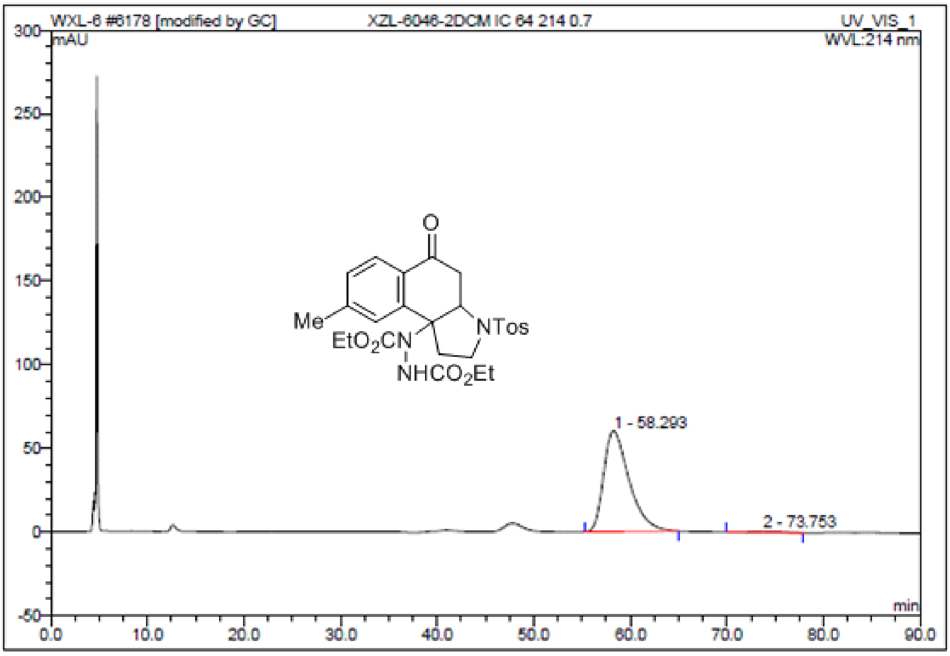

No.	Ret.Time min	Peak Name	Height mAU	Area mAU*min	Rel.Area %	Amount	Type
1	58.29	n.a.	60.276	184.639	98.80	n.a.	BMB
2	73.75	n.a.	0.604	2.240	1.20	n.a.	BMB*
Total:			60.881	186.878	100.00	0.000	

Supplementary Figure 105 HPLC Chromatographs of **2q**

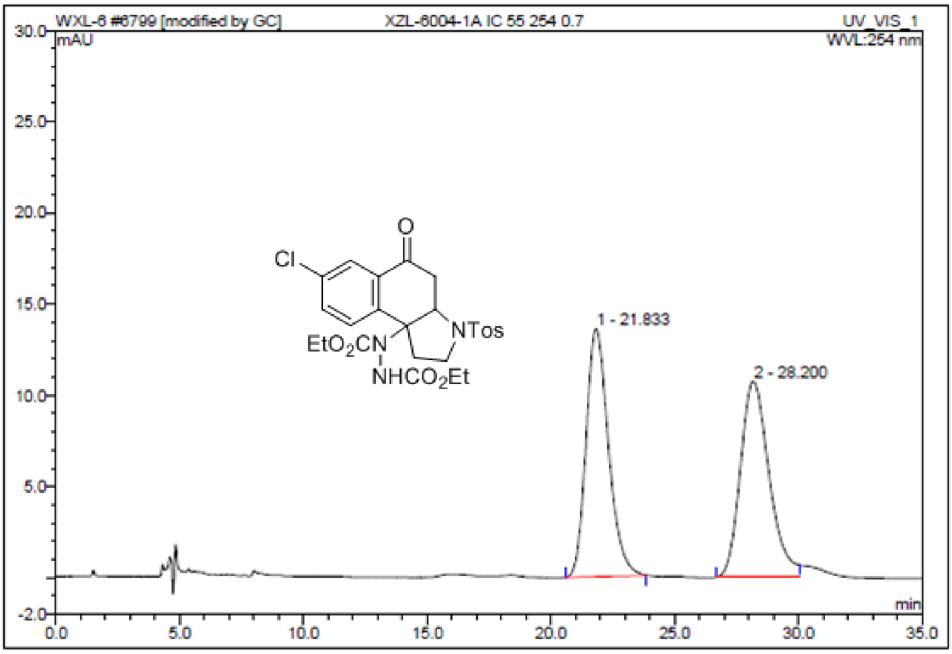

No.	Ret.Time min	Peak Name	Height mAU	Area mAU*min	Rel.Area %	Amount	Type
1	21.83	n.a.	13.591	14.296	49.69	n.a.	BMB
2	28.20	n.a.	10.753	14.472	50.31	n.a.	BM *
Total:			24.344	28.768	100.00	0.000	

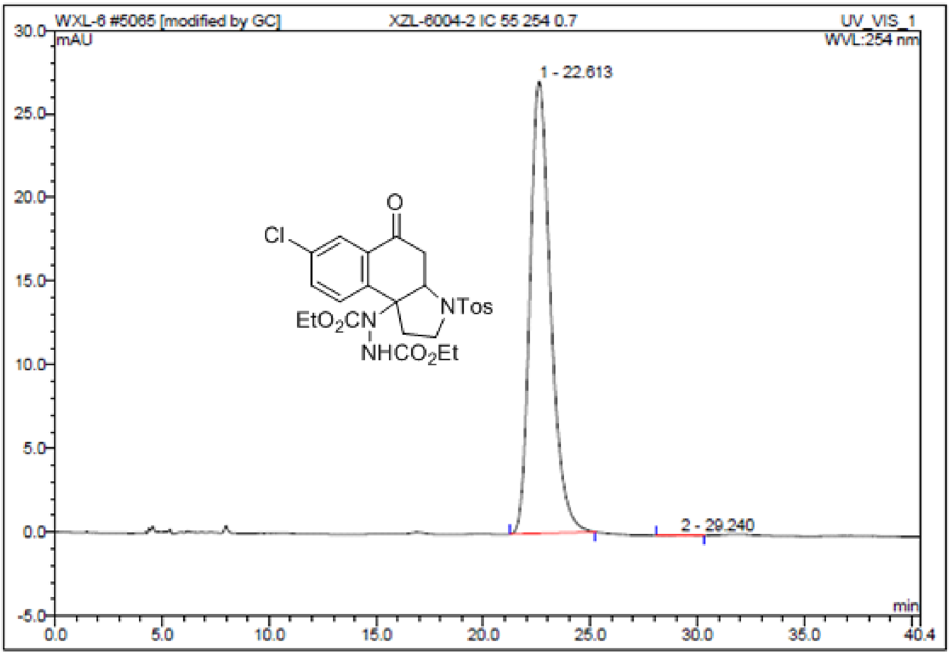

No.	Ret.Time min	Peak Name	Height mAU	Area mAU*min	Rel.Area %	Amount	Type
1	22.61	n.a.	27.028	30.163	99.95	n.a.	BMB
2	29.24	n.a.	0.019	0.015	0.05	n.a.	BM *
Total:			27.048	30.178	100.00	0.000	

Supplementary Figure 106 HPLC Chromatographs of **2r**

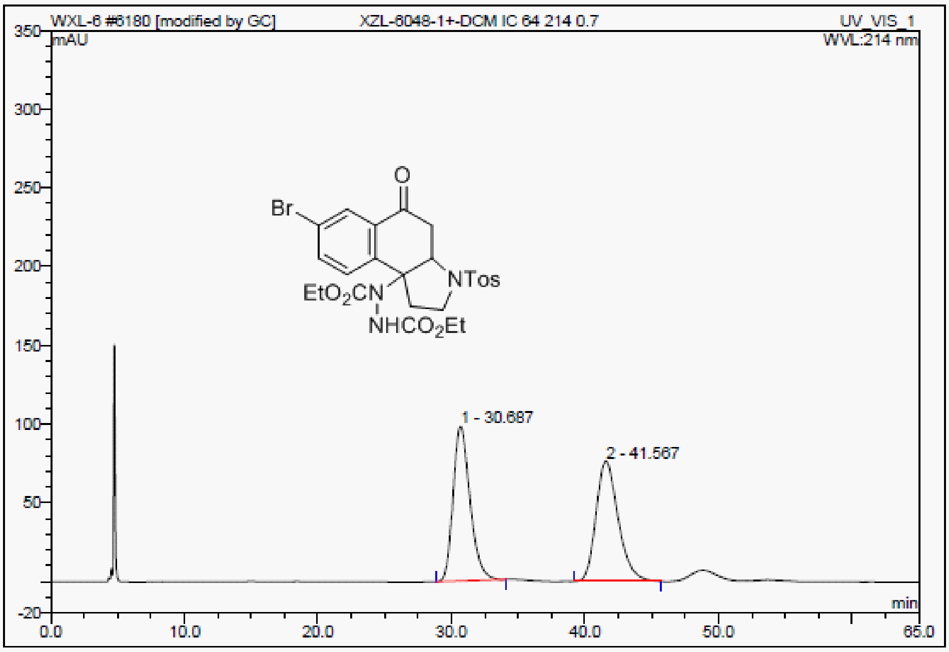

No.	Ret.Time min	Peak Name	Height mAU	Area mAU*min	Rel.Area %	Amount	Type
1	30.69	n.a.	98.105	144.054	49.57	n.a.	BMB*
2	41.57	n.a.	76.306	146.570	50.43	n.a.	BMB
Total:			174.410	290.624	100.00	0.000	

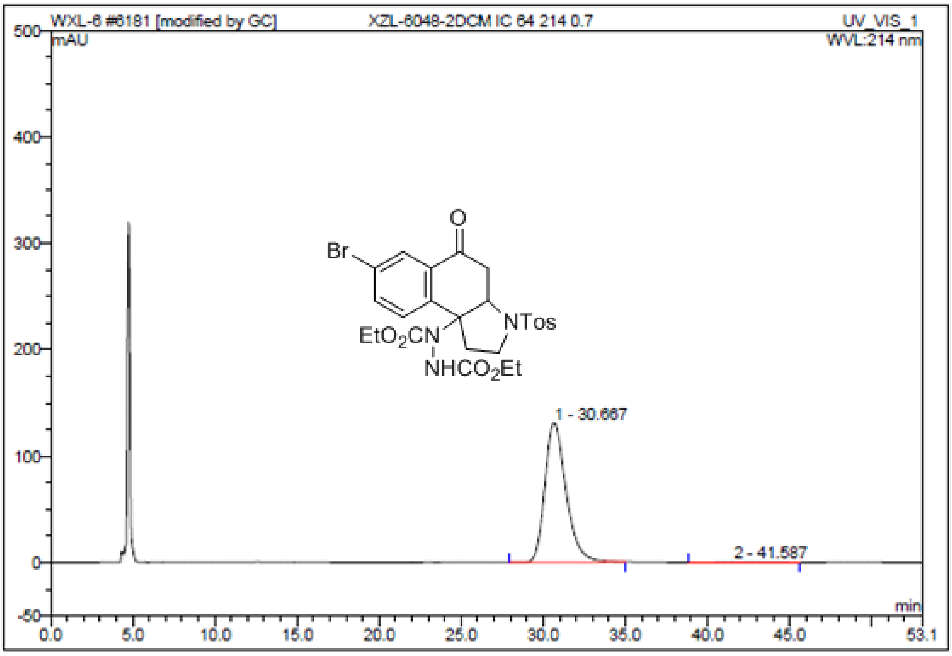

No.	Ret.Time min	Peak Name	Height mAU	Area mAU*min	Rel.Area %	Amount	Type
1	30.67	n.a.	131.067	195.926	99.63	n.a.	BMB*
2	41.59	n.a.	0.255	0.719	0.37	n.a.	BMB*
Total:			131.323	196.645	100.00	0.000	

Supplementary Figure 107 HPLC Chromatographs of **2s**

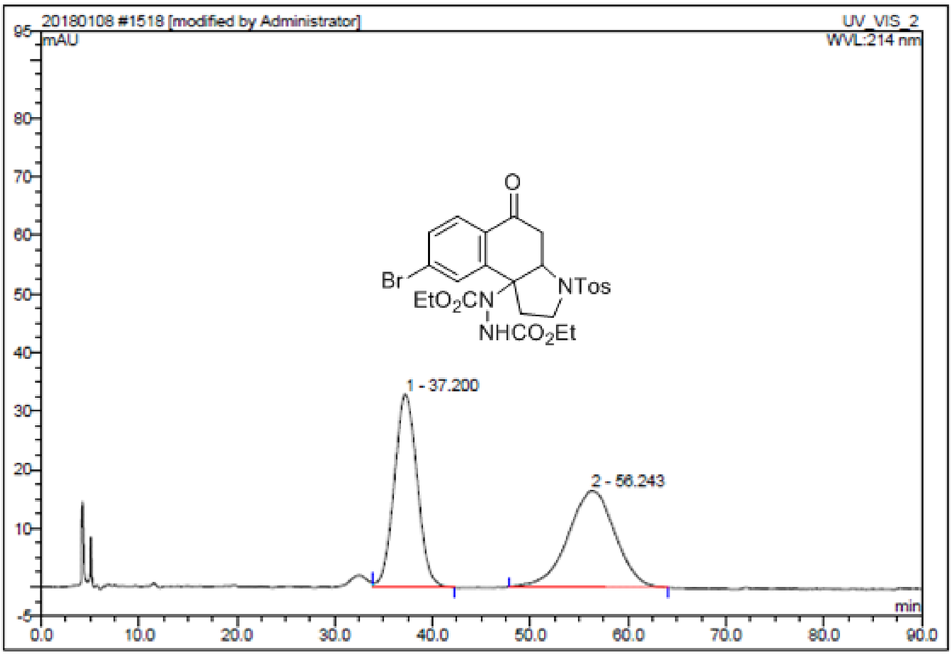

No.	Ret.Time min	Peak Name	Height mAU	Area mAU*min	Rel.Area %	Amount	Type
1	37.20	n.a.	33.026	90.140	49.71	n.a.	MB*
2	56.24	n.a.	16.460	91.208	50.29	n.a.	BMB*
Total:			49.485	181.348	100.00	0.000	

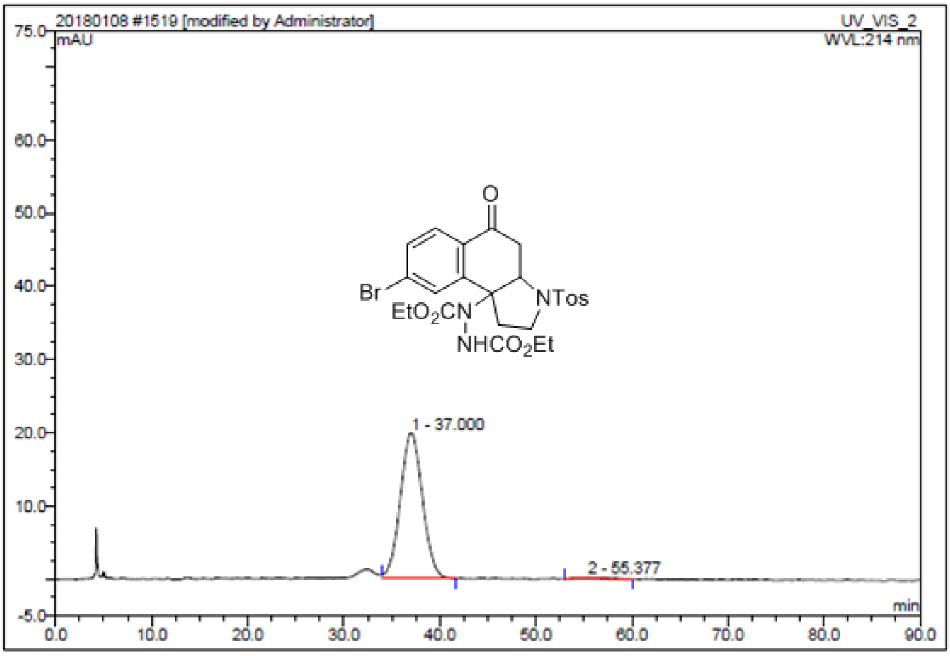

No.	Ret.Time min	Peak Name	Height mAU	Area mAU*min	Rel.Area %	Amount	Type
1	37.00	n.a.	19.943	53.423	98.82	n.a.	MB*
2	55.38	n.a.	0.176	0.638	1.18	n.a.	BMB*
Total:			20.120	54.061	100.00	0.000	

Supplementary Figure 108 HPLC Chromatographs of **2t**

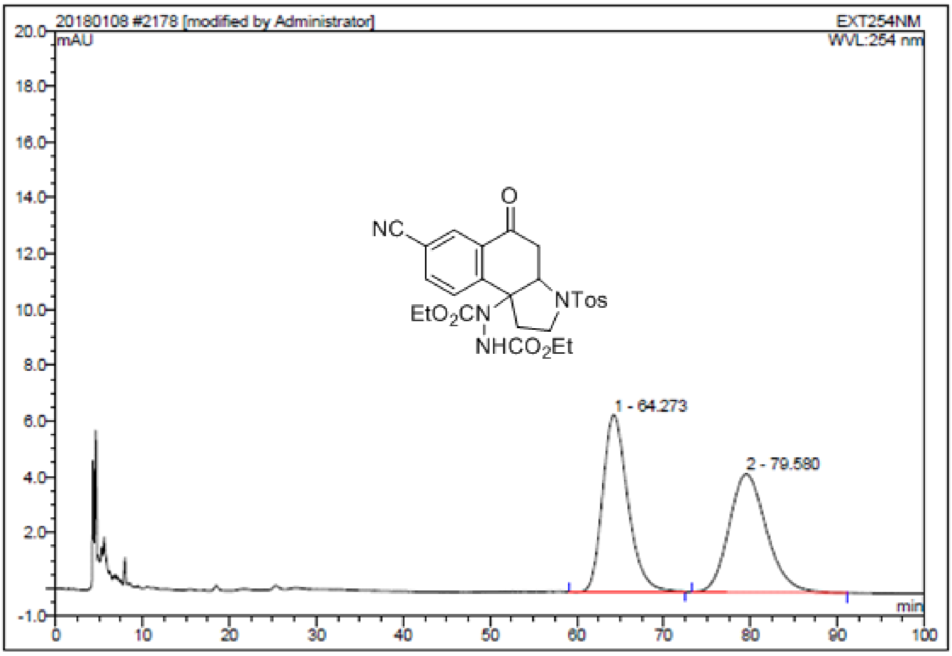

No.	Ret.Time min	Peak Name	Height mAU	Area mAU*min	Rel.Area %	Amount	Type
1	64.27	n.a.	6.351	21.399	50.26	n.a.	BMB*
2	79.58	n.a.	4.248	21.175	49.74	n.a.	BMB*
Total:			10.599	42.574	100.00	0.000	

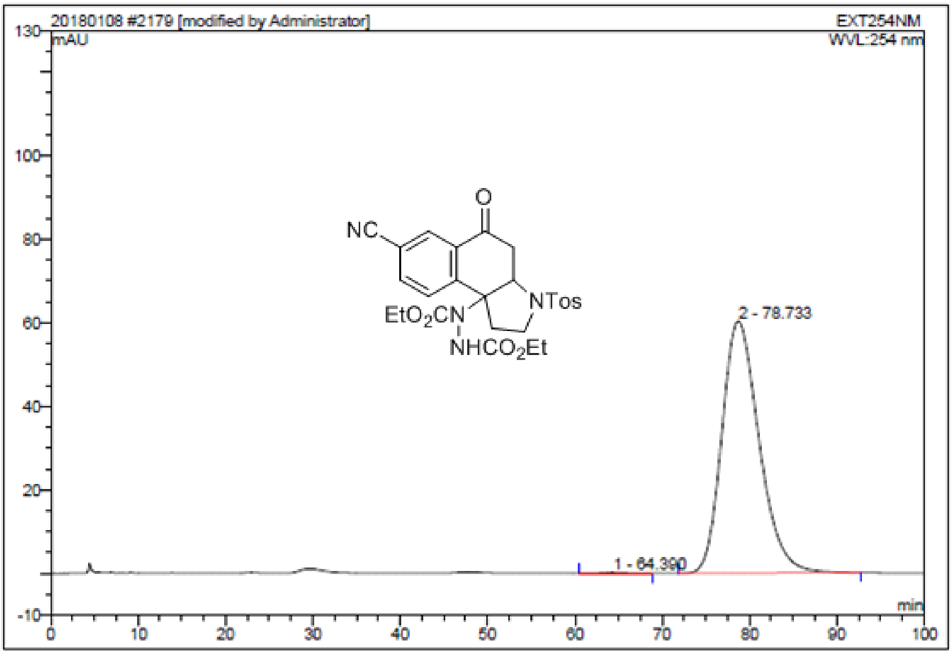

No.	Ret.Time min	Peak Name	Height mAU	Area mAU*min	Rel.Area %	Amount	Type
1	64.39	n.a.	0.228	0.728	0.24	n.a.	BMB*
2	78.73	n.a.	60.282	297.955	99.76	n.a.	BMB*
Total:			60.511	298.684	100.00	0.000	

Supplementary Figure 109 HPLC Chromatographs of **2u**

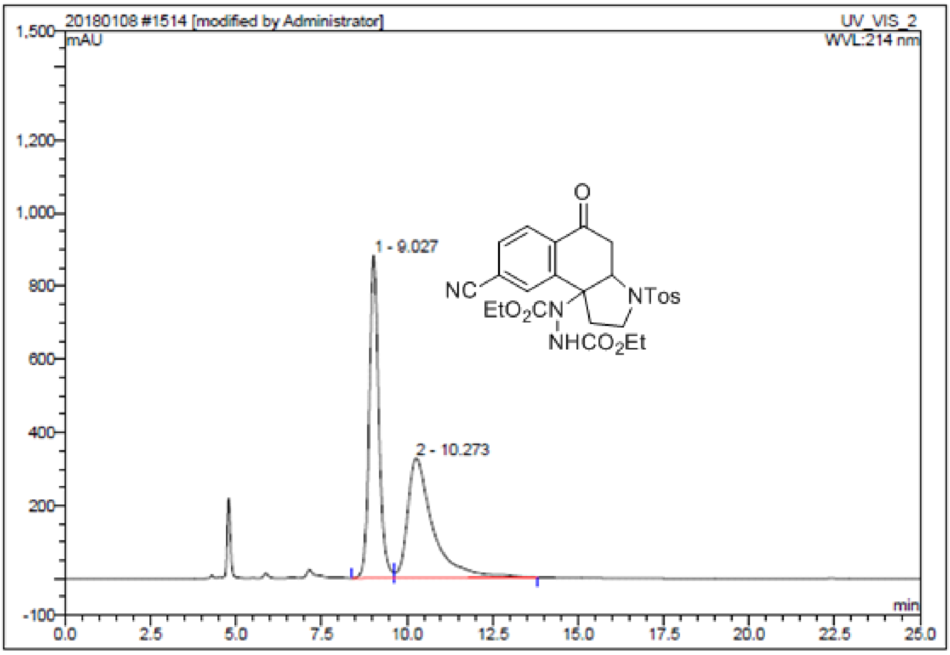

No.	Ret.Time min	Peak Name	Height mAU	Area mAU*min	Rel.Area %	Amount	Type
1	9.03	n.a.	882.037	286.060	50.63	n.a.	BM *
2	10.27	n.a.	327.045	278.991	49.37	n.a.	MB*
Total:			1209.082	565.051	100.00	0.000	

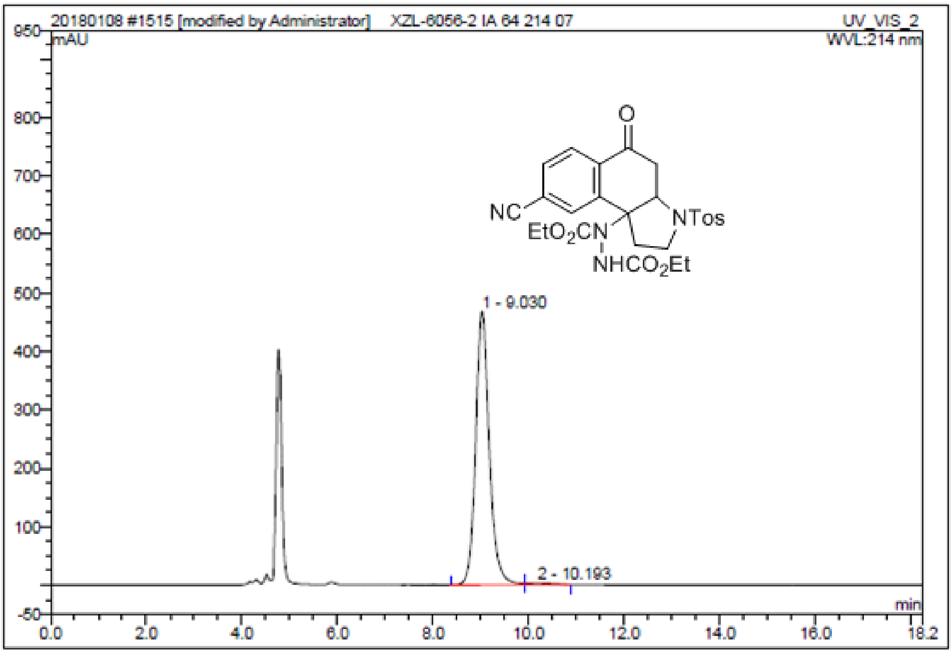

No.	Ret.Time min	Peak Name	Height mAU	Area mAU*min	Rel.Area %	Amount	Type
1	9.03	n.a.	468.316	153.858	98.99	n.a.	BM *
2	10.19	n.a.	2.722	1.563	1.01	n.a.	MB*
Total:			471.038	155.421	100.00	0.000	

Supplementary Figure 110 HPLC Chromatographs of **2v**

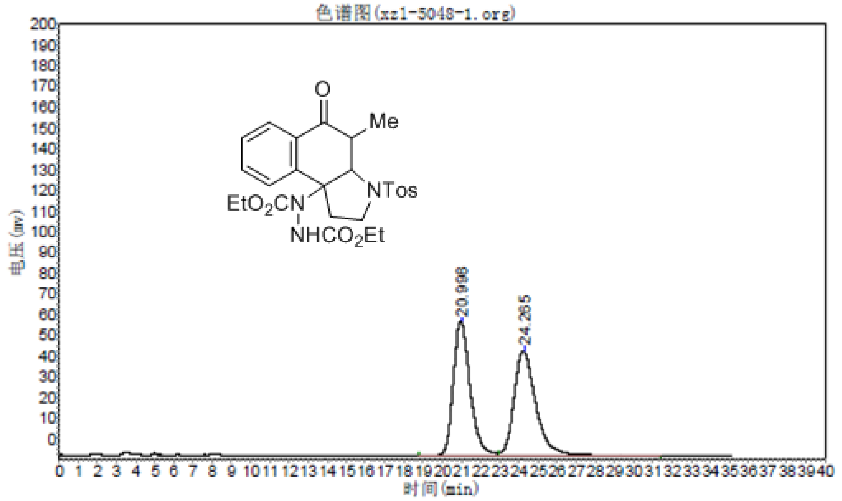

No.	RT/min	Hight	Area	Amount/%
1	20.998	64930.402	3924460.750	49.5645
2	24.265	50701.680	3993425.000	50.4355

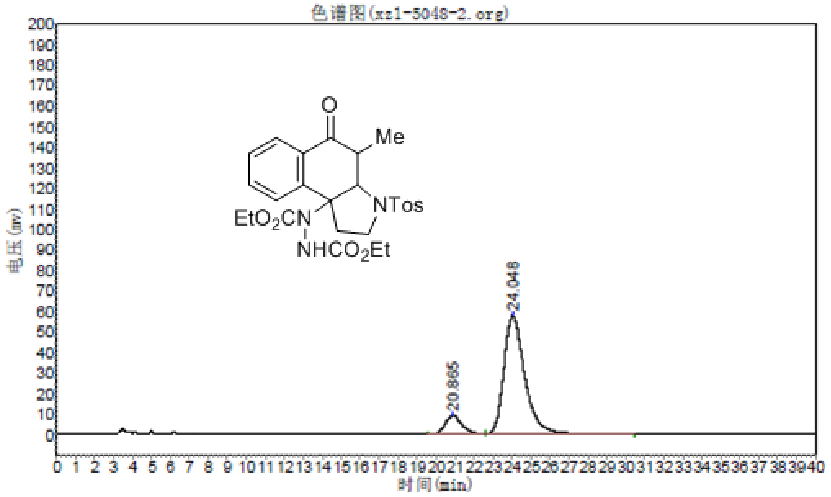

No.	RT/min	Hight	Area	Amount/%
1	20.865	8896.725	547060.625	11.0830
2	24.048	57410.523	4388963.500	88.9170

Supplementary Figure 111 HPLC Chromatographs of **4a**

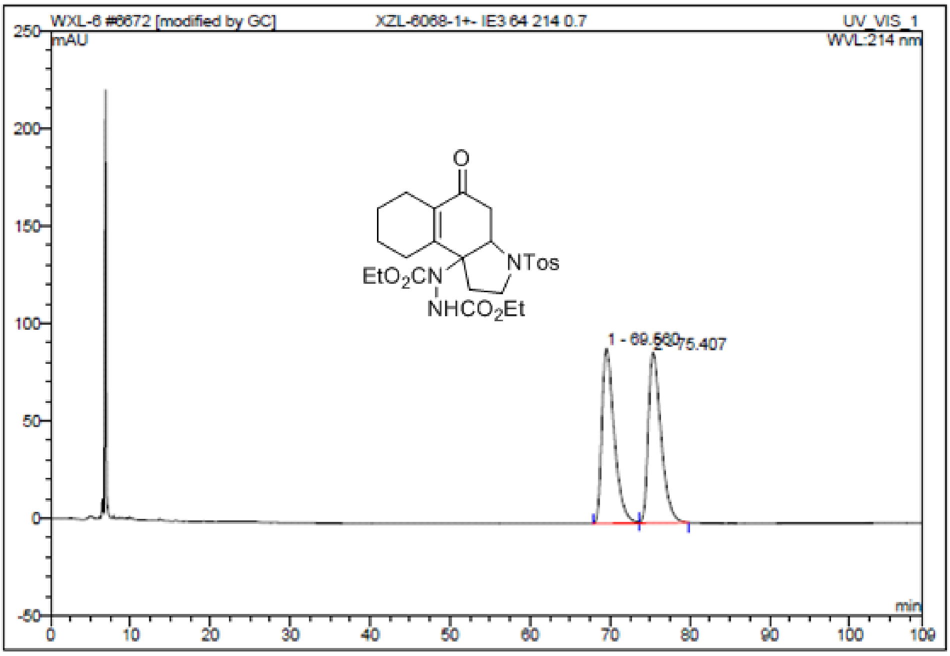

No.	Ret.Time min	Peak Name	Height mAU	Area mAU*min	Rel.Area %	Amount	Type
1	69.56	n.a.	89.706	161.652	50.06	n.a.	BM
2	75.41	n.a.	87.322	161.243	49.94	n.a.	MB
Total:			177.027	322.895	100.00	0.000	

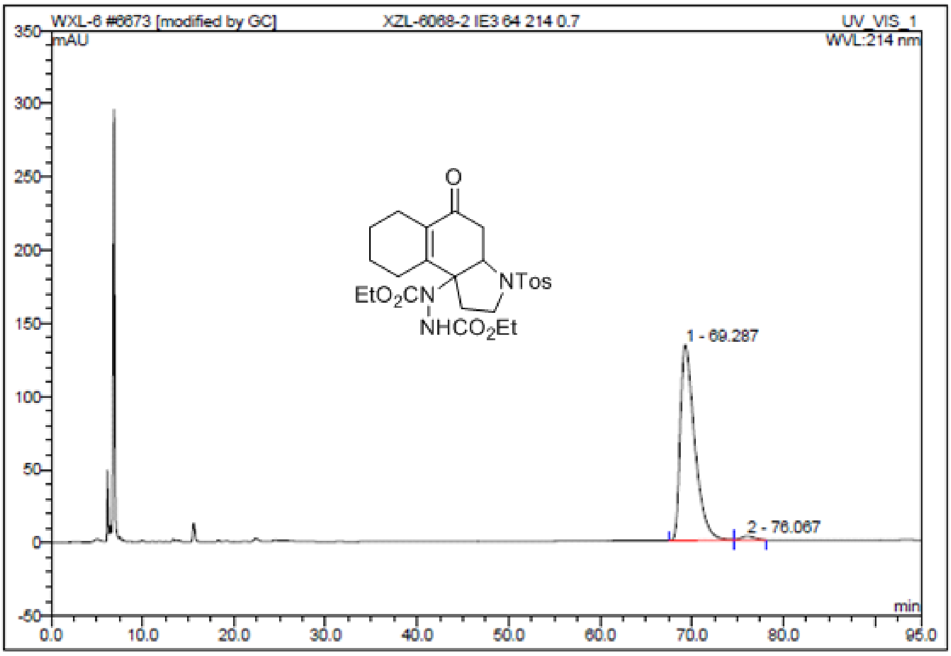

No.	Ret.Time min	Peak Name	Height mAU	Area mAU*min	Rel.Area %	Amount	Type
1	69.29	n.a.	133.546	244.776	98.17	n.a.	BM
2	76.07	n.a.	2.654	4.571	1.83	n.a.	MB
Total:			136.200	249.348	100.00	0.000	

Supplementary Figure 112 HPLC Chromatographs of **4b**

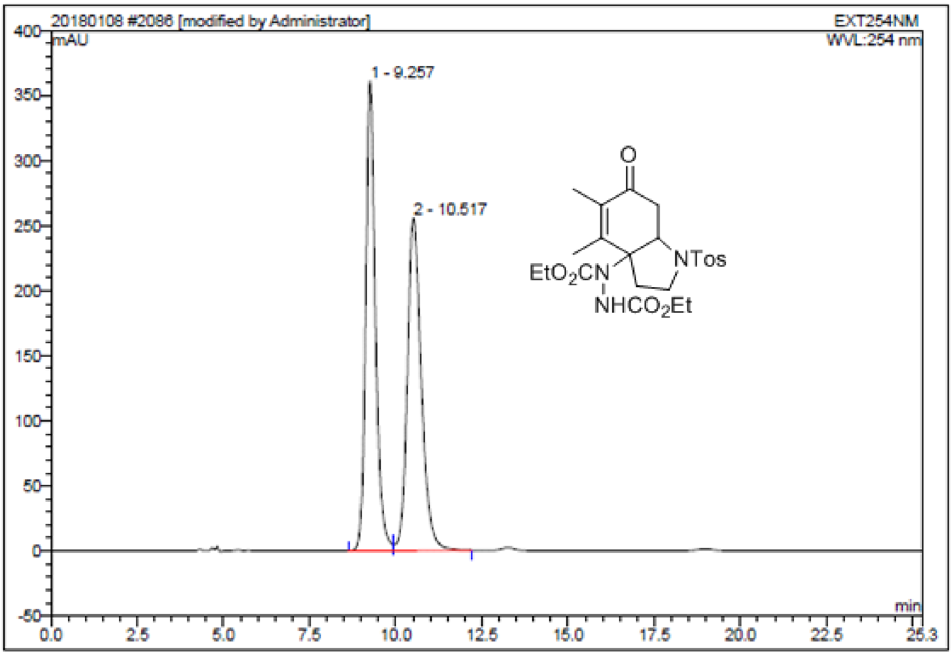

No.	Ret.Time min	Peak Name	Height mAU	Area mAU*min	Rel.Area %	Amount	Type
1	9.26	n.a.	360.912	117.934	49.55	n.a.	BM
2	10.52	n.a.	255.296	120.074	50.45	n.a.	MB
Total:			616.208	238.008	100.00	0.000	

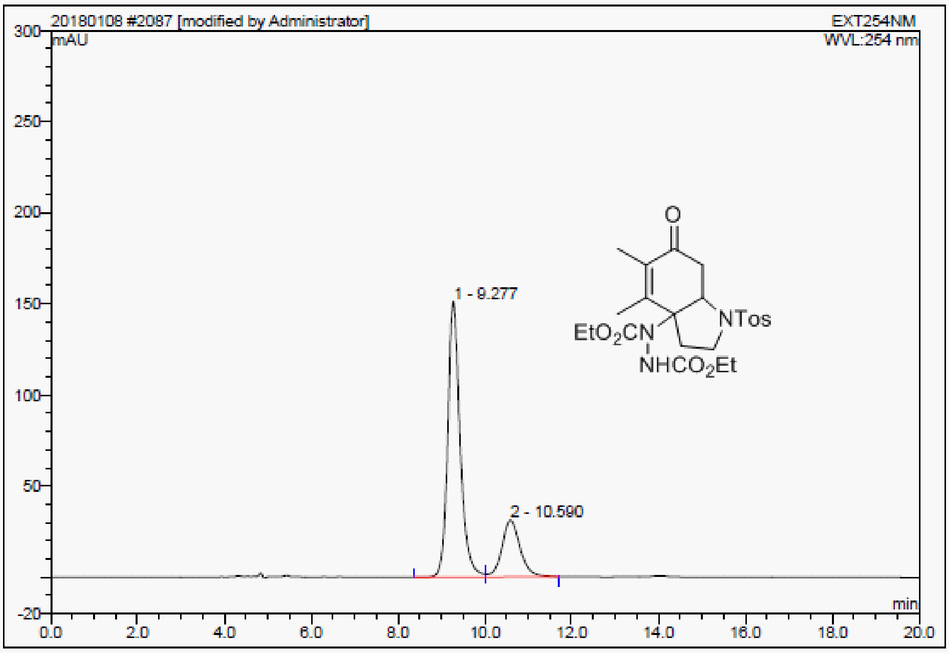

No.	Ret.Time min	Peak Name	Height mAU	Area mAU*min	Rel.Area %	Amount	Type
1	9.28	n.a.	151.073	49.878	76.71	n.a.	BM *
2	10.59	n.a.	31.184	15.141	23.29	n.a.	MB*
Total:			182.257	65.019	100.00	0.000	

Supplementary Figure 113 HPLC Chromatographs of **6**

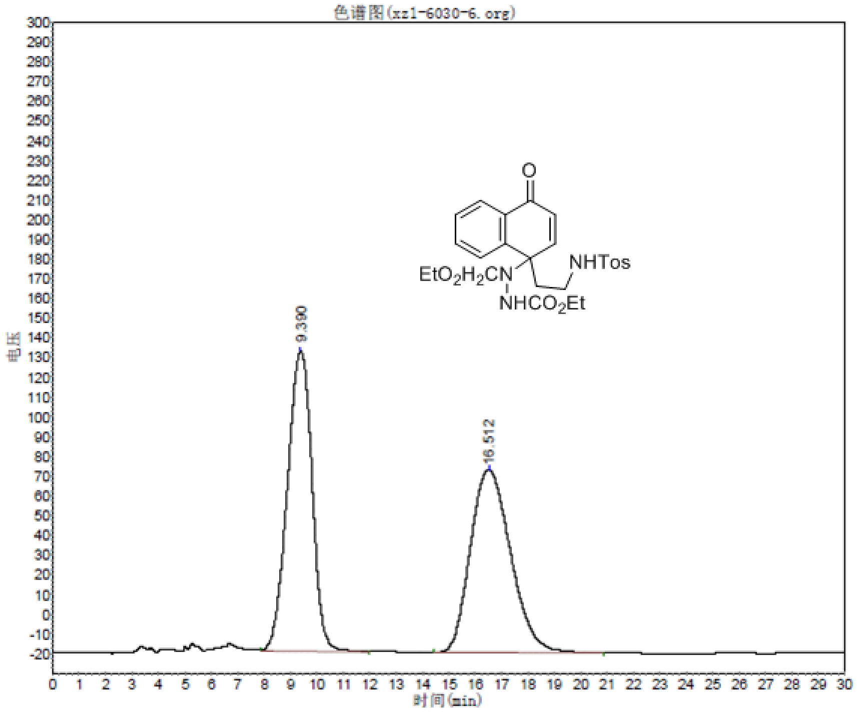

No.	RT/min	Hight	Area	Amount/%
1	9. 390	152187. 922	9888698. 000	50. 1878
2	16. 512	92794. 906	9814709. 000	49. 8122

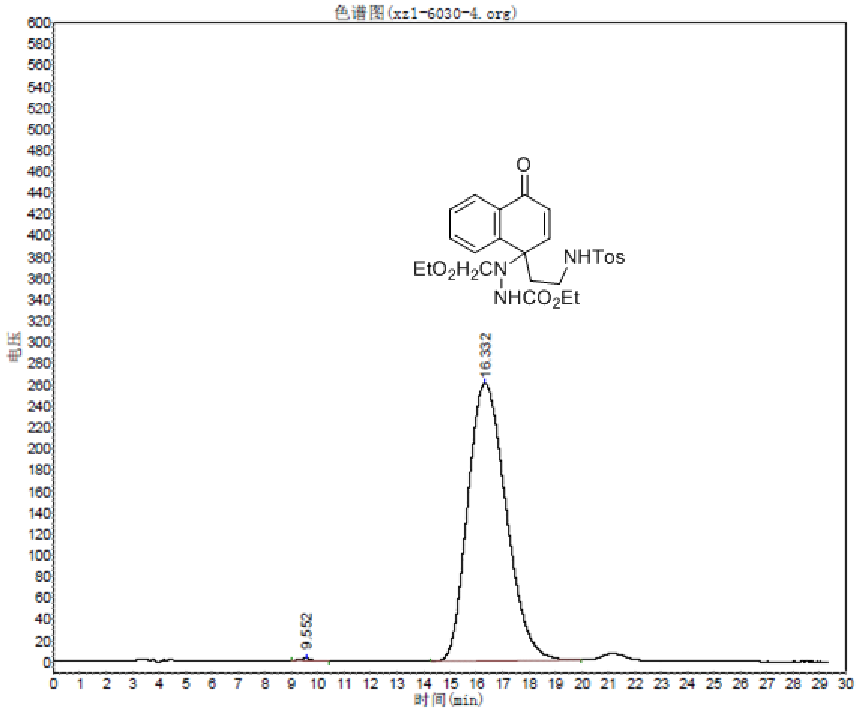

No.	RT/min	Hight	Area	Amount/%
1	9. 552	2553. 440	53474. 895	0. 2033
2	16. 332	260437. 250	26250856. 000	99. 7967

Supplementary Figure 114 HPLC Chromatographs of **7**

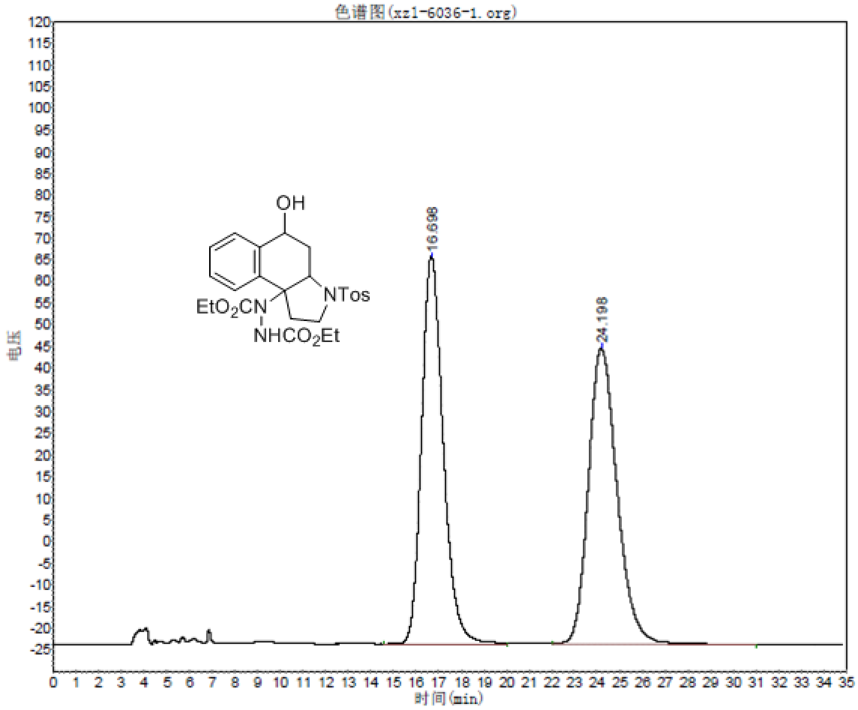

No.	RT/min	Hight	Area	Amount/%
1	16.698	89556.234	5962889.000	49.5444
2	24.198	68294.922	6072551.000	50.4556

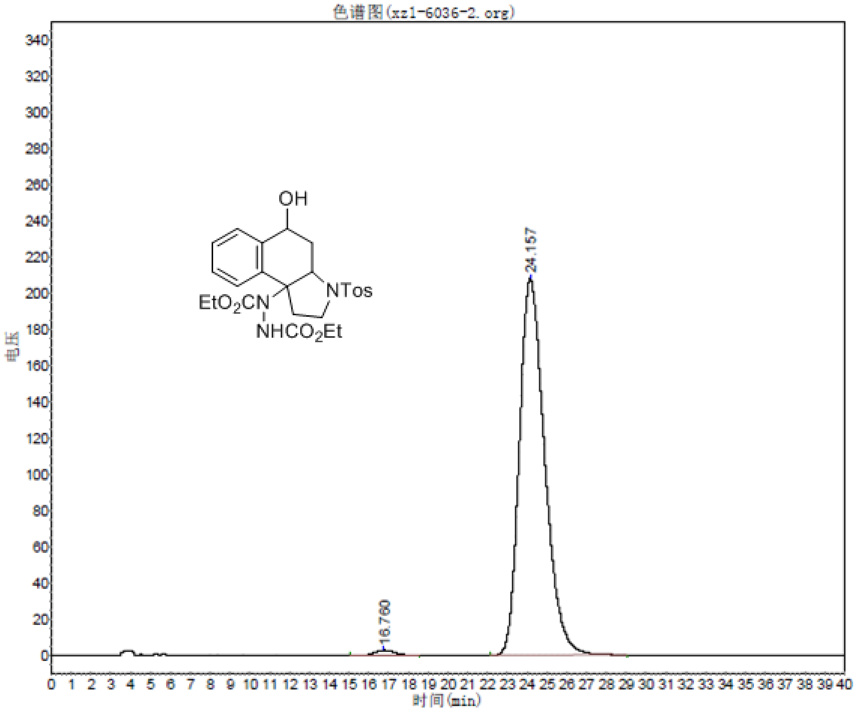

No.	RT/min	Hight	Area	Amount/%
1	16.760	2655.146	176307.297	0.9447
2	24.157	207975.578	18487226.000	99.0553

Supplementary Figure 115 HPLC Chromatographs of **8**

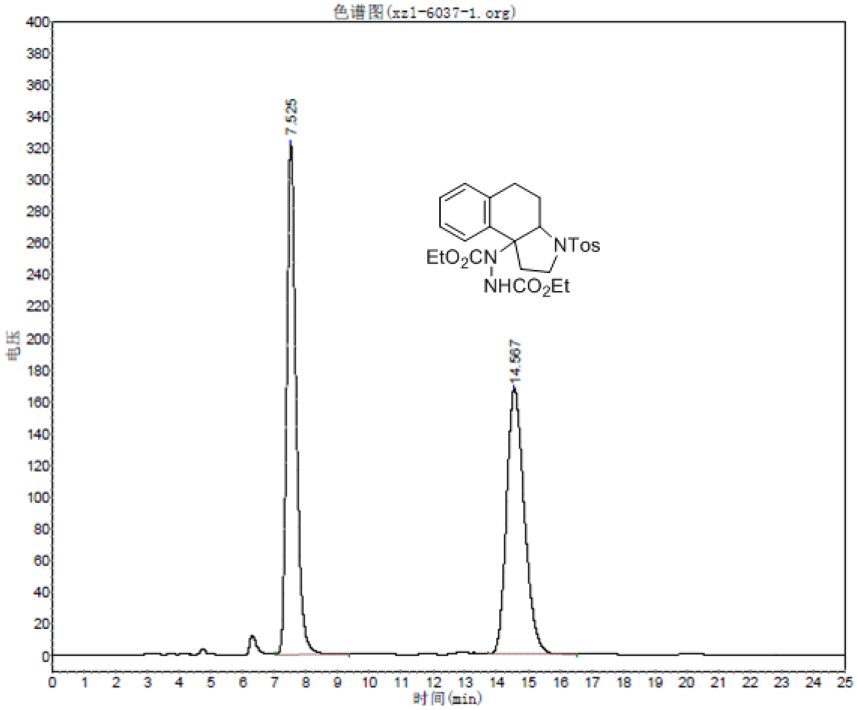

No.	RT/min	Hight	Area	Amount/%
1	7.525	322274.406	6675691.000	50.4317
2	14.567	167111.016	6561406.500	49.5683

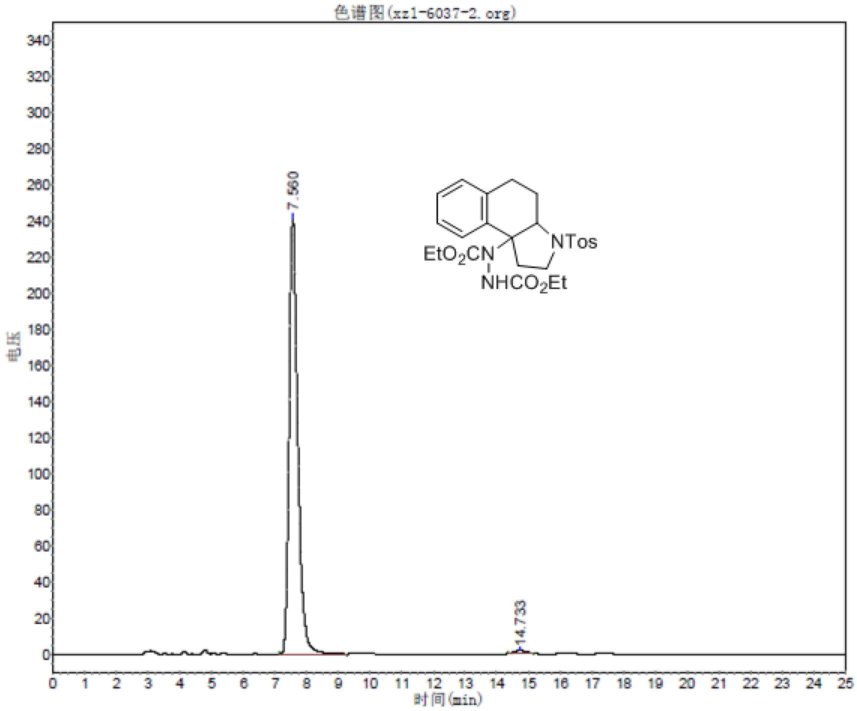

No.	RT/min	Hight	Area	Amount/%
1	7.560	241621.219	4609380.000	99.1937
2	14.733	1369.019	37465.703	0.8063

Supplementary Figure 116 HPLC Chromatographs of **9**

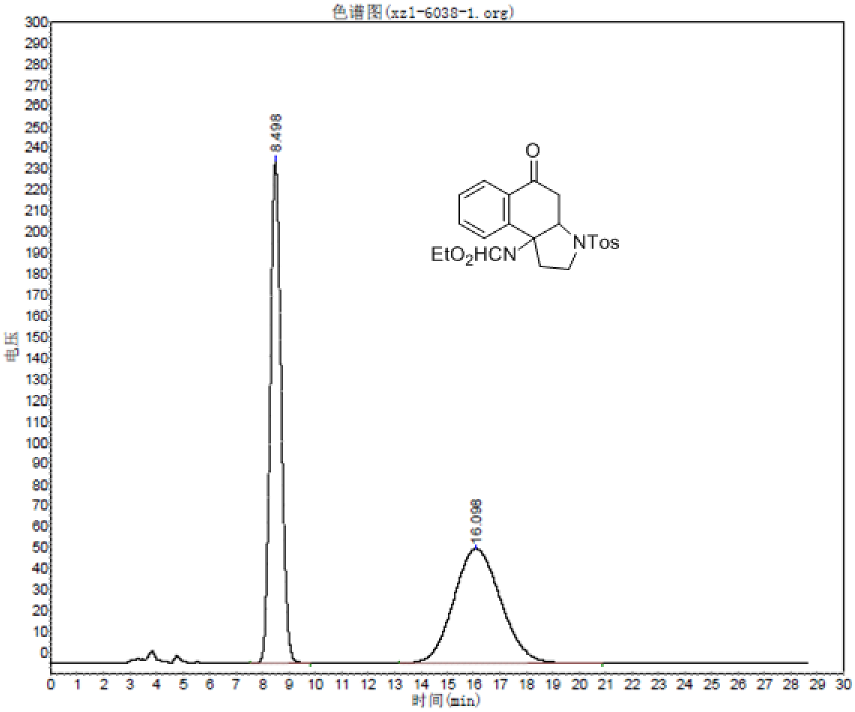

No.	RT/min	Hight	Area	Amount/%
1	8.498	239279.094	6915176.000	50.5850
2	16.098	54512.480	6755222.000	49.4150

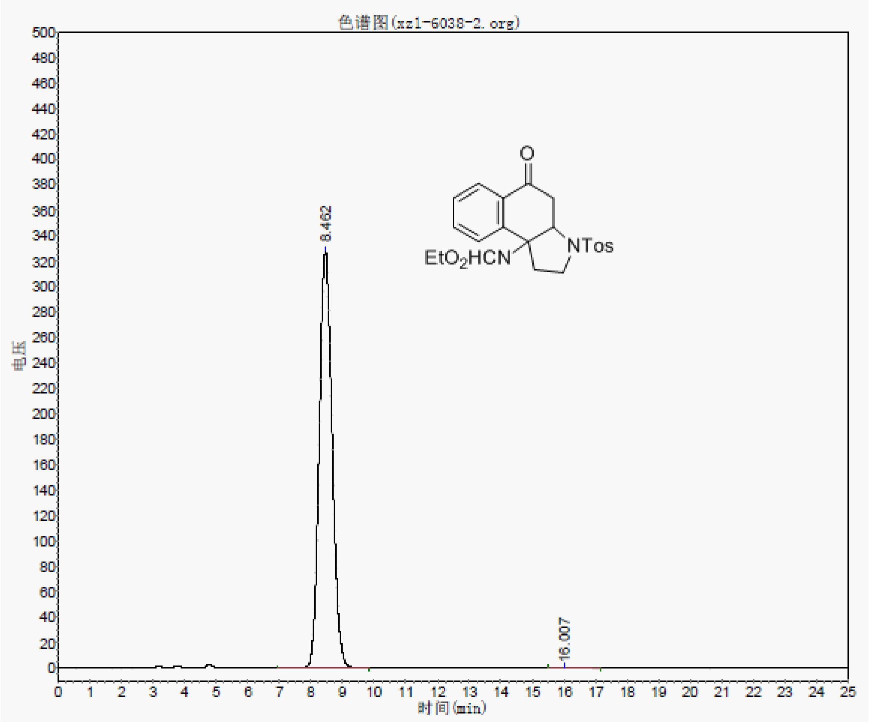

No.	RT/min	Hight	Area	Amount/%
1	8.462	328019.219	9303388.000	99.9391
2	16.007	94.450	5664.801	0.0609

X-Ray of **2a** (CCDC 1888563)

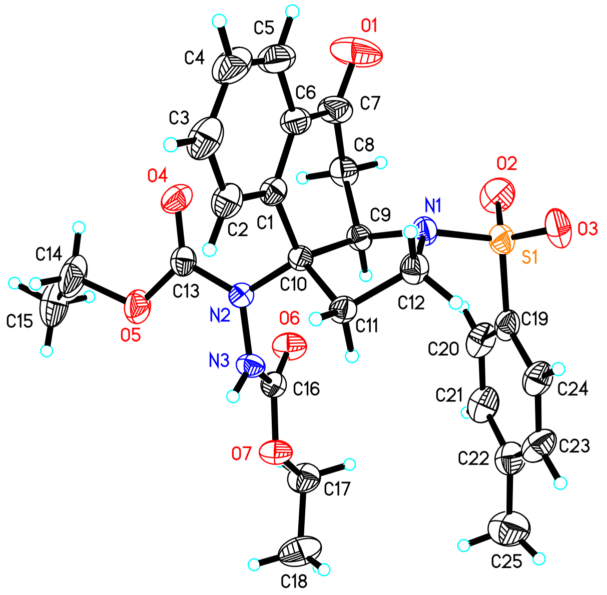

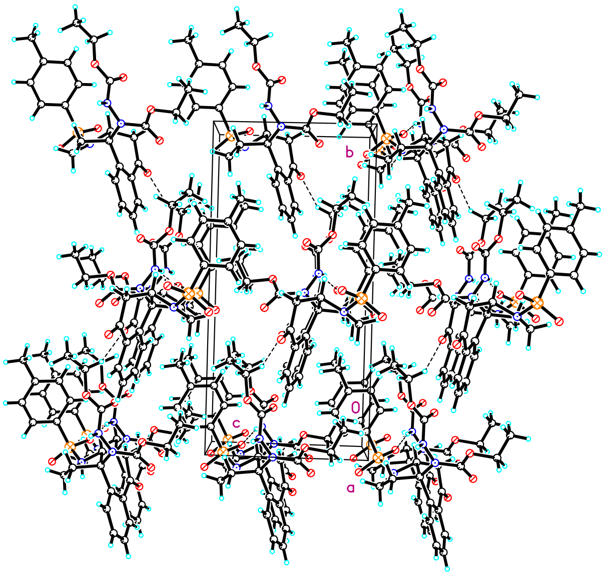

Supplementary Table 1. Crystal data and structure refinement for mo_d8v17430_0m.

Identification code	mo_d8v17430_0m	
Empirical formula	C25 H29 N3 O7 S	
Formula weight	515.57	
Temperature	296(2) K	
Wavelength	0.71073 Å	
Crystal system	Monoclinic	
Space group	P 21	
Unit cell dimensions	a = 8.1363(3) Å	α= 90°.
	b = 17.8021(7) Å	β= 94.7700(10)°.
	c = 8.7245(3) Å	γ = 90°.
Volume	1259.31(8) Å^3	
Z	2	
Density (calculated)	1.360 Mg/m^3	
Absorption coefficient	0.178 mm^{-1}	
F(000)	544	
Crystal size	0.180 x 0.150 x 0.100 mm^3	
Theta range for data collection	2.760 to 25.486°.	
Index ranges	-9<=h<=9, -21<=k<=21, -10<=l<=10	
Reflections collected	20598	
Independent reflections	4636 [R(int) = 0.0262]	
Completeness to theta = 25.242°	99.0 %	

Absorption correction	Semi-empirical from equivalents
Max. and min. transmission	0.7456 and 0.6882
Refinement method	Full-matrix least-squares on F^2
Data / restraints / parameters	4636 / 1 / 333
Goodness-of-fit on F^2	1.033
Final R indices [I>2sigma(I)]	R1 = 0.0316, wR2 = 0.0797
R indices (all data)	R1 = 0.0348, wR2 = 0.0829
Absolute structure parameter	0.016(19)
Extinction coefficient	0.038(9)
Largest diff. peak and hole	0.175 and -0.206 e.$Å^{-3}$

DFT Calculations

Computational Methods

All the calculations in this study were performed with Gaussian16 package.[13] The density functional theory (DFT) method was employed using the B97D functional.[14] The standard 6-31G** basis sets implemented in Gaussian were applied for all atoms. Optimizations were conducted without any constraint using implicit solvation model (SMD)[15] in 1,2-dichloroethane ($\varepsilon = 10.125$). In order to avoid the narrow crevices and channels during the building of solute cavities that might introduce artifacts such as the abnormal SCF energies and/or failures in the inversion of the PCM matrix, solvent-accessible surface (SAS) was employed. Frequency analyses (at 323.15 K and 1 atm) were carried out to confirm each structure being a minimum (no imaginary frequency) or a transition state (only one imaginary frequency). The energies were further estimated by single-point calculations using ωB97XD functional with the polarized, triple-ζ valence quality def2-TZVPP basis set of Weigend and Ahlrich[16] for all atoms in 1,2-dichloroethane (SMD model with default van der Waals surface). The B97D and ωB97XD functionals were known to provide reliable relative free energies for many asymmetric reactions where noncovalent interactions are important.[17] The Gibbs free energies in 1,2-dichloroethane (ΔG), including the single-point corrections, were discussed throughout this paper. The 3D structures shown in Figue 1 of the main text were prepared using VMD.[18]

The origin of enantioselectivity

According to the preliminary experimental results, the aminative dearomatization step was identified as the stereochemistry-determining step. In order to provide deep insights into the origin of the enantioselectivity of this reaction, an array of transition states of this step of the reaction between **1a** and DEAD by (*S*)-**C2** were located. It is well known that chiral phosphoric acid can act as a bifunctional catalyst which interacts both substrates with hydrogen bonding.[19-22] The C4 position of the *α*-naphthol ring can attack DEAD *via* its *Si*- or *Re*-face, leading to either (3*R*,4*R*)-**2a** or (3*S*,4*S*)-**2a**. In each series (*Si*- or *Re*-face attack), four transition states with different conformation of DEAD were considered (Supplementary Figure 117). **TS-*Si*-C** (0.0 kcal/mol) and **TS-*Re*-C** (2.0 kcal/mol) (referred to as **TS-major** and **TS-minor** in the main text) were found as the most stabilized transition states leading to (3*R*,4*R*)-**2a** (major enantiomer) and (3*S*,4*S*)-**2a** (minor enantiomer), respectively. These results correspond well with the experimental observations. The high enantioselectivity could be attributed to the excellent discrimination of the prochiral faces of the C4 position of the *α*-naphthol ring by the chiral phosphoric acid in that in **TS-major**, the *Si*-face attack allowed the intact benzene ring of *α*-naphthol in distal to the chiral pocket. Whereas in **TS-minor**, strong steric congestion was developed due to the *Re*-face attack and the concomitant approximation of the intact benzene ring of *α*-naphthol to one TRIP group of **C2**. A working model accounting for the origination of the stereochemical induction process is shown in Supplementary Figure 118.

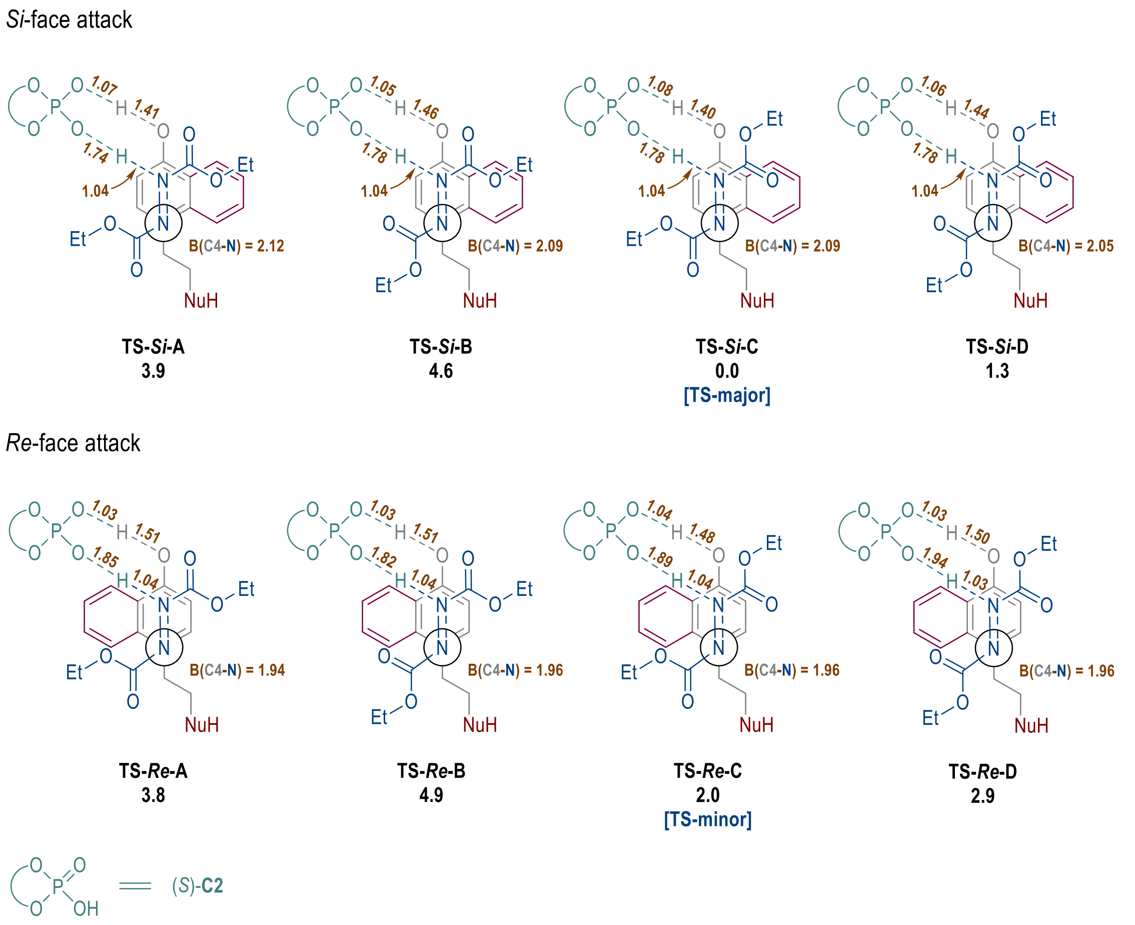

Supplementary Figure 117 The schematic description of the transition states of the aminative dearomatization step of the reaction between **1a** and DEAD by (*S*)-**C2**. Calculated at ωB97XD/def2-TZVPP//B97D/6-31G** level of theory. The relative Gibbs free energy (Δ*G*) are in kcal/mol and selected bond distances are in angstrom.

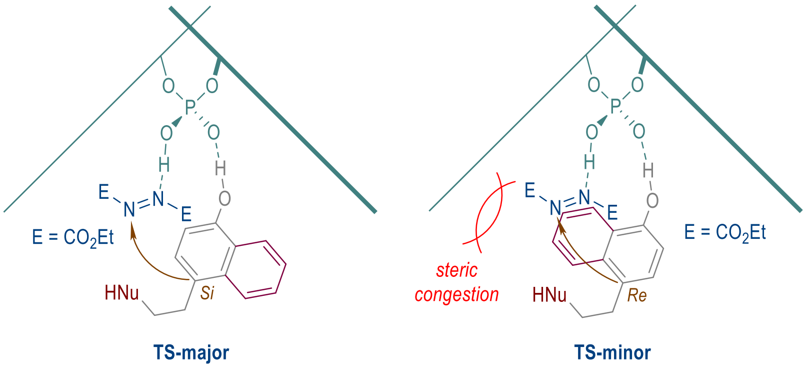

Supplementary Figure 118 A working model describing the origin of the enantioselectivity.

Supplementary References

1. Xu, R.-Q., Gu, Q. & You, S.-L. Construction of the benzomesembrine skeleton: palladium(0)-catalyzed intermolecular arylative dearomatization of α-naphthols and subsequent aza-Michael reaction. *Angew. Chem., Int. Ed*. **56**, 7252-7256 (2017).
2. Antien, K., Viault, G., Pouységu, L., Peixoto, P. A. & Quideau, S. Asymmetric dearomative spirolactonization of naphthols using λ3-iodanes under chiral phase-transfer catalysis. *Tetrahedron* **73**, 3684-3690 (2017).
3. Fan, Y., Feng, P., Liu, M., Pan, H. & Shi, Y. A concise approach to the dalesconol skeleton. *Org. Lett.* **13**, 4494-4497 (2011).
4. Synder, S. A., Sherwood, T. C. & Ross, A. G. Total syntheses of dalesconol A and B. *Angew. Chem., Int. Ed.* **49**, 5146-5150 (2010).
5. Hulme, A. N., Henry, S. S. & Meyers, A. I. Asymmetric synthesis of the key intermediates leading to (-)-Aphanorphine and (-)-Eptazocine. *J. Org. Chem.* **60**, 1265-1270 (1995).
6. Batt, D. G., Jones, D. G. & Greca, S. L. Regioselectivity in the acid-catalyzed isomerization of 2-substituted 1,4-dihydro-1,4-epoxynaphthalenes. *J. Org. Chem.* **56**, 6704-6708 (1991).
7. Mayer, P., Alibert, S., Brunel, P. & Imbert, T. Synthesis of benzo[de]chromene analogue of efaroxan. *Heterocycles*, **55**, 387-392 (2001).
8. Shen, Q., Qian, Y., Huang, X., Xu, X., Li, W., Liu, J. & Fu, W. Discovery of potent and selective agonists of δ opioid receptor by revisiting the "Message-Address" concept. *ACS Med. Chem. Lett.* **7**, 391-396 (2016).
9. Lee, W.-G., Frey, K. M., Gallardo-Macias, R., Spasov, K. A., Bollini, M., Anderson, K. S. & Jorgensen, W. L. Picomolar inhibitors of HIV-1 reverse transcriptase: design and crystallography

of naphthyl phenyl ethers. *ACS Med. Chem. Lett.* **5**, 1259-1262 (2014).

10. ARRAY BIOPHARMA INC. WO2009/158426, 2009, A1.

11. Yang, C.-T., Han, J., Liu, J., Liu, J., Gu, M., Li, Y., Wen, J., Yu, H.-Z., Hu, S. & Wang, X. “One-pot” synthesis of amidoxime viaPd-catalyzed cyanation and amidoximation. *Org. Biomol. Chem.* **13**, 2541-2545 (2015).

12. Magnus, P., Garizi, N., Seibert, K. A. & Ornholt, A. Synthesis of carbamates from diethoxycarbonyl hydrazine derivatives by E1cB dliminative cleavage of the N-N′-bond rather than reduction. *Org. Lett.* **11**, 5646-5648 (2009).

13. Gaussian 16, Revision A.03, Frisch, M. J., Trucks, G. W., Schlegel, H. B., Scuseria, G. E., Robb, M. A., Cheeseman, J. R., Scalmani, G., Barone, V., Pe-tersson, G. A., Nakatsuji, H., Li, X., Caricato, M., Marenich, A. V., Bloino, J., Janesko, B. G., Gomperts, R., Mennucci, B., Hratchian, H. P., Ortiz, J. V., Iz-maylov, A. F., Sonnenberg, J. L., Williams-Young, D., Ding, F., Lipparini, F., Egidi, F., Goings, J., Peng, B., Petrone, A., Henderson, T., Ranasinghe, D., Zakrzewski, V. G., Gao, J., Rega, N., Zheng, G., Liang, W., Hada, M., Ehara, M., Toyota, K., Fukuda, R., Hasegawa, J., Ishida, M., Nakajima, T., Honda, Y., Kitao, O., Nakai, H., Vreven, T., Throssell, K., Montgomery, J. A., Jr., Peralta, J. E., Ogliaro, F., Bearpark, M. J., Heyd, J. J., Brothers, E. N., Kudin, K. N., Staroverov, V. N., Keith, T. A., Kobayashi, R., Normand, J., Raghavachari, K., Rendell, A. P., Burant, J. C., Iyengar, S. S., Tomasi, J., Cossi, M., Millam, J. M., Klene, M., Adamo, C., Cammi, R., Ochterski, J. W., Martin, R. L., Morokuma, K., Farkas, O., Foresman, J. B. & Fox, D. J. Gaussian, Inc., Wallingford CT, 2016.

14. Grimme, S. Semiempirical GGA-type density functional constructed with a long-range dispersion correction. *J. Comp. Chem.* **27**, 1787-1799 (2006).

15. Marenich, A. V., Cramer, C. J. & Truhlar, D. G. Universal solvation model based on solute electron density and on a continuum model of the solvent defined by the bulk dielectric constant and atomic surface tensions. *J. Phys. Chem. B* **13**, 6378-6396 (2009).

16. Weigend, F. & Ahlrichs, R. Balanced basis sets of split valence, triple zeta valence and quadruple zeta valence quality for H to Rn: Design and assessment of accuracy. *Phys. Chem. Chem. Phys.* **7**, 3297-3305 (2005).

17. Wheeler, S. E., Seguin, T. J., Guan, Y. & Doney, A. C. Noncovalent interactions in organocatalysis and the prospect of computational catalyst design. *Acc. Chem. Res.* **49**, 1061-1069 (2016).

18. Humphrey, W., Dalke, A. & Schulten, K. VMD: visual molecular dynamics. *J. Molec. Graphics* **14**, 33-38 (1996).

19. Reid, J. P., Simón, L. & Goodman, J. M. A practical guide for predicting the stereochemistry of bifunctional phosphoric acid catalyzed reactions of imines. *Acc. Chem. Res.* **49**, 1029-1041 (2016).

20. Reid, J. P. & Goodman, J. M. Goldilocks catalysts: computational insights into the role of the 3,3′ substituents on the selectivity of BINOL-derived phosphoric acid catalysts. *J. Am. Chem. Soc.* **138**, 7910-7917 (2016).

21. Reid, J. P. & Goodman, J. M. Selecting chiral BINOL-derived phosphoric acid catalysts: general model to identify steric features essential for enantioselectivity. *Chem. Eur. J.* **23**, 14248-14260 (2017).

22. Maji, R., Mallojjala, S. C. & Wheeler, S. E. Chiral phosphoric acid catalysis: from numbers to insights. *Chem. Soc. Rev.* **47**, 1142-1158 (2018).
