# Supplementary material for: Chiral phosphoric acid catalyzed aminative dearomatization of α-naphthols/Michael addition sequence
Source: Nat Commun. 2019 Jul 17;10:3150. doi: 10.1038/s41467-019-11109-9 (PMC6637135; doi:10.1038/s41467-019-11109-9)
Supplement: Supplementary file 2 — Description of Additional Supplementary Files [file 41467_2019_11109_MOESM2_ESM.pdf]

Description of Additional Supplementary Files

File Name: Supplementary Data 1

Description: Cartesian coordinates and energies of all optimized structures
