# Supplementary material for: Chiral phosphoric acid catalyzed aminative dearomatization of α-naphthols/Michael addition sequence
Source: Nat Commun. 2019 Jul 17;10:3150. doi: 10.1038/s41467-019-11109-9 (PMC6637135; doi:10.1038/s41467-019-11109-9)
Supplement: Supplementary file 3 — Supplementary Data 1 [file 41467_2019_11109_MOESM3_ESM.pdf]

Cartesian coordinates and energies and of all optimized structures

TS-*Si*-A
Opt @ B97D/6-31G** in 1,2-dichloroethane (SMD model, SAS)
SCF Done: E(RB97D) = -4638.63617334 a.u.
Zero-point correction = 1.438455 Hartree/Particle
Thermal correction to Gibbs Free Energy (at 323.15 K) = 1.290889 a.u.
Imaginary Frequency = -50.958 cm^{-1}
SP @ ωB97XD/def2-TZVPP in 1,2-dichloroethane (SMD model, vdW surface)
SCF Done: E(RwB97XD) = -4641.57772870 a.u.

N,0,-1.3531227311,0.0674470761,-2.6534862438
N,0,-2.6098219752,-0.3838235874,-2.6363221452
C,0,-0.8188731342,0.7433215668,-3.7712325324
O,0,0.3222715691,1.1703153984,-3.7636561727
O,0,-1.6979981566,0.8062956419,-4.7892168122
C,0,-2.8111683247,-1.5911378467,-1.9007017175
O,0,-3.9095917658,-2.1209820092,-1.8187650257
O,0,-1.6631388582,-2.0933783298,-1.3931807091
C,0,-1.2033065655,1.5035597766,-5.9710670332
C,0,-2.3188542275,1.4689799411,-7.0021157589
H,0,-0.2943165825,0.994965096,-6.3250638677
H,0,-0.9339193221,2.5291765951,-5.684081753
H,0,-1.9872112459,1.9820585276,-7.9171008911
H,0,-2.5831178476,0.4331428131,-7.2580585045
H,0,-3.2149285117,1.9762897807,-6.6198592132
C,0,-1.7221230148,-3.4324243443,-0.809451494
C,0,-0.4767927579,-3.6092389074,0.0434155399
H,0,-1.7644149768,-4.1521354145,-1.6412474372
H,0,-2.6475519191,-3.5223527799,-0.2270044935
H,0,-0.4695009389,-4.6241755677,0.4685346471
H,0,0.4287126941,-3.474146362,-0.5586885701
H,0,-0.4560330988,-2.8811040714,0.8621006539
H,0,-0.6661765108,-0.1038756148,-1.8855175876
P,0,1.1102517619,0.2111922693,0.537598512
O,0,1.095818225,1.7437111598,0.8645945379
O,0,0.6982285662,-0.2566962874,-0.8158612174
C,0,1.6932203399,-1.4285140829,3.3345456323
C,0,2.1173029636,-1.4413971244,4.7122335204
C,0,1.332118001,-0.7321783386,5.694130104
C,0,0.1885338525,0.0031535729,5.2778635283
C,0,-0.1712853482,0.102889226,3.9430933519
C,0,0.6040866982,-0.6222818095,2.9911914432
C,0,2.409177794,-2.2097682952,2.2841095351
C,0,2.6118944135,-3.6330896563,2.3781731846
C,0,3.3814613777,-4.2945641928,1.350468878
C,0,3.8743533351,-3.5460959581,0.2453771371
C,0,3.5980779465,-2.1947283291,0.088401765
C,0,2.86563394,-1.5620575802,1.1373931086
H,0,-0.4148858397,0.5104425838,6.0308263349
H,0,4.4440785282,-4.0629034258,-0.5274537394
O,0,2.6196551141,-0.1893204113,1.0132652594
O,0,0.175851863,-0.5701114504,1.6623658378
C,0,-2.4546122103,4.3951623395,-4.0740947988
C,0,-1.7156910896,3.9668378093,-2.9714309918
C,0,-2.189669023,2.9109295749,-2.1655492346
C,0,-3.4054717766,2.2447897891,-2.4992590601
C,0,-4.1318332758,2.6923291744,-3.6255914964

C,0,-3.673411312,3.7631004953,-4.3929765698
H,0,-2.0918966395,5.2209378097,-4.6870466338
H,0,-0.7684925778,4.4322513543,-2.705230731
C,0,-1.4164836674,2.4981273256,-0.9729872134
C,0,-3.8269291559,1.0862978257,-1.7046534519
H,0,-5.0569465469,2.1921455661,-3.9044089665
H,0,-4.257086883,4.1041676944,-5.2484791909
C,0,-3.19019634,0.9056582809,-0.4218142681
C,0,-2.015700405,1.5236382016,-0.0866157815
H,0,-3.627922388,0.1833564854,0.2636539825
H,0,-1.5409898968,1.3193913759,0.8690260028
O,0,-0.2911721734,3.0366543918,-0.7343983157
H,0,0.503624134,2.3254028078,0.186976566
C,0,-5.2390061333,0.5622058659,-1.8376890908
H,0,-5.4910821102,0.3794851988,-2.8911975073
H,0,-5.3178130329,-0.3995626298,-1.3216919781
C,0,-6.2346379246,1.5539095395,-1.1996919052
H,0,-6.2448443916,2.5161013455,-1.7361643286
H,0,-5.9252496602,1.760645759,-0.1658269878
N,0,-7.5643649367,0.9206427881,-1.1412098091
H,0,-7.9815291013,0.7448213024,-2.056935175
S,0,-8.7461120442,1.7215862084,-0.1979259913
O,0,-8.6220728037,3.196191607,-0.2797110176
O,0,-10.0203077407,1.0625492641,-0.5523120421
C,0,-8.1908756366,1.2405591247,1.4536926094
C,0,-7.6673475582,-0.0403903736,1.6888643752
C,0,-8.3350024658,2.1674858994,2.4962085087
C,0,-7.2518397128,-0.3744268133,2.9836336925
H,0,-7.5692093167,-0.7447755629,0.8646559385
C,0,-7.9271737031,1.8085421179,3.786060088
H,0,-8.7337039935,3.1588330875,2.2876061444
C,0,-7.3640817954,0.5446567463,4.0472870913
H,0,-6.8329895588,-1.3636119105,3.173843358
H,0,-8.0252329273,2.5286677465,4.5988945036
C,0,-6.8372894421,0.2061393156,5.4222695112
H,0,-5.7777632877,0.4987097845,5.5057033675
H,0,-7.3924318963,0.7402655312,6.2067445885
H,0,-6.8969965783,-0.872559165,5.6247489251
C,0,3.3016668464,-2.10496688,5.1507976653
C,0,3.6666684224,-2.1043200711,6.4873967379
C,0,2.8695902876,-1.4400586703,7.4570576667
C,0,1.7281937899,-0.7637850738,7.063049079
H,0,3.9247425152,-2.6120775343,4.4178878249
H,0,4.5795484488,-2.6151461826,6.7961429211
H,0,3.1661083318,-1.4528456604,8.5064933311
H,0,1.1168697936,-0.229592773,7.7926528328
C,0,3.5969838885,-5.7010696838,1.4394563456
C,0,3.054242518,-6.4403781121,2.4763495098
C,0,2.2632902708,-5.7973373078,3.4651097126
C,0,2.0479726919,-4.4290682284,3.4179941621
H,0,4.1875232638,-6.1854258353,0.6596903222
H,0,3.2208048733,-7.5169454413,2.5288605175
H,0,1.8163610083,-6.3860071876,4.2672343381
H,0,1.4363551798,-3.9473800919,4.1775751198
C,0,-1.3713244167,0.8945574914,3.5186940805
C,0,-1.2949264279,2.3089682746,3.3982813118
C,0,-2.5876671641,0.221852688,3.2471439106
C,0,-2.4498075224,3.0151243115,3.0241924355
C,0,-3.7167808979,0.9758224096,2.8810813471
C,0,-3.6678052161,2.370108743,2.7585696228
H,0,-2.3947422357,4.0978908835,2.916738613

H,0,-4.6577313748,0.4629861873,2.6738870453
C,0,3.9666895479,-1.4292324642,-1.1419745426
C,0,4.8276687664,-0.3007704416,-1.0551056641
C,0,3.3965288193,-1.7861198973,-2.3952990957
C,0,5.0737320058,0.4603311041,-2.2069330793
C,0,3.683451302,-0.9913378703,-3.5170630314
C,0,4.4981750228,0.1438376994,-3.4461006237
H,0,5.7283580276,1.3286633417,-2.1293895984
H,0,3.2334974208,-1.2505860166,-4.4767047241
C,0,-2.7168004475,-1.2974443368,3.3287193118
H,0,-1.7437208293,-1.7191445461,3.6125540744
C,0,0.0045901746,3.0646441062,3.6750956894
H,0,0.8377291574,2.3710245354,3.4988161319
C,0,-4.8972686002,3.1580798787,2.3304781293
H,0,-5.7001051609,2.433625036,2.1277540855
C,0,2.4841586127,-2.9963998061,-2.598238718
H,0,2.2392027075,-3.4193960571,-1.6169393924
C,0,5.5534508895,0.0896205363,0.2330701089
H,0,5.1868506238,-0.5382346065,1.0551556812
C,0,4.6858103344,1.038956154,-4.6632684597
H,0,4.3239078818,0.480407818,-5.5429487543
C,0,3.2173541546,-4.0931329267,-3.4009471967
H,0,4.1506658182,-4.4002904036,-2.9042881421
H,0,2.5771344226,-4.982300983,-3.5170082493
H,0,3.4799593136,-3.7277287033,-4.4059297535
C,0,1.1491120177,-2.6244013657,-3.2769000971
H,0,0.6189846234,-1.8695924193,-2.6892061321
H,0,1.3084315073,-2.2302888687,-4.2919662885
H,0,0.5121766084,-3.5191249534,-3.365044147
C,0,6.1609521567,1.4119656738,-4.9122502294
H,0,6.2558208571,2.0047594956,-5.8353603375
H,0,6.5600292831,2.0202708932,-4.0860083729
H,0,6.7912311756,0.5149304764,-5.0081231354
C,0,3.8153637237,2.308695255,-4.518426858
H,0,2.7576753314,2.0483069818,-4.3739119108
H,0,4.1426126587,2.8946596214,-3.6450685043
H,0,3.908666563,2.9464737254,-5.4128260358
C,0,5.3024163975,1.5607607817,0.6251814054
H,0,5.7341237878,2.2483919489,-0.1186305771
H,0,4.22817163,1.769135799,0.7080810635
H,0,5.7781069529,1.7790597621,1.5943626523
C,0,7.06731429,-0.1870260676,0.0929500475
H,0,7.2567254546,-1.2460631803,-0.1393834606
H,0,7.5002948996,0.4194911698,-0.7176141243
H,0,7.5945306435,0.0639930738,1.0272704713
C,0,-3.0966105941,-1.8962370869,1.9586254725
H,0,-4.1020706242,-1.5717109145,1.6478227949
H,0,-2.3769153243,-1.5916149201,1.1892898185
H,0,-3.1067154669,-2.9956258137,2.0132401403
C,0,-3.7364571062,-1.7123351855,4.4090029871
H,0,-3.8051758404,-2.809497387,4.4762363148
H,0,-3.4488846017,-1.3221261318,5.3968150828
H,0,-4.7351524961,-1.3219640202,4.1665998382
C,0,0.2129235183,4.2797460625,2.7476526553
H,0,0.053324694,4.0207137837,1.6939138097
H,0,-0.4685659348,5.1053772372,3.0073596195
H,0,1.2404323976,4.6559973684,2.8604237616
C,0,0.0608438212,3.5234160758,5.1497897446
H,0,0.0115937354,2.6722386762,5.8422265369
H,0,0.9963562088,4.0708569357,5.3457913193
H,0,-0.7830825402,4.1952886227,5.3730974911

C,0,-5.3812200958,4.0884665597,3.461872358
H,0,-5.5937394302,3.5206471119,4.3791838479
H,0,-4.6113316444,4.8393125351,3.6985818475
H,0,-6.2956425642,4.6224882588,3.1612181209
C,0,-4.647397052,3.9699677158,1.0413301086
H,0,-4.2657898806,3.3315472375,0.2341786931
H,0,-5.5815827134,4.4430055202,0.7023870535
H,0,-3.9046056789,4.763502292,1.2165398063

TS-*Si*-B
Opt @ B97D/6-31G** in 1,2-dichloroethane (SMD model, SAS)
SCF Done: E(RB97D) = -4638.62916434 a.u.
Zero-point correction = 1.438828 Hartree/Particle
Thermal correction to Gibbs Free Energy (at 323.15 K) = 1.288662 a.u.
Imaginary Frequency = -87.5931 cm^{-1}
SP @ ωB97XD/def2-TZVPP in 1,2-dichloroethane (SMD model, vdW surface)
SCF Done: E(RwB97XD) = -4641.57442631 a.u.

N,0,-1.4162059863,0.1050258413,-2.3948815323
N,0,-2.6427059697,-0.3569260982,-2.1226580844
C,0,-1.0951609625,0.691435705,-3.6313292615
O,0,0.0169001766,1.1395932222,-3.8546670917
O,0,-2.1364997685,0.6600235977,-4.487454067
C,0,-2.5778223016,-1.5357186207,-1.3249874484
O,0,-1.5686417891,-2.1846311895,-1.0966257866
O,0,-3.8279993045,-1.8952385603,-0.9458670669
C,0,-1.8741325082,1.2814393675,-5.7797024325
C,0,-3.1493660676,1.1544586437,-6.5965266657
H,0,-1.02495485,0.7662840869,-6.2525165919
H,0,-1.5909075723,2.3296502852,-5.6128742583
H,0,-2.9956791233,1.6059986973,-7.5879725721
H,0,-3.4238091473,0.0986416097,-6.7319654595
H,0,-3.9810767073,1.6728919384,-6.100168029
C,0,-3.9216661851,-3.1492969622,-0.2088456478
C,0,-5.2946169805,-3.1815647735,0.444068453
H,0,-3.11333891,-3.195738964,0.5315588893
H,0,-3.7821360741,-3.9754829314,-0.9224947331
H,0,-5.4210570479,-4.1356618901,0.9774877735
H,0,-5.3994810849,-2.3630904983,1.1689214232
H,0,-6.0929744054,-3.0950041228,-0.3070896434
H,0,-0.6110655151,-0.0522338811,-1.7517272715
P,0,1.5021879084,0.4097760263,0.4676117281
O,0,1.5527790965,1.9644669193,0.7060557262
O,0,0.8840736875,-0.1130059203,-0.7822347639
C,0,2.4818937218,-0.9428477646,3.2999277914
C,0,3.096467946,-0.7973896013,4.5958309808
C,0,2.43816566,0.0070695013,5.5967545189
C,0,1.2271000361,0.6756488519,5.2697271273
C,0,0.6834453256,0.6256570736,3.9955616717
C,0,1.3386559875,-0.1881333839,3.0246844681
C,0,3.0550516308,-1.8371856098,2.2546969355
C,0,3.3054947361,-3.2367230736,2.489381573
C,0,3.9535495329,-4.0100036418,1.4568481777
C,0,4.2822738262,-3.393568858,0.2175794135
C,0,3.952539027,-2.0754202372,-0.0570479217
C,0,3.3391314057,-1.3268983107,0.9887957761
H,0,0.7185636964,1.2530224261,6.0418397292
H,0,4.7931981176,-3.9821400029,-0.5439194416
O,0,3.0684331525,0.0234982568,0.734173332
O,0,0.740357731,-0.2800879746,1.7655228391

C,0,-2.6739333827,4.3511660926,-3.8652813885
C,0,-1.7920194636,3.9809758864,-2.849887537
C,0,-2.1465941548,2.9685470566,-1.9352857292
C,0,-3.3895037749,2.2851066233,-2.0674444832
C,0,-4.2610727845,2.6711470385,-3.1103011077
C,0,-3.9178171786,3.7008419723,-3.9870740946
H,0,-2.4020260666,5.1438729001,-4.5630363274
H,0,-0.8209495123,4.4591500606,-2.7363845073
C,0,-1.2160909764,2.6209066068,-0.8350269249
C,0,-3.6985751116,1.1717574744,-1.1568096906
H,0,-5.209085473,2.1536754185,-3.2416714304
H,0,-4.6115375313,3.9929794182,-4.7759188405
C,0,-2.8933130723,1.0676695833,0.0414218501
C,0,-1.6894475382,1.7003752529,0.1789732573
H,0,-3.2317002179,0.3870695408,0.8196490881
H,0,-1.0885662465,1.5493610925,1.0711480034
O,0,-0.0748836241,3.1670192991,-0.7799199578
H,0,0.8802923558,2.4944681306,0.0938095405
C,0,-5.1384854908,0.7173014814,-1.0383507997
H,0,-5.5650724738,0.4794350711,-2.0219358245
H,0,-5.1891403822,-0.1919499401,-0.4357261216
C,0,-5.9639706828,1.8072379285,-0.3199162027
H,0,-5.9726658475,2.7492394451,-0.8887077899
H,0,-5.4873724676,2.0259025795,0.6433157113
N,0,-7.3274677701,1.3106508128,-0.053979827
H,0,-7.9791032889,1.4331147818,-0.8296485533
S,0,-8.1080667697,1.891838094,1.3539198062
O,0,-7.6469616422,3.2502029442,1.7245501157
O,0,-9.5412152395,1.6186157525,1.1216194381
C,0,-7.4428519032,0.7470892712,2.5837279583
C,0,-7.4683791188,-0.6337510409,2.3265342669
C,0,-6.9855171462,1.2476884802,3.8093777171
C,0,-7.027006301,-1.5159572363,3.3169164823
H,0,-7.8143141897,-0.9996733154,1.3613990106
C,0,-6.5420998203,0.3465463453,4.7890195583
H,0,-6.9673249035,2.3217191425,3.9834193195
C,0,-6.5666054536,-1.0427393066,4.5650248354
H,0,-7.0422589677,-2.5897864347,3.1245489652
H,0,-6.1745905762,0.7285604096,5.7423270554
C,0,-6.1367679572,-2.0185557149,5.6367631456
H,0,-5.6018317294,-1.5141102541,6.4522569507
H,0,-7.014533803,-2.5254803794,6.0703976909
H,0,-5.4800401844,-2.7986721505,5.2253151512
C,0,4.3496122243,-1.3921973285,4.9295108051
C,0,4.8994920919,-1.237101057,6.1917821403
C,0,4.2280677146,-0.4794814104,7.1878935548
C,0,3.0234897821,0.1335591191,6.890320626
H,0,4.8774265266,-1.9694522982,4.1740990855
H,0,5.861565811,-1.6976347615,6.4199185584
H,0,4.6698767622,-0.3710688953,8.1791592648
H,0,2.5058738679,0.7381467358,7.6373622103
C,0,4.2312706713,-5.3887632472,1.6892430464
C,0,3.861983535,-5.9992421927,2.875406348
C,0,3.1872491286,-5.2500420063,3.8754709764
C,0,2.9154512148,-3.9042634919,3.6877149012
H,0,4.7297135341,-5.9569831267,0.9017431983
H,0,4.0749471499,-7.0564483954,3.0386943288
H,0,2.8745332547,-5.7391598354,4.7989681193
H,0,2.3928567092,-3.3422292275,4.4584146784
C,0,-0.5836838335,1.3550829741,3.6706358626
C,0,-0.5693036066,2.759557506,3.4527863374

C,0,-1.8029021338,0.6365015023,3.6035989479
C,0,-1.7849593173,3.412152473,3.1894966272
C,0,-2.9915046184,1.3373346302,3.3366734435
C,0,-3.0039183889,2.7210073239,3.1195243134
H,0,-1.7779518734,4.4863710988,3.0087102944
H,0,-3.9343957381,0.7890055183,3.2888714856
C,0,4.2300338457,-1.4408750569,-1.3838907924
C,0,5.2723890495,-0.4891803969,-1.5055927975
C,0,3.4219745553,-1.7658291543,-2.5062469587
C,0,5.5026179133,0.1149060958,-2.7531720194
C,0,3.7052183007,-1.1455480447,-3.7323792381
C,0,4.7277111668,-0.1966187969,-3.8779918869
H,0,6.2995406719,0.8541967512,-2.8349966486
H,0,3.0818308768,-1.3719856318,-4.5981613837
C,0,-1.8785811682,-0.8717586851,3.8319456662
H,0,-0.8657622879,-1.2557744905,4.0079143452
C,0,0.7288890112,3.5655410149,3.5028300956
H,0,1.5586111682,2.8760615027,3.2971879478
C,0,-4.3063227609,3.4414957198,2.8050613544
H,0,-5.0703786457,2.673344832,2.6159753744
C,0,2.2406080674,-2.7324911652,-2.40836678
H,0,1.8972358699,-2.737445086,-1.3651427328
C,0,6.1594570408,-0.086022905,-0.3280838816
H,0,5.8312674005,-0.6329116996,0.5660840799
C,0,4.8769459164,0.5374887298,-5.2052373107
H,0,4.535097962,-0.1485138672,-5.9991318438
C,0,2.6569072568,-4.1699838611,-2.7910353145
H,0,3.4426499866,-4.562554208,-2.1309496443
H,0,1.7901753491,-4.8477405123,-2.7315724696
H,0,3.0387468068,-4.1930176141,-3.8244209953
C,0,1.0437329451,-2.2913922227,-3.2780684102
H,0,0.8659780753,-1.217163494,-3.1671133766
H,0,1.2249685842,-2.5041668921,-4.3445786407
H,0,0.1406282562,-2.8350732934,-2.9709618624
C,0,6.3224129732,0.9559843338,-5.5308657297
H,0,6.3743861685,1.3910212719,-6.5407249625
H,0,6.6831405454,1.720679228,-4.8255758925
H,0,7.01098977,0.0987320851,-5.4851428702
C,0,3.9394761455,1.7687859255,-5.2150657487
H,0,2.8987545048,1.4791506409,-5.0099859856
H,0,4.2477717214,2.4823315513,-4.4346928813
H,0,3.9803352207,2.2836193146,-6.1890949918
C,0,6.0341537334,1.4215442282,-0.020459203
H,0,6.3954576884,2.0258545452,-0.8672237389
H,0,4.9889533247,1.6934003867,0.1776655984
H,0,6.6394501103,1.6827081294,0.8623696166
C,0,7.6312578697,-0.4742809796,-0.5865950479
H,0,7.7297392391,-1.5557874575,-0.7657519783
H,0,8.0264328235,0.0516455487,-1.4694856815
H,0,8.2593681305,-0.2062878628,0.2779458822
C,0,-2.4393677131,-1.6065172262,2.5960365234
H,0,-3.4681673284,-1.2782502684,2.3796060214
H,0,-1.8109587268,-1.4298395729,1.7131975431
H,0,-2.4699548817,-2.691144527,2.7856820951
C,0,-2.7224009903,-1.183909521,5.0855098258
H,0,-2.7659332733,-2.2698978698,5.2632143555
H,0,-2.2942029312,-0.7038043941,5.9783400546
H,0,-3.749053283,-0.8127774489,4.9589651569
C,0,0.7890640792,4.6864515907,2.4440104795
H,0,0.5247669272,4.3193178459,1.4449800093
H,0,0.1098332822,5.5160020134,2.6969793421

H,0,1.8081017897,5.0991837248,2.4037862789
C,0,0.937919167,4.1722385939,4.9086008631
H,0,0.9949840068,3.3955497511,5.6831682288
H,0,1.8713239153,4.7563402859,4.9397384704
H,0,0.1035229046,4.8456099895,5.1614201764
C,0,-4.7924816068,4.2716481893,4.0114692847
H,0,-4.899465435,3.6424602986,4.9084155392
H,0,-4.0711333661,5.0696034727,4.2477381219
H,0,-5.7637855921,4.7388607754,3.7884323114
C,0,-4.2043746377,4.3256118955,1.5458260438
H,0,-3.8193756675,3.7550755228,0.689430868
H,0,-5.1953546327,4.7258408876,1.2886483221
H,0,-3.5237024055,5.1742607527,1.7139216932

TS-*Si*-C (TS-major)
Opt @ B97D/6-31G** in 1,2-dichloroethane (SMD model, SAS)
SCF Done: E(RB97D) = -4638.64141809 a.u.
Zero-point correction = 1.438311 Hartree/Particle
Thermal correction to Gibbs Free Energy (at 323.15 K) = 1.290377 a.u.
Imaginary Frequency = -84.4517 cm^{-1}
SP @ ωB97XD/def2-TZVPP in 1,2-dichloroethane (SMD model, vdW surface)
SCF Done: E(RwB97XD) = -4641.58349437 a.u.

N,0,-1.6114419875,0.0491391286,-2.6252614725
N,0,-2.8484615999,-0.4389455159,-2.5387530514
C,0,-1.2324534401,0.7499538288,-3.7935642418
O,0,-1.8809075252,0.834442,-4.816633572
O,0,-0.0131084841,1.2804543831,-3.5490083951
C,0,-2.9673286837,-1.6484022572,-1.7926435507
O,0,-4.0477489763,-2.1710195506,-1.5613522349
O,0,-1.7633482455,-2.170297006,-1.4550429583
C,0,0.6262295098,1.9573905767,-4.6650622318
C,0,1.9245885297,2.537983421,-4.1269376779
H,0,-0.0581018561,2.7283084235,-5.0472913091
H,0,0.7998533646,1.2226456931,-5.4661932587
H,0,2.4516700092,3.0675032857,-4.9344168832
H,0,1.7179494481,3.2466315192,-3.312292246
H,0,2.5728841526,1.7403520752,-3.740432931
C,0,-1.776289254,-3.4732411356,-0.7904206488
C,0,-0.4507602713,-3.6279467938,-0.0621966188
H,0,-1.9193233749,-4.2401712082,-1.5671959557
H,0,-2.6328504335,-3.5116436943,-0.1067834901
H,0,-0.4477690619,-4.5825036837,0.4851931523
H,0,0.3888351922,-3.6321243254,-0.7677449358
H,0,-0.3000100031,-2.8093407476,0.6514697991
H,0,-0.8898831448,-0.0973570325,-1.8873129538
P,0,0.9973998982,0.336203284,0.4528690492
O,0,0.9483081352,1.8727677447,0.7488677804
O,0,0.5750376704,-0.1758621217,-0.8824840733
C,0,1.6600066837,-1.2932773138,3.23456922
C,0,2.1188566621,-1.3160676638,4.6001055482
C,0,1.3557176216,-0.6162641589,5.6057896886
C,0,0.1942389435,0.1097680047,5.2236950345
C,0,-0.2052157271,0.2094737004,3.9000586763
C,0,0.5555615017,-0.4975707579,2.923647426
C,0,2.3574969451,-2.0546656542,2.1588442193
C,0,2.5571392114,-3.4791519439,2.2249991611
C,0,3.310340885,-4.1251336631,1.1762286708
C,0,3.816673026,-3.3521224199,0.0946692116
C,0,3.5563070481,-1.994697171,-0.0250139858

C,0,2.8038167376,-1.3849441226,1.020622371
H,0,-0.3959089368,0.6043738927,5.9954425368
H,0,4.4048775238,-3.8486099326,-0.6767376852
O,0,2.5315028773,-0.0154894273,0.9033862648
O,0,0.0980489751,-0.4476591995,1.6045883199
C,0,-2.8799798064,4.3232675731,-4.0712769417
C,0,-2.1059031097,3.9576496717,-2.9703351428
C,0,-2.508033191,2.8899287191,-2.1416969898
C,0,-3.6970703428,2.1620668677,-2.4394223116
C,0,-4.4596917514,2.5479218099,-3.563662961
C,0,-4.0646027999,3.6196807282,-4.3628192999
H,0,-2.5686274776,5.1536320648,-4.7058994283
H,0,-1.1849037476,4.483069331,-2.7239578736
C,0,-1.6798925445,2.5156420956,-0.9755238762
C,0,-4.0528921865,0.9985984063,-1.6148980477
H,0,-5.3594817239,1.9933730033,-3.8206978184
H,0,-4.6685700344,3.9020325414,-5.2252694635
C,0,-3.3724846136,0.8659045092,-0.3464259521
C,0,-2.209794072,1.5292942812,-0.0597776485
H,0,-3.7607036858,0.1374706501,0.3614152914
H,0,-1.6932886573,1.3519099593,0.8793671037
O,0,-0.5607779144,3.0906066957,-0.7876499487
H,0,0.291375481,2.4225366497,0.0969231477
C,0,-5.4607337628,0.4471615268,-1.6874524169
H,0,-5.7398673964,0.2271844505,-2.7269634147
H,0,-5.5069304961,-0.4981005035,-1.1394345774
C,0,-6.453242865,1.4432900287,-1.0525325089
H,0,-6.485349266,2.3929548401,-1.6096152038
H,0,-6.1250002572,1.675957427,-0.0301787254
N,0,-7.7749409822,0.798003916,-0.9530092729
H,0,-8.2172180169,0.613249273,-1.8549355823
S,0,-8.9358135881,1.5868253189,0.0240218138
O,0,-8.8285891482,3.0628009582,-0.0583177153
O,0,-10.213893532,0.9165590619,-0.2938093365
C,0,-8.3285245449,1.1100189158,1.6587128918
C,0,-7.7724471142,-0.1603552141,1.8749417802
C,0,-8.4636591284,2.0302335391,2.7085503527
C,0,-7.3150863053,-0.4899368878,3.1568073596
H,0,-7.6821064706,-0.8599073533,1.0457875071
C,0,-8.0137975856,1.6757341896,3.9854234797
H,0,-8.8879168098,3.0139302749,2.5147068571
C,0,-7.417567281,0.4229273851,4.2265855229
H,0,-6.8710201759,-1.4709569983,3.3315595033
H,0,-8.1050186122,2.3909865786,4.8034043081
C,0,-6.8475896079,0.0915764943,5.5860704722
H,0,-5.7995443982,0.427050364,5.6505592024
H,0,-7.4070901723,0.5941027704,6.3882332161
H,0,-6.8584871278,-0.9906748672,5.7772749526
C,0,3.3144931782,-1.9812786433,5.0037877403
C,0,3.7133934916,-1.9875964328,6.3306651228
C,0,2.9406692037,-1.3292748807,7.3238883846
C,0,1.787659571,-0.6537750408,6.963597881
H,0,3.9187658787,-2.4839465154,4.2521628879
H,0,4.6340417754,-2.4994377061,6.6135181745
H,0,3.2643310898,-1.3470716392,8.3651718991
H,0,1.1936964585,-0.1262668313,7.7121185858
C,0,3.511568579,-5.5353348342,1.2311694986
C,0,2.9720631743,-6.2912338414,2.2577049059
C,0,2.2019700275,-5.6614005757,3.2714192785
C,0,2.000740427,-4.2903512579,3.257190596
H,0,4.0894270508,-6.0085196604,0.4352877932

H,0,3.1268449461,-7.3704452843,2.2845191736
H,0,1.7604466213,-6.2628485811,4.0669891349
H,0,1.4046775319,-3.8180996561,4.0349697262
C,0,-1.4406846309,0.9649032486,3.5149018778
C,0,-1.4089605498,2.3783282034,3.3715067463
C,0,-2.6461910897,0.2526669084,3.3014908769
C,0,-2.5964058849,3.0453991076,3.0294268986
C,0,-3.8079840407,0.9678986534,2.9610282475
C,0,-3.8029802313,2.3609290662,2.8146192502
H,0,-2.5774730475,4.1274872187,2.9045392258
H,0,-4.7400471474,0.4254120595,2.7925111184
C,0,4.0354823565,-1.1954973943,-1.1963469981
C,0,5.0176941459,-0.1869827157,-1.0123766884
C,0,3.5024245421,-1.4389217666,-2.4908189999
C,0,5.4571601332,0.5471627877,-2.125332378
C,0,3.9798473048,-0.6776753128,-3.5706660303
C,0,4.9497569743,0.3228776445,-3.4128015684
H,0,6.2136814589,1.3173016351,-1.974924788
H,0,3.5694380667,-0.8566054657,-4.5660985059
C,0,-2.7237228969,-1.2687866319,3.4102014405
H,0,-1.7400137772,-1.6499882267,3.7138381166
C,0,-0.1213992355,3.1712273171,3.5931915644
H,0,0.7230011698,2.4998660967,3.3859512646
C,0,-5.0681820123,3.1063499954,2.4157262786
H,0,-5.8530944432,2.3553378165,2.2415964292
C,0,2.4227209034,-2.4879973096,-2.7512784547
H,0,1.9935731178,-2.7706071917,-1.7830927142
C,0,5.6478943464,0.1217511809,0.3457901006
H,0,5.1821267735,-0.5142866886,1.1092182343
C,0,5.4545543519,1.0996358803,-4.6228646559
H,0,4.6874071006,1.0112861325,-5.4105588716
C,0,3.025443531,-3.7528108991,-3.3995240257
H,0,3.8119033202,-4.1995019557,-2.7736109068
H,0,2.2439355054,-4.511704528,-3.5647728064
H,0,3.4734946949,-3.5071359936,-4.3752202869
C,0,1.2674508154,-1.942792771,-3.6164735551
H,0,0.9139886645,-0.9896774768,-3.2149320212
H,0,1.583753728,-1.793455255,-4.6611500752
H,0,0.4310770483,-2.6576003356,-3.623224901
C,0,6.7569860227,0.4629297711,-5.1586903061
H,0,7.1110931189,0.99128539,-6.0582654006
H,0,7.5484088417,0.5162379763,-4.3946882988
H,0,6.603792187,-0.5962625644,-5.4136900303
C,0,5.6681354475,2.5990400315,-4.3356894749
H,0,4.7693079196,3.0565671612,-3.8987268622
H,0,6.5024700309,2.7534051752,-3.6344095831
H,0,5.9172729489,3.132960824,-5.2655337214
C,0,5.4126682376,1.5903327216,0.7586416873
H,0,5.9115750264,2.2801410592,0.060111316
H,0,4.340637725,1.8262119829,0.7735836565
H,0,5.8262193868,1.7727082233,1.7630521864
C,0,7.1558810229,-0.2122249325,0.3369115077
H,0,7.3262855847,-1.2688533534,0.0805794411
H,0,7.6910415834,0.4054608071,-0.4007657054
H,0,7.5983691764,-0.0187796438,1.3268297889
C,0,-3.0618448475,-1.9043854814,2.0455157527
H,0,-4.0753957862,-1.6260813777,1.7174407275
H,0,-2.3447676865,-1.58472322,1.2792904209
H,0,-3.0290587914,-3.0023388896,2.1198602914
C,0,-3.741283263,-1.7048404182,4.4838786113
H,0,-3.7688399269,-2.8026895295,4.5666189024

H,0,-3.4810445947,-1.2899491892,5.4691551702
H,0,-4.751349364,-1.3576210836,4.2243771924
C,0,0.0167737645,4.3874682065,2.6547242645
H,0,-0.1731807783,4.1184270464,1.6084268456
H,0,-0.6793043796,5.1936182692,2.9359272277
H,0,1.0359752381,4.7946856601,2.7285824797
C,0,-0.0190384398,3.6352289688,5.063743921
H,0,-0.0176531154,2.7841058082,5.7581012444
H,0,0.9083253563,4.2081697342,5.2218662045
H,0,-0.8714656844,4.2843072167,5.3196693349
C,0,-5.5489870515,4.0299410508,3.55404616
H,0,-5.7173427034,3.4628913159,4.4809283069
H,0,-4.7966949246,4.8064132678,3.76320579
H,0,-6.4880325817,4.5324014065,3.2758327598
C,0,-4.8812371262,3.9155216244,1.1143141703
H,0,-4.4983566128,3.2842993791,0.3021125745
H,0,-5.840419877,4.3517674504,0.7968704246
H,0,-4.1630948791,4.7366159943,1.2637177275

TS-*Si*-D

Opt @ B97D/6-31G** in 1,2-dichloroethane (SMD model, SAS)
SCF Done: E(RB97D) = -4638.63485955 a.u.
Zero-point correction = 1.438899 Hartree/Particle
Thermal correction to Gibbs Free Energy (at 323.15 K) = 1.290319 a.u.
Imaginary Frequency = -104.8439 cm^{-1}
SP @ ωB97XD/def2-TZVPP in 1,2-dichloroethane (SMD model, vdW surface)
SCF Done: E(RwB97XD) = -4641.58143907 a.u.

N,0,-1.6136862559,0.1977231739,-2.6808417438
N,0,-2.8503342582,-0.2998643013,-2.5943562454
C,0,-1.226896877,0.9042536342,-3.835986295
O,0,-1.8746304949,1.0177585482,-4.8576202991
O,0,-0.0003252151,1.4176126616,-3.5824610326
C,0,-2.8415442923,-1.5655896729,-1.9432287168
O,0,-1.8497386343,-2.2564232897,-1.7660454546
O,0,-4.1071114796,-1.9465159366,-1.647016123
C,0,0.652804736,2.0935540445,-4.6901715899
C,0,1.9656609143,2.6352017733,-4.1459225297
H,0,-0.0129200758,2.8862805957,-5.0610012335
H,0,0.8083642164,1.3662695512,-5.5018400889
H,0,2.5066552221,3.1609829162,-4.9466009931
H,0,1.776984022,3.339167759,-3.322725294
H,0,2.5930851968,1.8167753775,-3.7682950311
C,0,-4.2448845242,-3.2817841627,-1.0806589453
C,0,-5.650030591,-3.3798379642,-0.5069564583
H,0,-3.4746025803,-3.4300607785,-0.3134168329
H,0,-4.0758018603,-4.0143497026,-1.8842994475
H,0,-5.8125774169,-4.3920968907,-0.1085263757
H,0,-5.7851196123,-2.6615306514,0.3144842915
H,0,-6.4085902229,-3.1837425958,-1.2781212077
H,0,-0.8887055588,-0.0113454797,-1.9627190547
P,0,0.971958559,0.3141011827,0.4716657645
O,0,0.9347823261,1.8431390009,0.8315866335
O,0,0.5228749147,-0.1306091126,-0.8776234135
C,0,1.6757683717,-1.291811665,3.2544070654
C,0,2.1371155332,-1.27391629,4.6199799662
C,0,1.3483330762,-0.5899945421,5.6164181811
C,0,0.1638194569,0.0909590332,5.2236850726
C,0,-0.2326687376,0.1654266454,3.8975042474
C,0,0.5487618793,-0.5325983268,2.9305895508

C,0,2.3895359807,-2.0583134997,2.1939472755
C,0,2.6520195876,-3.4705669902,2.3070237681
C,0,3.4360583702,-4.1170337956,1.2816657479
C,0,3.8935066392,-3.3629327399,0.1655606519
C,0,3.5634497349,-2.0269595226,-0.0021624078
C,0,2.8031413587,-1.4086901064,1.0316738461
H,0,-0.4423474726,0.5777341188,5.9877763654
H,0,4.5092484393,-3.8558939824,-0.5863031038
O,0,2.5139379654,-0.0459065466,0.8843880467
O,0,0.0950035578,-0.5052371516,1.6099775711
C,0,-2.7940579068,4.5072974004,-3.987153202
C,0,-2.0456400587,4.0994092363,-2.8828054258
C,0,-2.4702061195,3.0054491417,-2.1020602438
C,0,-3.6536600654,2.2939700416,-2.4503809265
C,0,-4.3878005289,2.7193618913,-3.5786187623
C,0,-3.9724339925,3.817920699,-4.330576907
H,0,-2.4659503371,5.3583001919,-4.5849455358
H,0,-1.1269472737,4.6096333321,-2.5984782984
C,0,-1.6648083598,2.583631888,-0.9333560409
C,0,-4.0329722995,1.1007917221,-1.6726527493
H,0,-5.2801872028,2.1747538907,-3.8793112379
H,0,-4.5549407886,4.1305694586,-5.1972936728
C,0,-3.383603029,0.9291226676,-0.3882450065
C,0,-2.2234595606,1.5727596556,-0.0585553544
H,0,-3.7942410984,0.1846914421,0.2895368234
H,0,-1.7254882817,1.3622143959,0.8838768315
O,0,-0.547104735,3.1385721918,-0.7062791938
H,0,0.3081216397,2.4121326311,0.1949506756
C,0,-5.4613019938,0.6040585349,-1.7801008474
H,0,-5.7187241779,0.3855588642,-2.8258345936
H,0,-5.5631562524,-0.3286848324,-1.2223455522
C,0,-6.4467400032,1.6247712267,-1.174517895
H,0,-6.4423759739,2.5777038164,-1.7256219409
H,0,-6.1452014339,1.8438102071,-0.1415066129
N,0,-7.7833439593,1.0029913772,-1.1229780949
H,0,-8.2221364934,0.8818454155,-2.0371938573
S,0,-8.940651407,1.7473579359,-0.1072192098
O,0,-8.8095626267,3.2232767627,-0.0942484585
O,0,-10.2248715774,1.1184097048,-0.4794011389
C,0,-8.357396798,1.1585632673,1.4996548947
C,0,-7.8116966061,-0.1267367853,1.6413650452
C,0,-8.5028500031,2.0092678787,2.6052300471
C,0,-7.372003472,-0.5407670277,2.9049420806
H,0,-7.7191973456,-0.7729585585,0.7702337998
C,0,-8.0740685351,1.56972064,3.8625057254
H,0,-8.9189765824,3.0061311241,2.4701064234
C,0,-7.4860066606,0.3012415401,4.0303001957
H,0,-6.9357971004,-1.5338036913,3.0237588735
H,0,-8.1747502344,2.2304794783,4.7239258006
C,0,-6.9387539214,-0.1199843806,5.3739615462
H,0,-5.8938869314,0.2144286647,5.4800187556
H,0,-7.5151804024,0.3246744634,6.197967048
H,0,-6.9476750823,-1.2127053339,5.4910481648
C,0,3.3607884236,-1.880830658,5.0319981849
C,0,3.7608613337,-1.8509384786,6.3581829802
C,0,2.961237701,-1.2130063428,7.3433946713
C,0,1.7811799346,-0.5911157749,6.9745497657
H,0,3.9860464687,-2.3670526667,4.2869321591
H,0,4.7034505853,-2.3179005611,6.6464987415
H,0,3.2858319098,-1.2026707774,8.3845413276
H,0,1.166545696,-0.0769136663,7.7156752593

C,0,3.7218192518,-5.5088917827,1.3960629863
C,0,3.2344712804,-6.2502518598,2.4585240381
C,0,2.4296437378,-5.6242163269,3.4470159646
C,0,2.1452582599,-4.2700166222,3.3736470283
H,0,4.3232010277,-5.9805778564,0.6168181456
H,0,3.455163053,-7.3158477148,2.5317963979
H,0,2.026168747,-6.2156744604,4.2699085599
H,0,1.5225336601,-3.8027826077,4.1331559638
C,0,-1.4713458548,0.9092736513,3.5021121107
C,0,-1.4555937333,2.3277523952,3.4097897455
C,0,-2.6631807735,0.1905011565,3.2366863644
C,0,-2.6449393655,2.9922658828,3.0704758138
C,0,-3.8273816276,0.9045331362,2.9010787741
C,0,-3.8382418608,2.3023753281,2.8082206278
H,0,-2.6371451049,4.078472533,2.9865254585
H,0,-4.7500369914,0.3582168821,2.6959169435
C,0,4.0062888327,-1.2462113784,-1.2006725166
C,0,5.0428945335,-0.2881821356,-1.0698490317
C,0,3.3788081537,-1.4555281519,-2.457092649
C,0,5.4576552841,0.4261204787,-2.2055633099
C,0,3.8361961418,-0.7190040584,-3.562824467
C,0,4.8680918357,0.2267417138,-3.4614901742
H,0,6.2545715782,1.1619126752,-2.0965546571
H,0,3.3580197893,-0.8667390261,-4.532390742
C,0,-2.7270349549,-1.3348331285,3.2983267104
H,0,-1.7487771622,-1.7154207104,3.6193746268
C,0,-0.1849475428,3.1345953942,3.6766149943
H,0,0.674206707,2.4747625899,3.4962807527
C,0,-5.1044146939,3.0504211333,2.4179296012
H,0,-5.8761087029,2.2996069214,2.1908455887
C,0,2.2185206673,-2.4360719171,-2.6326534425
H,0,1.7459542312,-2.5768164094,-1.6516555723
C,0,5.7331658905,0.0028929094,0.2622867618
H,0,5.289836567,-0.6350863173,1.0382056083
C,0,5.3415365116,0.9740604371,-4.7029528199
H,0,4.5143455679,0.9499927381,-5.4324248539
C,0,2.7215805526,-3.8077796138,-3.1338853145
H,0,3.4333707046,-4.2667010735,-2.4336256796
H,0,1.8749386478,-4.5002738949,-3.2648949925
H,0,3.2268177698,-3.6968657883,-4.106808723
C,0,1.1284178943,-1.9006270757,-3.5843999817
H,0,0.8889755646,-0.8622942598,-3.3375948796
H,0,1.4534338401,-1.9483292643,-4.6367918539
H,0,0.2162778701,-2.5034532329,-3.4822757883
C,0,6.5485376279,0.2446782982,-5.3354168945
H,0,6.8749102961,0.7511584033,-6.25795224
H,0,7.3958317001,0.2316141545,-4.6318983416
H,0,6.296134109,-0.7978009584,-5.5801566032
C,0,5.6888672348,2.4508187714,-4.4310269584
H,0,4.8631564672,2.9712497207,-3.9253847191
H,0,6.5839796589,2.5372301766,-3.7960632568
H,0,5.9054745274,2.9714956414,-5.3764710794
C,0,5.5205795035,1.4705265201,0.691475351
H,0,5.9835298485,2.1609946666,-0.0308537617
H,0,4.4501856571,1.7060229878,0.7591290499
H,0,5.9831006033,1.6511181058,1.6748492388
C,0,7.2371084894,-0.3395508073,0.1948972487
H,0,7.3911940708,-1.3949884881,-0.076362886
H,0,7.7493765376,0.2809853115,-0.5565663251
H,0,7.7174852177,-0.1572300691,1.1691952881
C,0,-3.0243644046,-1.9405380496,1.9100525311

H,0,-4.0143711217,-1.6166843516,1.5523569815
H,0,-2.2641016717,-1.6461071451,1.1753041985
H,0,-3.03673495,-3.0399322053,1.9743674489
C,0,-3.7717108931,-1.8096501785,4.3295083877
H,0,-3.7783085793,-2.9090563981,4.393320094
H,0,-3.5551612599,-1.4063218967,5.329957132
H,0,-4.7802231598,-1.4800102874,4.0409103458
C,0,-0.0327587015,4.3544765911,2.743989775
H,0,-0.18807264,4.085884565,1.6920747125
H,0,-0.7463006801,5.1520733776,3.005081536
H,0,0.978866016,4.7736132551,2.8497110682
C,0,-0.1374499966,3.6011002786,5.1491169247
H,0,-0.1489153756,2.7519561337,5.8457035037
H,0,0.7767671262,4.1860351796,5.3370340516
H,0,-1.0058603538,4.2400514323,5.3752050137
C,0,-5.618397726,3.9130104635,3.5893210484
H,0,-5.7963662556,3.3012355647,4.4853597168
H,0,-4.8791490101,4.6868041176,3.8487315273
H,0,-6.5580386054,4.4176886272,3.3174587199
C,0,-4.9005494521,3.9264826449,1.1629314747
H,0,-4.4858350878,3.3436374948,0.330344541
H,0,-5.8589774573,4.3631936046,0.8439516766
H,0,-4.2009812735,4.7505660651,1.3714028219

TS-*Re*-A
Opt @ B97D/6-31G** in 1,2-dichloroethane (SMD model, SAS)
SCF Done: E(RB97D) = -4638.62249975 a.u.
Zero-point correction = 1.438041 Hartree/Particle
Thermal correction to Gibbs Free Energy (at 323.15 K) = 1.286094 a.u.
Imaginary Frequency = -154.9248 cm^{-1}
SP @ ωB97XD/def2-TZVPP in 1,2-dichloroethane (SMD model, vdW surface)
SCF Done: E(RwB97XD) = -4641.57318371 a.u.

N,0,-0.7389984461,1.8149252025,-4.3488917651
N,0,-1.9150634853,1.657839067,-5.0039456737
C,0,0.3835805355,2.372638321,-4.965834989
O,0,1.4172554657,2.5789009121,-4.3508091658
O,0,0.1640542213,2.6275482361,-6.2778011837
C,0,-2.6502772897,0.498508219,-4.5862645793
O,0,-3.6280541415,0.0982615766,-5.1982911027
O,0,-2.1448596726,-0.0636774812,-3.4751373188
C,0,1.2969778349,3.2263062134,-6.9683108953
C,0,0.8699962796,3.4519950421,-8.4099397095
H,0,2.1582287975,2.5473128383,-6.8928321244
H,0,1.566292449,4.166582401,-6.4639700812
H,0,1.7023155445,3.8999457703,-8.9727602593
H,0,0.5942623402,2.5017562731,-8.8886736079
H,0,0.0077419924,4.1324186485,-8.4623486676
C,0,-2.8140188337,-1.2477724256,-2.9533349621
C,0,-2.2047790867,-1.531969642,-1.5896057037
H,0,-2.6630832915,-2.0708818206,-3.6675324839
H,0,-3.8909258611,-1.0350471708,-2.8945395818
H,0,-2.7035187228,-2.4071862186,-1.1459182569
H,0,-1.1297599773,-1.7351227886,-1.6712709078
H,0,-2.3369989734,-0.6689852828,-0.9267732073
H,0,-0.5927324543,1.50830589,-3.3706316605
P,0,0.6237811762,1.3561574529,-0.4569716112
O,0,0.8347586802,2.9011040393,-0.2234881413
O,0,0.0345181695,0.8945463147,-1.7433503064
C,0,1.2074188821,-0.3391006693,2.3080919401

C,0,1.73536382,-0.4552706005,3.6459216986
C,0,1.0871239645,0.2541014246,4.7203115114
C,0,-0.0363107626,1.0717433844,4.4287338331
C,0,-0.4900353515,1.2701598143,3.1328311009
C,0,0.1813700422,0.5833142528,2.0756305388
C,0,1.7604055358,-1.1755356907,1.2057208675
C,0,1.8179470835,-2.6127691516,1.2974541057
C,0,2.5054132111,-3.3462875121,0.2620917057
C,0,3.0767959023,-2.6443599467,-0.8344181967
C,0,2.9359601498,-1.271440632,-0.9886065743
C,0,2.2482647794,-0.574138325,0.0488464429
H,0,-0.5566168148,1.5672671135,5.2492656426
H,0,3.6116679007,-3.207941193,-1.5992410688
O,0,2.1169984332,0.8150772664,-0.0776840829
O,0,-0.3118849532,0.7747827945,0.7813548685
C,0,-1.2850802619,4.7306906774,-4.0197712278
C,0,-2.1895476434,4.2654963474,-4.9208774557
C,0,-1.1976675741,4.1617820709,-2.6707179785
C,0,-3.0842958808,3.1496415018,-4.6071305377
C,0,-3.3028143151,2.8865105236,-3.1633191043
C,0,-2.3122970144,3.3020105077,-2.2259861417
O,0,-0.2095671568,4.4536331163,-1.9459539428
H,0,0.3713147582,3.5115720172,-0.9185825501
C,0,-4.2708935622,3.0100378766,-5.5487973759
H,0,-3.9161374465,3.1153855925,-6.5838048711
H,0,-4.7156829382,2.0150516379,-5.4633618735
C,0,-5.3484150099,4.0695822023,-5.2178541414
H,0,-5.080494356,5.0608713171,-5.619016349
H,0,-5.4265667376,4.1718887066,-4.1270218629
N,0,-6.6634806529,3.5990431165,-5.6947261544
H,0,-6.7883353054,3.6539649315,-6.707248862
S,0,-8.0339159225,4.32521972,-4.9648066482
O,0,-7.8505106377,5.7786867483,-4.7465663041
O,0,-9.183765117,3.8202238372,-5.7419227725
C,0,-7.9483681565,3.5167988516,-3.3481813882
C,0,-8.0141802295,2.1170398262,-3.2765174085
C,0,-7.8251630919,4.2993591456,-2.1921449331
C,0,-7.9371306294,1.4991964666,-2.0222174345
H,0,-8.1097239845,1.5283592821,-4.1876340767
C,0,-7.7567652706,3.6630526695,-0.9467188539
H,0,-7.7763523254,5.3836977133,-2.2784108256
C,0,-7.7998892689,2.2596189707,-0.8428721888
H,0,-7.9786728478,0.4107341511,-1.9551927907
H,0,-7.652888726,4.2631701457,-0.0431347625
C,0,-7.6507447541,1.5928723724,0.5064524096
H,0,-6.6161446388,1.6922297208,0.8740240022
H,0,-8.306130274,2.0596206515,1.2573534567
H,0,-7.8915421121,0.5215156472,0.4583578342
C,0,2.8966721992,-1.2246847088,3.9566811875
C,0,3.3662982905,-1.3144734692,5.2569865175
C,0,2.7015895575,-0.6440128151,6.3180246017
C,0,1.585904566,0.1291323832,6.0497013769
H,0,3.4228859282,-1.7373099399,3.1551358653
H,0,4.2609528828,-1.9023536931,5.465713543
H,0,3.079504207,-0.7294117917,7.3374710517
H,0,1.0740253213,0.6676919528,6.8492401979
C,0,2.5849039567,-4.7671290066,0.3499698346
C,0,1.9909428971,-5.4488315118,1.3980205164
C,0,1.2845434228,-4.730667212,2.3991962396
C,0,1.2013486634,-3.3482137462,2.3518059028
H,0,3.1146974452,-5.3067421546,-0.4370964418

H,0,2.0534978996,-6.5363881896,1.4512051452
H,0,0.7982469495,-5.2722849711,3.211492321
H,0,0.653803515,-2.8085488578,3.1213374629
C,0,-1.7017879385,2.1257457418,2.9100727008
C,0,-1.553558665,3.5052173132,2.6121965569
C,0,-2.9935342819,1.5620452531,3.0844955981
C,0,-2.7079198071,4.2856361377,2.4461993677
C,0,-4.1181742646,2.3943625959,2.9269023009
C,0,-3.9965160493,3.7484937762,2.5853449426
H,0,-2.6017471199,5.3408154897,2.1979258413
H,0,-5.1177776494,1.9757696828,3.0511408934
C,0,3.4597999969,-0.5558922615,-2.1910739173
C,0,4.4586240734,0.4469624378,-2.0495213006
C,0,2.9541957892,-0.8720978547,-3.481276224
C,0,4.9101893077,1.1222509983,-3.1917994104
C,0,3.4432724161,-0.1615244127,-4.5904362352
C,0,4.4042308076,0.8474583709,-4.4698495312
H,0,5.6736339197,1.8924584513,-3.0769057433
H,0,3.0443637396,-0.388977935,-5.5807081797
C,0,-3.1734230067,0.0664133711,3.3530169086
H,0,-2.2903715661,-0.2838882655,3.9061156857
C,0,-0.1702937352,4.1510564156,2.5538394111
H,0,0.5418004234,3.3947831431,2.1999226933
C,0,-5.2243252606,4.6212503929,2.3599619377
H,0,-6.1133828747,3.9715694187,2.429827598
C,0,1.8958600996,-1.9472698428,-3.7337773415
H,0,1.5241231705,-2.3124033138,-2.7685349546
C,0,5.1152857441,0.7890737158,-0.7113779854
H,0,4.6231820977,0.2210916907,0.087897174
C,0,4.882285953,1.6280959909,-5.6859100959
H,0,4.261245213,1.3154706484,-6.5432108586
C,0,2.5107196377,-3.1481726068,-4.484082746
H,0,3.3577093854,-3.5804484687,-3.9292758646
H,0,1.7570365751,-3.9372243893,-4.6360297676
H,0,2.8842508381,-2.8384706161,-5.4725497208
C,0,0.6771994051,-1.3884579231,-4.4949369827
H,0,0.2257997341,-0.5697296088,-3.928789007
H,0,0.9538403846,-1.0166505694,-5.4934199107
H,0,-0.0764561373,-2.1802422911,-4.6334587157
C,0,6.3524397952,1.2965627255,-6.0207358936
H,0,6.6846647022,1.8484091688,-6.914568103
H,0,7.008844847,1.5798577217,-5.1831635205
H,0,6.4861457241,0.2200529196,-6.2057503
C,0,4.6955152103,3.1480031637,-5.4875148919
H,0,3.665588071,3.3753436936,-5.1839308174
H,0,5.37084793,3.5197898692,-4.7012946955
H,0,4.9352986866,3.6912087311,-6.4160482694
C,0,4.9796652543,2.2876193393,-0.3685434007
H,0,5.5289720995,2.9089493576,-1.0929050849
H,0,3.9274284627,2.598869114,-0.3737485588
H,0,5.4007456101,2.4867800645,0.62966999
C,0,6.5999476902,0.3610687216,-0.7176447656
H,0,6.7009340742,-0.7162533898,-0.9191682085
H,0,7.160423691,0.9042375547,-1.4941958651
H,0,7.0679581807,0.578371166,0.2557395497
C,0,-3.2150240252,-0.7119887046,2.0166571555
H,0,-4.1039886686,-0.4211619552,1.4341372592
H,0,-2.3261774608,-0.5119653428,1.4067492967
H,0,-3.2735455538,-1.7958643567,2.2042129494
C,0,-4.4116499737,-0.2708781025,4.204899367
H,0,-4.399115796,-1.3382552745,4.4714911534

```
H,0,-4.4371592449,0.3186107046,5.1334347262
H,0,-5.3472645884,-0.0839062621,3.6551013907
C,0,-0.078689729,5.3528197927,1.5943065573
H,0,-0.4773032994,5.1207158581,0.5982215025
H,0,-0.6227782483,6.2254512651,1.9893238831
H,0,0.9739068669,5.6474076603,1.4759300126
C,0,0.2621987654,4.5816816045,3.9745731501
H,0,0.2974257048,3.7236263951,4.6597216773
H,0,1.2628525712,5.0409952635,3.9467893113
H,0,-0.4454969232,5.3205952855,4.3826725715
C,0,-5.3425538634,5.70905762,3.449113349
H,0,-5.3859414101,5.2621810132,4.4533952183
H,0,-4.4728311955,6.3831768936,3.4155287801
H,0,-6.2490316488,6.3158775111,3.2975498298
C,0,-5.2039282714,5.261604373,0.9544700945
H,0,-5.1460879489,4.4989654542,0.1659111573
H,0,-6.1071147085,5.8712387638,0.7939782346
H,0,-4.3334509085,5.9258389209,0.8444772234
H,0,-0.549158957,5.492822771,-4.2747564405
H,0,-2.1948052197,4.6384738741,-5.9452543195
C,0,-4.4546427548,1.70496026,-1.3705379649
C,0,-3.4640162029,2.0975650571,-0.4538866191
C,0,-2.4143067908,2.9094745037,-0.8768664335
C,0,-4.3837113897,2.1015805332,-2.7073452498
H,0,-5.2910666315,1.0895615858,-1.0432276348
H,0,-3.5118752686,1.7869169117,0.5834342638
H,0,-1.6718434031,3.2415177326,-0.1580113185
H,0,-5.1586491989,1.7844647726,-3.3999761916
-------------------------------------------------------------------------------
```

TS-*Re*-B

Opt @ B97D/6-31G** in 1,2-dichloroethane (SMD model, SAS)
SCF Done: E(RB97D) = -4638.61565947 a.u.
Zero-point correction = 1.437423 Hartree/Particle
Thermal correction to Gibbs Free Energy (at 323.15 K) = 1.285095 a.u.
Imaginary Frequency = -155.5869 cm^{-1}
SP @ ωB97XD/def2-TZVPP in 1,2-dichloroethane (SMD model, vdW surface)
SCF Done: E(RwB97XD) = -4641.57041723 a.u.

```
-------------------------------------------------------------------------------
N,0,-0.8284609759,1.5942269275,-4.3045003681
N,0,-1.9786952427,1.4445373669,-4.9948369878
C,0,0.3188902686,2.123895224,-4.8977613399
O,0,1.3348644917,2.3492122846,-4.2615995109
O,0,0.1352422541,2.341075346,-6.2242631492
C,0,-2.6859143304,0.2902818417,-4.536112597
O,0,-2.3219870628,-0.4804009523,-3.6661154806
O,0,-3.8104351649,0.136188915,-5.2764697244
C,0,1.2827051726,2.924732397,-6.9012147827
C,0,0.9055980327,3.079790407,-8.3659077644
H,0,2.1490620499,2.2634212016,-6.7651841857
H,0,1.5211351171,3.8907528809,-6.4311370978
H,0,1.7523587159,3.514126718,-8.9177846095
H,0,0.6605065102,2.1049394989,-8.8107243621
H,0,0.0371574491,3.7445038307,-8.481585903
C,0,-4.7039881189,-0.9255344836,-4.8318512907
C,0,-5.9944381249,-0.7752438262,-5.6236048236
H,0,-4.8632595841,-0.8234687713,-3.7488847137
H,0,-4.2143951836,-1.8932254767,-5.0139815775
H,0,-6.7000382218,-1.5655709186,-5.3275837452
H,0,-6.4650856509,0.2009653767,-5.4353210807
H,0,-5.8048558333,-0.8658104701,-6.7025666762
```

H,0,-0.7233987294,1.2687418204,-3.3243696919
P,0,0.4146483126,1.1456809923,-0.3843988152
O,0,0.4910790477,2.6851706931,-0.0386587971
O,0,-0.2302608362,0.7281584837,-1.6584678418
C,0,1.3754385081,-0.4050667247,2.3499037919
C,0,1.999621208,-0.3677069453,3.6502304601
C,0,1.3636817059,0.3769134138,4.7095158287
C,0,0.1680514735,1.0938250254,4.4358245776
C,0,-0.3818119069,1.1482386478,3.1641516481
C,0,0.2510828568,0.3941277487,2.1334924337
C,0,1.9472338005,-1.2269039496,1.2471422695
C,0,2.1912284274,-2.6389494231,1.3930469395
C,0,2.9040217033,-3.3340193246,0.3485100135
C,0,3.3363718558,-2.6162268932,-0.79989268
C,0,3.0272460718,-1.2766599934,-0.9933617249
C,0,2.2866874642,-0.6236989212,0.0363603228
H,0,-0.3255961794,1.6266977976,5.2487743673
H,0,3.9131857882,-3.1389067098,-1.5636877446
O,0,1.9820823233,0.7285536546,-0.1508506303
O,0,-0.3523901681,0.4057957972,0.8720228849
C,0,-1.2813552534,4.5203217484,-3.9903484036
C,0,-2.1115496486,4.0931453495,-4.9769238631
C,0,-1.3593642335,3.9633039614,-2.6348866976
C,0,-3.0932846653,3.0328257114,-4.7497071302
C,0,-3.4696864331,2.7975485904,-3.3339014575
C,0,-2.5490922498,3.1535068402,-2.3028456209
O,0,-0.4482288675,4.2418793561,-1.8129065784
H,0,0.0582763246,3.2961083038,-0.7453014324
C,0,-4.1743940883,2.9628120566,-5.8164490008
H,0,-3.6974220686,3.0153658502,-6.8053687269
H,0,-4.6994387713,2.008894429,-5.761857762
C,0,-5.2094282917,4.0977743608,-5.6333035337
H,0,-4.8696810687,5.042197973,-6.0886287808
H,0,-5.3539395381,4.2838490044,-4.5604435396
N,0,-6.51302824,3.6438494622,-6.1625711756
H,0,-6.5429000196,3.5877467788,-7.1833083432
S,0,-7.8820894104,4.5588959799,-5.6660536185
O,0,-7.6073202626,6.0140050718,-5.6718853984
O,0,-9.009661414,3.9965985219,-6.4356599646
C,0,-7.9573138722,4.0170488818,-3.9448506095
C,0,-8.3774804875,2.7105992406,-3.6568117313
C,0,-7.5499188327,4.8930268854,-2.9294584882
C,0,-8.3731505496,2.278603745,-2.3248225217
H,0,-8.7049523939,2.0540671565,-4.4617834636
C,0,-7.5515448,4.4423479399,-1.6044697697
H,0,-7.2389815828,5.9051441269,-3.1837320573
C,0,-7.9523681792,3.1308785626,-1.2834491691
H,0,-8.69959573,1.2649403781,-2.0860964022
H,0,-7.2262874694,5.1116609763,-0.8086817597
C,0,-7.8926455006,2.646279229,0.1473923094
H,0,-6.8469722992,2.5667522735,0.4824063854
H,0,-8.3963792722,3.3490541046,0.8278710801
H,0,-8.3649115118,1.6603161039,0.2603086139
C,0,3.2419381587,-1.0095359226,3.9352612981
C,0,3.7989814081,-0.9558533868,5.2027966957
C,0,3.1468805262,-0.2587780338,6.2542476106
C,0,1.9548283203,0.3988152162,6.0064250972
H,0,3.7586967262,-1.5405257452,3.1397551078
H,0,4.7527053899,-1.450148239,5.391999178
H,0,3.5940636176,-0.2309414989,7.2486326084
H,0,1.4518434428,0.959401194,6.7965099566

C,0,3.1629670136,-4.7287490143,0.4905936122
C,0,2.7213345854,-5.4242104509,1.6030192636
C,0,1.9935666085,-4.7474304015,2.6178328641
C,0,1.7361810193,-3.3896095724,2.5166954584
H,0,3.7094766786,-5.2386322496,-0.3049048433
H,0,2.9212720471,-6.4922524127,1.6979381268
H,0,1.6288773271,-5.3023552277,3.483201542
H,0,1.1737088649,-2.8809895658,3.296801542
C,0,-1.6386105778,1.9304365994,2.9321521845
C,0,-1.5823029121,3.3426710907,2.7887259226
C,0,-2.8860467674,1.2609114603,2.9411581231
C,0,-2.7869501215,4.0558947504,2.6844510906
C,0,-4.0645315576,2.0239624218,2.8513481286
C,0,-4.0373801371,3.4185196634,2.7222787575
H,0,-2.7504339041,5.1392247199,2.5726596113
H,0,-5.0299570967,1.5134483833,2.8707183317
C,0,3.4814732024,-0.5395632209,-2.2113082954
C,0,4.412673578,0.5280636725,-2.084015107
C,0,3.0034081763,-0.9130122999,-3.4944719416
C,0,4.834785107,1.2004306679,-3.2387002381
C,0,3.4566140563,-0.1993734673,-4.6172712154
C,0,4.359198247,0.8637952825,-4.513547565
H,0,5.5479101653,2.0190737065,-3.1358489928
H,0,3.0738093535,-0.471006424,-5.6031493587
C,0,-2.9957680239,-0.2605255825,3.0191861183
H,0,-1.9888289119,-0.6758393513,3.1578730318
C,0,-0.2519585993,4.0956478659,2.7814056063
H,0,0.5316588884,3.3898951335,2.4768956597
C,0,-5.3246477573,4.2253594497,2.6193946097
H,0,-6.1651830965,3.5115744241,2.59990816
C,0,1.9983266542,-2.0422662682,-3.7220708579
H,0,1.7066242468,-2.4588535636,-2.7501013689
C,0,5.020823372,0.9555406045,-0.7472131109
H,0,4.5762858454,0.360038903,0.0596262162
C,0,4.805578756,1.6412914806,-5.7433389248
H,0,4.184847514,1.301950231,-6.5907898648
C,0,2.6325740457,-3.1825017772,-4.5472835748
H,0,3.5364422069,-3.5792642277,-4.059047658
H,0,1.916070875,-4.0093508131,-4.6750670198
H,0,2.9232480225,-2.8280005902,-5.5485634702
C,0,0.7079022246,-1.5246927636,-4.3894357956
H,0,0.2148702324,-0.791319838,-3.744298523
H,0,0.9168235636,-1.0584998272,-5.3652301951
H,0,0.0052299955,-2.3559468389,-4.5552625495
C,0,6.2797807267,1.3414212994,-6.0902336044
H,0,6.5916990423,1.8935217577,-6.9913596682
H,0,6.9361817197,1.6457536539,-5.260060034
H,0,6.4358403097,0.2667586989,-6.2680170373
C,0,4.5846095677,3.1587919132,-5.5615410056
H,0,3.5639141745,3.3618862637,-5.2129586031
H,0,5.2835723764,3.5635098715,-4.8133647195
H,0,4.7651625041,3.6937893368,-6.5079559213
C,0,4.7349870379,2.4406827054,-0.4390658541
H,0,5.2175026938,3.0959793462,-1.1809948545
H,0,3.6564880099,2.643078774,-0.4479203498
H,0,5.1341254895,2.7053445864,0.5530629672
C,0,6.539641439,0.6737940105,-0.7271873864
H,0,6.7470002344,-0.3921243594,-0.9069619201
H,0,7.0574383839,1.2549462074,-1.5058870949
H,0,6.9700494259,0.9522741648,0.2479745564
C,0,-3.5522931227,-0.8324204833,1.6967055739

H,0,-4.57678957,-0.4693349775,1.5153403855
H,0,-2.9217099131,-0.5410121685,0.8461684125
H,0,-3.589623721,-1.9321851125,1.7421401264
C,0,-3.8496530686,-0.7130523926,4.2217314848
H,0,-3.876333949,-1.8123005205,4.2785993584
H,0,-3.4396049031,-0.3265496095,5.1669240172
H,0,-4.8873953556,-0.3565846653,4.1316489405
C,0,-0.2237243097,5.2713672737,1.782926138
H,0,-0.5545433765,4.9686037064,0.781548931
H,0,-0.8613971885,6.103655848,2.1207593094
H,0,0.8026654482,5.6585183947,1.7004342034
C,0,0.0846363032,4.6101744381,4.1991746166
H,0,0.167852125,3.7846950935,4.919207731
H,0,1.0410458778,5.1565829335,4.1921092611
H,0,-0.7003920331,5.2966044319,4.5543943141
C,0,-5.5072818962,5.1409947621,3.8489417166
H,0,-5.506754345,4.5568134995,4.7809766406
H,0,-4.6882466662,5.8741500097,3.9083572633
H,0,-6.4567353393,5.6955989467,3.7850101274
C,0,-5.370570849,5.0534225846,1.3176670631
H,0,-5.2592864717,4.4142577976,0.4306808162
H,0,-6.3227213375,5.6028537382,1.2476922765
H,0,-4.5570179664,5.794290809,1.2993633186
H,0,-0.487838462,5.2450501566,-4.1701884927
H,0,-1.9963979859,4.4602007215,-5.9967817608
C,0,-4.8887508692,1.7431924123,-1.657327313
C,0,-3.9623558453,2.0634831795,-0.6485665353
C,0,-2.8085387369,2.7786409459,-0.967673107
C,0,-4.650707454,2.1089523113,-2.9835774213
H,0,-5.8061258244,1.2116820844,-1.4074733708
H,0,-4.1381354226,1.7681634133,0.3817529608
H,0,-2.1064321723,3.0546449738,-0.1844176001
H,0,-5.3812203781,1.8612701164,-3.7495443188

TS-*Re*-C (TS-minor)
Opt @ B97D/6-31G** in 1,2-dichloroethane (SMD model, SAS)
SCF Done: E(RB97D) = -4638.63005719 a.u.
Zero-point correction = 1.438555 Hartree/Particle
Thermal correction to Gibbs Free Energy (at 323.15 K) = 1.288060 a.u.
Imaginary Frequency = -147.7004 cm^{-1}
SP @ ωB97XD/def2-TZVPP in 1,2-dichloroethane (SMD model, vdW surface)
SCF Done: E(RwB97XD) = -4641.57806470 a.u.

N,0,-1.0169635055,1.9253003683,-4.3519526819
N,0,-2.1545594499,1.7431734533,-5.0509748068
C,0,0.0559998534,2.6138104706,-4.9383861935
O,0,0.0919851426,3.0537484245,-6.0723377167
O,0,1.0180319925,2.6945265998,-3.9889663524
C,0,-2.8080617584,0.5061713268,-4.7488038575
O,0,-3.8133259584,0.1337105867,-5.3339467114
O,0,-2.1756807794,-0.1867837876,-3.7817137135
C,0,2.226319748,3.4266551136,-4.3300448314
C,0,2.9078684659,3.7608739835,-3.012617206
H,0,1.950439136,4.3237099089,-4.9038090635
H,0,2.8476322308,2.7785215919,-4.9638630272
H,0,3.8422134696,4.3076216812,-3.2063019424
H,0,2.2495073655,4.3845510132,-2.3928786802
H,0,3.1530389891,2.8425300388,-2.4673498088
C,0,-2.7614687843,-1.4588413317,-3.382596775
C,0,-1.9182111518,-1.9901622931,-2.2337861125

H,0,-2.764517711,-2.1299552766,-4.2544020511
H,0,-3.8048162929,-1.277261199,-3.0864516594
H,0,-2.3507092836,-2.9365326437,-1.8749828372
H,0,-0.8853991953,-2.1743156342,-2.5578898015
H,0,-1.8908325422,-1.2671066366,-1.4093942146
H,0,-0.8828094137,1.5361964319,-3.402258861
P,0,0.4261260068,1.3555425779,-0.5235487147
O,0,0.6095421235,2.9043099287,-0.3105306838
O,0,-0.20274409,0.8583885469,-1.7788155713
C,0,1.1461629693,-0.3330182334,2.2131321858
C,0,1.7266246736,-0.4597525427,3.5278826036
C,0,1.1377324389,0.2679926007,4.6250002309
C,0,0.0310508499,1.1228627066,4.3760845903
C,0,-0.4712389215,1.3274036164,3.0997645466
C,0,0.1232660235,0.5994265864,2.0250885307
C,0,1.6618955785,-1.1476449059,1.077172485
C,0,1.7258536596,-2.5852265179,1.1367246669
C,0,2.4036121013,-3.2962570177,0.0797572594
C,0,2.9937527816,-2.5656324873,-0.9876255557
C,0,2.8609344568,-1.1882325779,-1.1005579394
C,0,2.1333809279,-0.5189860713,-0.0737623028
H,0,-0.4326518018,1.6439795932,5.2143881137
H,0,3.5554269586,-3.1055289236,-1.7503847738
O,0,1.9586191123,0.8653549728,-0.2035170195
O,0,-0.4382919499,0.761138147,0.7550300281
C,0,-1.7397815542,4.7957582787,-3.8896543804
C,0,-2.6150116374,4.3202428595,-4.8149461041
C,0,-1.6044417758,4.1588441164,-2.5807935131
C,0,-3.4396956868,3.1409931051,-4.5581198827
C,0,-3.6169504012,2.7681597769,-3.1349794125
C,0,-2.6489148872,3.1982191254,-2.1789188302
O,0,-0.6240678698,4.4713597107,-1.8477881202
H,0,0.0378878862,3.5103701064,-0.9321781037
C,0,-4.6286419835,2.9903864999,-5.4936977566
H,0,-4.2962914137,3.1758827681,-6.5245693948
H,0,-5.0183920781,1.9700723265,-5.4603871756
C,0,-5.7510331658,3.9759316359,-5.082109186
H,0,-5.5711708645,4.9857039216,-5.486261143
H,0,-5.7694534865,4.0623778936,-3.9870765825
N,0,-7.0623202257,3.4271905263,-5.4804509664
H,0,-7.2369452704,3.4540143684,-6.4872150826
S,0,-8.4279085236,4.1191725206,-4.7041401087
O,0,-8.2997305402,5.5869746006,-4.5515001655
O,0,-9.5920957995,3.5308433898,-5.3964260859
C,0,-8.2199514579,3.3782619866,-3.0675733838
C,0,-8.3415177973,1.9879314992,-2.9270779009
C,0,-7.9221891718,4.1981060071,-1.9704508772
C,0,-8.1418482031,1.4162009046,-1.6647222516
H,0,-8.5787980016,1.3710894048,-3.7925914186
C,0,-7.7292836447,3.6073419825,-0.715875538
H,0,-7.8383850815,5.2748169637,-2.109591593
C,0,-7.8218052419,2.2128700293,-0.5466480922
H,0,-8.2270457791,0.3349218695,-1.5438981439
H,0,-7.4865062176,4.2350552313,0.1410445868
C,0,-7.5293594505,1.592900042,0.8009956365
H,0,-6.462856464,1.7110608634,1.0510222376
H,0,-8.1063144764,2.0822732705,1.6007069975
H,0,-7.7666823038,0.5197520061,0.8148888093
C,0,2.8813251527,-1.2549171655,3.7951603766
C,0,3.3980517707,-1.3547149242,5.0768219757
C,0,2.7906274199,-0.6677903518,6.1612098637

C,0,1.6846099322,0.1320571097,5.9342441705
H,0,3.3646507325,-1.7814655634,2.9759523544
H,0,4.2856610419,-1.9637551285,5.2522289468
H,0,3.2051454281,-0.7611989242,7.1655759297
H,0,1.2179458954,0.684516362,6.7517408893
C,0,2.4764164498,-4.7189768574,0.1323695487
C,0,1.8885673329,-5.4221259766,1.1697580453
C,0,1.1984303586,-4.7247133115,2.196869693
C,0,1.1211501305,-3.3412839278,2.1831904149
H,0,2.9983618978,-5.2425023203,-0.6705812168
H,0,1.9458192501,-6.5108870005,1.1963890187
H,0,0.7204168945,-5.2835518901,3.0023239161
H,0,0.5863835662,-2.8153925825,2.9713149217
C,0,-1.6455405475,2.2335810139,2.8854859667
C,0,-1.4379912904,3.5967651491,2.5432208778
C,0,-2.9588807504,1.7259955826,3.0594572583
C,0,-2.5593127074,4.4051614085,2.3033400336
C,0,-4.0482898391,2.5849604649,2.8195028447
C,0,-3.8695631902,3.9126433278,2.4095775595
H,0,-2.410964724,5.4447247752,2.0145997578
H,0,-5.0650033843,2.2074627966,2.9368357987
C,0,3.5079374001,-0.4398104626,-2.2221053404
C,0,4.5685951553,0.4673083341,-1.9458950006
C,0,3.1112994085,-0.6753459081,-3.5646494611
C,0,5.2144883898,1.1032159241,-3.0174508924
C,0,3.7898341496,-0.0065871075,-4.5988331712
C,0,4.8465790154,0.8795598364,-4.3525653714
H,0,6.0373329654,1.7840156864,-2.798274182
H,0,3.4892489501,-0.1909273941,-5.6319601938
C,0,-3.1992380204,0.2652115087,3.4512554425
H,0,-2.3165099416,-0.0774008115,4.009083889
C,0,-0.0314264588,4.1940014694,2.5116755721
H,0,0.6563750807,3.4171828307,2.1524941084
C,0,-5.056867056,4.8065239482,2.0757386926
H,0,-5.9717735924,4.1987423398,2.1807982747
C,0,1.9864374045,-1.6378616959,-3.9450763072
H,0,1.5072765426,-1.9957243035,-3.0248149071
C,0,5.0859235421,0.7494808538,-0.5338090253
H,0,4.4954324506,0.1732174879,0.1886782611
C,0,5.6212555278,1.5063570021,-5.5063173426
H,0,5.0280611352,1.357177481,-6.4239290744
C,0,2.5471099439,-2.8664090271,-4.6930607022
H,0,3.3049397457,-3.3940066181,-4.0938012898
H,0,1.7379031654,-3.5752613173,-4.9293408909
H,0,3.0213076247,-2.5642168956,-5.6396764775
C,0,0.8957174179,-0.9356279274,-4.7769756253
H,0,0.4528130171,-0.1219140816,-4.1979073553
H,0,1.3006061378,-0.5254961567,-5.7146706172
H,0,0.0997475042,-1.648693168,-5.0406851217
C,0,6.9702420505,0.7737930934,-5.6900291416
H,0,7.5227924791,1.1815586598,-6.5512625986
H,0,7.5959323792,0.8949523479,-4.7919709681
H,0,6.8161679863,-0.3033461188,-5.8517560841
C,0,5.8570694559,3.0204137279,-5.3344346229
H,0,4.9095978914,3.5694979301,-5.2466341841
H,0,6.4563965844,3.2298705033,-4.4354470462
H,0,6.4068307314,3.4199395802,-6.2003490819
C,0,4.9430103531,2.240876608,-0.1620769
H,0,5.5391687816,2.874237128,-0.8372938112
H,0,3.8951436625,2.5620160231,-0.2170021266
H,0,5.3049530834,2.4101563194,0.8642617125

C,0,6.5516926759,0.2847785298,-0.3851077232
H,0,6.6536233604,-0.7852073396,-0.6212279285
H,0,7.2157057395,0.8453887141,-1.0608591167
H,0,6.9016336958,0.448330498,0.6461985213
C,0,-3.3218680655,-0.6376459072,2.2018501199
H,0,-4.2462169661,-0.4054618853,1.6488702651
H,0,-2.4717600902,-0.5052763422,1.5206182544
H,0,-3.3704021224,-1.6966571684,2.5003724818
C,0,-4.4217681901,0.0695824647,4.3689541391
H,0,-4.4586779596,-0.9717802287,4.7226034273
H,0,-4.3790201688,0.7329147006,5.2454625131
H,0,-5.3656998277,0.266596697,3.8372704702
C,0,0.1148334901,5.4114358753,1.578913523
H,0,-0.2979196518,5.2216368471,0.580562406
H,0,-0.3888851702,6.2974564646,1.9975512614
H,0,1.1798075121,5.6617535287,1.4676909272
C,0,0.4001626774,4.589250576,3.943550036
H,0,0.4029301256,3.7236379239,4.6190576555
H,0,1.4146583592,5.0179567181,3.9310941753
H,0,-0.2878737555,5.3451190246,4.3541128934
C,0,-5.160069378,5.9951050682,3.0552957095
H,0,-5.239850308,5.6467414924,4.0957035335
H,0,-4.2682712527,6.6360142116,2.9799752959
H,0,-6.04193317,6.6133788625,2.8254447938
C,0,-4.9763712073,5.3103220356,0.6172541588
H,0,-4.9555329046,4.4741759046,-0.0942492106
H,0,-5.8375169198,5.9552488788,0.3812616325
H,0,-4.0636146936,5.9061483661,0.4656773059
H,0,-1.0518056445,5.6110431253,-4.1109655522
H,0,-2.6403636099,4.7415984343,-5.8196398377
C,0,-4.6620860691,1.4012440292,-1.4103611076
C,0,-3.6971686692,1.8153532745,-0.4763646989
C,0,-2.7105613751,2.7240443165,-0.853118889
C,0,-4.6326205284,1.8786485408,-2.7221512962
H,0,-5.4497569814,0.7106935921,-1.1125396714
H,0,-3.7178482499,1.448719555,0.5431391438
H,0,-1.9927508791,3.0749947651,-0.1173227853
H,0,-5.3902445886,1.5512793805,-3.4288366584

TS-*Re*-D
Opt @ B97D/6-31G** in 1,2-dichloroethane (SMD model, SAS)
SCF Done: E(RB97D) = -4638.62345391 a.u.
Zero-point correction = 1.438823 Hartree/Particle
Thermal correction to Gibbs Free Energy (at 323.15 K) = 1.287609 a.u.
Imaginary Frequency = -158.3589 cm^{-1}
SP @ ωB97XD/def2-TZVPP in 1,2-dichloroethane (SMD model, vdW surface)
SCF Done: E(RwB97XD) = -4641.57610984 a.u.

N,0,-1.0787340144,1.8751444232,-4.3744856492
N,0,-2.200406581,1.7435785244,-5.109357146
C,0,0.0224245169,2.5545556913,-4.9115582074
O,0,0.0940012166,3.022328703,-6.0336004965
O,0,0.9590036792,2.6049713815,-3.9362185924
C,0,-2.8044807845,0.4755835153,-4.8478926926
O,0,-2.3690003952,-0.3861360375,-4.1036418898
O,0,-3.912728153,0.3410828206,-5.6140854062
C,0,2.1873434082,3.3218200517,-4.2375694223
C,0,2.8458990458,3.6206683672,-2.9004734039
H,0,1.9390762792,4.2335708067,-4.8008501333
H,0,2.811526289,2.6738841549,-4.8680966841

H,0,3.7925643096,4.1551032334,-3.0659774538
H,0,2.185395611,4.2437921284,-2.2822561066
H,0,3.0648580664,2.6888892449,-2.3677346701
C,0,-4.7092507939,-0.8510307997,-5.3527218469
C,0,-6.0297722538,-0.6693179344,-6.0866732488
H,0,-4.845501016,-0.9566944301,-4.2668406105
H,0,-4.1515404803,-1.7285031864,-5.7112391673
H,0,-6.6600454595,-1.5574050551,-5.9312696741
H,0,-6.573067598,0.2126787722,-5.7161257733
H,0,-5.8638641752,-0.5464729117,-7.1663258343
H,0,-0.9802003005,1.4195879231,-3.4509188183
P,0,0.3612273083,1.2283278812,-0.5096179828
O,0,0.5090071739,2.7759804089,-0.2404581896
O,0,-0.2896794253,0.7609989394,-1.7633448461
C,0,1.2328088346,-0.3749334207,2.2354879205
C,0,1.8541481779,-0.4197643994,3.5370162849
C,0,1.2688564139,0.337571785,4.6164061653
C,0,0.1334258695,1.1519304012,4.3592618134
C,0,-0.4044033869,1.2844869302,3.0885122343
C,0,0.1696232207,0.5080507512,2.0376889171
C,0,1.757524194,-1.2005932593,1.1122196367
C,0,1.9054816013,-2.6293873698,1.2148689304
C,0,2.5980560191,-3.3367885909,0.1650002391
C,0,3.1286127412,-2.6085583647,-0.9351326835
C,0,2.9218550985,-1.2443110245,-1.0815966853
C,0,2.1674382323,-0.5831812089,-0.0691980625
H,0,-0.3213953781,1.6987315551,5.1856794648
H,0,3.7113729794,-3.1382744857,-1.6893720281
O,0,1.9222237098,0.7834254086,-0.2428664872
O,0,-0.4378971264,0.5767186176,0.7807209354
C,0,-1.7267967131,4.7677968984,-3.8469098721
C,0,-2.5674239479,4.3320343115,-4.819661447
C,0,-1.656829096,4.0939498183,-2.5498375604
C,0,-3.4367657622,3.1698151299,-4.6241801304
C,0,-3.6744975828,2.7629960988,-3.2179101823
C,0,-2.7298015937,3.1372741455,-2.2166215214
O,0,-0.7066923597,4.3771318038,-1.7712614558
H,0,-0.0566439377,3.3826015369,-0.8536036825
C,0,-4.6136670164,3.1283892273,-5.5881903207
H,0,-4.2532236689,3.3448355924,-6.6034755703
H,0,-5.0559460019,2.1325934464,-5.6094680132
C,0,-5.7096099248,4.1329751636,-5.1538422351
H,0,-5.5069476986,5.1496188453,-5.5273519884
H,0,-5.7352351472,4.1856836528,-4.0570610891
N,0,-7.0288295814,3.619859479,-5.5811012765
H,0,-7.1922450037,3.6968522904,-6.5876696848
S,0,-8.3916570217,4.3186908768,-4.7969877288
O,0,-8.2261042004,5.7762419387,-4.595623232
O,0,-9.5577430135,3.779768832,-5.5249635603
C,0,-8.2314434687,3.5182288985,-3.1847024955
C,0,-8.493276683,2.1446791616,-3.0757982442
C,0,-7.8275824683,4.2736768238,-2.0750126971
C,0,-8.3288295988,1.5216189019,-1.8325733343
H,0,-8.8221397333,1.5821044534,-3.948475938
C,0,-7.6719000033,3.6327857331,-0.8404918971
H,0,-7.6398233989,5.3403160807,-2.187853254
C,0,-7.9067733999,2.2516312756,-0.703027978
H,0,-8.5256884316,0.4526388773,-1.7350365773
H,0,-7.3481457968,4.2086002998,0.0256602422
C,0,-7.661793341,1.5733623652,0.6256577491
H,0,-6.5933144452,1.6264018681,0.888576075

H,0,-8.2201481674,2.0677812701,1.435615696
H,0,-7.9573743803,0.5150966567,0.6030903765
C,0,3.0451866383,-1.1584497341,3.8070897393
C,0,3.5977582292,-1.1827698539,5.0774851753
C,0,2.9923689918,-0.4718400773,6.1473486451
C,0,1.8527363234,0.2778863144,5.91533831
H,0,3.5272725825,-1.7026560952,2.9988750458
H,0,4.5120017432,-1.7505582351,5.2547816248
H,0,3.4350219682,-0.5061241728,7.1434987368
H,0,1.387789545,0.8503129894,6.7200514156
C,0,2.7529990235,-4.7504267794,0.2623896872
C,0,2.2324523496,-5.4498341414,1.3377065324
C,0,1.5290994866,-4.7576986563,2.359379481
C,0,1.3713643404,-3.382284136,2.301318607
H,0,3.2848605857,-5.2707803644,-0.5361627147
H,0,2.3531069581,-6.532087829,1.3986939538
H,0,1.1044327681,-5.3144984292,3.1956353412
H,0,0.8268653985,-2.8601435154,3.0853588816
C,0,-1.5929101995,2.1650367622,2.8496365463
C,0,-1.4107506889,3.5412526677,2.5439431936
C,0,-2.8948781653,1.6175365406,2.9537252979
C,0,-2.5462521023,4.3253871468,2.2911472749
C,0,-4.0002641153,2.4511715624,2.6988656667
C,0,-3.8464919777,3.796217935,2.3408653118
H,0,-2.4165328377,5.3765070843,2.037011906
H,0,-5.007883766,2.0355556516,2.7654433612
C,0,3.5449957812,-0.4864901005,-2.2107874626
C,0,4.6060577176,0.4207708604,-1.9395783709
C,0,3.1285811456,-0.7127767255,-3.5469553099
C,0,5.235639476,1.0674220485,-3.0140937397
C,0,3.7920270381,-0.0352566173,-4.5856858893
C,0,4.8495650909,0.8520369318,-4.3458476432
H,0,6.0573972579,1.7518049502,-2.8010889385
H,0,3.4753749623,-0.2104930057,-5.6156196903
C,0,-3.1245226283,0.1485591391,3.3138829907
H,0,-2.1701070154,-0.2676960562,3.6633193199
C,0,-0.0204853602,4.1771601765,2.5505009453
H,0,0.6987430054,3.4124355447,2.2285113984
C,0,-5.0483034608,4.6742528876,2.0158466804
H,0,-5.9453774252,4.0318605035,2.0361314396
C,0,1.9929783801,-1.6684959063,-3.9079756421
H,0,1.529928881,-2.0263159424,-2.9795267225
C,0,5.1277625159,0.699365129,-0.5283752084
H,0,4.5575506216,0.0979536727,0.1902659773
C,0,5.6019796321,1.500184744,-5.5026359807
H,0,5.010039669,1.3342356894,-6.4181358262
C,0,2.533263753,-2.89625398,-4.6719133224
H,0,3.2929449827,-3.4358065955,-4.085284428
H,0,1.7140458758,-3.5949969441,-4.9032729029
H,0,2.9988369369,-2.5914562613,-5.6223063758
C,0,0.8894252788,-0.9554262961,-4.7131068768
H,0,0.4886577283,-0.1232402313,-4.1296091604
H,0,1.2711732623,-0.5693868842,-5.6713544023
H,0,0.0635275317,-1.648764126,-4.9270644855
C,0,6.9723294796,0.8105980059,-5.6922142924
H,0,7.5086835567,1.2357891125,-6.5553569722
H,0,7.5977685086,0.9506184964,-4.7967242149
H,0,6.8513219884,-0.2707754997,-5.8539449107
C,0,5.7909786062,3.0209729411,-5.3288683755
H,0,4.8268183313,3.5403078853,-5.2400578159
H,0,6.383354519,3.2467178181,-4.4291825348

H,0,6.3279106479,3.4393709006,-6.1940926894
C,0,4.9435883519,2.1812265031,-0.1369190077
H,0,5.5104344387,2.8400385373,-0.8130661879
H,0,3.8853884489,2.4695776372,-0.1747084942
H,0,5.3135500196,2.3501626214,0.8867356477
C,0,6.6068691174,0.2755613174,-0.3936601421
H,0,6.7383610236,-0.7876854847,-0.6451711111
H,0,7.2512414124,0.8643516359,-1.0645013442
H,0,6.9576000503,0.4349755258,0.638074658
C,0,-3.5565261977,-0.6767674353,2.0824374479
H,0,-4.5455011486,-0.3497215921,1.7220072703
H,0,-2.8337489691,-0.5757563799,1.2614564459
H,0,-3.6372169058,-1.742704552,2.3472653052
C,0,-4.1420313157,-0.0144182624,4.4617576734
H,0,-4.2219017007,-1.0742201446,4.7487841489
H,0,-3.8398449181,0.5636762035,5.3477034199
H,0,-5.1456317717,0.3246795911,4.1620466892
C,0,0.1223948264,5.3805714557,1.5980559469
H,0,-0.2561471999,5.1608145618,0.5928661508
H,0,-0.4155741937,6.2614869706,1.983762159
H,0,1.1836244517,5.6571853221,1.5142670509
C,0,0.3529098094,4.6164187551,3.9857096255
H,0,0.3608440543,3.7678197209,4.6820587017
H,0,1.3535852787,5.0767292233,3.9953717186
H,0,-0.370568631,5.3594274668,4.3571817199
C,0,-5.2322765106,5.780388,3.0772002297
H,0,-5.3431534403,5.3501779882,4.0835695134
H,0,-4.359015975,6.4505392967,3.0897154075
H,0,-6.1239108686,6.3882571682,2.8568540931
C,0,-4.9284166496,5.2916760132,0.6050041635
H,0,-4.8525809308,4.5133866091,-0.1659286701
H,0,-5.8008271354,5.9271058215,0.3844108052
H,0,-4.0320396679,5.9260210193,0.535185981
H,0,-1.0160920723,5.57604458,-4.0160474134
H,0,-2.5435011055,4.7785702454,-5.8133965493
C,0,-4.817779575,1.3756697226,-1.5751444906
C,0,-3.8742259435,1.7325962441,-0.5972221228
C,0,-2.8477283552,2.6216842724,-0.9092260285
C,0,-4.7282048202,1.8921860789,-2.8685683354
H,0,-5.6328865367,0.6984604431,-1.3253971362
H,0,-3.941114255,1.3327006524,0.4077488522
H,0,-2.1429291907,2.9277247681,-0.1402133177
H,0,-5.475862326,1.6193635836,-3.6092356736
